# Impact of Ligands and Metals on the Formation of Metallacyclic Intermediates and a Nontraditional Mechanism for Group VI Alkyne Metathesis Catalysts

**DOI:** 10.1021/jacs.1c01843

**Published:** 2021-06-10

**Authors:** Richard R. Thompson, Madeline E. Rotella, Xin Zhou, Frank R. Fronczek, Osvaldo Gutierrez, Semin Lee

**Affiliations:** † Department of Chemistry, Louisiana State University, Baton Rouge, Louisiana 70803, United States; ‡ Department of Chemistry and Biochemistry, University of Maryland, College Park, Maryland 20742, United States

## Abstract

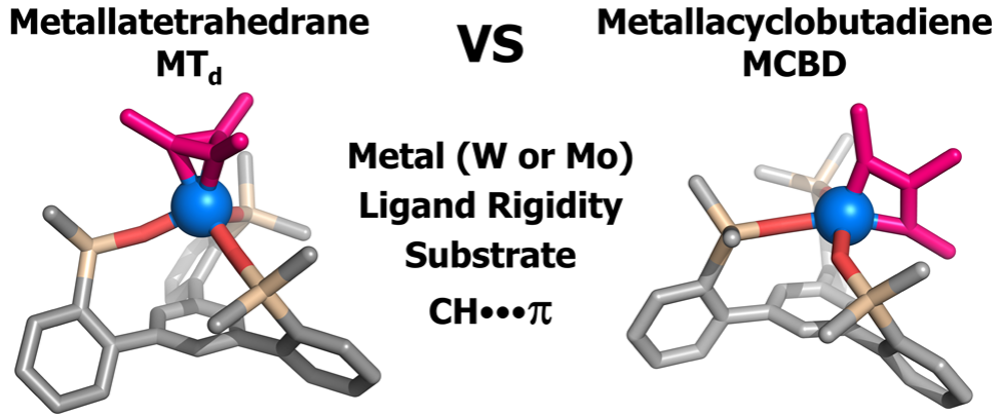

The intermediacy of metallacyclobutadienes as part of a [2 + 2]/retro-[2 + 2] cycloaddition-based mechanism is a well-established paradigm in alkyne metathesis with alternative species viewed as off-cycle decomposition products that interfere with efficient product formation. Recent work has shown that the exclusive intermediate isolated from a siloxide podand-supported molybdenum-based catalyst was not the expected metallacyclobutadiene but instead a dynamic metallatetrahedrane. Despite their paucity in the chemical literature, theoretical work has shown these species to be thermodynamically more stable as well as having modest barriers for cycloaddition. Consequentially, we report the synthesis of a library of group VI alkylidynes as well as the roles metal identity, ligand flexibility, secondary coordination sphere, and substrate identity all have on isolable intermediates. Furthermore, we report the disparities in catalyst competency as a function of ligand sterics and metal choice. Dispersion-corrected DFT calculations are used to shed light on the mechanism and role of ligand and metal on the intermediacy of metallacyclobutadiene and metallatetrahedrane as well as their implications to alkyne metathesis.

## Introduction

**Scheme 1:**
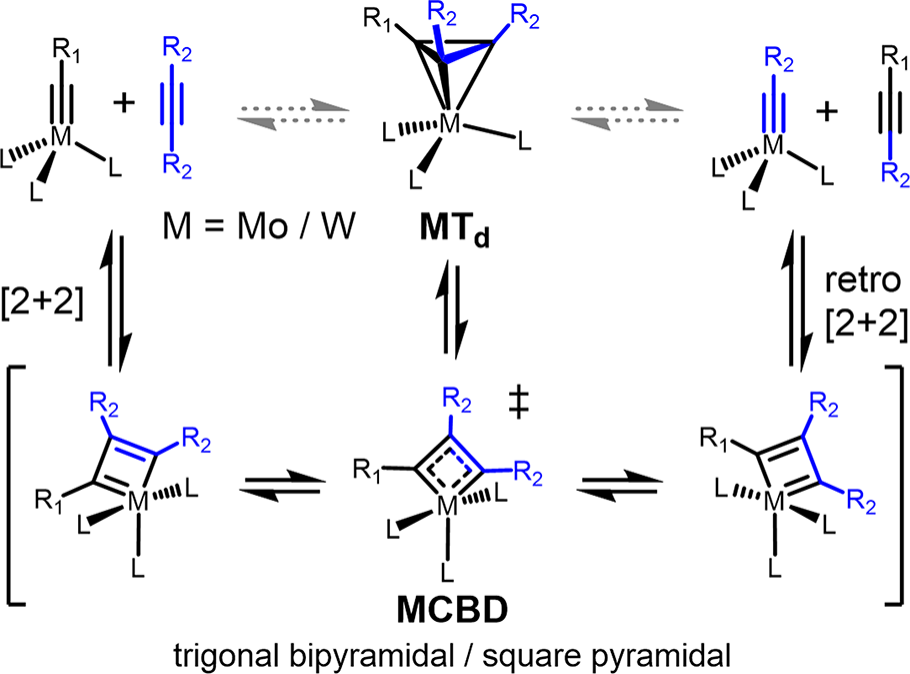
Commonly Accepted Mechanism for Alkyne Metathesis by Way of a MCBD Intermediate MT_d_ formation is considered as an off-cycle intermediate.

The catalytic formation of carbon–carbon bonds remains one of the most crucial applications in organometallic chemistry. Consequentially, cross-coupling, olefin metathesis, and polymerization reactions have all garnered extensive attention with myriad studies on optimizing conditions, improving substrate scope, and expanding the applications of these transformations following their initial discoveries.^1−4^ On the other hand, alkyne metathesis has been a relatively dormant field until recent advances by Bunz,^5^ Fürstner,^6^ Tamm,^7^ Moore,^8^ Zhang,^9,10^ Buchmeiser,^11−13^ and Jia.^14^ These studies have demonstrated that alkyne metathesis is an incredibly powerful tool for generating pharmaceuticals,^15^ complex natural products,^16−24^ supramolecular structures,^25−37^ and polymers.^5,38−44^ The currently accepted mechanism for alkyne metathesis consists of a [2 + 2]/retro-[2 + 2] cycloaddition mechanism (Scheme 1) akin to those implicated in olefin metathesis. Significant evidence for this pathway exists due to the isolation of a number of metallacyclobutadiene (MCBD) intermediates.^45−54^ Typically, the directionality/reversibility of this mechanism is taken for granted as the geometric flexibility of most supporting ligands allows for facile reorganization within the metallacyclic core. Despite the relative paucity of isolated and characterized metallatetrahedrane (MT_d_) isomeric structures, speculation about their intermediacy has lingered since 1982.^55^ Further, computational studies indicate a greater thermodynamic stability for these species in comparison to the more common MCBD.^56,57^ Theory also suggests that direct interconversion between a metal alkylidyne and alkyne with a MT_d_ should be symmetry forbidden and thus implicate the intermediacy of a MCBD en route to the MT_d_.^58^

**Scheme 2:**
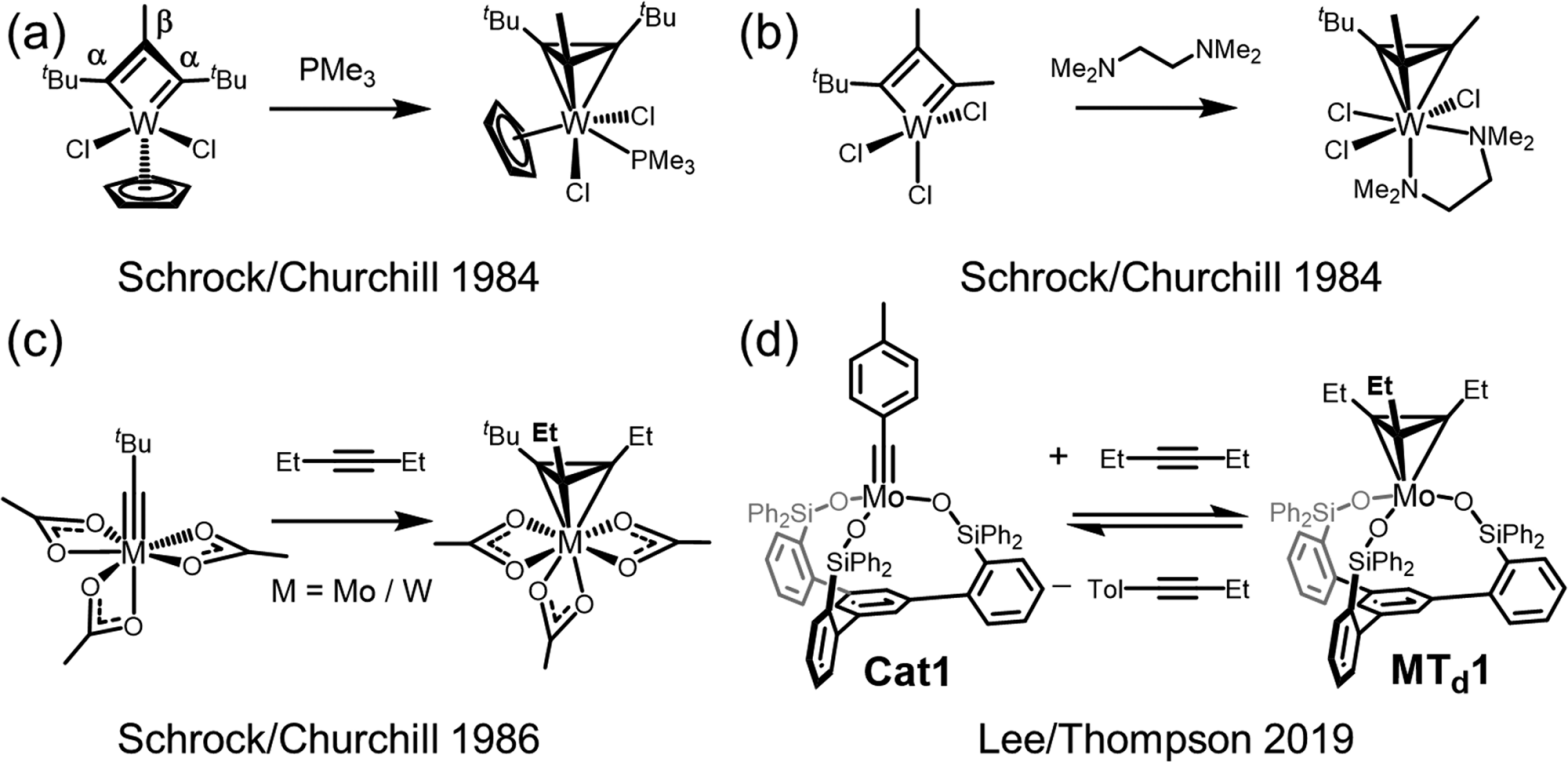
Precedence of Mo(VI)/W(VI)-Based Metallatetrahedranes (MT_d_) Complexes

The first alkyne metathesis-related metallatetrahedrane was reported by Schrock and Churchill in 1984 (Scheme 2a).^53^ They first isolated a bent tungsten metallacyclobutadiene, where the β-carbon protruded out-of-plane. This bent metallacycle was viewed as a structural intermediate between a planar metallacyclobutadiene and a metallatetrahedrane. When trimethylphosphine (PMe_3_) was added, the bent metallacyclobutadiene motif converted completely into a tetrahedral structure. Soon after, another example was reported by coordinating tetramethylethylenediamine (TMEDA) to a W(VI)-MCBD complex, which converted into an octa-coordinate metallatetrahedrane (Scheme 2b).^52^ In 1986, Schrock and Churchill reported yet another metallatetrahedrane that formed directly from Mo(VI)- and W(VI)-alkylidyne complexes when combined with 3-hexyne (Scheme 2c).^51^ These three cases, despite being isolated metallatetrahedranes, have been coordinatively saturated and catalytically inert.^51,52,54,59^ Experimental research on the role of Mo- and W-metallatetrahedranes and their implications to alkyne metathesis remains underexplored and has been almost dormant for more than 30 years.

Beyond mechanistic arguments, studying the stability of early transition metal metallatetrahedranes is important for better understanding fundamental organometallic bonding. While cyclopentadienyl and cyclobutadienyl complexes are well-known, classical organometallic ligands, their 3-member ring analogues are much less well-studied and, even in instances where they have been, it is often as cyclopropenylium cations.^60−62^ As noted by Schrock, the binding of the C_3_ fragments to high-valent, early transition metal centers is significantly stronger than with associated cyclopropenylium adducts and thus can be considered chemically distinct.^52−54^ Furthermore, the tetrahedrane-bonding motif is much more common for the analogous cyclo-P_3_^63,64^ ligand despite phosphorus being considered an elemental “carbon-copy”.^65^ Cummins went as far as to show that treatment of a molybdenum phosphide, (N^*t*^BuAr)_3_Mo≡P, with either diphosphorus (P_2_) or the phosphaalkyne, AdC≡P, resulted in the related cyclo-P_3_ or cyclo-CP_2_ tetrahedranes, respectively.^66^ An improved understanding on the synthesis and stability of metallatetrahedranes could extend its synthetic utility in transferring the cyclopropyl fragment to generate novel tetrahedrane cores as was previously reported by Cummins with a niobium cyclo-P_3_ complex to generate the interpnictogen compound, AsP_3_.^67^

Recently, both our research group^68^ and Fürstner’s^69^ independently reported the same siloxide podand-based ligand scaffold (SiP) for Mo(VI)-alkylidyne catalysts. Fürstner and co-workers identified a number of remarkable improvements in substrate scope (including protic substrates) and moderate tolerance to water using further optimized Mo(VI)-siloxide podand catalysts, which they named “canopy catalysts”.^70^ On the other hand, our group focused on the mechanistic studies of the catalysts and reported the serendipitous discovery of the exclusive formation of a Mo-based metallatetrahedrane intermediate, **MT**_**d**_**1** (Scheme 2d).^68^ The new Mo-metallatetrahedrane complex formed directly from 3-hexyne and the Mo-alkylidyne catalyst supported by the SiP-ligand. In stark contrast to previously reported species,^52−54^ the siloxide podand-based Mo-metallatetrahedrane was identified as *a dynamic intermediate* that interconverts with alkylidynes and continues to behave as an alkyne metathesis catalyst. Given that SiP-supported molybdenum alkylidynes represent an incredible advancement in alkyne metathesis catalyst design, coupled with the fact that **MT**_**d**_**1** was the sole isolated intermediate, we deemed a thorough understanding of metallatetrahedrane formation a paramount question. We recognized that the modular synthesis of the podand ligand could serve as a platform for a systematic investigation on the formation of metallacycles in both Mo(VI) and W(VI) alkylidyne catalysts and, in turn, provide a blueprint for future rational design. Here, we investigate the effect of ligands and metal choice on the intermediate formation and their role in catalysis, both experimentally and computationally.

## Results and Discussion

### Synthesis of Mo/W-SiP Catalysts

**Scheme 3:**
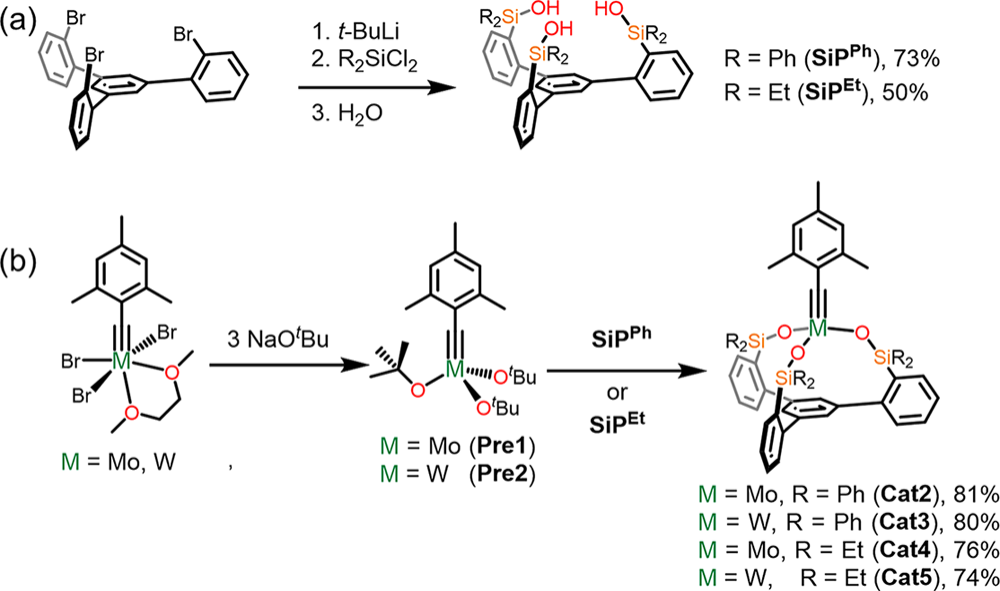
(a) Synthesis of SiP^R^ (R = Ph, Et) Ligands **SiP**^**Ph**^ and **SiP**^**Et**^; (b) Synthesis of Catalysts **Cat2**–**5** via Protonolysis of Catalyst Precursors **Pre1** and **Pre2**

An important feature of the SiP ligand, highlighted by both our group and Fürstner’s, was its rigidity.^68,69^ Specifically, both structural and theoretical probing found the C_3_-symmetric scaffold to enforce the same geometry on organometallic species and that gearing occurred when modulation of one of the podand arms was attempted. This trait is important given the considerable role ligand distortion and geometry play on achieving putative MCBD intermediates.^6,7^ Such distortions would be less necessary if an alternative mechanism involving the experimentally isolated MT_d_ were implicated. With these concepts in mind, we set out to synthesize a new, less sterically imposing, ethyl siloxide podand (**SiP**^**Et**^) ligand to give a direct comparison to the previously reported phenyl variant (**SiP**^**Ph**^)^68,69^ with the idea that structural rearrangements may be more facile. The use of the alkyl substituted variant also allows for the deconvolution of any effects the six aryl groups of **SiP**^**Ph**^ may have on MT_d_ formation. Furthermore, we produced the molybdenum and tungsten catalysts with both ligands to investigate the role of metal choice (Mo vs W) in both activity and more importantly, on the identity and stability of intermediates.^22^ The synthesis of both SiP^R^ ligands, **SiP**^**Ph**^ (R = Ph) and **SiP**^**Et**^ (R = Et), could be achieved in good yield via lithiation of a tribromo-precursor followed by treatment with R_2_SiCl_2_ and aqueous workup (Scheme 3a). A sign of a marked difference between the two ligands is the chemical shift of the O-*H* with those of **SiP**^**Ph**^ coming in at 4.45 ppm while those associated with **SiP**^**Et**^ are upfield by nearly 1 ppm at 3.60 ppm. To further examine the structural differences between the two ligands, we collected X-ray diffraction data on single crystals of **SiP**^**Et**^ (Supporting Information Figure S44) to complement the data which was already collected on **SiP**^**Ph**^. While both compounds crystallized in the space group *P*1̅, **SiP**^**Ph**^ contains a single molecule (as a hydrate) in the asymmetric unit, **SiP**^**Et**^ forms with two independent molecules in the asymmetric unit and no solvent cocrystallizing. In spite of these modest differences, all three silanol OH groups in both independent molecules of **SiP**^**Et**^ are pointing to form intramolecular hydrogen-bonded pockets.

**Figure 1:**
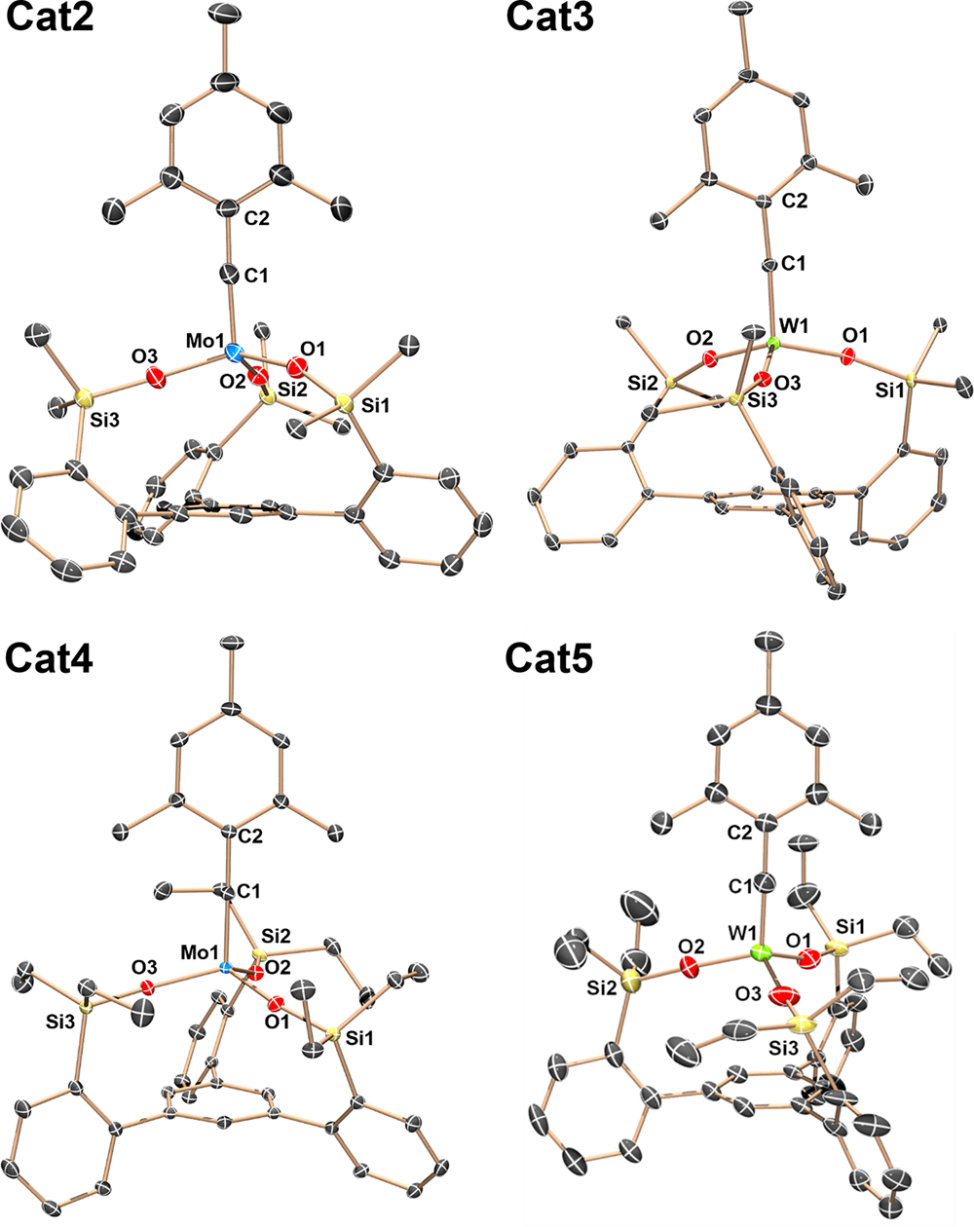
Molecular structures of compounds **Cat2**–**5** at 90 K showing thermal ellipsoids at the 50% probability level with H atoms, solvent, disordered groups, and peripheral phenyl groups omitted for clarity. Mo: light blue, W: bright green, C: gray, O: red, Si: yellow.

Salt-metathesis directly from ArC≡MoBr_3_(dme) (Ar = tolyl and mesityl) was previously shown to be a viable route for appending the SiP scaffold onto molybdenum.^68^ However, we found that protonolysis of the silanol with a precursor tris-*tert*-butoxide species (Scheme 3b) was a much more facile and high yielding method.^69,70^ Specifically, we generated catalyst precursor species with both molybdenum (**Pre1**)^46^ and tungsten (**Pre2**), in high yields as colorless, crystalline solids. Treatment of **Pre1** or **Pre2** with **SiP**^**Ph**^ or **SiP**^**Et**^ allowed for the synthesis of species **Cat2**–**5** in good yields and all readily crystallized when stored at −37 °C (Figure 1, Table 1). In all cases, the SiP ligand was shown to coordinate to the metal center in a mononuclear, tridentate fashion, regardless of the increased Lewis acidity associated with tungsten relative to molybdenum or the reduced sterics of the ethyl groups relative to phenyl groups. The utility of 2,6-disubstitution on the alkylidyne aryl group appears to be particularly useful in preventing the same aggregation observed in related species which lack it.^69,70^ Notably, the M≡C1 (carbyne) distances all fall within the range of typical M-carbon triple bonds.^71^ The most dramatic geometric difference among the four X-ray structures is that the **SiP**^**Et**^-supported species have substantially reduced dihedral angles between the metal carbyne and the siloxide O–Si bonds, likely a result of increased flexibility. Additionally, due to two independent molecules with markedly different bond metrics crystallizing in the asymmetric unit of **Cat5**, we can intuit the increased flexibility associated with the **SiP**^**Et**^ support. While it is tempting to draw a relationship between W–C1 bond length as a function of W–O–Si angle and/or C1–W–O-Si dihedral, in the context of the other W alkylidynes, **Cat3** and **Pre2**, no clear pattern seems to arise. The identity of the R-group on SiP ligands has a modest effect on the electrophilicity of the corresponding alkylidyne, as gauged by ^13^C carbyne chemical shifts. For both the Mo and W systems, there is a ∼6 ppm deshielding of the **SiP^Ph^**derivatives, relative to the **SIP^Et^** analogues.

**Table 1:** List of Bond Lengths (Å), Angles (deg), and Carbyne ^13^C (ppm) for **Cat2**–**5**

	**Cat2**	**Cat3**	**Cat4**	**Cat5**^a^
M–C1	1.746(3)	1.768(3)	1.747(13)	1.743(6)/1.760(5)
M–O_Avg_	1.870(1)	1.873(5)	1.879(5)	1.859(2)/1.859(2)
O–Si_Avg_	1.628(1)	1.632(6)	1.632(6)	1.633(2)/1.657(3)
M–C1–C2	177.0(3)	174.9(3)	174.95(10)	174.3(4)/175.6(4)
M–O–Si_Avg_	167.4(8)	167.7(3)	158.8(3)	171.6(1)/165.5(2)
C1–M–O–Si_Avg_	115.1(3)	121.6(8)	74.4(7)	68.4(1)/57.7(2)
^13^C	313.3	288.4	306.7	281.9

a Two independent molecules in the asymmetric unit.

### Intermediates of Mo(VI) Catalysts

One of the most intriguing findings associated with our previous report was the isolation of a rare metallatetrahedrane (**MT**_**d**_**1**, Scheme 2d) which was found to be *dynamic* in solution.^68^ Due to the SiP ligand’s preference toward *C*_3_-symmetry, as well its presumed rigidity, we were curious if it exclusively supported the formation of metallatetrahedranes or if it would be possible to isolate the more conventional metallacyclobutadiene (MCBD) isomer.

**Scheme 4:**
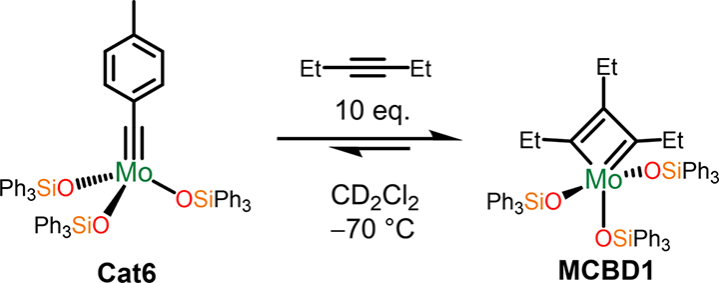
Formation of Mo(OSiPh_3_)_3_(C_3_Et_3_), **MCBD1** Confirmed by NMR at −70 °C.

As a control experiment, we examined the intermediate species that are formed using the nontethered, tris-triphenylsiloxide molybdenum alkylidyne catalyst, **Cat6**. Our hypothesis was that the nontethered three siloxide ligands could more easily reorganize to the geometries associated with MCBD intermediates.^45,72,73^ Furthermore, this experiment would test whether the rigidity of the tripodal siloxide ligand itself is responsible for the formation of MT_d_. CD_2_Cl_2_ solutions of **Cat6**_,_ exposed to 5 equiv to 3-hexyne gave broad ^1^H NMR resonances (Supporting Information Figure S11) indicative of rapid exchange between free and bound alkyne. However, cooling the solution to −70 °C led to sharp ^1^H NMR peaks and the presence of two sets of ethyl resonances ascribed to the new MCBD organometallic species, **MCBD1** (Scheme 4). In particular, the 2:1 ratio of these new ^1^H NMR peaks and distinct ^13^C NMR resonances at 147.2 and 249.8 ppm (associated with the C_β_ and C_α_ respectively) suggested the formation of a C_s_-symmetric MCBD intermediate (Figure S12–S15). Overall, the formation of **MCBD1** implied that *ligand rigidity* and *enforcement of C*_*3*_*-symmetry* may play a crucial role on formation of the metallatetrahedrane over the more conventional MCBD. While this manuscript was under review, Fürstner also reported spectroscopic evidence for **MCBD1**. Further, they were able to isolate crystalline material for this elusive intermediate and scrutinize it via single crystal X-ray diffraction studies to confirm its isomeric identity.^74^

The formation of **MCBD1** from **Cat6**, as a result of *increased ligand flexibility* suggests that geometry and secondary coordination sphere may play a crucial role in the determining the identity of metallacyclic intermediates. We therefore turned our attention toward the impact of ancillary ligand sterics and noncovalent interactions. Interestingly, when **Cat4** was treated with 6 equiv of 3-hexyne, incomplete transfer of the mesityl-carbyne was observed by both ^1^H and ^13^C NMR. Use of 20 equiv of alkyne substrate were required to fully drive such elimination with **Cat4**; however, there were minimal signs of a new organometallic bond. Our attempts to identify the fate of the Mo-containing product were ultimately stymied due to the high concentration of 3-hexyne required to drive the reaction to completion which results in substrate polymerization.

**Figure 2:**
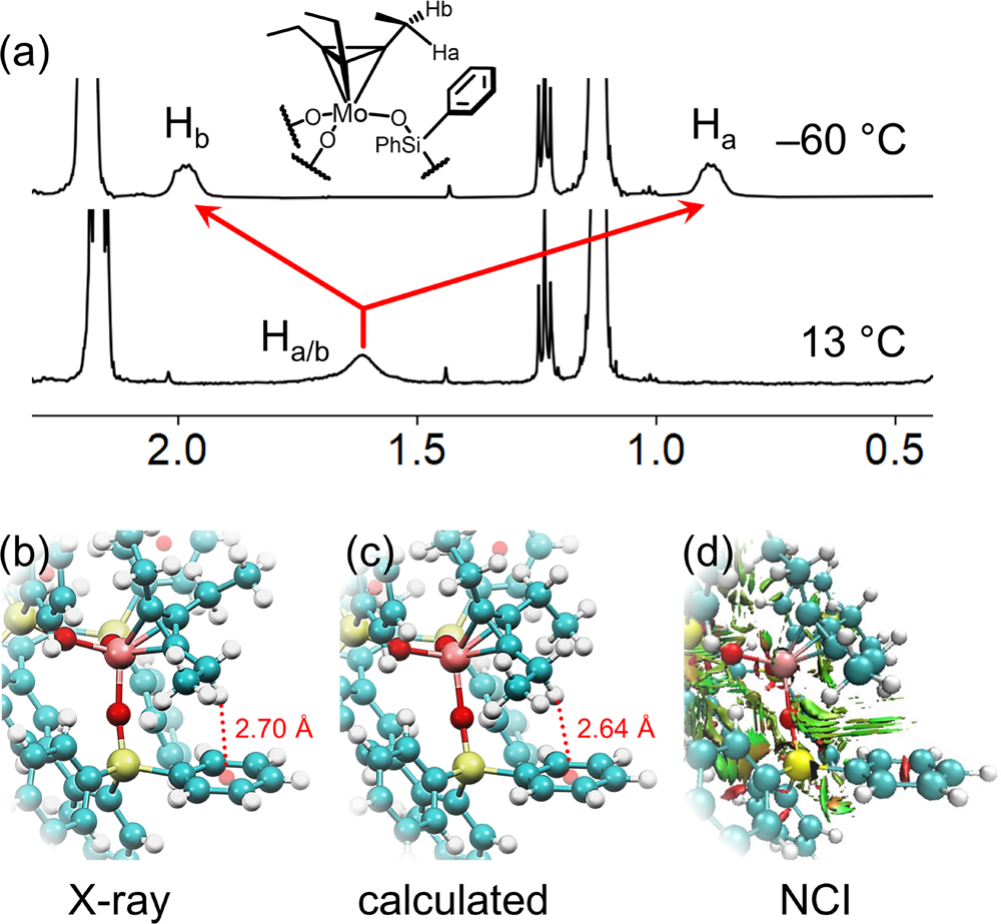
(a) ^1^H NMR of **MT**_**d**_**1** highlighting the upfield shifted methylene proton as a result of CH···π interaction. CH···phenyl_centroid_ distance highlighted in (b) X-ray crystal structure and (c) energy-minimized structure and the corresponding (d) NCI plot of **MT**_**d**_**1**. Green color represents van der Waals interaction.

Since no MT_d_ species were observed using **Cat4** and **Cat6**, we hypothesized that both the *C*_3_-symmetry and phenyl substituents of **SiP**^**Ph**^ and **Cat1** were playing major roles in stabilizing the **MT**_**d**_**1** structure. Here we specifically focused on the potential noncovalent CH···π-interactions between the ethyl groups and the ligand phenyl groups. Spectroscopic evidence for stabilizing CH···π-interactions can be observed when comparing the highly shielded methylene protons of **MT**_**d**_**1** (1.62 ppm) against those reported by Schrock for an MT_d_ with related ethyl substituents but lacking phenyls in the secondary coordination sphere (2.91 ppm).^51^ Furthermore, cooling of the same solution of **MT**_**d**_**1** to −60 °C resulted in a splitting of the diastereotopic methylene protons with chemical shifts centered at 0.88 ppm (H_a_) and 1.98 ppm (H_b_, Figure 2a). This is a result of differing magnetic environments associated with H_a_ interacting with the adjacent π-surface of the phenyl groups on **SiP**^**Ph**^ while H_b_ is not. This dramatic disparity in magnetic environment was further corroborated by simulated ^1^H NMR chemical shifts on an energy minimized structure with chemical shifts of 0.10 and 1.05 ppm for H_a_ and H_b_, respectively (Supporting Information Figure S57).

Further evidence for CH···π-interactions can be found in the solid-state structure of **MT**_**d**_**1** with an average CH_2_···phenyl_centroid_ distance of 2.70 Å for ethyl groups (Figure 2b) which is consistent with observed CH···π interaction distances.^75^ Additionally, the energy minimized structure of **MT**_**d**_**1** also show CH_2_···phenyl_centroid_ distances averaging at 2.64 Å (Figure 2c), which is consistent with the solid-state structure. Finally, noncovalent interaction (NCI) plots show clear signatures of van der Waals interaction between the ethyl groups and the phenyl groups (Figure 2d). Taken together, these results provide strong support for intramolecular CH···π-interactions playing a subtle but crucial role in stabilizing the unique MT_d_ moiety, a design principal which will be further investigated and exploited by our group in the coming years.

**Figure 3:**
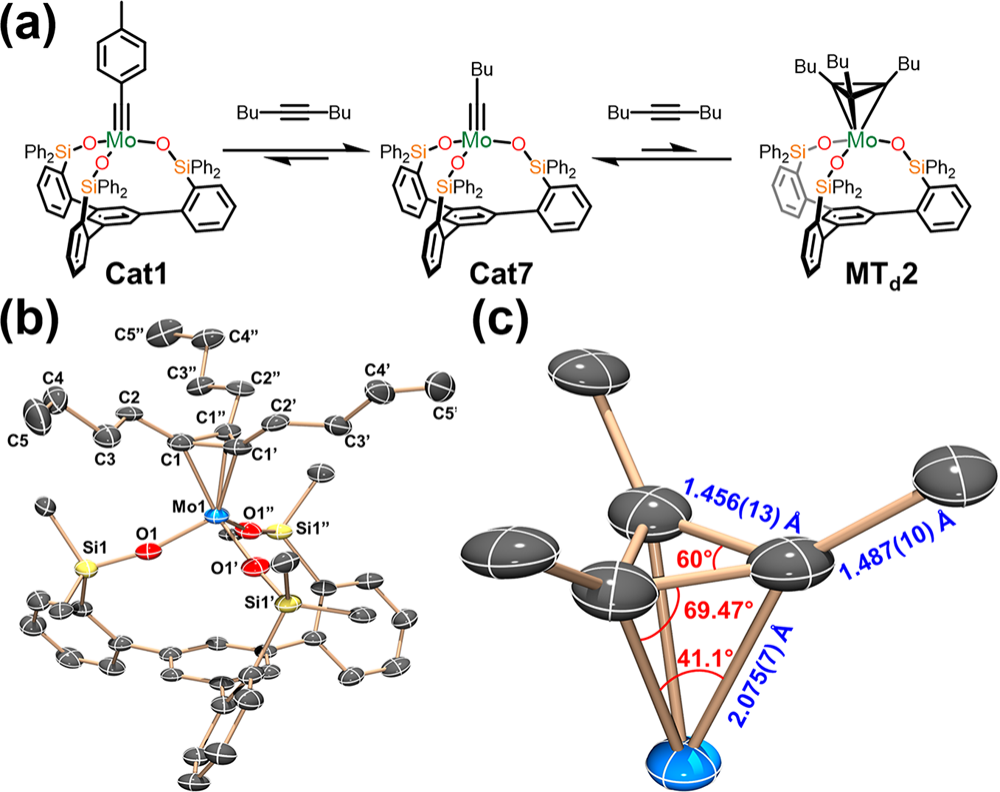
(a) Formation of a pentylidyne (**Cat7**) and a metallatetrahedrane (**MT**_**d**_**2**) using 5-decyne. (b) Molecular structure of compound **MT**_**d**_**2** at 90 K showing thermal ellipsoids at the 50% probability level with H atoms and peripheral phenyl groups omitted for clarity. Mo: light blue, C: gray, O: red, Si: yellow. (c) Local geometry about Mo1 in molecular structure of **MT**_**d**_**2**. Bond distances are colored in blue and bond angles in red.

The use of alternative alkynyl-compounds was investigated to gauge substrate scope for the MT_d_ formation. Treatment of **Cat1** with 6 equiv of diparatolylacetylene failed to produce a triaryl substituted analogue of the MT_d_, presumably due to the increased sterics that lead to high energetic barrier to form this species. Instead, ^1^H NMR revealed the original catalyst to be the only organometallic species visible in solution. Use of the longer chain alkyne, 5-decyne, produced a pentylidyne species (**Cat7**, Figure 3a) which could be cleanly isolated and characterized. Addition of 10 equiv of 5-decyne to **Cat7** allowed for the observation of **MT**_**d**_**2**, as evidenced by a peak at 83.4 ppm in the ^13^C NMR spectrum which corresponds with the *C*_*3*_R_3_ ring of a metallatetrane. Despite the large excess of alkynyl substrate, **MT**_**d**_**2** still exists in equilibrium with **Cat7**. The isolation of **Cat7** and its equilibrium with **MT**_**d**_**2** suggests that, previously unappreciated, alkyl length may contribute to the delicate balance of isolable species supported by the SiP ligand. The importance of chain length was further supported by Fürstner while this manuscript was under review. They reported that an equilibrium mixture of the analogous methyl-substituted MT_d_ and MCBD was observed when the reaction was performed using the shorter chain alkyne, 2-butyne. However, crystallographic characterization of either species was not reported.^74^ To our delight, storage of a concentrated dichloromethane solution of the **MT**_**d**_**2** reaction mixture for 6 months at −37 °C resulted in the serendipitous formation of brown crystalline material which confirmed its identity via X-ray diffraction studies. The solid-state structure of **MT**_**d**_**2** is shown in Figure 3b and 3c. The Mo1-centroid distance of 1.897 Å is comparable to those previously reported for **MT**_**d**_**1** (1.884 Å). Furthermore, the CH_2_···phenyl_centroid_ distance of 2.68 Å again suggests evidence of CH···π interactions as a crucial element in stabilizing the tetrahedrane core. The formation and crystallographic characterization of **MT**_**d**_**2** as only the second ever Mo-metallatetrahedrane suggests the potential for the synthesis of a library of metallatetrahedranes and the privileged role which **SiP**^**Ph**^ plays in supporting these unique species.

### Intermediates of W(VI) Catalysts and Reactivity Studies

**Figure 4:**
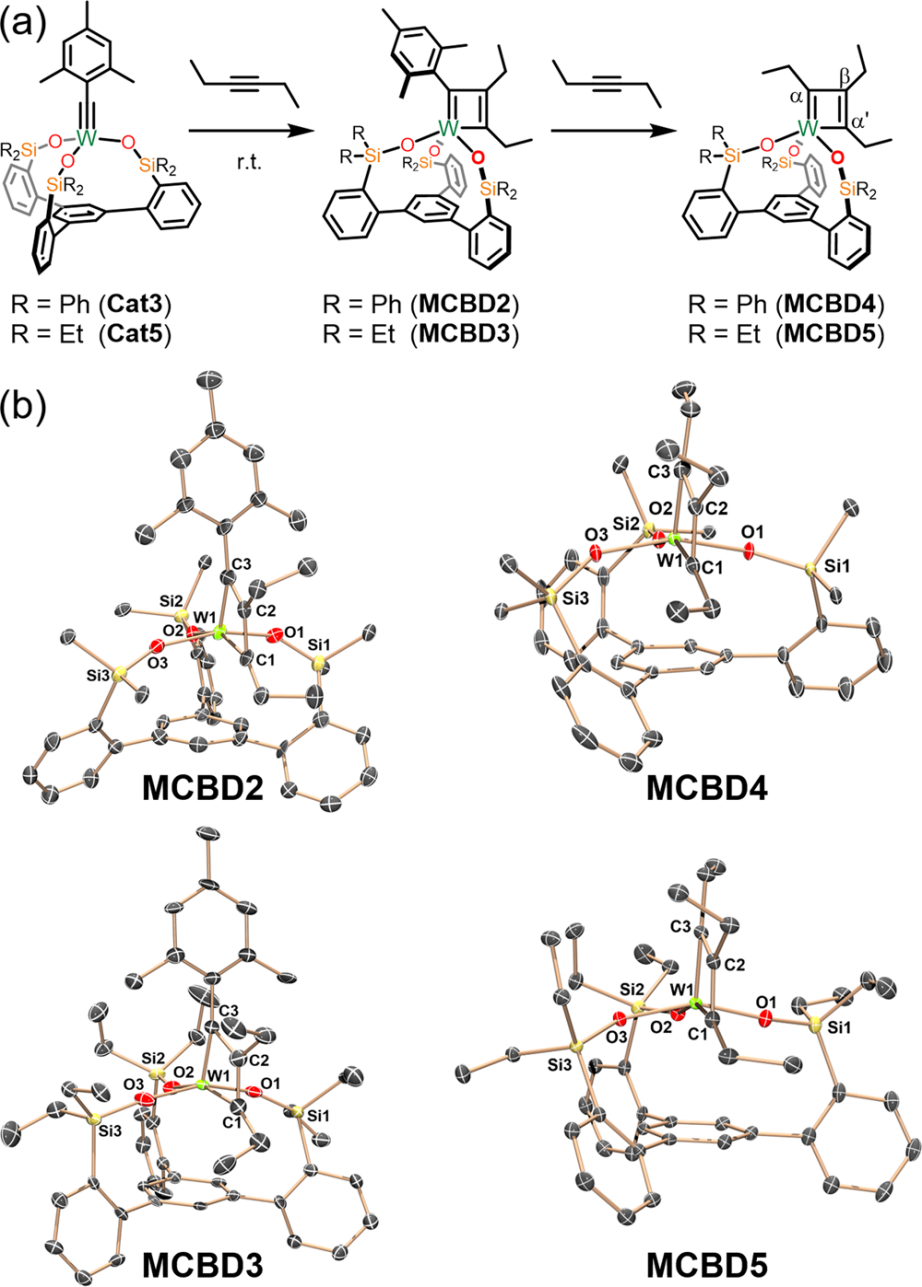
(a) Synthesis of **MCBD2**–**5** from **Cat3** and **Cat5**. (b) Molecular structures of compounds **MCBD2**–**5** at 90 K showing thermal ellipsoids at the 50% probability level with H atoms, solvent and peripheral phenyl groups omitted for clarity. W: bright green, C: gray, O: red, Si: yellow.

In order to investigate the effect of metal selection on the intermediate formation, W(VI)-based catalysts **Cat3** and **Cat5** were treated with a slight excess of 3-hexyne (Figure 4a). Both reactions resulted in an immediate change of color from yellow-orange to purple. Further, the ^1^H NMR spectra of the immediate products indicated that the mesityl group was retained in both cases. These “trapped” MCBD intermediates, **MCBD2** and **MCBD3** were further confirmed by X-ray diffraction studies (Figure 4b) which resemble similar species recently reported by Fürstner.^76^ Storage of C_6_D_6_ solution of **MCBD3** for 3 h at room temperature revealed the elimination of 1-mesityl-1-butyne product as well as the formation of a new organometallic product, **MCBD5**, with three distinct ethyl environments, suggesting the product to be structurally (and chemically) distinct from the *C*_3_-symmetric metallatetrahedrane previously reported. Interrogation of **MCBD5** by ^13^C NMR revealed resonances at 132, 222, and 229 ppm, indicative of the β, α, and α′ ring resonances, respectively, of a metallacyclobutadiene (Figure 4a). The enlistment of 2D NMR techniques allowed for full assignment and correlation of the germane ^1^H and ^13^C resonances. Specifically, NOESY showed a cross peak between the methylene protons of α′ and that of the proximal basal arene protons (Figure S35), itself identifiable by its splitting and coupling from the other two distal arene protons.

The same transformation of **MCBD2** to **MCBD4** could be achieved by heating a C_6_D_6_ solution to 60 °C for 15 min. However, ^1^H NMR spectra of **MCBD4** in CD_2_Cl_2_ at room temperature resulted in very broad peak widths and difficulty locating all of the organometallic resonances. Lowering the temperature to −70 °C allowed us to resolve the ^1^H and ^13^C NMR spectra. **MCBD4** also exhibited C_s_-symmetry with ^13^C resonances of 138, 230, and 235 ppm, indicative of the β, α, and α′ ring resonances, respectively. It should be noted that Fürstner reported the formation of **MCBD4** over the course of 7 days at room temperature, as gauged by ^1^H and ^13^C NMR.^76^

From a mechanistic perspective, we deemed it crucial to not just unequivocally establish the identities of **MCBD4** and **MCBD5** as metallacyclobutadienes via NMR spectroscopy but to analyze the geometry of their primary coordination sphere. As such, single crystals of each were subjected to X-ray diffraction studies (Figure 4b). Gratifyingly, both produced the expected result with each conforming to a “long-short-long-short” bonding motif along the W1–C1–C2–C3 ring (Table 2) indicative of localized π-environments.^45,77^ Despite this bonding mode, there was negligible puckering of the ring (**MCBD4**: 2.58°, **MCBD5**: 1.80°). This observation stands in stark contrast to the fluxional, nonplanar species reported by Schrock^59^ which was thought to be an intermediate between the two isomeric extremes of MCBD and MT_d_. In addition, both **MCBD4** and **MCBD5** assume a decidedly square-pyramidal geometry at W with τ_5_ = 0.04 and 0.10, respectively. This geometry appears to be retained in solution based on the spectroscopic differentiation of α and α′ in ^1^H and ^13^C NMR. This latter point is seemingly unique among previously reported metallacyclobutadienes including **MCBD1** and likely is a result of basal arene enforcing a break in symmetry. Importantly, the isolation of **MCBD4** using the identical **SiP^Ph^** support as was used for **MT_d_1** shows that metal choice has a dramatic effect on the identity of isolable metallacyclic intermediates. The broad NMR resonances associated with **MCBD4** at room temperature, suggested the possibility that it might be unstable to loss of 3-hexyne. Surprisingly, both **MCBD4** and **MCBD5** retained their metallacyclic character upon exposure to a vacuum. It should also be noted that **MT**_**d**_**1** reverted back to **Cat1** under similar conditions.

**Table 2:** List of Bond Lengths (Å) and Angles (deg) for Metallacyclobutadienes **MCBD4**–**6**

	**MCBD4**	**MCBD5**	**MCBD6**
W1–C1	1.9750(16)	1.967(2)	1.963(5)
C1–C2	1.420(2)	1.415(4)	1.413(7)
C2–C3	1.501(2)	1.487(4)	1.504(6)
C3–W1	1.8626(17)	1.865(3)	1.867(6)
W1–C1–C2	76.26(10)	77.04(16)	77.3(3)
C1–C2–C3	121.99(14)	121.41(2)	121.1(5)
C2–C3–W1	78.26(10)	77.04(16)	78.5(3)
C3–W1–C1	83.40(7)	82.65(11)	83.1(2)
τ_5_^a^	0.04	0.10	0.01

a τ_5_ = (β – α)/60° where β > α are the two largest angles at the coordination center.

**Figure 5:**
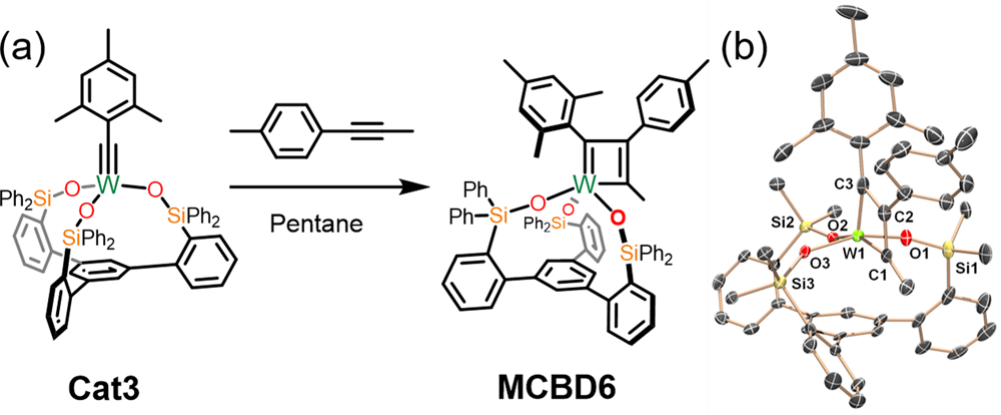
(a) Synthesis of **MCBD6** from **Cat3**. (b) Molecular structure of compound **MCBD6** at 90 K showing thermal ellipsoids at the 50% probability level with H atoms, solvent and peripheral phenyl groups omitted for clarity. W: bright green, C: gray, O: red, Si: yellow.

While 3-hexyne (or related, symmetric alkynes) historically have served as model substrates for understanding the geometry of MCBD intermediates,^77^ we next turned our attention toward the use of asymmetric alkynes to gauge the effect the basal arene has on the selectivity and geometry of substrate approach. Treatment of **Cat3** with *p*-tolylpropyne in pentane (Figure 5a) was performed with the hypothesis that the tolyl group of the substrate would face away from the basal arene to avoid steric repulsion with the ligand scaffold. While ^13^C NMR supported the formation of another MCBD, violet single crystals of the initial intermediate **MCBD6** were successfully obtained and subjected to X-ray diffraction studies for probing the orientation of initial alkyne approach. X-ray analysis (Figure 5b, Table 2) of **MCBD6** revealed it to be another square-pyramidal (τ_5_ = 0.01) metallacyclobutadiene and, as expected, the tolyl group points away from the basal arene. Further analysis of the metallacycle shows the repeat of the “long-short-long-short” bonding motif as well as the exclusive preference of the two aryl groups being in proximity to one another. This preference is presumed to minimize excessive crowding near the basal arene as supported by quantum mechanical calculations (vide infra, Table S6 in the Supporting Information). On the basis of these observations, we anticipate that control of MCBD formation in nonsymmetrical alkynes via steric crowding could lead to the design of highly selective alkyne metathesis catalysts.

### Alkyne Metathesis: Reaction Rates and Substrate Scope

**Figure 6:**
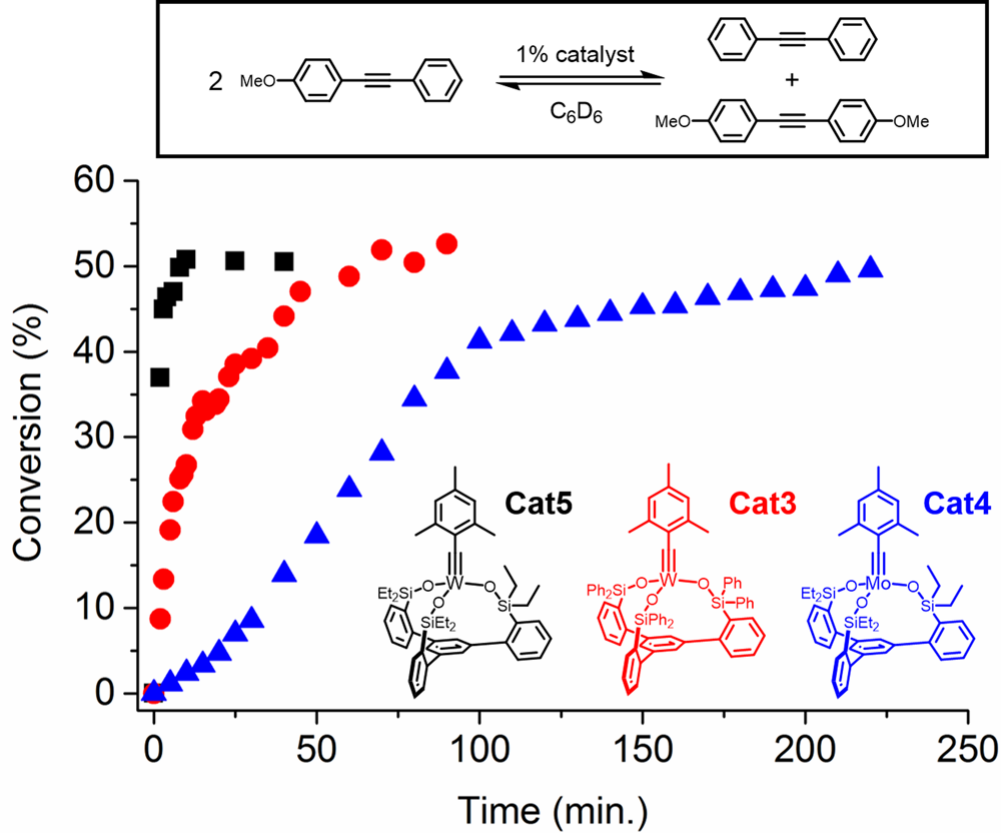
Dynamic scrambling of 1-methoxy-4-(phenylethynyl)benzene (0.1 mM in C_6_D_6_) catalyzed by 1 mol % of **Cat3**–**5** at rt monitored by ^1^H NMR.

Previously, Fürstner reported the improved tolerance of **SiP**^**Ph**^-supported Mo alkylidynes toward highly problematic substrates including primary alcohols, phthalimides, secondary and tertiary amines, among others.^69^ This improvement in group functional tolerance did, however, come at a cost of reduced reaction rates.^68,69^ In parallel to this, we undertook a study to probe the effect both metal choice and ligand sterics have on the dynamic scrambling of the mixed diarylalkyne, 1-methoxy-4-(phenylethynyl)-benzene, at room temperature using 1 mol % of catalysts **Cat1**–**Cat5**. The reaction progress was monitored by ^1^H NMR until it reached equilibrium with a statistical mixture of diarylalkynes (Figure 6). The experiments were performed in sealed NMR tubes (closed system) and the substrate and products were nonvolatile. Therefore, the methoxy (OC*H*_3_) peak integration ratio between the substrate and product served as a useful handle for tracking the reaction. It was already established that **Cat2** was incapable of catalyzing this reaction at room temperature.^68^ We also previously reported that reducing the steric bulk on the alkylidyne by replacing the mesityl group to a tolyl group remedied that problem. The results of the scrambling experiment showed dramatic difference in rates of reaction between the four catalysts with the trend being **Cat5** > **Cat3** > **Cat4** > **Cat1** (Figure 6, see Figure S41 for **Cat4** > **Cat1**). It was clear that the catalysts **Cat4** and **Cat5** with the less bulky ligand were faster at reaching equilibrium compared to **Cat1** and **Cat3**, respectively. The sigmoidal shape of the curve associated with **Cat4** was attributed to an initial inhibition of catalysis due to the steric bulk of the mesityl group, the same culprit which prevented **Cat2** from engaging in catalysis at room temperature. In light of the stability of the initial approach intermediates such as **MCBD2**, **MCBD3**, and **MCBD6**, it was surprising to see that **Cat3** and **Cat5** were more active relative to their Mo(VI) counterparts. In contrast, intermediates derived from Mo(VI) either could not be observed or were found to be much more dynamic. The relative stability of W-MCBD species implies that retro-[2 + 2] reaction would occur slowly in W(VI)-based catalysts.^46^ In addition, while this manuscript was under preparation, Fürstner observed that a similar catalyst with W(VI) was slower in catalyzing a cross-metathesis of an aryl-propyne substrate compared to an isostructural catalyst with Mo(VI).^76^ In contrast, our reaction rates were compared using the scrambling of diarylacetylene substrates (Figure 6). Under similar conditions to those used to observe and isolate **MCBD2** and **MCBD6**, we were not able to observe or isolate any triaryl MCBD intermediates derived from **Cat3** using 1-methoxy-4-(phenylethynyl)benzene as a substrate. These results suggest that there is a significant substrate-dependence on reaction rates in the W(VI)-based catalysts (for a computational study of this substrate-dependence on reaction rates, see Figure S58 and S59 in the Supporting Information).

Historically, while tungsten-based catalysts have been associated with higher reactivity, they have also been found to be less compatible with functional groups due to the heightened electropositivity and oxophilicity. As such, we attempted a small substrate scope study on **Cat5**. Disappointingly, the only substrates which gave acceptable yields were those with highly inert substituents such as *ortho* or *para*-methyl, methoxy, and dimethylamino (Figure S43). Not surprisingly, all carbonyl-containing substrates failed entirely as did phenols. Cyano and thiophenyl species gave very poor yields. Notably, increasing catalyst loading failed to improve the outcomes.

**Figure 7:**
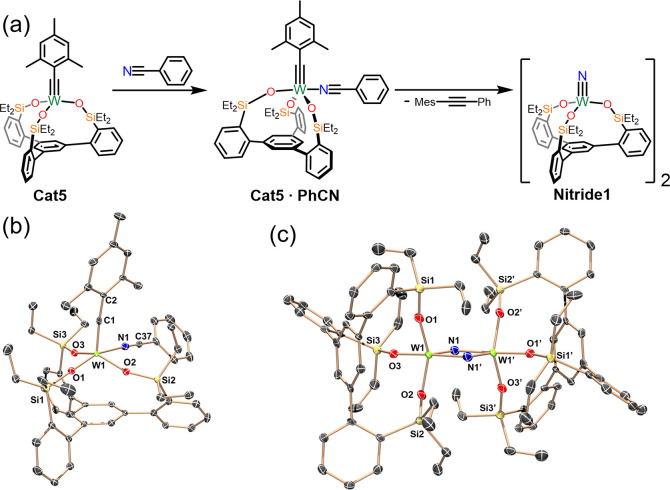
(a) Synthesis of **Nitride1** via nitrile metathesis with **Cat5**. (b) Molecular structure of **Cat5·PhCN** at 90 K showing thermal ellipsoids at the 50% probability level with H atoms omitted for clarity. W: bright green, C: gray, N: blue, O: red, Si: yellow. (c) Molecular structure of **Nitride1** at 90 K showing thermal ellipsoids at the 50% probability level with H atoms and solvent omitted for clarity. W: bright green, C: gray, N: blue, O: red, Si: yellow.

The failure of **Cat5** to provide acceptable yields with cyano-substrates, while disappointing, did suggest the possibility of nitrile metathesis as a means of catalyst deactivation. To better investigate this possibility, **Cat5** was treated with 1.06 equiv of benzonitrile, leading to a purple-red solution which spectroscopically suggested the production of the benzonitrile adduct, **Cat5·PhCN** (Figure 7a,b). An undisturbed pentane solution of **Cat5·PhCN** at room temperature deposited dark purple-red single crystals which unequivocally confirmed this identity (Figure 7b). The metal center adopts the expected 5-coordinate, square pyramidal geometry (τ_5_ = 0.33) which is more distorted than the related Mo-acetonitrile analogue reported by Fürstner (τ_5_ = 0.17).^60^ The W1≡C1 distance of 1.777 Å is elongated relative to both independent molecules of **Cat5** (1.743 and 1.760 Å), while the W1–C1–C2 angle of 171.5° is comparable (174.3° and 175.6°). This relatively linear angle is also distinct from the analogous Mo species which had a noticeable kink of 161.4°.

Storage of a C_6_D_6_ solution of **Cat5·PhCN** for 6 h at room temperature resulted in a new yellow solution as well as the production of phenyl-mesityl acetylene, as gauged by ^1^H NMR. While spectroscopic evidence of the new tungsten species was obfuscated by poor solubility, fortuitous crystals grown from slow-evaporation of the benzene solution gave proof of the dinuclear metal nitride species, **Nitride1** (Figure 7a,c). The formation of metal-nitrides via metathesis of alkylidynes with nitriles has previously been reported by Johnson^78^ and is the microscopic reverse of the initial route by which siloxide-support Mo alkyne metathesis catalysts could be synthesized.^79^ The solid-state structure of **Nitride1** (Figure 7c) reveals it to be dimeric with W1–N1 and W1–N1′ distances of 1.765 and 2.061 Å, respectively, indicative of distinct, localized W double and single bonds. The geometry about each metal center is best described as distorted trigonal bipyramidal (τ_5_ = 0.78) with the shorter W–N1 bond occupying one of the equatorial sites and the longer W–N1′ in the axial position. The trigonal bipyramidal geometry of **Nitride1** is unique among SiP-supported metal compounds as all MCBD intermediates have been decidedly square-pyramidal as was the acetonitrile adduct of the Mo alkylidyne reported by Fürstner.^69^ To our surprise, the related pyridine adduct, (pyridine)(Ph_3_SiO)_3_Mo≡N is both monomeric and decidedly more square pyramidal with a τ_5_ = 0.37.^79^ The differences in geometry between **Nitride1** and the different 5-coordinate Mo species reported by Fürstner, as well as **Cat5·PhCN**, is likely due to the rigid geometric constraints of the SiP ligand (in addition to the N atoms in **Nitride1** functioning as bridging ligands) overriding the strong trans influence of multiply bonded ligands (i.e., nitride, alkylidyne) which prefer occupying the apical position in square pyramidal geometries.

### Quantum Mechanical Calculations

**Figure 8:**
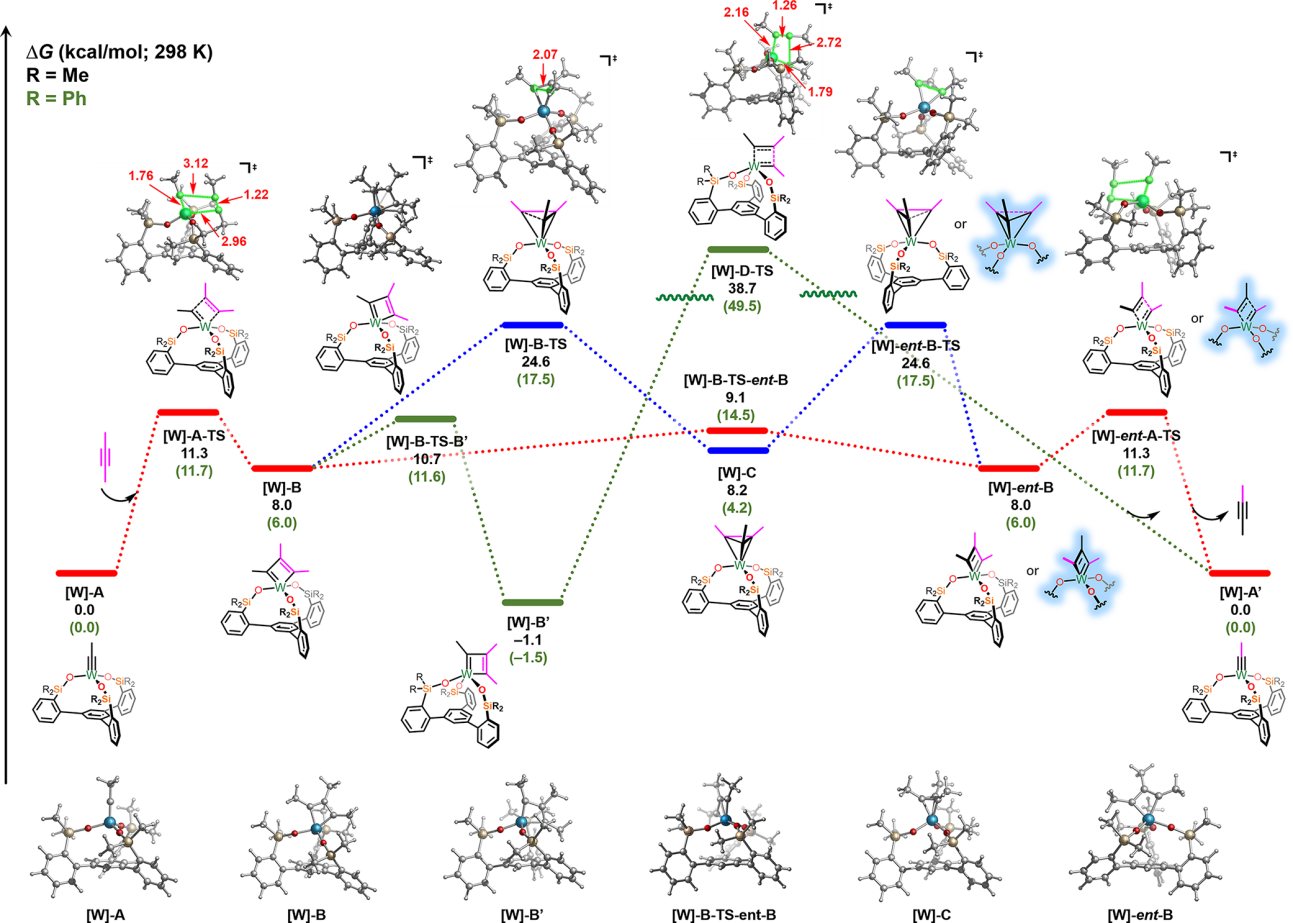
Energetics of MCBD and MT_d_ formation via [2 + 2] cycloaddition for tungsten with **SiP**^**Me**^ (outside parentheses) and **SiP**^**Ph**^ (inside parentheses) ligands. Free energies (kcal/mol) are computed at the B3LYP-D3/def2TZVP-SDD(W)-CPCM(benzene)//B3LYP-D3/def2SVP-LANL2DZ(W)-CPCM(benzene) level of theory. For enthalpy and electronic energies, refer to Figure S60 and S61. The red pathway shows the [2 + 2] cycloaddition followed by pseudorotation and finally retro-[2 + 2] to yield product. Alternatively, the red pathway from the [2 + 2] cycloaddition can lead to the blue pathway in which the metallatetrahedrane is involved in the product formation. The structures highlighted in blue are accessible via the metallatetrahedrane pathway (blue lines). Notably, the green pathway in which the [2 + 2]/retro-[2 + 2] occurs with no pseudorotation is energetically inaccessible.

To better understand the ligand and metal effects on the formation of intermediates, we turned to dispersion-corrected density functional theory calculations [B3LYP-D3/def2TZVP-SDD(M)-CPCM(benzene)//B3LYP-D3/def2SVP-LANL2DZ(M)-CPCM(benzene)] (where M is W or Mo depending on the system being studied; see Supporting Information for computational details and justification for the choice of method).^80−84^ Initially, to reduce computational cost,^85^ the conformationally flexible ethyl groups on the podand ligand (**SiP**^**Et**^) were modeled as methyl groups (**SiP**^**Me**^) and the tolyl substrate was modeled as a methyl. Overall, this method was able to capture the structural parameters of the isolated species, confirming the suitability of our computational method (see Supporting Information Table S7). To explore the ligand effect, we began our analysis by studying the mechanism for alkyne metathesis and formation of MCBD and MT_d_ species of tungsten paired with the **SiP**^**Me**^ and **SiP**^**Ph**^ ligands (Figure 8). In the case of the **SiP**^**Me**^ ligand (black values), the barrier for concerted [2 + 2] cycloaddition to form the symmetrical MCBD **[W]-B** intermediate is only 11.3 kcal/mol (via an early transition state **[W]-A-TS**). In turn, this MCBD could undergo a rapid isomerization (via a pseudorotation of the MCBD moiety with a barrier of 9.1 kcal/mol) to form **[W]-*ent*-B** followed by a retro-[2 + 2] to furnish the desired product (red pathway). These barriers are reasonable with experimental results for which the observed rate of reaction for alkyne metathesis was approximately 1 h. Alternatively, as shown in green, the symmetrical MCBD **[W]-B** could instead isomerize to the most energetically favored (and experimentally observed and characterized; see Figure 4) unsymmetrical MCBD **[W]-B′** (downhill by 9.1 kcal/mol with respect to **[W]-B)** via [**W]-B-TS-B′** (barrier of 10.7 kcal/mol) prior to undergoing alkyne metathesis. We also explored the traditional mechanistic pathway proposed for alkyne metathesis in which the MCBD forms by [2 + 2] cycloaddition and the product is expelled by a retro-[2 + 2] transition state directly from **[W]-B′** to product (Figure 8, green). However, the barrier for this process is found to be prohibitively high in energy (38.7 kcal/mol via [**W]-D-TS**) and therefore energetically inaccessible at the experimental conditions. Consequently, we concluded that this system does not follow the traditional [2 + 2]/retro-[2 + 2] mechanism that has previously been proposed and instead the C_3_-symmetric ligand changes the mechanism to [2 + 2]/isomerization(pseudorotation)/retro-[2 + 2] as observed independently by Fürstner/Neese in parallel to this study^74^ (see Figure S63 in the Supporting Information for a comparison of the energetics). Finally, we explored another possible pathway in which the metallatetrahedrane **[W]-C** was an on-cycle intermediate involved in product formation. As shown in Figure 8 (blue), the MCBD **[W]-B** forms the nearly isoenergetic metallatetrahedrane **[W]-C** directly via a ring-closing transition state (**[W]-B-TS**) with a relative barrier of 16.6 kcal/mol (with respect to **[W]-B**). In this pathway, this symmetrical metallatetrahedrane **[W]-C** can then undergo ring opening followed by retro-[2 + 2] to form the alkyne metathesis product. Overall, while the blue pathway is energetically feasible, the pathway shown in red in Figure 8 is much lower in energy and therefore the most likely mechanism the reaction follows. We also note that these computational results are in accord with experiment where only the thermodynamically more stable MCBD intermediate (akin to **[W]-B′**) was observed and not the (much higher in energy) transient MT_d_ intermediate (akin to **[W]-C**).

Further, to probe the effect of the ligand on this process, the reaction coordinate was then explored for the tungsten **SiP**^**Ph**^ system (Figure 8; green values). Overall, similar energetics were observed for the alkyne metathesis pathway but we observed pronounced effects of the ligand scaffold on the pathway for formation of the MT_d_ intermediate. Specifically, both the overall (17.5 kcal/mol vs 24.6 kcal/mol) and relative (11.5 kcal/mol vs 16.6 kcal/mol) barriers for the MT_d_-formation (via **[W]-B-TS**) are significantly lower and more exergonic (4.2 kcal/mol vs 8.2 kcal/mol) with the more sterically hindered ligand (green values). As such, qualitatively, these results suggest faster and more favorable MT_d_-formation via more sterically hindered **SiP**^**Ph**^ podand ligands.

**Figure 9:**
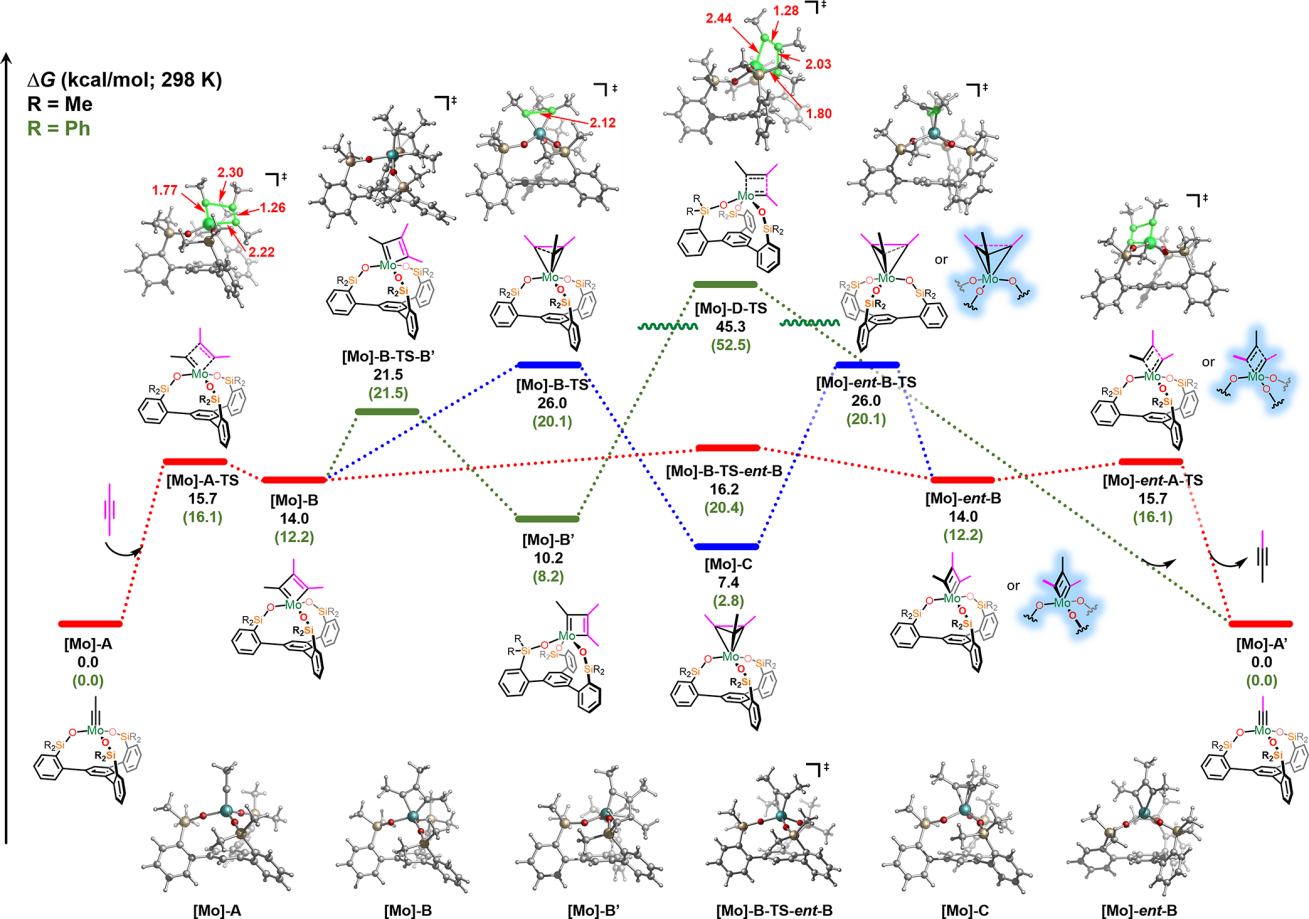
Energetics of MCBD and MT_d_ formation via [2 + 2] cycloaddition for molybdenum with **SiP**^**Me**^ (outside parentheses) and **SiP**^**Ph**^ (inside parentheses) ligands. Free energies (kcal/mol) are computed at the B3LYP-D3/def2TZVP-SDD(Mo)-CPCM(benzene)//B3LYP-D3/def2SVP-LANL2DZ(Mo)-CPCM(benzene) level of theory. For enthalpy and electronic energies, refer to Figure S62 and S63. The red pathway shows the [2 + 2] cycloaddition followed by pseudorotation and finally retro-[2 + 2] to yield product. Alternatively, the red pathway from the [2 + 2] cycloaddition can lead to the blue pathway in which the metallatetrahedrane is involved in the product formation. The structures highlighted in blue are accessible via the metallatetrahedrane pathway (blue lines). Notably, the green pathway in which the [2 + 2]/retro-[2 + 2] occurs with no pseudorotation is energetically inaccessible.

Next we explored the reaction coordinates for the **SiP**^**Me**^ and **SiP**^**Ph**^ ligands for the molybdenum system (Figure 9). The operative mechanism of alkyne metathesis in the case of either ligand for the molybdenum catalyst is relatively the same as that observed for tungsten but proceeds via a flatter surface. Starting with the **SiP**^**Me**^ ligand, **[Mo]-A** undergoes [2 + 2]-cycloaddition via **[Mo]-A-TS** (barrier of 15.7 kcal/mol) to form the very shallow symmetrical MCBD **[Mo]-B** (14.0 kcal/mol) intermediate. The MCBD can then undergo a rapid isomerization (via pseudorotation) with a barrier of 16.2 kcal/mol to form [**Mo]-*ent*-B**, which then undergoes retro-[2 + 2] via to form the desired product. On the other hand, the symmetrical MCBD [**Mo]-B** can also isomerize to the unsymmetrical and more thermodynamically favored MCBD [**Mo]-B′** via **[Mo]-B-TS-B′** (barrier of 21.5 kcal/mol) prior to product formation. However, this unsymmetric intermediate, in contrast to the tungsten systems, is overall uphill in energy by 10.2 kcal/mol compared to the catalyst **[Mo]-A**! These results are in agreement with experimental evidence in which the unsymmetrical (and significantly thermodynamically unstable) MCBD **[Mo]-B′** was not observed. On the other hand, the metallatetrahedrane **[Mo]-C** (7.4 kcal/mol) which forms by ring-closing of **[Mo]-B** via **[Mo]-B-TS** (barrier of 26.0 kcal/mol), is more thermodynamically favored than the MCBD intermediates and is in qualitative accord with experiment in which this intermediate is observed experimentally (**MT**_**d**_**1**, Scheme 2). Notably, the computationally predicted metallatetrahedrane **[Mo]-C** has similar structural features to the X-ray structure (Table S8 in the Supporting Information). It is worth noting that while the metallatetrahedrane **[Mo]-C** with both the truncated ligand and substrate is uphill in energy from **[Mo]-A**, the system studied experimentally (3-hexyne) yields a thermodynamically favorable metallatetrahedrane (see Figure S64 in the Supporting Information). From the metallatetrahedrane intermediate **[Mo]-C**, ring opening via [**Mo]-*ent*-B-TS** followed by retro-[2 + 2] can lead to product formation. However, this pathway (highlighted in blue, Figure 9) is higher in energy than the red pathway ([2 + 2]/isomerization/retro-[2 + 2]) and therefore is likely a secondary route toward product formation. Finally, similar to the tungsten system, we also explored the traditional [2 + 2]/retro-[2 + 2] mechanism in which the MCBD **[Mo]-B** forms product prior to isomerization to **[Mo]-*ent*-B**, but, akin to W-system, we found that the barrier for this pathway is insurmountable and therefore is likely not operative for this system.

We note that these computational results are consistent with experimental observations for the **SiP**^**Ph**^ ligand, in which the barrier to MCBD formation through the [2 + 2] transition state is higher than that of the tungsten system (16.1 kcal/mol vs 11.7 kcal/mol) given that slower reaction rates were observed for alkyne metathesis with the molybdenum system. Akin to the tungsten system, the symmetrical MCBD **[Mo]-B** with the **SiP**^**Me**^ ligand (14.0 kcal/mol) is also energetically favored to isomerize to the unsymmetrical MCBD **[Mo]-B′** (10.2 kcal/mol), albeit this intermediate **[Mo]-B′** is significantly higher in energy (and uphill) than the analogous structure for the tungsten system (**[W]-B′**). Overall, these results are also consistent with experiment in which the MCBD is not observed for molybdenum.

To investigate the steric effects of the ligand, we compared the barriers to metallatetrahedrane formation for the **SiP**^**Me**^ system with the **SiP**^**Ph**^. The destabilization of the **SiP**^**Me**^ molybdenum MT_d_ can partially be attributed to greater steric hindrance as the methyl fragments of the ligand and the substrate are much closer in the case of the **SiP**^**Me**^ ligand. Furthermore, the noncovalent interactions in the **SiP**^**Ph**^ system between the CH of the substrate and the π system of the phenyl ring, which can be observed in the NCI plots (Figure 2d), are responsible for the stabilization of the MT_d_ and the transition state to its formation. However, the other stages of the alkyne metathesis pathway are unaffected by the CH···π interactions, which are not present in the NCI plots of the MCBD (Supporting Information Figure S66). Energy decomposition analysis (EDA) based on the absolutely localized molecular orbitals^86^ (ALMO-EDA) method implemented in Q-Chem 5.0^87^ and described by Liu^89^ was utilized to investigate the specific energetic contributions that control MCBD or MT_d_ formation. Specifically, the energy of the MCBD and MTd intermediates for both metals was decomposed into its energetic components including the Pauli repulsion (Δ*E*_Pauli_), the electrostatic (Δ*E*_elstat_), the polarization (Δ*E*_pol_), and the charge transfer (Δ*E*_ct_) energies using the HF method with the 6-311G(d,p) basis set as employed by Liu.^88^ It was found that Δ*E*_elstat_, or electrostatic energy between the ligand and the alkyne substrate, appeared to contribute to controlling the intermediate formation. Overall, we observed that the Δ*E*_elstat_ was lowest in the case of the favored intermediate (MCBD for W and MT_d_ for Mo; see Figure S67 and S68). Furthermore, the distortion energy^90^ required to convert the geometry of the intermediate into the transition state geometry was also calculated for the MCBD and MT_d_ formation transition states. It was found that the low distortion energy required to form the MCBD in the case of tungsten drives the formation of this intermediate and explains this observed preference while the distortion energy does not appear to play a role in the preference for the MT_d_ in the molybdenum case (see Figure S69 and S70). Instead, for molybdenum it appears to be the favorable CH···π interactions in the MT_d_ intermediate that drive its formation. Overall, the formation of the metallocyclobutadiene or metallatetrahedrane intermediates using these podand ligands appears instead to be dependent on the nature of the metal with the favorable electrostatic energy between the ligand and substrate leading to its preference for the MCBD intermediate in the case of tungsten and the MT_d_ intermediate in the case of molybdenum.

## Conclusions

The metallatetrahedrane has been a scantly investigated organometallic species despite its potential role in alkyne metathesis as well as its similarity to analogous heteroatom-based E_3_ species which are abundant in the chemical literature. This work has shown, both experimentally and computationally, that isolation of such species requires a confluence of myriad and subtle factors including metal choice, supporting ligand rigidity, and the presence of noncovalent interactions. The greater electrophilicity of W (relative to Mo) stabilizes MCBD over MT_d_, regardless of all other factors. The ability of ancillary ligand(s) to more readily distort to accommodate catalyst geometries also leads to a MCBD preference. Finally, CH···π interactions appear to be indispensable in stabilizing the MT_d_. We have also shown that while the greater electrophilicity of tungsten results in faster scrambling of alkyne substrate, this comes at the significant reduction of functional group tolerance. Significantly, the W≡C bond was found to be capable of undergoing metathesis with the C≡N bond of benzonitrile, resulting in bridging nitride species, suggesting that SiP-supported species show potential for catalyzing nitrile-alkyne cross metathesis.

## Supporting Information Available

Experimental procedures, NMR spectral data, and Cartesian coordinates for the calculated structures (PDF)
Crystallographic information (XYZ)

## Supplementary Material

ja1c01843_si_001.pdf

ja1c01843_si_002.xyz

## Accession Codes

CCDC 2027036, 2027039–2027042, 2027044, 2031393, 2031394, 2031597, 2031604, 2060297, 2067843, and 2071696 contain the supplementary crystallographic data for this paper. These data can be obtained free of charge via www.ccdc.cam.ac.uk/data_request/cif, or by emailing data_request@ccdc.cam.ac.uk, or by contacting The Cambridge Crystallographic Data Centre, 12 Union Road, Cambridge CB2 1EZ, UK; fax: +44 1223 336033.
