# Supplementary material for: Impact of Ligands and Metals on the Formation of Metallacyclic Intermediates and a Nontraditional Mechanism for Group VI Alkyne Metathesis Catalysts
Source: J Am Chem Soc. 2021 Jun 10;143(24):9026–39. doi: 10.1021/jacs.1c01843 (PMC8227475; doi:10.1021/jacs.1c01843)
Supplement: Supplementary file 1 — ja1c01843_si_001.pdf [file ja1c01843_si_001.pdf]

Supporting Information
for the
Article Entitled

Impact of Ligands and Metals on the Formation of Metallacyclic Intermediates and a Non-traditional Mechanism for Group VI Alkyne Metathesis Catalysts

Authored by

Richard R. Thompson, Madeline E. Rotella, Xin Zhou, Frank R. Fronczek, Osvaldo Gutierrez, Semin Lee

Department of Chemistry
Louisiana State University, Baton Rouge, Louisiana 70803

Department of Chemistry and Biochemistry
University of Maryland, College Park, Maryland 20742

TABLE OF CONTENTS

Experimental Details S2
General considerations S2
Synthesis of new compounds S2
Supporting NMR Spectra S10
Rate of Alkyne Metathesis using Mo(VI) and W(VI)-based SiP Catalysts S39
Substrate Scope using Cat5 ..... S41
Crystallographic Information S41
Computational Details S52
References S93

Experimental Details

General considerations

All manipulations were performed under an inert atmosphere of Ar using standard Schlenk-line or glovebox techniques. Anhydrous n-pentane, toluene, diethyl ether and tetrahydrofuran were purchased and dried by passage through two columns of activated alumina and a Q-5 column. Benzene-d_6 was degassed by three cycles of freeze-pump thaw and then stored over 4 A molecular sieves. Celite and molecular sieves were dried under reduced pressure at 150 °C for three days. Cooling for the reactions was performed in the internal freezer (-37 °C) of the glovebox used. $Mo(CO)_6$, $W(CO)_6$, 2-bromoacetophenone, oxayl bromide, 1,2-dimethoxyethane, tetramethylammonium bromide, 4-bromotoluene, 2-bromomesitylene, 3-hexyne, 5-decyne, NaO^tBu, Ph_2SiCl_2 and Et_2SiCl_2 were all purchased from Acros Organics and used as received. $C_6H_3(C_6H_4Br)_3$,[1] alkynyl substrates,[2] Mo(≡CMes)Br_3(DME),[3] W(≡CMes)Br_3(DME),[3] **Pre1**,[4] **SiP^{Ph}**[5], **Cat1**[5] , and **Cat6**[5] were synthesized via previously reported methods. ^1H and $^{13}C\{^1H\}$ NMR spectra were recorded on either Varian 400 MHz or 500 MHz NMR spectrometers with benzene-d_6 referenced at 7.16 and 128.06 ppm, respectively. Low-temperature NMR spectra were collected with dichloromethane-d_2 referenced at 5.32 and 53.84, respectively.

Synthesis of new compounds

$C_6H_3(C_6H_4SiEt_2OH)_3$ (SiP^{Et})

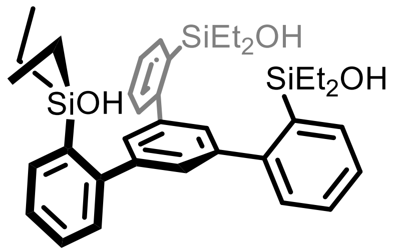

To a solution/suspension of $C_6H_3(C_6H_4Br)_3$ (1.00 g, 1.84 mmol) in 150 mL ether was dropwise added *t*-BuLi (1.8 M, 7.2 mL) at –78 °C. The solution immediately formed precipitates while adding (1-2eq). After further addition (5-7eq) the precipitates disappeared and formed a transparent yellow-brown solution which gets darker with time. The solution was stirred at –78 °C for 4.5 h. Et_2SiCl_2 (0.9 ml, 6.07 mmol) was added dropwise to the reaction mixture resulting in a red-purple solution. The cooling bath was removed and the reaction mixture was allowed to warm to rt and stirred overnight. Large amount of salt precipitates form at rt and the red/brown solution turns to light yellow. The reaction mixture was cooled using an ice bath. The reaction was quenched with water (~30 mL) and the ice bath was removed. The reaction was stirred at rt for 30 min. The mixture was extracted with ether (× 3) and washed with brine. The organic phase was collected and dried with Mg_2SO_4, then filtered. The solvent was removed under reduced pressure to give a yellow oil. The oil gradually crystallizes over time and samples suitable for X-ray diffraction crystallography were grown by a concentrated pentane solution at –30 °C. Yield = 0.565 g (50%). ^1H NMR (400 MHz, $CDCl_3$, 25 °C): 0.76 (12H, q, $^3J_{HH}$ = 7.6 Hz, SiCH_2CH$_3$), 0.87 (18H, t, $^3J_{HH}$ = 7.6 Hz, SiCH$_2$CH_3), 3.60 (3H, br s, SiO*H*), 7.23 (3H, s, C$_6$*H*$_3$), 7.32 (3H, t, $^3J_{HH}$ = 7.6 Hz, Ar-*H*), 7.34-7.41 (6H, m, Ar-*H*), 7.48 (3H, d, $^3J_{HH}$ = 7.2 Hz, Ar-*H*). $^{13}C\{^1H\}$ NMR (125.77 MHz, C_6D_6, 25 °C): 6.91 (SiCH$_2$*C*H$_3$), 8.28 (Si*C*H$_2$CH$_3$), 126.43 (*Ar*), 127.72 (*Ar*), 128.95 (*Ar*), 129.94 (*Ar*), 134.68 (*Ar*), 136.00 (*Ar*), 144.20 (*Ar*), 149.08 (*Ar*). HRMS-ESI (m/z): calcd. for $C_{36}H_{49}O_3Si_3^+$, $[M + H]^+$, 613.2984, found, 613.3002.

(^{t}BuO)$_3$W≡CMes (Pre2)

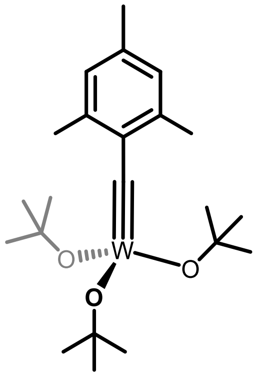

W(≡CMes)Br_3(DME) (495 mg/0.75 mmol) was dissolved into 10 ml of THF and NaO^{t}Bu (229 mg/2.38 mmol) was dissolved in 5 ml of THF and both solutions were chilled to –37 ^{o}C. The colorless solution of NaO^{t}Bu was dropwise added to the stirring, green solution of W(≡CMes)Br_3(DME), resulting in the solution becoming yellow-brown and the precipitation of KBr by-product. The reaction stirred at room-temperature for 0.5 h at which point solvent was removed under reduced pressure, the product extracted into 10 ml of pentane and the KBr impurity removed by filtering through 3” celite plug on a medium porosity fritted funnel. Removal of all volatile resulted in material pure enough for subsequent chemistry, storage of a concentrated pentane solution at -37 °C resulted in single crystals suitable for X-ray diffraction studies. Yield (of crystalline material) = 204 mg (51.0%). ^{1}H NMR (500 MHz, C_6D_6, 25 °C): 1.43 (27H, s, C(C*H*$_3$)$_3$), 2.27 (3H, s, *p*-C*H*$_3$), 2.93 (6H, s, *o*-C*H*$_3$), 6.87 (2H, s, *o*-$C_6H_2Me_3$). ^{13}C{^{1}H} NMR (125.77 MHz, C_6D_6, 25 °C): 271.33 (W≡*C*), 143.2 (*Ar*), 140.6 (*Ar*), 135.1 (*Ar*), 127.6 (*Ar*), 79.6 (*C*(CH$_3$)$_3$), 32.2 (C(*C*H$_3$)$_3$), 21.7 (*o*-*C*H$_3$), 20.9 (*p*-*C*H$_3$).

[C_6H_3($C_6H_4SiPh_2O$)$_3$]Mo≡CMes (Cat2)

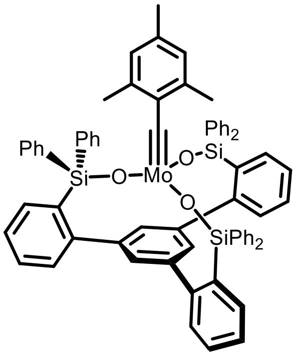

Mo(≡CMes)Br_3(DME) (1.00 g/1.80 mmol) was dissolved into 10 ml of THF and NaO^{t}Bu (0.518 g/5.39 mmol) was dissolved in 10 ml of THF and both solutions were chilled to -37 ^{o}C. The colorless solution of NaO^{t}Bu was dropwise added to the stirring, green solution of Mo(≡CMes)Br_3(DME), resulting in the solution becoming yellow-brown and the precipitation of KBr by-product. The reaction stirred at room-temperature for 0.5 h at which point solvent was removed under reduced pressure, the product extracted into 10 ml of pentane and the KBr impurity removed by filtering through 3” celite plug on a medium porosity fritted funnel. The volatiles were removed under reduced pressure and the resulting off-white solids were redissolved into 10 ml toluene and **SiP^{Ph}** (1.62 g/1.80 mmol) was added directly to the solution, resulting in a color change from yellow to orange. After stirring for 10 min. the volatiles were removed under reduced pressure. Single crystals (orange plates) suitable for X-ray diffraction studies were grown from slow-evaporation of dichloromethane orange, pure powder was obtained by chilling a concentrated toluene solution to -37 ^{o}C. Yield = 1.64 g (81.0%). ^{1}H NMR (500 MHz, C_6D_6, 25 °C): 2.14 (6H, s, *o*-C*H*$_3$), 2.16 (3H, s, *p*-C*H*$_3$), 6.48 (2H, s, C_6*H*$_2$Me$_3$), 6.90 (3H, d, $^3J_{HH}$ = 10.0 Hz, Ar-*H*), 7.03-7.10 (24H, m, Overlap of several Ar-*H*), 7.28 (3H, s, C_6*H*$_3$Ar$_3$), 7.76 (3H, d, $^3J_{HH}$ = 10.0 Hz, Ar-*H*), 7.83 (12H, d, $^3J_{HH}$ = 10.0 Hz, *o*-SiPh$_2$). ^{13}C{^{1}H} NMR (125.77 MHz, C_6D_6, 25 °C): 288.42 (W≡*C*), 149.50 (*Ar*), 144.31 (*Ar*), 141.77 (*Ar*), 137.59 (*Ar*), 137.28 (*Ar*), 136.01 (*Ar*), 135.12 (*Ar*), 130.47 (*Ar*), 130.24 (*Ar*), 130.00 (*Ar*), 129.13 (*Ar*), 128.59 (*Ar*), 128.48 (*Ar*), 128.35 (*Ar*), 126.35 (*Ar*), 125.93 (*Ar*), 20.54 (*p*-*C*H$_3$), 20.13 (*o*-*C*H$_3$).

[$C_6H_3(C_6H_4SiPh_2O)_3$]W≡CMes (Cat3)

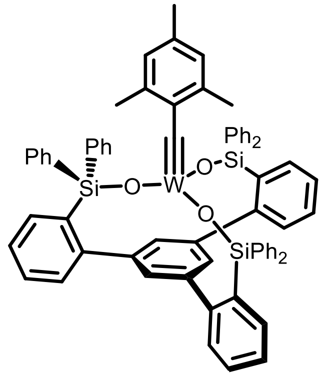

W(≡CMes)Br_3(DME) (1.00 g/1.52 mmol) was dissolved into 10 ml of THF and NaO^{t}Bu (0.437 mg/4.54 mmol) was dissolved in 10 ml of THF and both solutions were chilled to -37 ^{o}C. The colorless solution of NaO^{t}Bu was dropwise added to the stirring, green solution of W(≡CMes)Br_3(DME), resulting in the solution becoming yellow-brown and the precipitation of KBr by-product. The reaction stirred at room-temperature for 0.5 h at which point solvent was removed under reduced pressure, the product extracted into 10 ml of pentane and the KBr impurity removed by filtering through 3" celite plug on a medium porosity fritted funnel. The volatiles were removed under reduced pressure and the resulting off-white solids were redissolved into 10 ml toluene and **SiP^{Ph}** (1.37 g/1.52 mmol) was added directly to the solution, resulting in a color change from yellow to orange. After stirring for 10 min. the volatiles were removed under reduced pressure. Single crystals (orange plates) suitable for X-ray diffraction studies were grown from slow-evaporation of dichloromethane orange, pure powder was obtained by chilling a concentrated toluene solution to -37 ^{o}C. Yield = 1.47 g (79.6%). ^{1}H NMR (500 MHz, C_6D_6, 25 °C): 2.14 (6H, s, *o*-C*H*$_3$), 2.16 (3H, s, *p*-C*H*$_3$), 6.48 (2H, s, C_6*H*$_2$Me$_3$), 6.90 (3H, d, $^3J_{HH}$ = 10.0 Hz, Ar-*H*), 7.03-7.10 (24H, m, Overlap of several Ar-*H*), 7.28 (3H, s, C_6*H*$_3$Ar$_3$), 7.76 (3H, d, $^3J_{HH}$ = 10.0 Hz, Ar-*H*), 7.83 (12H, d, $^3J_{HH}$ = 10.0 Hz, *o*-SiPh$_2$). ^{13}C{^{1}H} NMR (125.77 MHz, C_6D_6, 25 °C): 288.42 (W≡*C*), 149.50 (*Ar*), 144.31 (*Ar*), 141.77 (*Ar*), 137.59 (*Ar*), 137.28 (*Ar*), 136.01 (*Ar*), 135.12 (*Ar*), 130.47 (*Ar*), 130.24 (*Ar*), 130.00 (*Ar*), 129.13 (*Ar*), 128.59 (*Ar*), 128.48 (*Ar*), 128.35 (*Ar*), 126.35 (*Ar*), 125.93 (*Ar*), 20.54 (*p*-*C*H$_3$), 20.13 (*o*-*C*H$_3$).

[$C_6H_3(C_6H_4SiEt_2O)_3$]Mo≡CMes (Cat4)

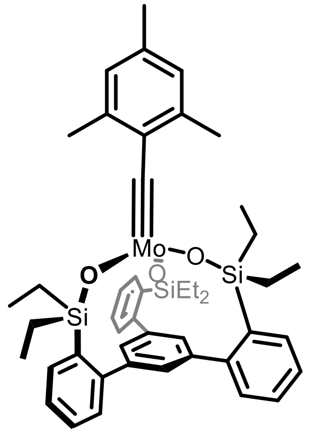

Mo(≡CMes)Br_3(DME) (280 mg/0.53 mmol) was dissolved into 10 ml of THF and NaO^{t}Bu (158 mg/1.59 mmol) was dissolved in 10 ml of THF and both solutions were chilled to -37 ^{o}C. The colorless solution of NaO^{t}Bu was dropwise added to the stirring, brown solution of Mo(≡CMes)Br_3(DME), resulting in the solution becoming yellow-brown and the precipitation of KBr by-product. The reaction stirred at room-temperature for 0.5 h at which point solvent was removed under reduced pressure, the product extracted into 10 ml of pentane and the KBr impurity removed by filtering through 3" celite plug on a medium porosity fritted funnel. The volatiles were removed under reduced pressure and the resulting off-white solids were redissolved into 10 ml pentane and **SiP^{Et}** (308 g/0.53 mmol) was added directly to the solution, resulting in a color change from yellow to orange-yellow. After stirring for 10 min. the volatiles were removed under reduced pressure. Single crystals (yellow plates) suitable for X-ray diffraction studies were grown from a concentrated Et_2O solution stored at -37 °C. Yield = 337 mg (75.5%). ^{1}H NMR (400 MHz, C_6D_6, 25 °C): 0.96 (12H, q, $^3J_{HH}$ = 4.0 Hz, SiC*H$_2$*CH$_3$), 1.01 (18H, t, $^3J_{HH}$ = 4.0 Hz, SiCH$_2$C*H$_3$*), 2.09 (3H, s, *p*-C*H*$_3$), 2.72 (6H, s, *p*-C*H*$_3$), 6.63 (2H, s, C_6*H*$_2$Me$_3$), 7.17-7.19 (12H, m, Ar-*H*), 7.28 (3H, d, $^3J_{HH}$ = 8.0 Hz, Ar-*H*), 7.45 (3H, d, $^3J_{HH}$ = 8.0 Hz, Ar-*H*), 7.50 (3H, s, C_6*H$_3$*). ^{13}C{^{1}H} NMR

(125.77 MHz, C_6D_6, 25 °C): 306.7 (Mo≡*C*), 149.68 (*Ar*), 144.69 (*Ar*), 143.73 (*Ar*), 138.38 (*Ar*), 137.19 (*Ar*), 136.49 (*Ar*), 134.80 (*Ar*), 130.81 (*Ar*), 129.13 (*Ar*), 138.58 (*Ar*), 127.51 (*Ar*), 126.72 (*Ar*), 21.07 (*o*-CH_3), 20.78 (*p*-CH_3), 9.62 (SiCH_2CH_3), 7.31 (SiCH_2CH_3).

[$C_6H_3(C_6H_4SiEt_2O)_3$]W≡CMes (Cat5)

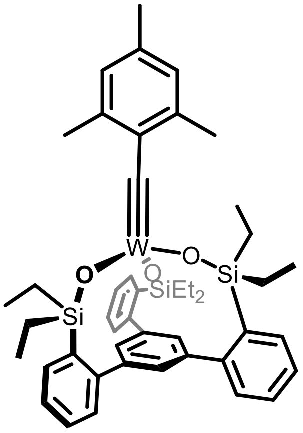

W(≡CMes)Br_3(DME) (250 mg/0.38 mmol) was dissolved into 10 ml of THF and NaO^tBu (125 mg/1.14 mmol) was dissolved in 10 ml of THF and both solutions were chilled to -37 °C. The colorless solution of NaO^tBu was dropwise added to the stirring, green solution of W(≡CMes)Br_3(DME), resulting in the solution becoming yellow-brown and the precipitation of KBr by-product. The reaction stirred at room-temperature for 0.5 h at which point solvent was removed under reduced pressure, the product extracted into 10 ml of pentane and the KBr impurity removed by filtering through 3" celite plug on a medium porosity fritted funnel. The volatiles were removed under reduced pressure and the resulting off-white solids were redissolved into 10 ml pentane and **SiP^{Et}** (238 mg/0.38 mmol) was added directly to the solution. After stirring for 10 min. the volatiles were removed under reduced pressure. Single crystals (yellow plates) suitable for X-ray diffraction studies were grown from a concentrated pentane solution to -37 °C. Yield = 260 mg (73.7%). ^{1}H NMR (400 MHz, C_6D_6, 25 °C): 0.86-0.97 (12H, m, Si$CH_2$$CH_3$), 1.00 (18H, m, Si$CH_2CH_3$), 2.33 (3H, s, *p*-$CH_3$), 2.89 (6H, s, *o*-$CH_3$), 6.88 (2H, s, $C_6H_2Me_3$), 7.18 (3H, m, Ar-*H*), 7.26 (3H, t, $^3J_{HH}$ = 8.4 Hz, Ar-*H*), 7.39 (3H, dd, $^3J_{HH}$ = 8.4 Hz, Ar-*H*), 7.47 (3H, s, C_6H_3). $^{13}C\{^1H\}$ NMR (125.77 MHz, C_6D_6, 25 °C): 281.9 (W≡*C*), 149.5 (*Ar*), 144.7 (*Ar*), 142.6 (*Ar*), 140.6 (*Ar*), 135.9 (*Ar*), 135.8 (*Ar*), 134.7 (*Ar*), 130.8 (*Ar*), 126.8 (*Ar*), 126.7 (*Ar*), 20.9 (*o*-CH_3), 20.7 (*p*-CH_3), 9.3 (SiCH_2CH_3), 7.1 (SiCH_2CH_3).

$(Ph_3SiO)_3Mo(C_3Et_3)$ (MCBD1)

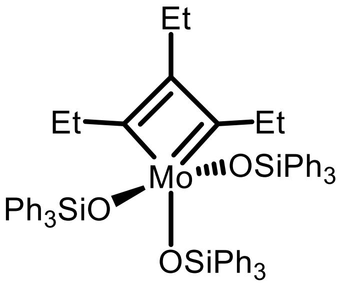

Cat6 (60 mg/0.059 mmol) was dissolved into 0.6 ml of CD_2Cl_2 and 3-hexyne (29 mg/0.351 mmol) was added. The solution was cooled to -70 °C and subjected to NMR interrogation. ^{1}H NMR (500 MHz, CD_2Cl_2, -70 °C): 0.03 (3H, t, $^3J_{HH}$ = 8.0 Hz, β-CCH_2CH_3), 1.49 (6H, t, $^3J_{HH}$ = 8.0 Hz, α-CCH_2CH_3), 2.87 (4H, q, $^3J_{HH}$ = 7.5 Hz, α-CCH_2CH_3), 2.14 (2H, q, $^3J_{HH}$ = 8.0 Hz, β-CCH_2CH_3), 2.87 (4H, q, $^3J_{HH}$ = 8.0 Hz, α-CCH_2CH_3), 7.00 (6H, t, $^3J_{HH}$ = 8.0 Hz, *p*-C_6H_5), 7.10 (12H, t, $^3J_{HH}$ = 8.0 Hz, *p*-C_6H_5) 7.15-7.30 (18H, m, *m*-C_6H_5), 7.38 (12H, d, $^3J_{HH}$ = 8.0 Hz, *o*-C_6H_5), 7.78 (6H, d, $^3J_{HH}$ = 4.0 Hz, *o*-C_6H_5). $^{13}C\{^1H\}$ NMR (125 MHz, CD_2Cl_2, -70 °C): 249.78 (α-*C*CH_2CH_3), 147.15 (β-*C*CH_2CH_3), 147.15 (*Ar*), 137.88 (*Ar*), 137.83 (*Ar*), 137.27 (*Ar*), 135.44 (*Ar*), 134.87 (*Ar*), 134.81 (*Ar*), 134.58 (*Ar*), 131.17 (*Ar*), 131.11 (*Ar*), 130.07 (*Ar*), 129.27 (*Ar*), 129.21 (*Ar*), 129.03 (*Ar*), 128.95 (*Ar*), 128.78 (*Ar*), 128.16 (*Ar*), 127.83 (*Ar*), 127.71 (*Ar*), 127.41 (*Ar*), 125.21 (*Ar*), 120.25 (*Ar*), 31.56 (α-CCH_2CH_3), 26.06 (β-CCH_2CH_3), 13.84 (α-CCH_2CH_3), 11.22 (β-CCH_2CH_3).

[$C_6H_3(C_6H_4SiPh_2O)_3$]Mo≡CBu (Cat7)

Cat1 (375 mg/0.320 mmol) was dissolved into 10 ml of toluene and 5-decyne (131 mg/0.960 mmol) leading to a dark brown solution. The solution was allowed to stir for 0.5 h at room-temperature. All volatiles were removed under reduced pressure and the crude product was washed

with pentane to give a grey-brown solid. The crude solid was redissolved in toluene and stirred over 5 Å molecular sieves (500 mg) for 2 h. The sieves were removed via filtration through a fine porosity fritted funnel and the volatiles of the supernatant were removed under reduced pressure while heating to 50 ˚C to give a yellow solid. Yield = 78.6 mg (23.1%). ^{1}H NMR (500 MHz, C_6D_6, 25 °C): 0.39 (3H, t, $^3J_{HH}$ = 6.0 Hz, $Mo\equiv CCH_2CH_2CH_2C\mathit{H}_3$), 0.39 (3H, t, $^3J_{HH}$ = 6.0 Hz, $Mo\equiv CCH_2CH_2CH_2C\mathit{H}_3$), 0.72 (2H, m, $^3J_{HH}$ = 4.0 Hz, $Mo\equiv CCH_2CH_2C\mathit{H}_2CH_3$, 0.91 (2H, m, $^3J_{HH}$ = 4.0 Hz, $Mo\equiv CCH_2C\mathit{H}_2CH_2CH_3$), 2.56 (2H, t, $^3J_{HH}$ = 8.0 Hz, $Mo\equiv CC\mathit{H}_2CH_2CH_2CH_3$), 6.87 (3H, d, $^3J_{HH}$ = 8.0, Ar-*H*), 7.05-7.14 (24H, m, Overlap of several Ar-*H*), 7.24 (3H, s, $C_6\mathit{H}_3Ar_3$), 7.76 (3H, d, $^3J_{HH}$ = 8.0 Hz, Ar-*H*), 7.87 (12H, d, $^3J_{HH}$ = 8.0 Hz, *o*-$SiPh_2$). $^{13}C\{^1H\}$ NMR (125.77 MHz, C_6D_6, 25 °C): 324.87 ($Mo\equiv \mathit{C}$), 149.53 (*Ar*), 144.13 (*Ar*), 138.35 (*Ar*), 135.10 (*Ar*), 130.44 (*Ar*), 130.08 (*Ar*), 129.81 (*Ar*), 129.18 (*Ar*), 126.24 (*Ar*), 50.95 ($Mo\equiv C\mathit{C}H_2CH_2CH_2CH_3$), 31.27 ($Mo\equiv CCH_2\mathit{C}H_2CH_2CH_3$), 22.36 ($Mo\equiv CCH_2CH_2\mathit{C}H_2CH_3$), 13.59 ($Mo\equiv CCH_2CH_2CH_2\mathit{C}H_3$).

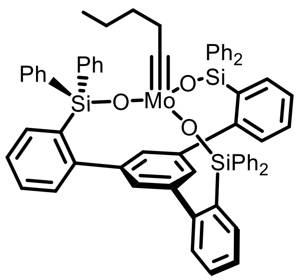

[$C_6H_3(C_6H_4SiPh_2O)_3$]Mo(C_3Bu_3) (MT$_d$2)

Cat7 (78 mg/0.073 mmol) was dissolved into 1.2 ml of CH_2Cl_2 and 5-decyne (101 mg/0.733 mmol) leading to a dark brown solution. The solution was stored at -37 °C for 6 months to give crystalline material of sufficient quality to acquire X-ray diffraction data. Not all shifts of **MT$_d$2** in the ^{1}H and ^{13}C NMR couple be assigned due to signal broadening and overlaps with 5-decyne and **Cat7**. ^{1}H NMR (500 MHz, C_6D_6, 25 °C): 0.62 (9H, t, $^3J_{HH}$ = 7.5 Hz, $Mo(CCH_2CH_2CH_2C\mathit{H}_3)_3$, 0.99 (6H, t, $^3J_{HH}$ = 7.5 Hz, $Mo(CC\mathit{H}_2CH_2CH_2CH_3)_3$, 1.18-.1.29 (12H, m, $^3J_{HH}$ = 7.5 Hz, $Mo(CCH_2C\mathit{H}_2C\mathit{H}_2CH_3)_3$. $^{13}C\{^1H\}$ NMR (125.77 MHz, C_6D_6, 25 °C): 150.6 (*Ar*), 144.5 (*Ar*), 137.9 (*Ar*), 137.7 (*Ar*), 137.3 (*Ar*), 135.6 (*Ar*), 129.6 (*Ar*), 129.3 (*Ar*), 128.8 (*Ar*), 128.6 (*Ar*), 125.7 (*Ar*), 125.7 (*Ar*), 83.4 (C_3Bu_3), 33.4 ($\mathit{C}H_2CH_2CH_2CH_3$), 26.1 ($CH_2\mathit{C}H_2CH_2CH_3$), 22.8 ($CH_2CH_2\mathit{C}H_2CH_3$), 14.2 ($CCH_2CH_2CH_2\mathit{C}H_3$).

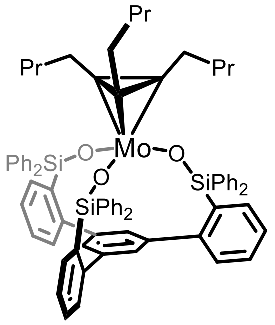

[$C_6H_3(C_6H_4SiPh_2O)_3$]W(C_3MesEt_2) (MCBD2)

Cat3 (73 mg/0.06 mmol) was suspended into 5 ml of pentane and to this was added 3-hexyne (10 mg/0.120 mmol), without stirring, resulting in a purple solution. Storage of this still solution at room temperature for 2 hr led to the deposition of crystalline material. Storage at -37 ˚C led to additional crystalline material. Yield = 32 mg (41%). ^{1}H NMR (400 MHz, C_6D_6, 25 °C): 0.33 (3H, br s, $CH_2C\mathit{H}_3$), 0.70 (3H, br s, $CH_2C\mathit{H}_3$), 1.52 (6H, br s, *o*-$C\mathit{H}_3$), 2.07 (3H, s, *p*-$C\mathit{H}_3$), 2.63 (2H, br s, $C\mathit{H}_2CH_3$), 3.09 (2H, br s, $C\mathit{H}_2CH_3$), 6.60 (2H, s, $C_6H_2Me_3$), 6.93-7.12 (22 H, m, Ar-*H*), 7.30-7.90 (20H, m, Ar-*H*), 8.01 (2H, br s, Ar-*H*), 8.39 (1H, br s, Ar-*H*).

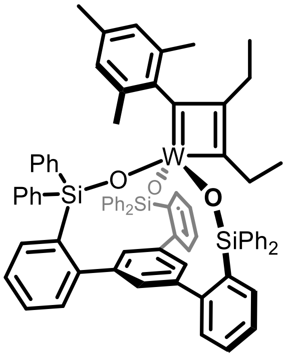

[$C_6H_3(C_6H_4SiEt_2O)_3$]W(C_3MesEt$_2$) (MCBD3)

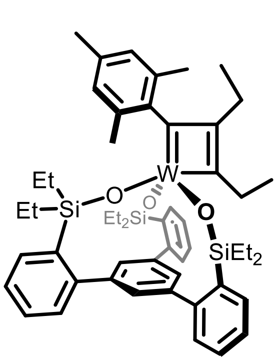

Cat5 (160 mg/0.151 mmol) was dissolved into 3 ml of pentane and to this solution was added 3-hexyne (13 mg/0.157 mmol) at room temperature, leading to a purple solution. was allowed to stir for 1.5 h while warming to room-temperature. Storage of the solution overnight at -37 ˚C lead to the deposition of purple crystalline material. Yield = 131 mg (86.1%). ^{1}H NMR (400 MHz, C_6D_6, 25 °C): 0.70 (15 H, br s, Si*CH_2*CH$_3$ & CH$_2$C*H$_3$*), 0.85-1.05 (21H, br s, SiCH$_2$C*H$_3$* & CH$_2$C*H$_3$*), 2.18 (3H, s, *p*-C*H$_3$*), 2.29 (6H, br s, *o*-C*H$_3$*), 2.93 (2H, br s, C*H$_2$*CH$_3$), 3.27 (2H, br s, C*H$_2$*CH$_3$), 6.91 (2H, s, $C_6H_2Me_3$), 7.21 (3 H, br s, Ar-*H*), 7.32 (3H, br s, Ar-*H*), 7.43 (3H, br s, Ar-*H*), 7.64 (3H, br s, Ar-*H*). Decompositon of **MCBD3** to **MCBD5** and 1-mesityl-1-butyne frustrated our attempts at ^{13}C collection.

[$C_6H_3(C_6H_4SiPh_2O)_3$]W(C_3Et_3) (MCBD4)

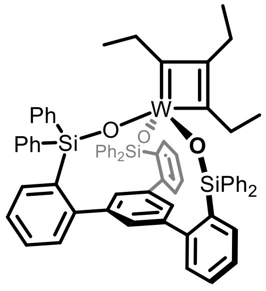

Cat3 (70 mg/0.058 mmol) was dissolved into 0.6 ml of C_6D_6 and 3-hexyne (12 mg/0.148 mmol) was added leading to a purple solution. The reaction was heated to 60 ˚C for 15 minutes leading to a reddish-maroon solution. All volatiles were removed under reduced pressure and the resulting oily-solid was dissolved into dichloromethane. Crystalline material was produced via slow-evaporation at -37° C overnight. Yield = 57.5 mg (82.3%). ^{1}H NMR (500 MHz, CD_2Cl_2, -70 °C): -0.28 (3H, t, $^3J_{HH}$ = 6.0 Hz, α'-CCH$_2$C*H$_3$*), 0.96 (3H, t, $^3J_{HH}$ = 6.0 Hz, α-CCH$_2$C*H$_3$*), 1.22 (3H, t, $^3J_{HH}$ = 6.0 Hz, β-CCH$_2$C*H$_3$*), 1.82 (2H, q, $^3J_{HH}$ = 6.0 Hz, α'-CC*H$_2$*CH$_3$), 3.29 (2H, q, $^3J_{HH}$ = 6.0 Hz, β-CC*H$_2$*CH$_3$), 4.16 (2H, q, $^3J_{HH}$ = 6.0 Hz, α-CC*H$_2$*CH$_3$), 6.82-7.86 (45H, m, *Ar*). ^{13}C{^{1}H} NMR (125 MHz, CD_2Cl_2, -70 °C): 234.78 (α'-*C*CH$_2$CH$_3$), 229.89 (α-*C*CH$_2$CH$_3$), 148.72 (*Ar*), 148.28 (*Ar*), 147.65 (*Ar*), 145.96 (*Ar*), 143.23 (*Ar*), 142.82 (*Ar*), 140.33 (*Ar*), 139.69 (*Ar*), 139.40 (*Ar*), 139.23 (*Ar*), 138.93 (*Ar*), 138.79 (β-*C*CH$_2$CH$_3$), 138.00 (*Ar*), 137.25 (*Ar*), 136.94 (*Ar*), 136.35 (*Ar*), 135.37 (*Ar*), 135.20 (*Ar*), 134.51 (*Ar*), 134.31 (*Ar*), 134.12 (*Ar*), 133.64 (*Ar*), 130.73 (*Ar*), 130.31 (*Ar*), 129.84 (*Ar*), 129.57 (*Ar*), 129.22 (*Ar*), 128.96 (*Ar*), 128.84 (*Ar*), 128.40 (*Ar*), 127.73 (*Ar*), 127.73 (*Ar*), 127.49 (*Ar*), 127.27 (*Ar*), 127.21 (*Ar*), 127.05 (*Ar*), 126.19 (*Ar*), 125.91 (*Ar*), 125.22 (*Ar*), 125.13 (*Ar*), 1215.57 (*Ar*), 30.07 (α-C*C*H$_2$CH$_3$), 28.17 (α'-C*C*H$_2$CH$_3$), 26.03 (β-C*C*H$_2$CH$_3$), 16.07 (α-CCH$_2$*C*H$_3$), 14.20 (α'-CCH$_2$*C*H$_3$), 13.89 (β-CCH$_2$*C*H$_3$).

[$C_6H_3(C_6H_4SiEt_2O)_3$]W(C_3Et_3) (MCBD5)

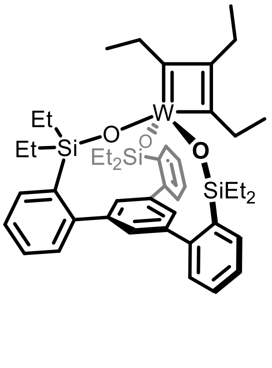

Cat5 (44 mg/0.036 mmol) was dissolved into 0.6 ml of C_6D_6 and 3-hexyne (15 mg/0.181 mmol) was added leading to a purple solution. The reaction proceeded overnight at which point all volatiles were removed under reduced pressure. The resulting purple solid was dissolved into minimal pentane and stored at -37° C overnight. Solvent was removed and the purple crystalline material was dried under reduced pressure. Yield = 18 mg (53%). ^{1}H NMR (500 MHz, C_6D_6, 25 °C): 0.12 (2H, q, $^3J_{HH}$ = 10.0 Hz, SiC*H$_2$*CH$_3$), 0.40 (3H, t, $^3J_{HH}$ = 7.6 Hz, α'-CCH$_2$C*H$_3$*),

0.60 (6H, t, $^3J_{HH}$ = 4.4 Hz, SiCH$_2$C*H*$_3$), 0.74 (3H, s, $^3J_{HH}$ = 7.6 Hz, β-CCH$_2$C*H*$_3$), 0.86-0.91 (10H, m, SiC*H*$_2$CH$_3$ and SiCH$_2$C*H*$_3$), 1.15 (2H, m, $^3J_{HH}$ = 7.5 Hz, SiC*H*$_2$CH$_3$), 1.25 (4H, m, $^3J_{HH}$ = 10 Hz, SiC*H*$_2$CH$_3$), 1.39 (6H, t, $^3J_{HH}$ = 7.5 Hz, SiCH$_2$C*H*$_3$), 1.44 (3H, t, $^3J_{HH}$ = 7.5 Hz, β-CCH$_2$C*H*$_3$), 2.96 (2H, q, $^3J_{HH}$ = 7.5 Hz, β-CC*H*$_2$CH$_3$), 3.07 (2H, q, $^3J_{HH}$ = 7.5 Hz, α'-CC*H*$_2$CH$_3$), 3.89 (2H, q, $^3J_{HH}$ = 7.5 Hz, α-CC*H*$_2$CH$_3$), 7.17-7.23 (4H, m, Ar-*H*), 7.29-7.33 (3H, m, Ar-*H*), 7.37 (2H, d, $^3J_{HH}$ = 5.0 Hz, Ar-*H*), 7.53 (1H , d, $^3J_{HH}$ = 5.0 Hz, Ar-*H*), 7.61 (2H, d, $^3J_{HH}$ = 10 Hz, Ar-*H*), 7.64 (2H, d, $^4J_{HH}$ = 5.0 Hz, C$_6$*H*$_3$), 7.96 (1H, t, $^4J_{HH}$ = 5.0 Hz, C$_6$*H*$_3$). ^{13}C{^{1}H} NMR (125.77 MHz, C$_6$D$_6$, 25 °C): 229.38 (α'-*C*CH$_2$CH$_3$), 221.95 (α-*C*CH$_2$CH$_3$), 149.43 (*Ar*), 149.23 (*Ar*), 146.00 (*Ar*), 143.23 (*Ar*), 140.11 (*Ar*), 138.21 (*Ar*), 135.15 (*Ar*), 133.91 (*Ar*), 131.73 (β-*C*CH$_2$CH$_3$), 131.02, 129.33 (*Ar*), 128.99 (*Ar*), 126.61 (*Ar*), 126.28 (*Ar*), 30.17 (α-C*C*H$_2$CH$_3$), 29.92 (α'-C*C*H$_2$CH$_3$), 24.81 (β-C*C*H$_2$CH$_3$), 17.23 (α-CCH$_2$*C*H$_3$), 15.09 (α'-CCH$_2$*C*H$_3$), 14.23 (β-CCH$_2$*C*H$_3$), 13.37 (Si*C*H$_2$CH$_3$), 10.39(Si*C*H$_2$CH$_3$), 10.25 (Si*C*H$_2$CH$_3$), 8.55 (Si*C*H$_2$CH$_3$), 7.98 (SiCH$_2$*C*H$_3$), 7.70 (SiCH$_2$*C*H$_3$), 7.07 (Si*C*H$_2$CH$_3$).

[C$_6$H$_3$(C$_6$H$_4$SiPh$_2$O)$_3$]W(C$_3$MesTolCH$_3$) (MCBD6)

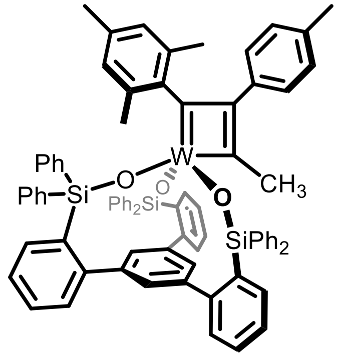

Cat3 (45 mg/0.037 mmol) was added to 10 ml of pentane to give a yellow suspension. *p*-tolylpropyne (24 mg/0.185 mmol) was added at room temperature resulting in the solution darkening to purple-brown. Dark purple, square crystals began forming after 5 minutes and continued to form for 2 hr at which point the solvent was decanted away and the solids dried under reduced pressure. Yield = 24.2 mg (48.6%). ^{1}H NMR (500 MHz, C$_6$D$_6$, 25 °C): 1.46 (3H, br s, C*H*$_3$), 1.87 (3H, br s, C*H*$_3$), 2.12 (6H, br s, *o*-C*H*$_3$), 2.23 (3H, s, *p*-C*H*$_3$), 6.43 (1H, br s, Ar-*H*), 6.68 (2H, br s, Ar-*H*), 6.92-7.09 (24 H, m, Ar-*H*), 7.31 (4 H, s, Ar-*H*), 7.65 (4 H, m, Ar-*H*), 7.98 (1 H, d, Ar-*H*), 8.08 (2 H, s, Ar-*H*). ^{13}C{^{1}H} NMR (125.77 MHz, C$_6$D$_6$, 25 °C): 231.50 (α-*C*$_3$), 228. 02 (α-*C*$_3$) 150.07 (*Ar*), 144.08 (β-*C*$_3$), 141.87 (*Ar*), 139.79 (*Ar*), 139.58 (*Ar*), 139.31 (*Ar*), 137.89 (*Ar*), 137.78 (*Ar*), 137.68 (*Ar*), 137.56 (*Ar*), 137.49 (*Ar*), 137.01 (*Ar*), 136.68 (*Ar*), 136.40 (*Ar*), 136.07 (*Ar*), 135.98 (*Ar*), 135.63 (*Ar*), 135.02 (*Ar*), 131.86 (*Ar*), 131.43 (*Ar*), 126.46 (*Ar*), 125.71 (*Ar*), 122.00 (*Ar*), 21.45 (*p*-*C*H$_3$), 20.96 (*o*-*C*H$_3$), 20.81 (*p*-*C*H$_3$), 20.27 (C$_2$*C*H$_3$).

[C$_6$H$_3$(C$_6$H$_4$SiEt$_2$O)$_3$]W≡CMes(PhCN) (Cat5●PhCN)

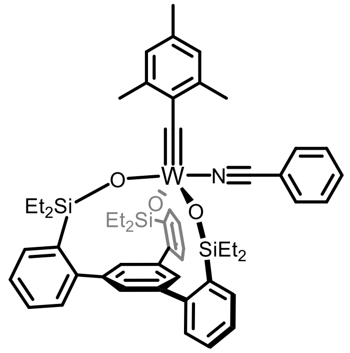

Cat5 (60 mg/0.065 mmol) was dissolved into 5 ml of pentane to give a yellow solution. Benzonitrile (7.4 µl/0.071 mmol) was added at room temperature without stirring. The resulting purple-red solution was left undisturbed for 1 h leading to the deposition of dark purple-red, crystalline material. The solvent was decanted and the solids dried under reduced pressure. Yield = 52 mg (78%). ^{1}H NMR (500 MHz, C$_6$D$_6$, 25 °C): 0.99 (12H, m, SiC*H*$_2$CH$_3$), 1.08 (18H, t, $^3J_{HH}$ = 7.5 Hz, SiCH$_2$C*H*$_3$), 2.33 (3H, s, *p*-C*H*$_3$), 2.96 (6H, s, *o*-C*H*$_3$), 6.55 (2H, d, $^3J_{HH}$ = 8.0 Hz, *m*-Ph-*H*), 6.69 (2H, t, $^3J_{HH}$ = 7.5 Hz, *o*-Ph-*H*), 6.77 (1H, t, $^3J_{HH}$ = 7.5 Hz, *p*-Ph-*H*), 6.89 (2 H, s, *o*-Mes-*H*), 7.29 (3H, m, $^3J_{HH}$ = 4.5 Hz, Ar-*H*), 7.45 (3H, m, $^3J_{HH}$ = 4.5 Hz, Ar-*H*), 7.50 (3H, s, C$_6$*H*$_3$). ^{13}C{^{1}H} NMR (125.77 MHz, C$_6$D$_6$, 25 °C): 288.40 (W≡*C*), 149.63 (*Ar*), 144.23 (*Ar*), 142.11 (*Ar*), 141.45 (*Ar*), 137.36 (*Ar*), 135.72 (*Ar*), 134.92 (*Ar*), 133.47 (*Ar*), 132.07 (*Ar*),

130.69 (*Ar*), 128.98 (*Ar*), 128.83 (*Ar*), 128.49 (*Ar*), 128.44 (*Ar*), 126.62 (*Ar*), 126.58 (*Ar*), 110.58 (*C*≡N), 20.95 (*p*-*C*H_3), 20.72 (*o*-*C*H_3), 9.54 (Si*C*H_2CH_3), 7.57 ($SiCH_2$*C*H_3).

{[$C_6H_3(C_6H_4SiEt_2O)_3$]W≡N}$_2$ (Nitride1)

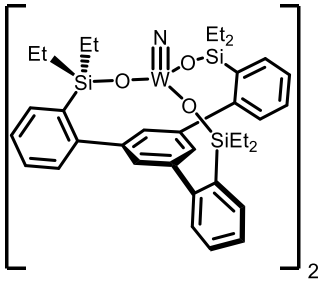

Cat5 (40 mg/0.043 mmol) was dissolved in 0.6 ml of C_6D_6 to give a yellow suspension. Benzonitrile (4.7 µl/0.046 mmol) was added at room temperature resulting in the solution darkening to purple-red. After 3 h at room temperature, the solution had become yellow-brown in color and the formation of mesityl-phenylacetylene could be seen forming along with the consumption of **Cat5●PhCN**. After 6 h the solution was bright yellow and all resonances associated with **Cat5●PhCN** had disappeared. Slow evaporation of the solvent led to the deposition of pale-yellow/colorless crystals of **Nitride1** suitable for X-ray diffraction studies. Yield = 17 mg (49%).

Supporting NMR spectra

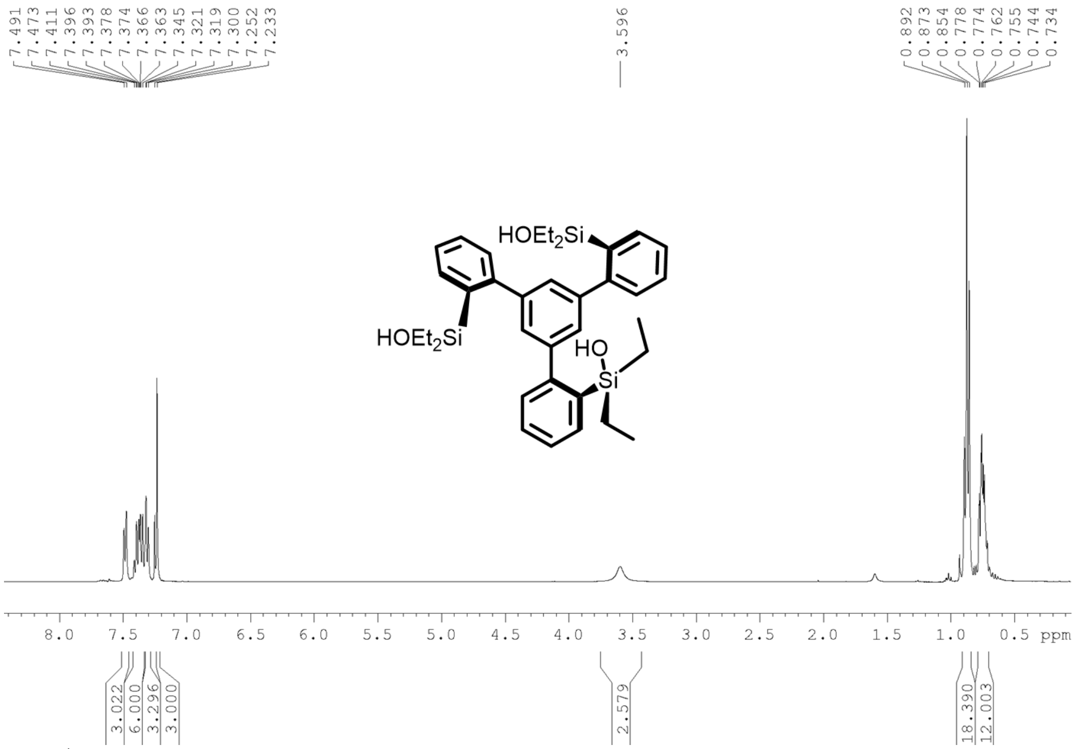

Figure S1. ^{1}H NMR (400 MHz, $CDCl_3$, 25 °C) spectrum of **SiP^{Et}**.

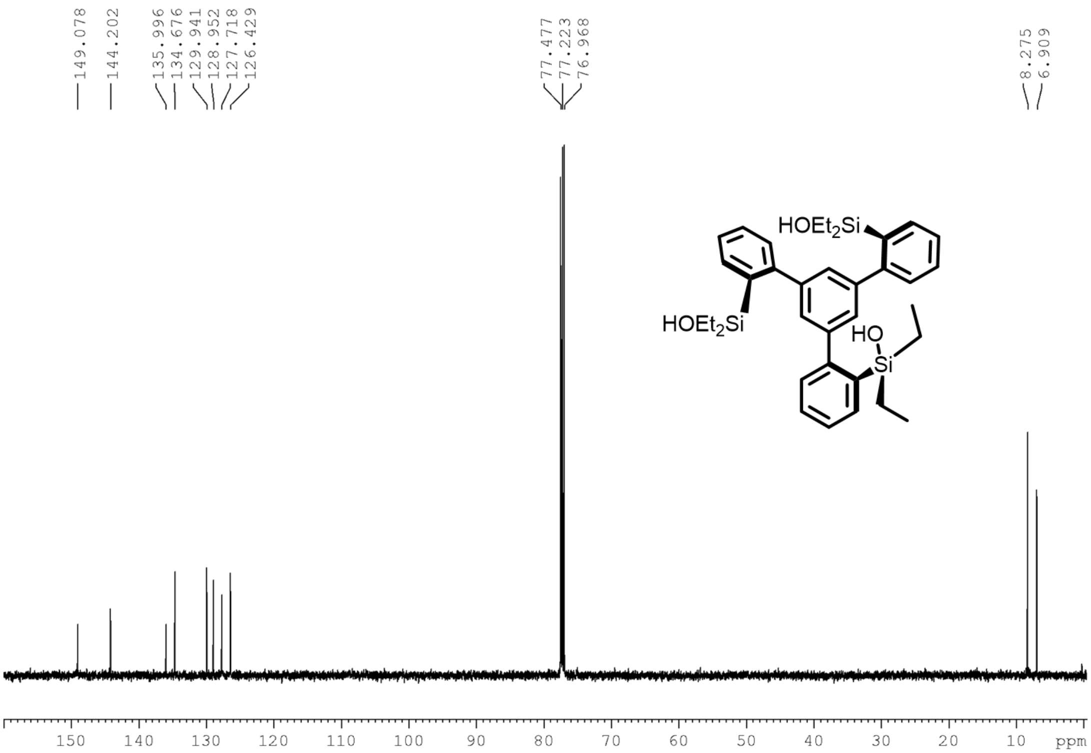

Figure S2. ^{13}C NMR (125 MHz, $CDCl_3$, 25 °C) spectrum of **SiP^{Et}**.

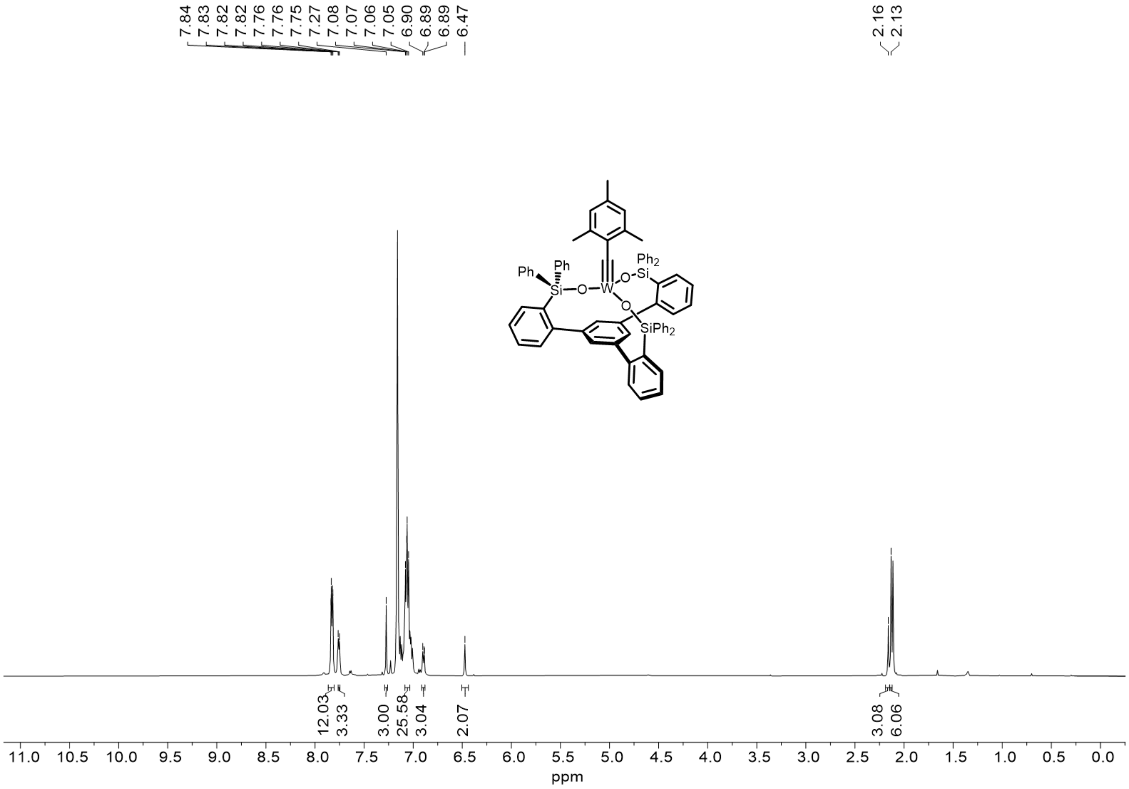

Figure S3. ^{1}H NMR (500 MHz, C_6D_6, 25 °C) spectrum of **Cat3**.

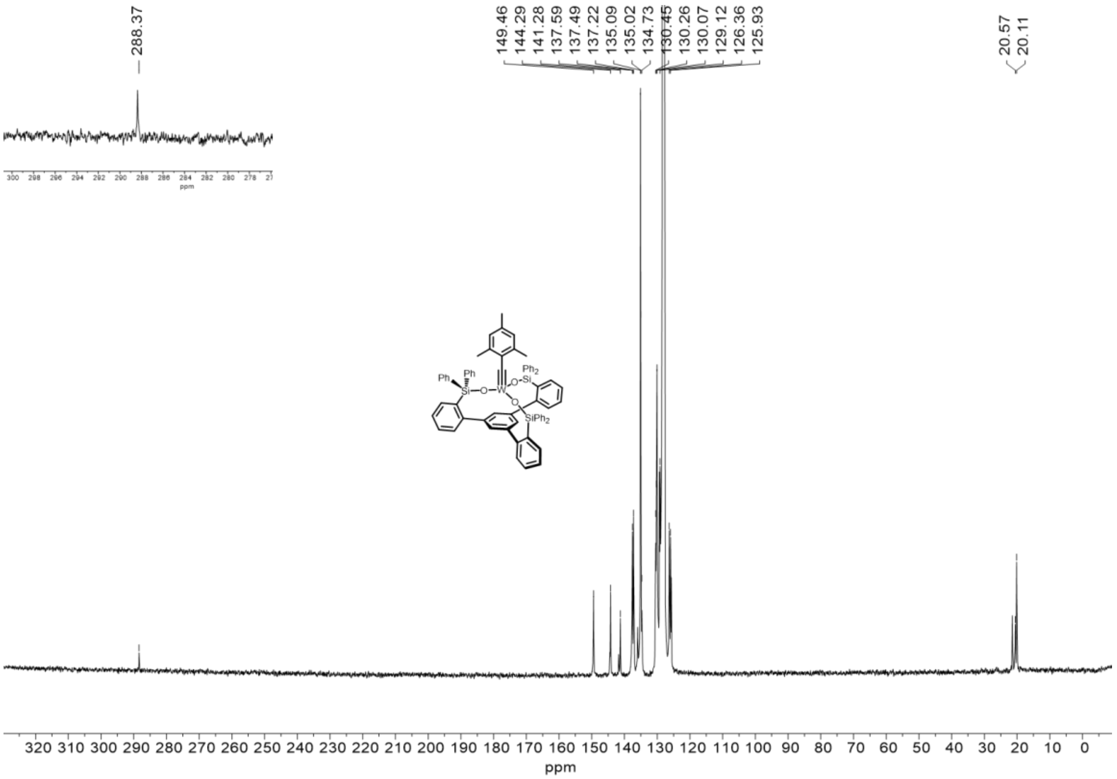

Figure S4. ^{13}C NMR (125 MHz, C_6D_6, 25 °C) spectrum of **Cat3**. The carbyne carbon is expanded in the inset.

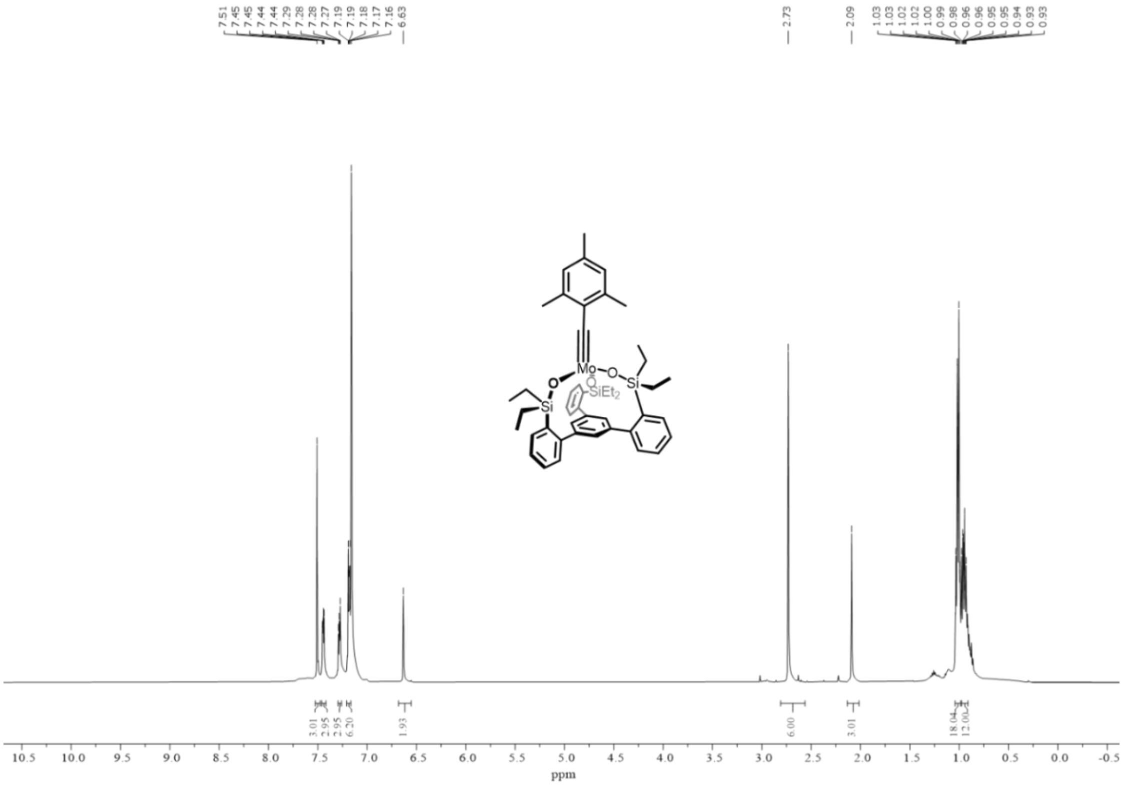

Figure S5. ^{1}H NMR (400 MHz, C_6D_6, 25 °C) spectrum of **Cat4**.

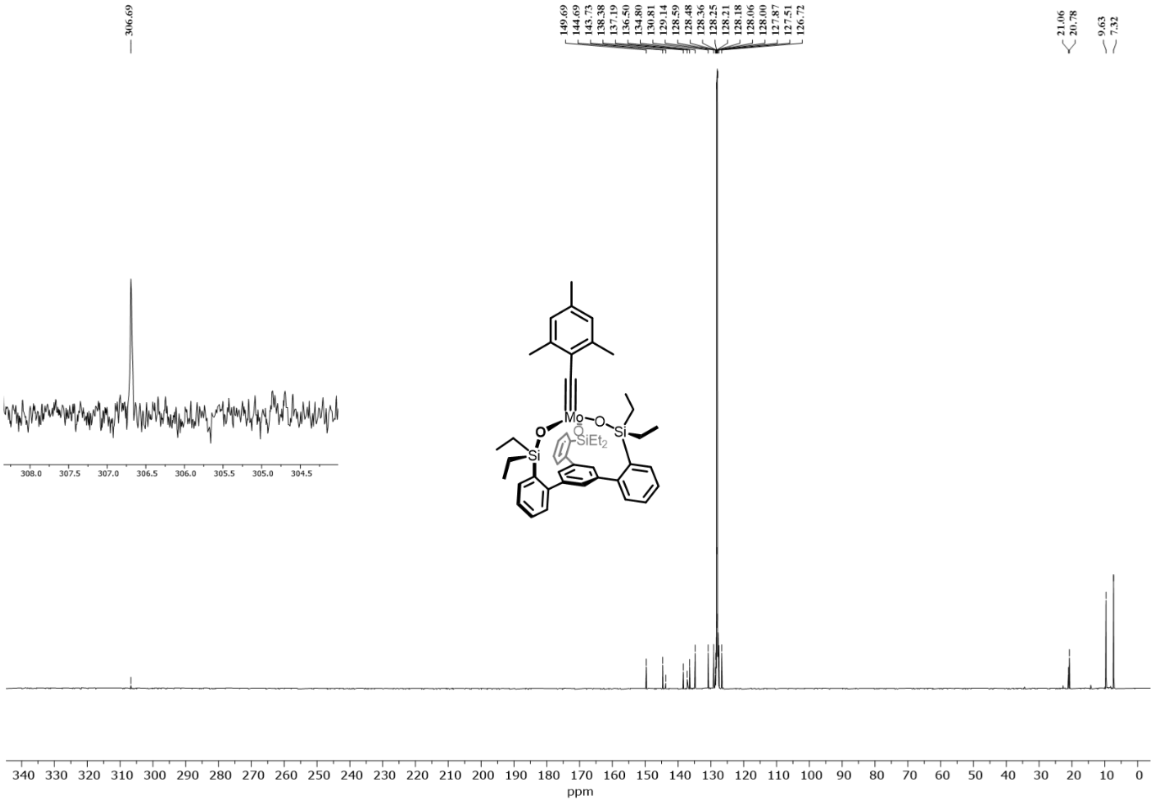

Figure S6. ^{13}C NMR (125 MHz, C_6D_6, 25 °C) spectrum of **Cat4**.

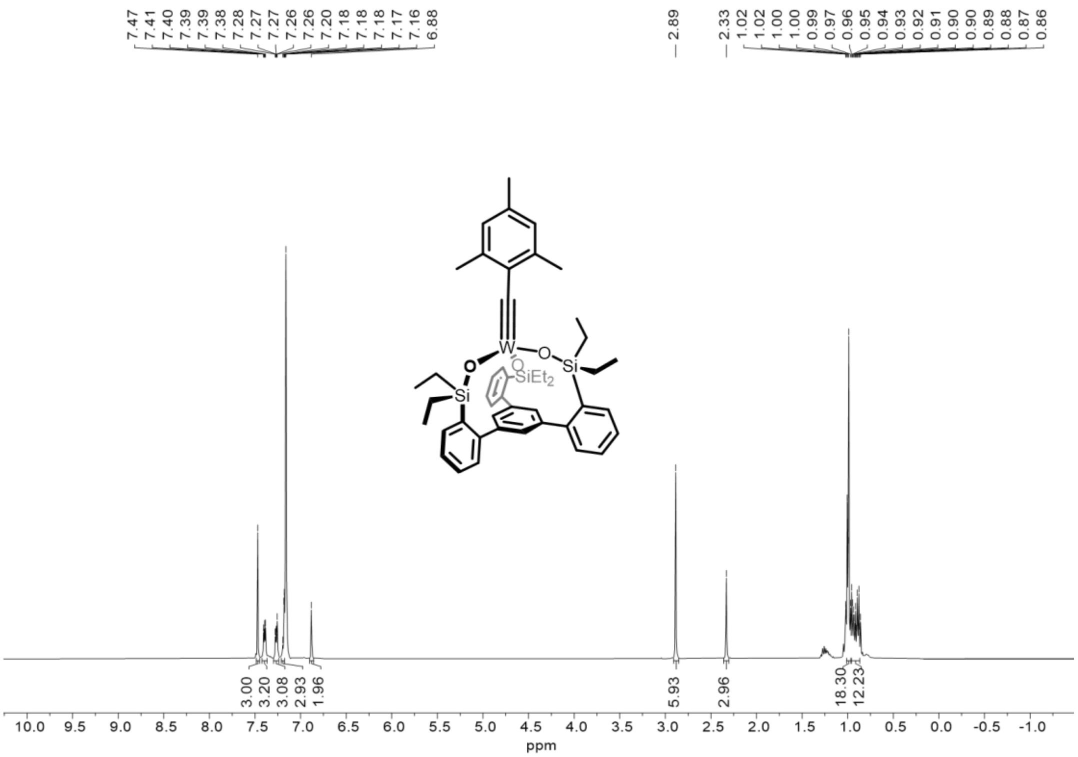

Figure S7. ^{1}H NMR (500 MHz, C_6D_6, 25 °C) spectrum of **Cat5**.

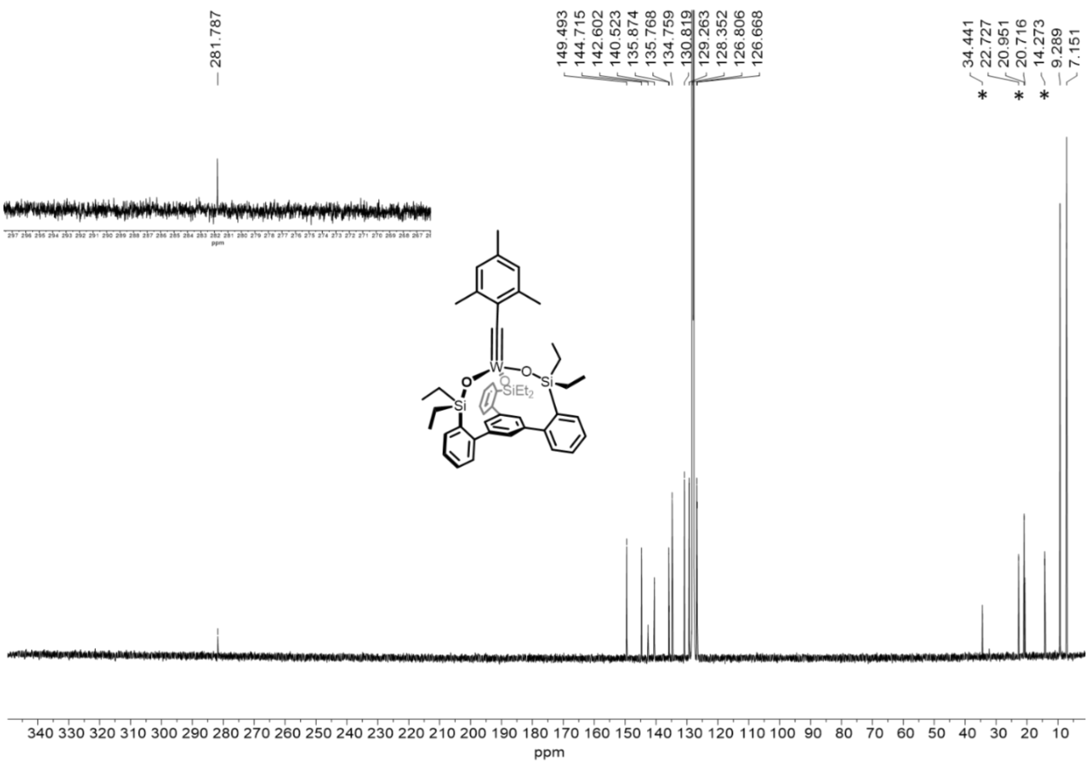

Figure S8. ^{13}C NMR (125 MHz, C_6D_6, 25 °C) spectrum of **Cat5**. The carbyne carbon is expanded in the inset. Pentane impurity is denoted by (*).

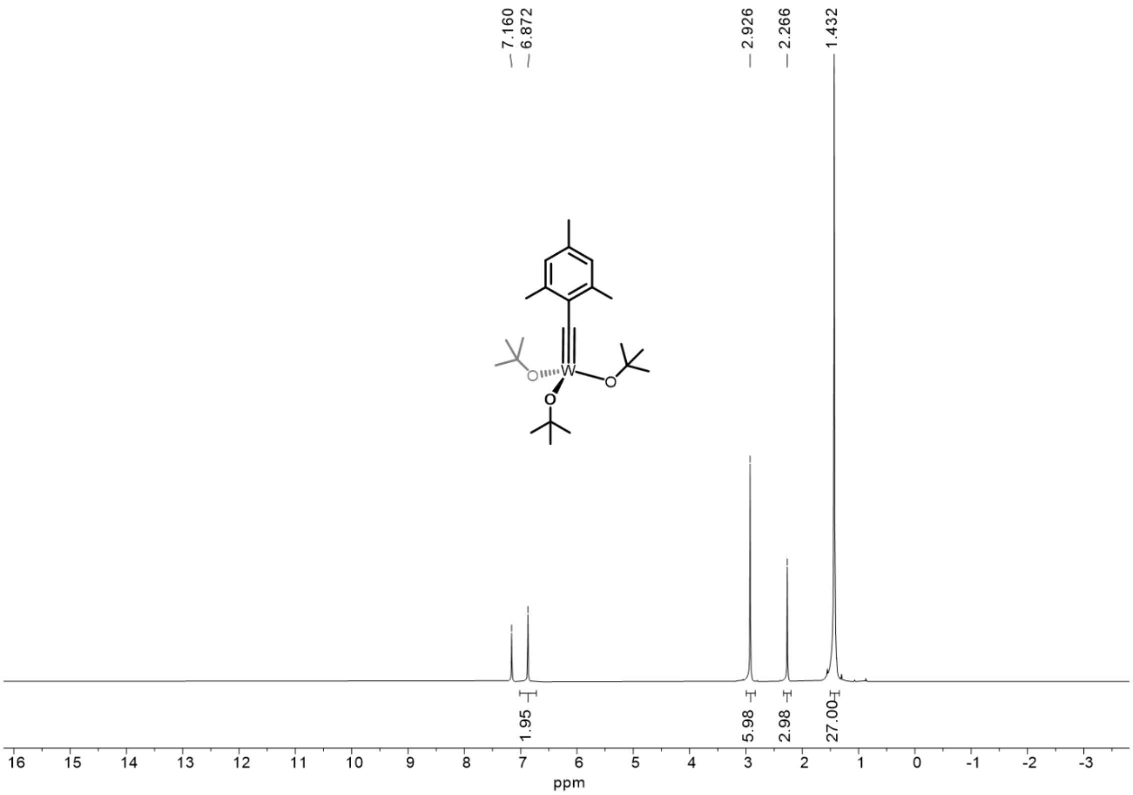

Figure S9. ^{1}H NMR (500 MHz, C_6D_6, 25 °C) spectrum of **Pre2**.

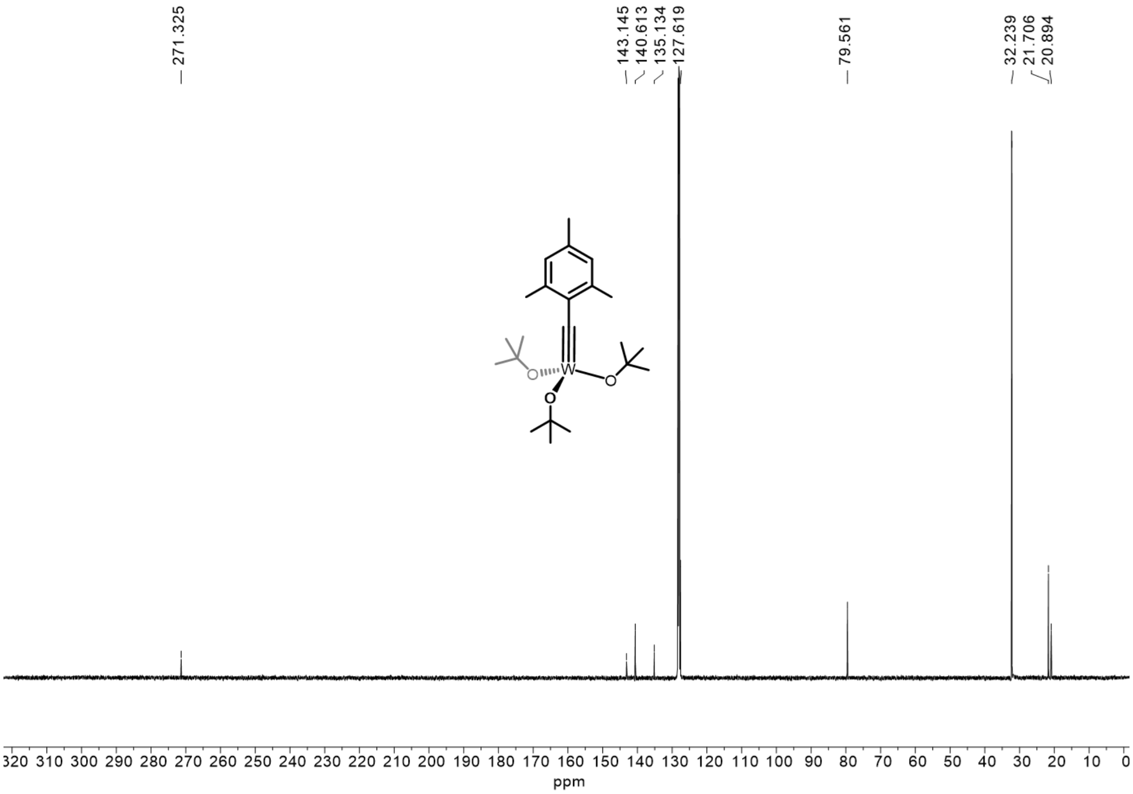

Figure S10. ^{13}C NMR (125 MHz, C_6D_6, 25 °C) spectrum of **Pre2**.

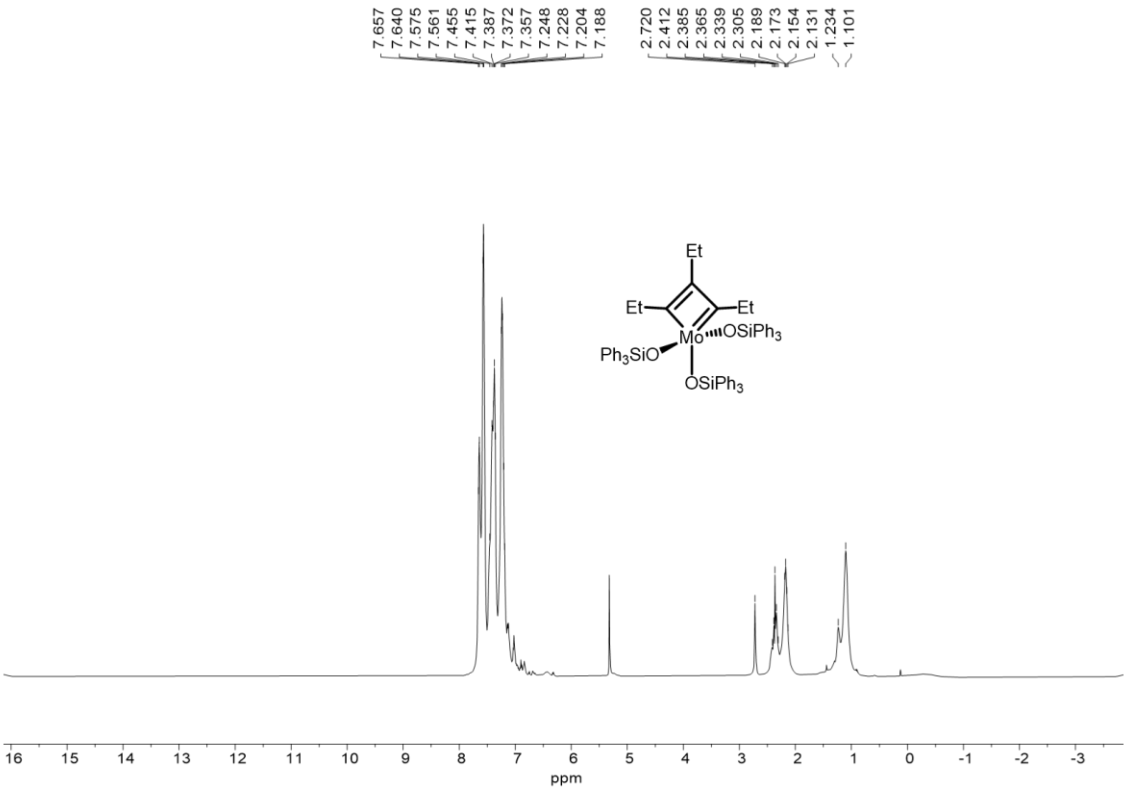

Figure S11. ^{1}H NMR (500 MHz, CD_2Cl_2, 25 °C) spectrum of **MCBD1**.

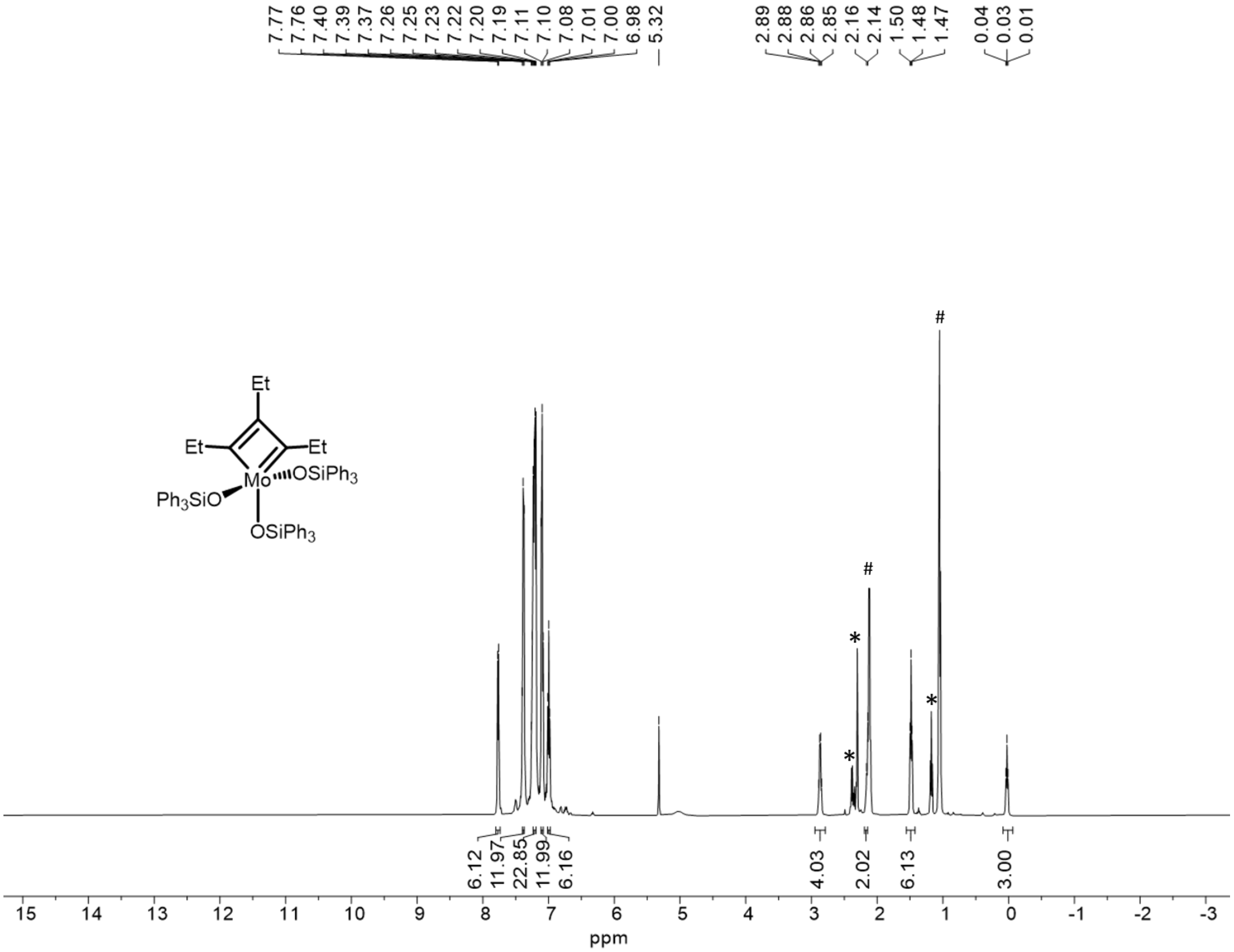

Figure S12. ^{1}H NMR (500 MHz, CD_2Cl_2, -70 °C) spectrum of **MCBD1**. * Denotes 4-tolyl-1-butyne byproduct and # denotes 3-hexyne.

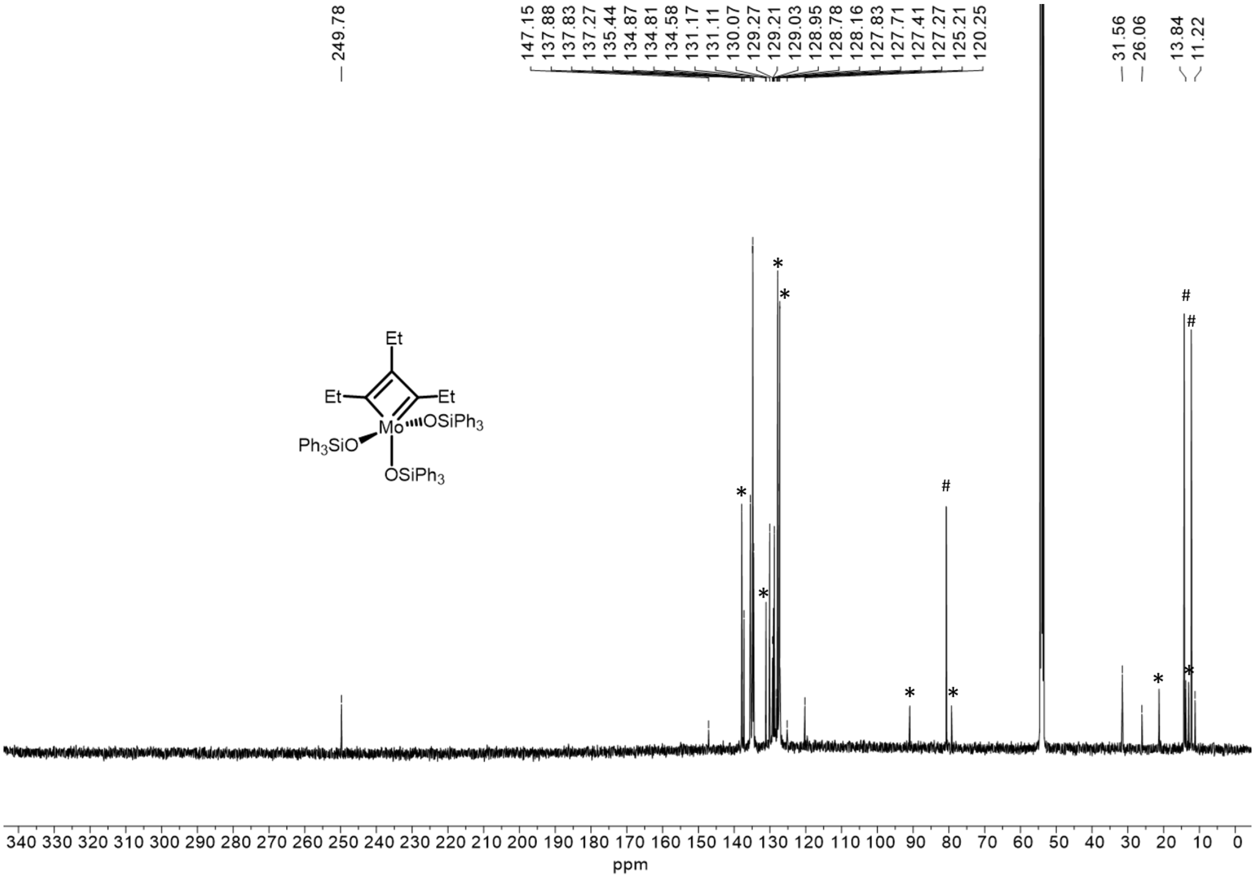

Figure S13. ^{13}C NMR (125 MHz, CD_2Cl_2, -70 °C) spectrum of **MCBD1**. * Denotes 4-tolyl-1-butyne byproduct and # denotes 3-hexyne.

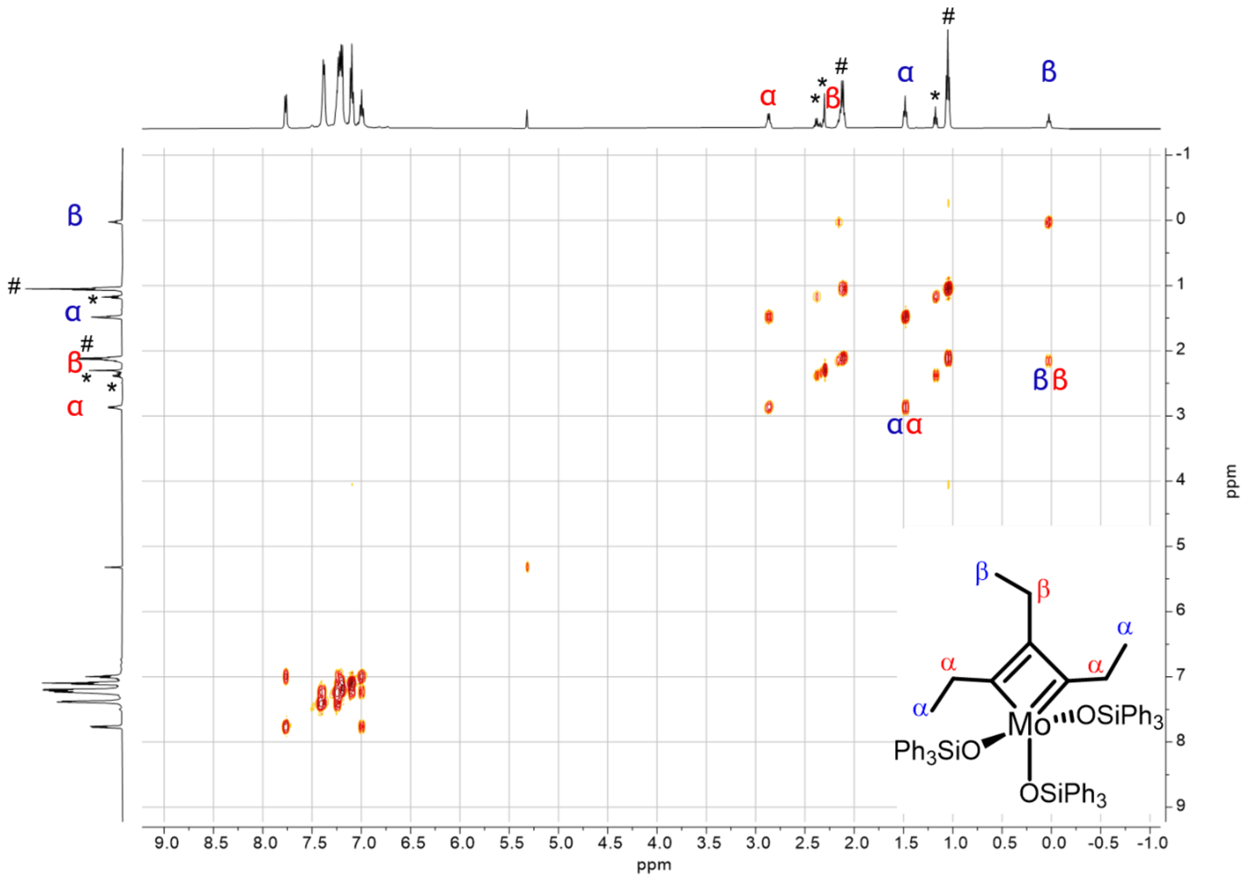

Figure S14. COSY NMR (500 MHz, CD_2Cl_2, -70 °C) spectrum of **MCBD1**. * Denotes 4-tolyl-1-butyne and # denotes 3-hexyne.

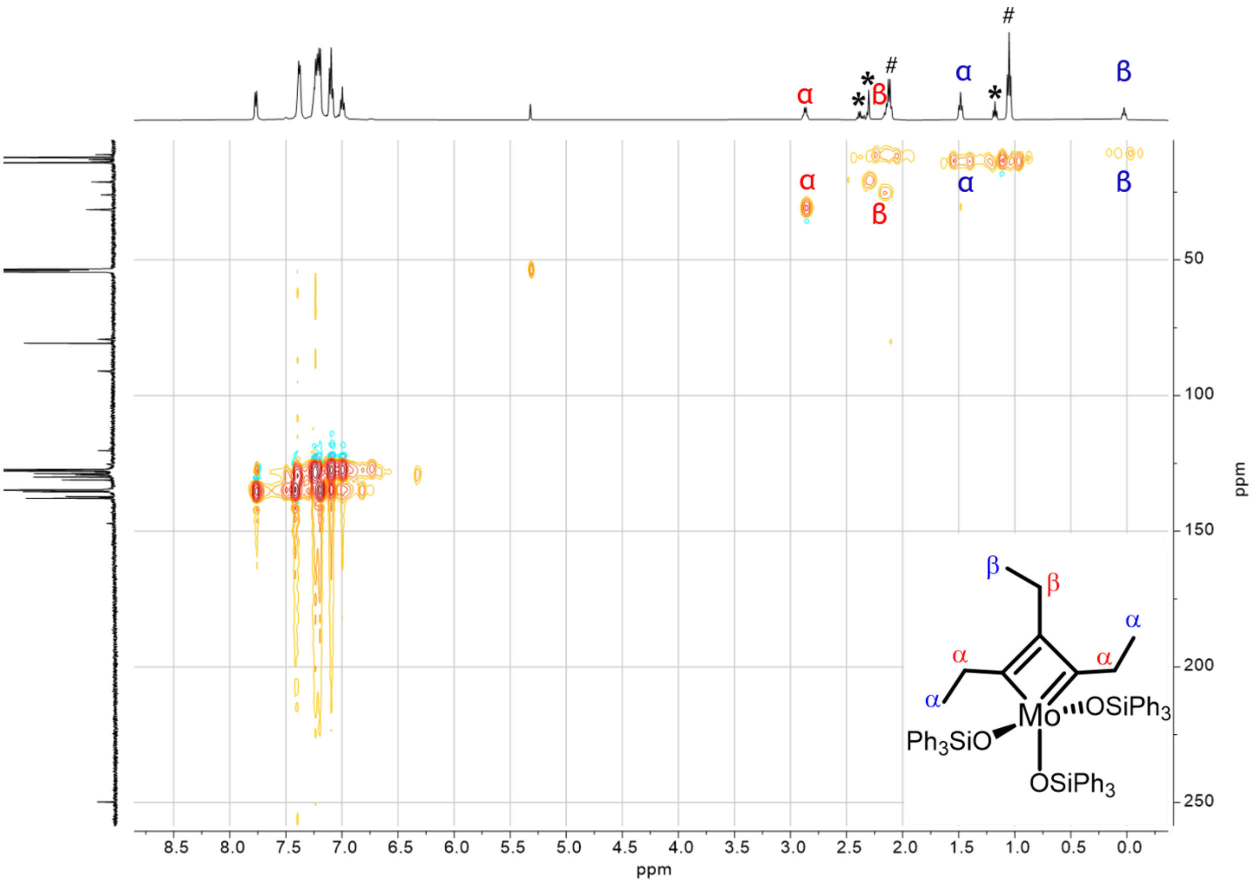

Figure S15. HSQC NMR (400 MHz, CD_2Cl_2, -70 °C) spectrum of **MCBD1**. * Denotes 4-tolyl-1-butyne byproduct and # denotes 3-hexyne.

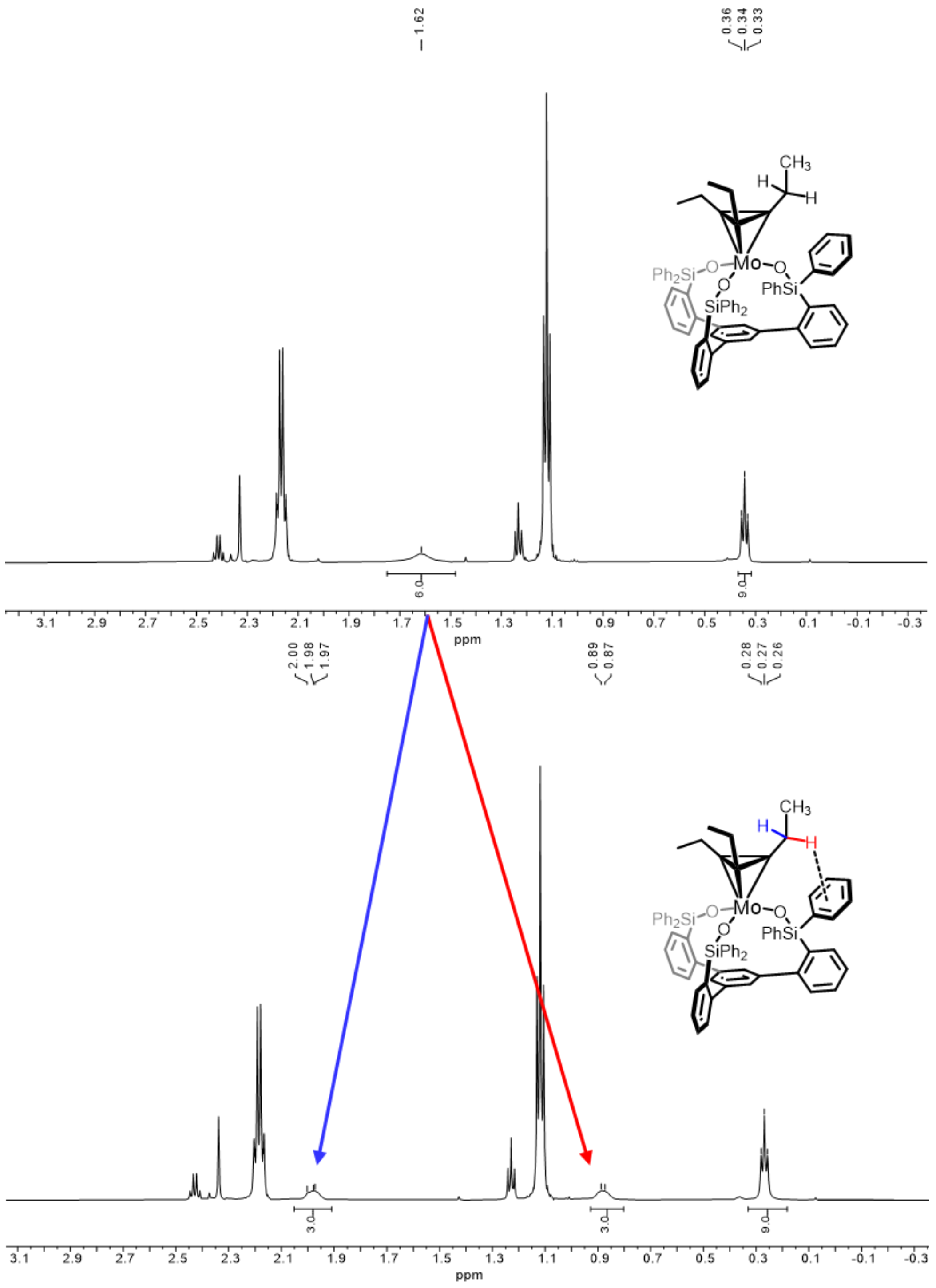

Figure S16. ^{1}H NMR ($CDCl_3$), 13 °C (top) and -60 °C (bottom) spectra of **Mt$_d$1** showing the splitting of the diastereotopic methylene protons with the C-H···π interactions strongly shielding one set.

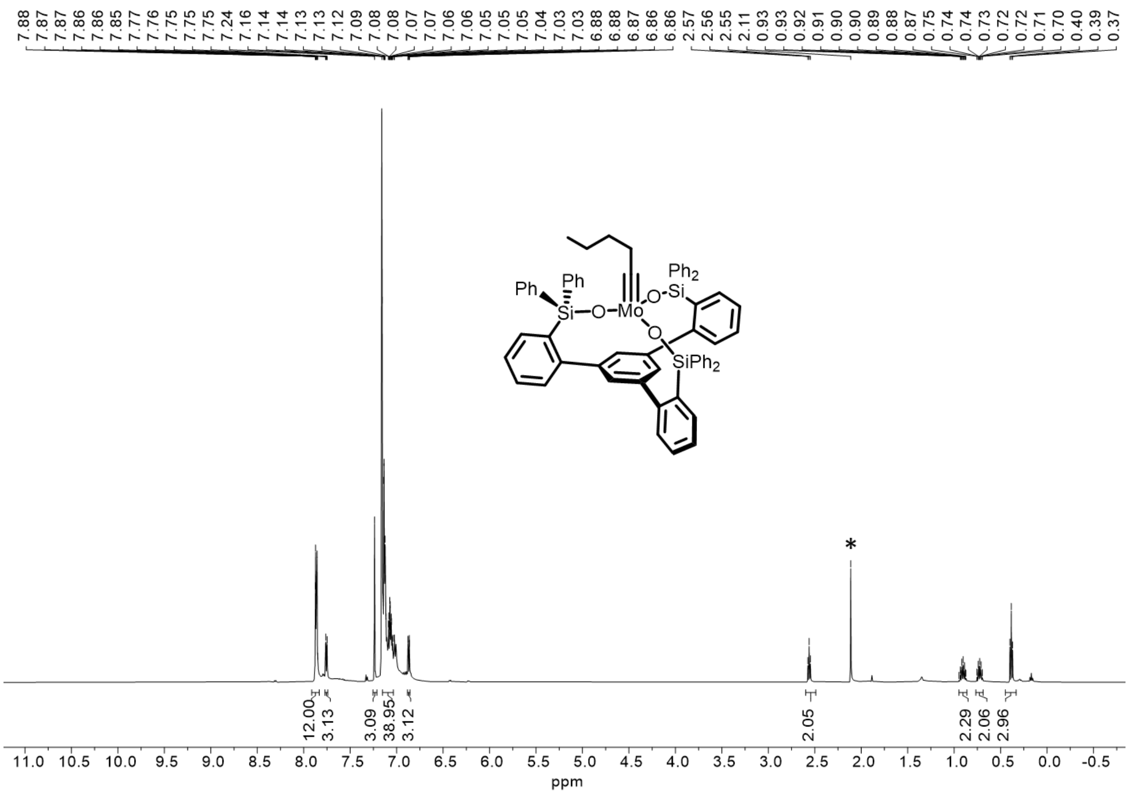

Figure S17. ^{1}H NMR (500 MHz, C_6D_6, 25 °C) spectrum of **Cat7**. * Denotes toluene impurity

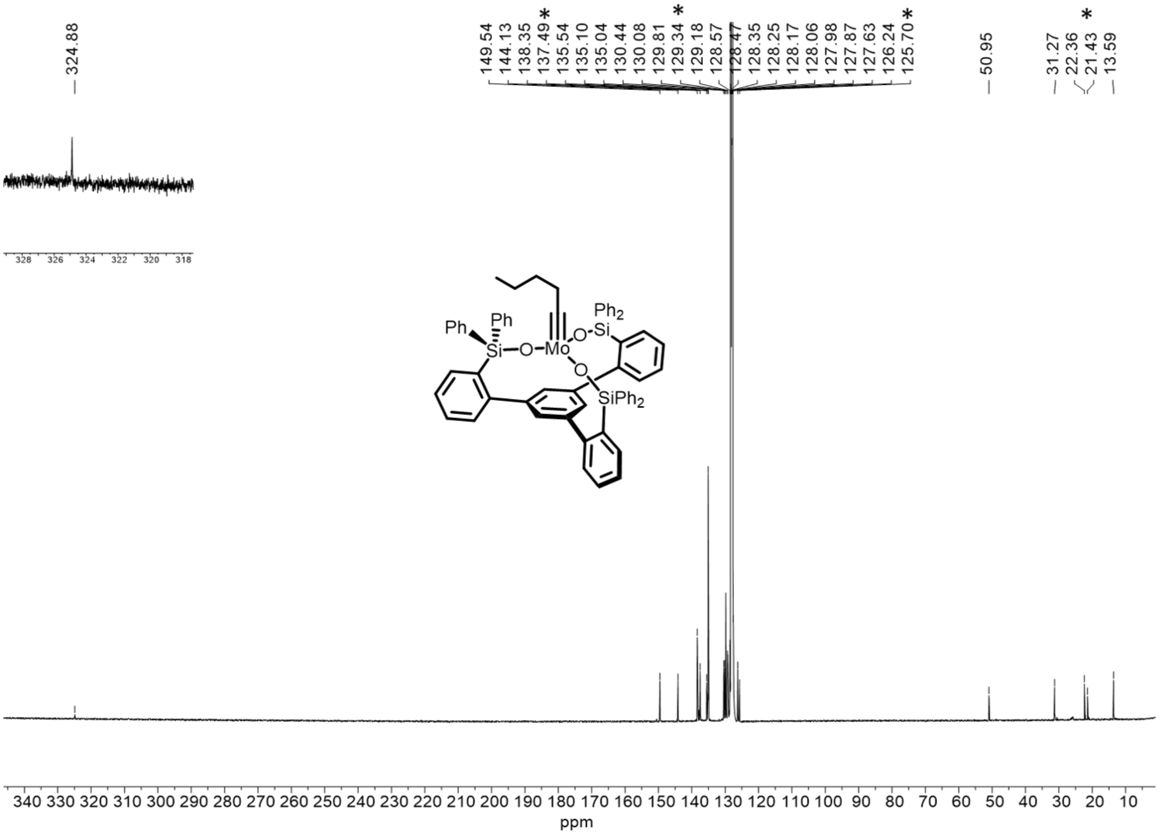

Figure S18. ^{13}C NMR (125 MHz, C_6D_6, 25 °C) spectrum of **Cat7**. * Denotes toluene impurity

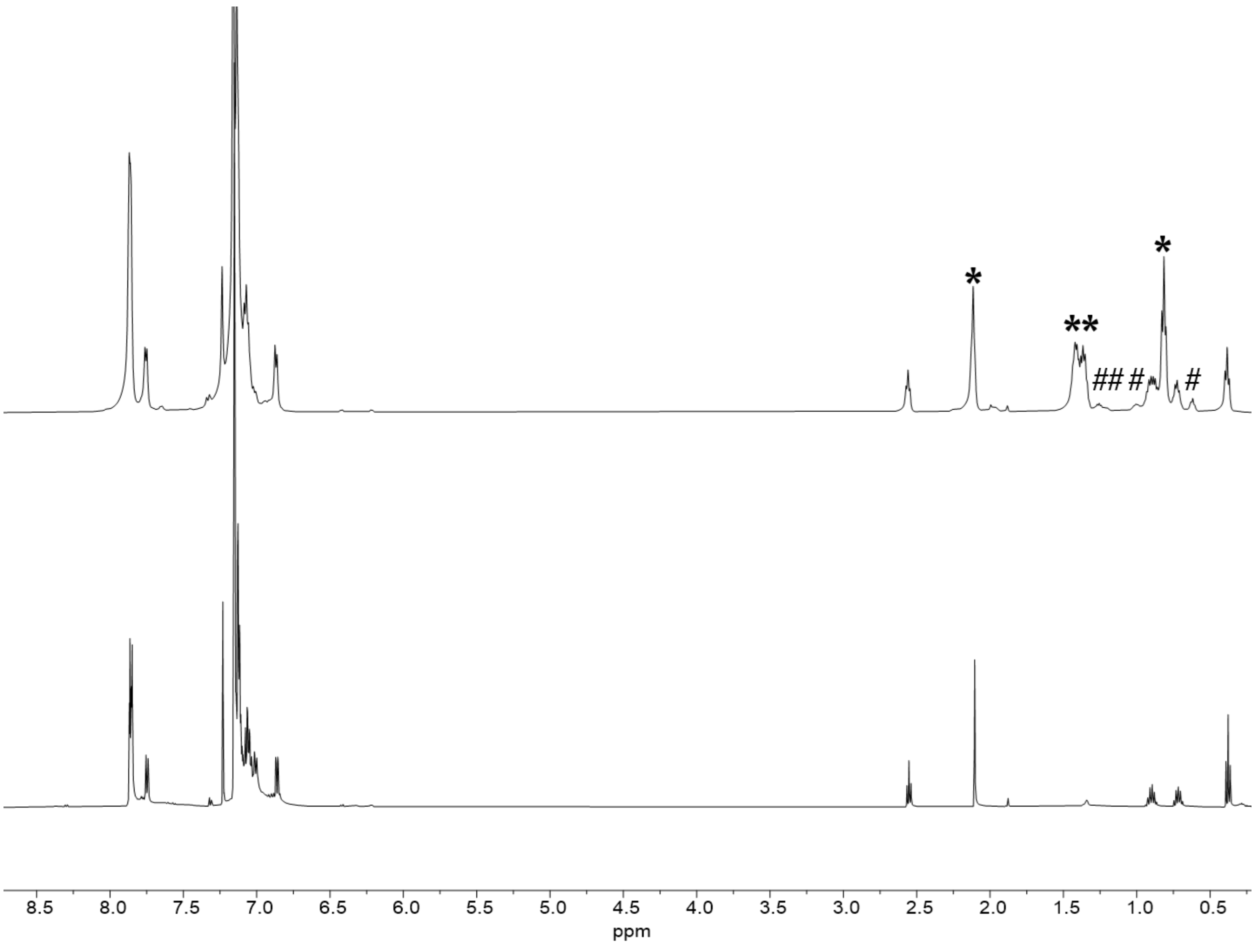

Figure S19. ^{1}H NMR (500 MHz, C_6D_6, 25 °C), of pure **Cat7** (bottom) and after adding 10 eq of 5-decyne (Top). **Mt_d2** is denoted by # and free 5-decyne is denoted by *.

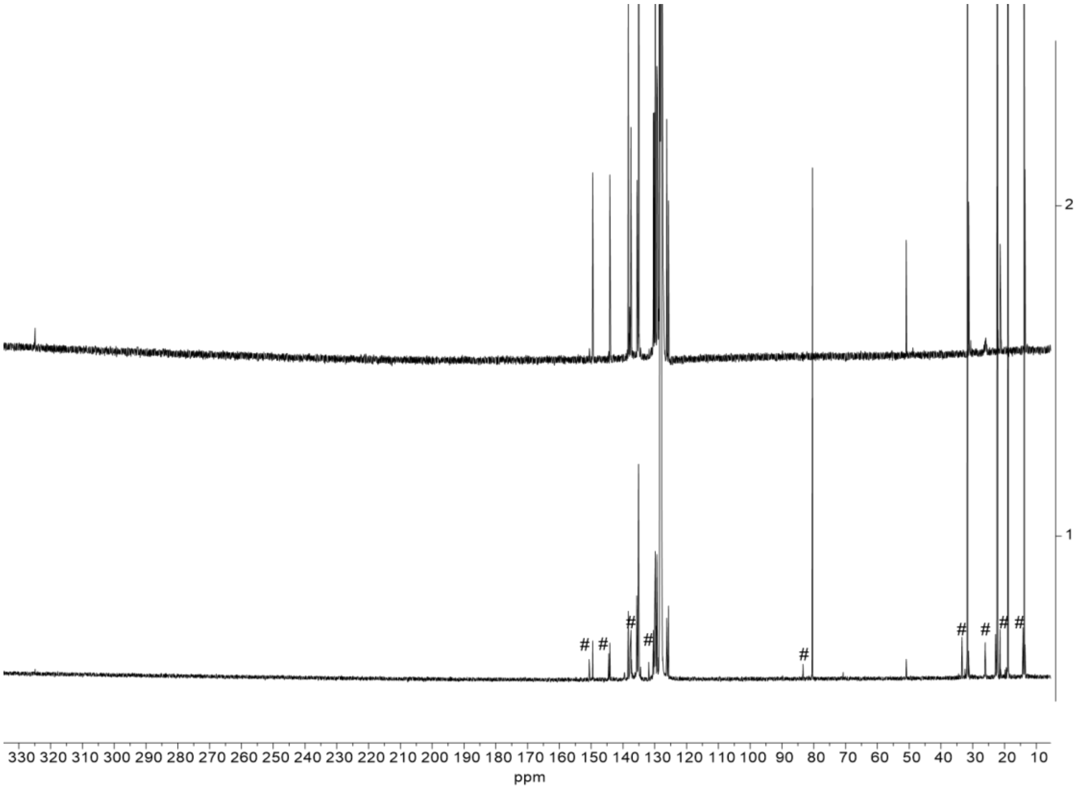

Figure S20. ^{13}C NMR (125 MHz, C_6D_6, 25 °C), of pure **Cat7** (top) and after adding 10 eq of 5-decyne (bottom). **Mt$_d$2** is denoted with #, the C_3Bu_3 resonance is identified at 83.4 ppm.

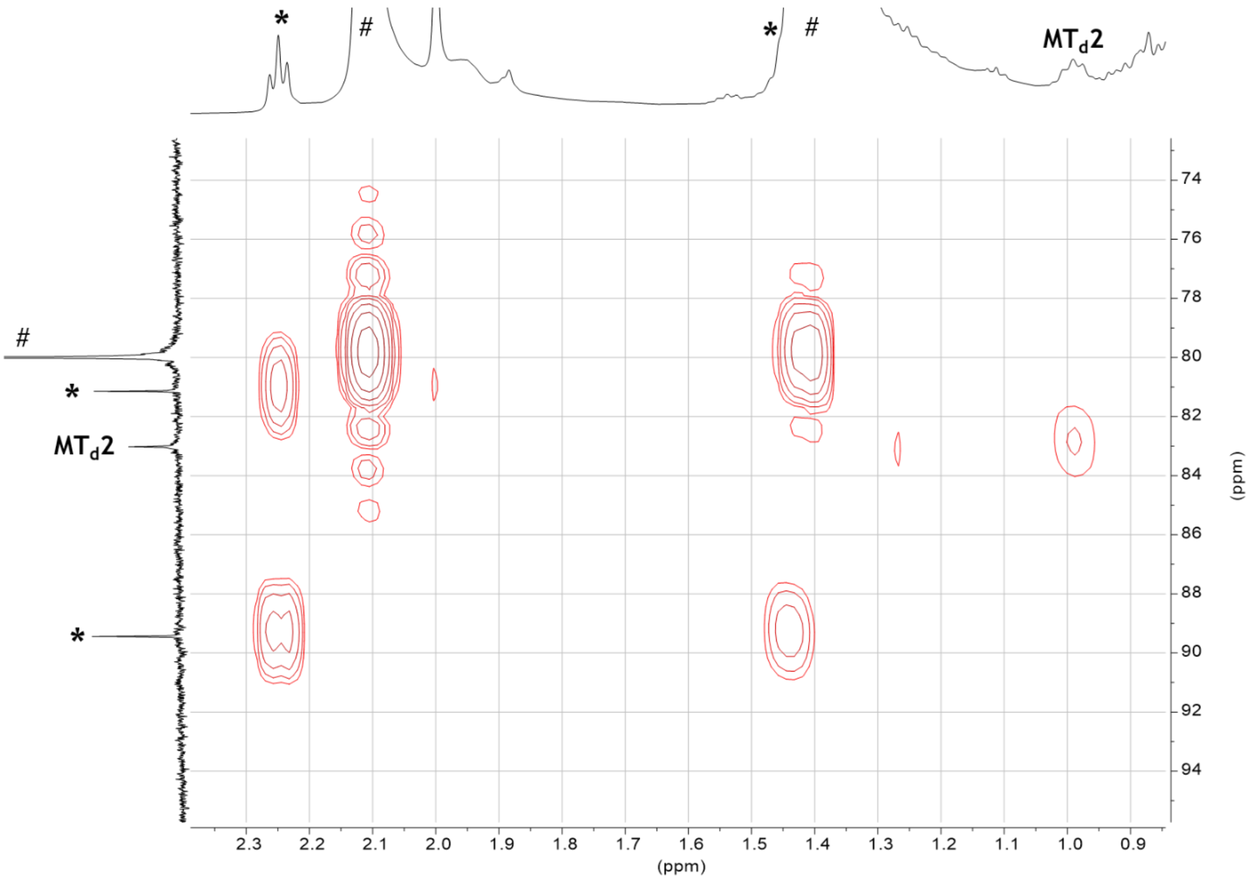

Figure S21. Long-range coupling ^{1}H-^{13}C gHMBC NMR (500 MHz, C_6D_6, 25 °C) spectrum of **MT$_d$2** generated in-situ by the addition of excess 5-decyne (#) to a solution of **Cat3.** 4-tolyl-1-hexyne impurity is denoted by *.

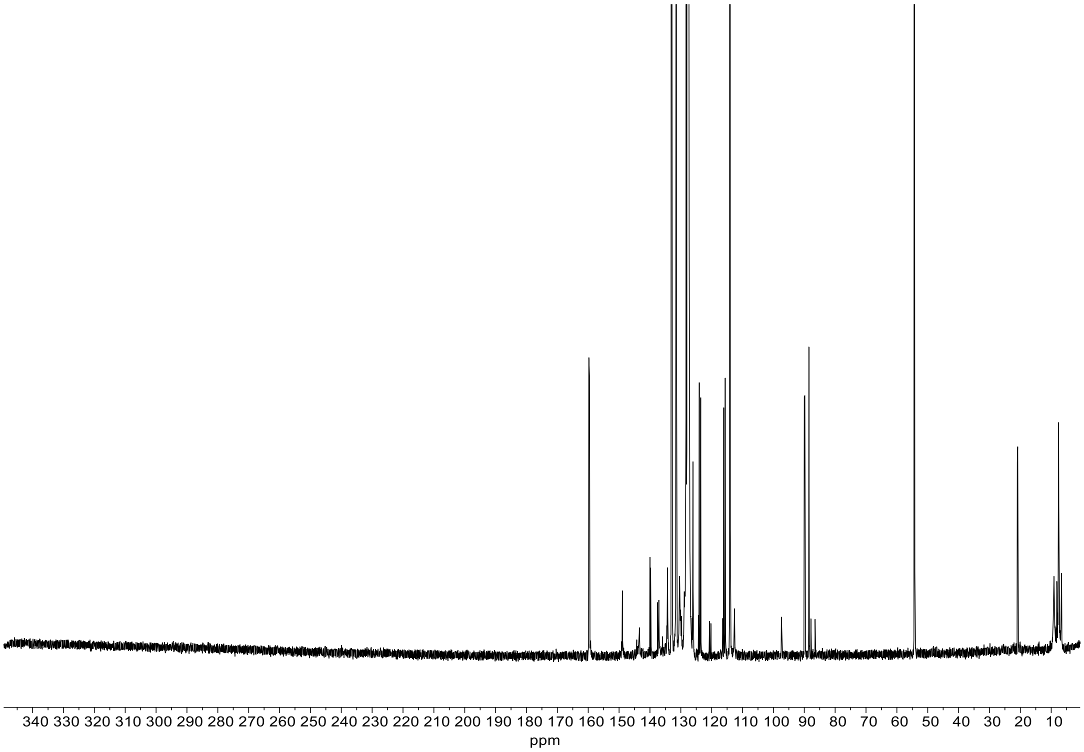

Figure S22. ^{13}C NMR (C_6D_6) of the reaction of **Cat5** with 6 eq. of 1-methoxy-4-(phenylethynl)benzene. The absence of resonances between 220-280 suggests that an all aryl MCBD is not the resting state.

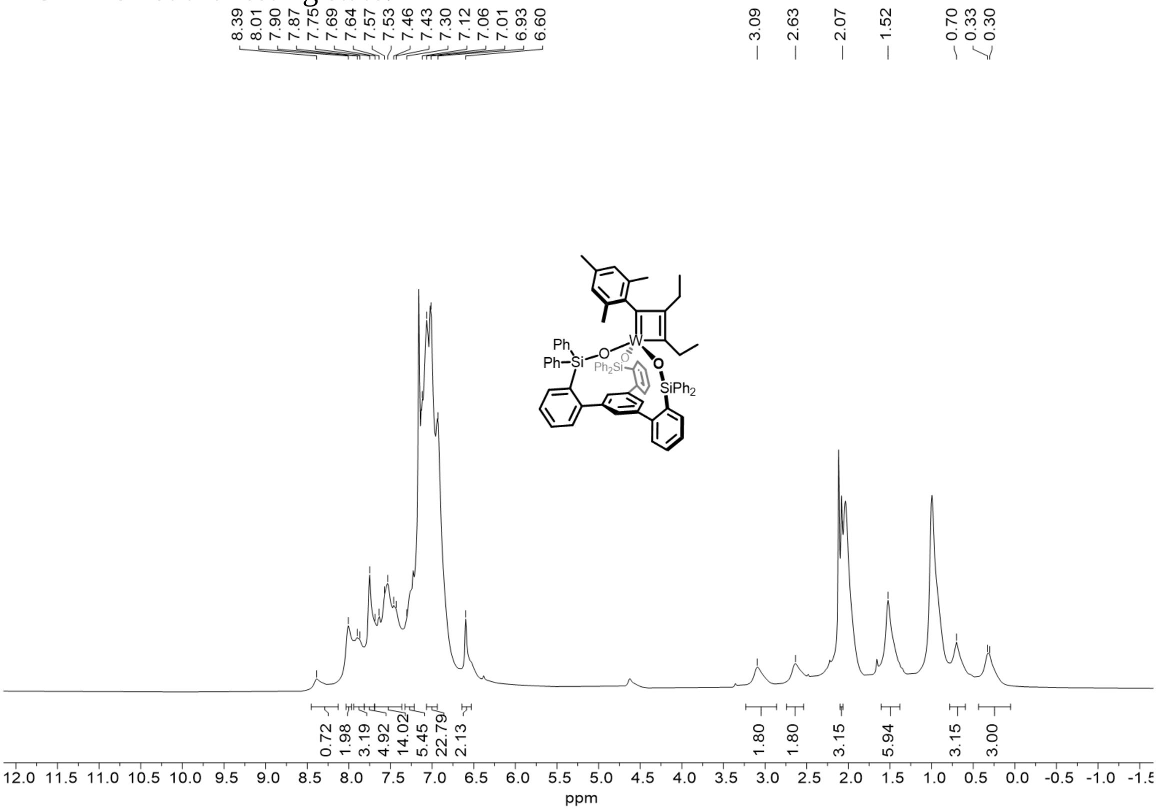

Figure S23 ^{1}H NMR (500 MHz, C_6D_6, 25 °C) spectrum of **MCBD2**.

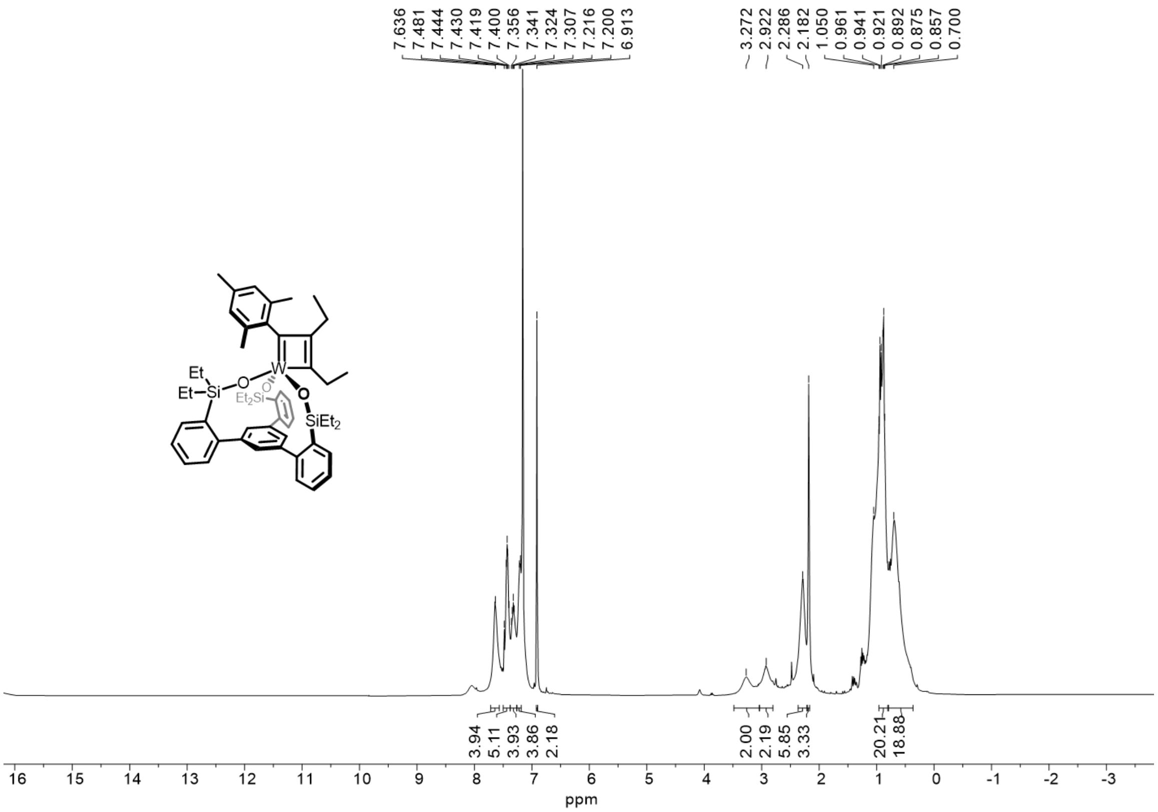

Figure S24. ^{1}H NMR (400 MHz, C_6D_6, 25 °C) spectrum of **MCBD3**.

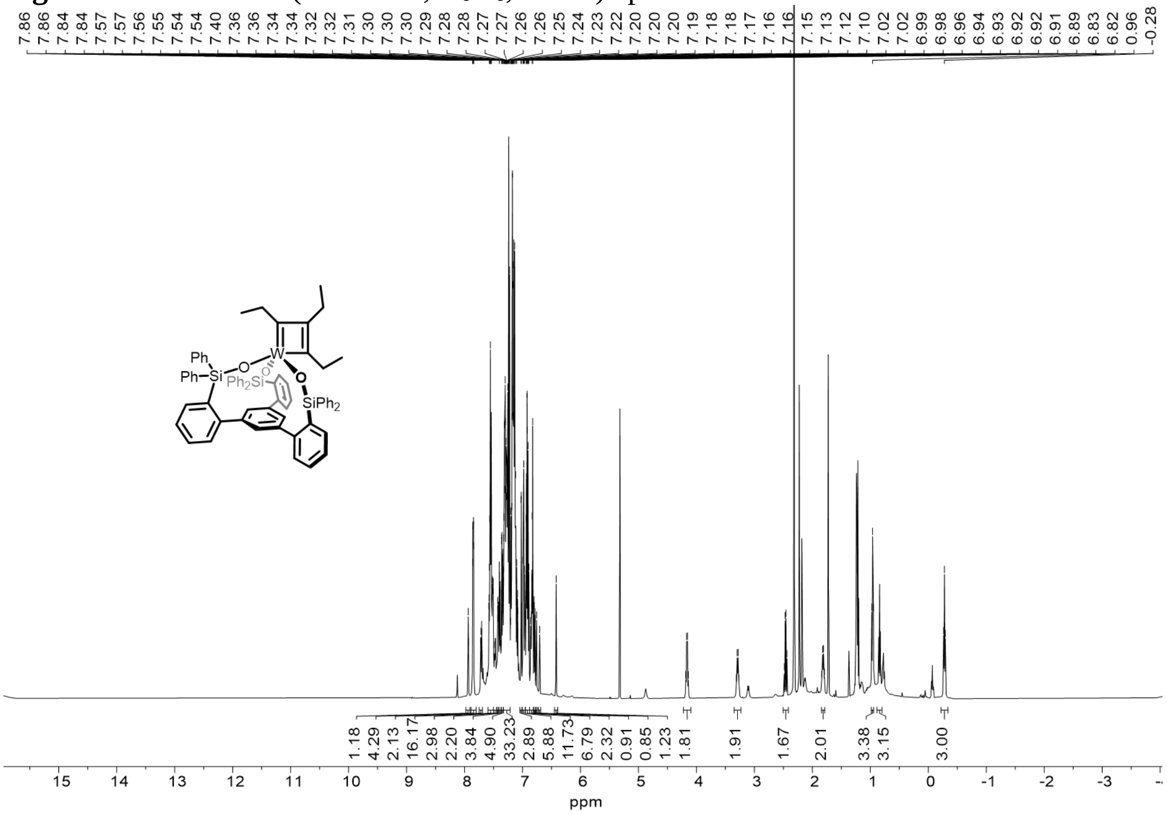

Figure S25. ^{1}H NMR (500 MHz, CD_2Cl_2, -70 °C) spectrum of **MCBD4**.

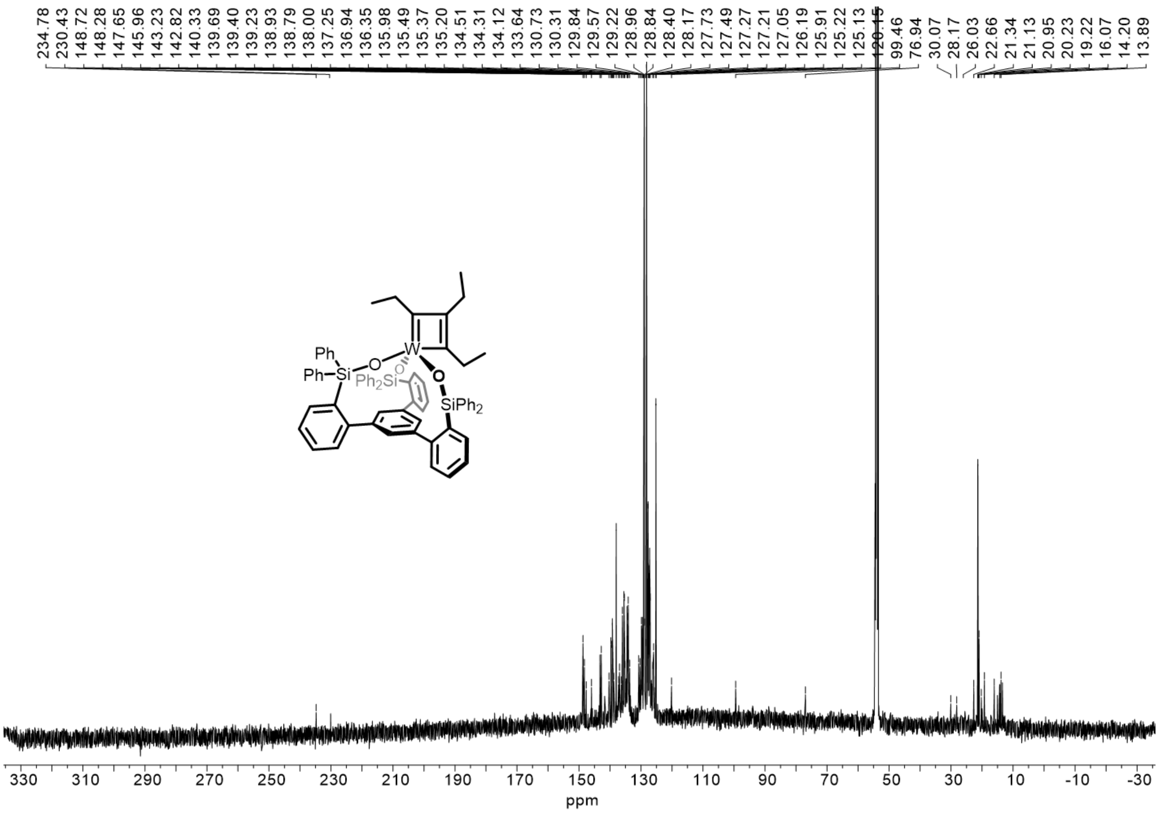

Figure S26. ^{13}C NMR (125 MHz, CD_2Cl_2, -70 °C) spectrum of **MCBD4**.

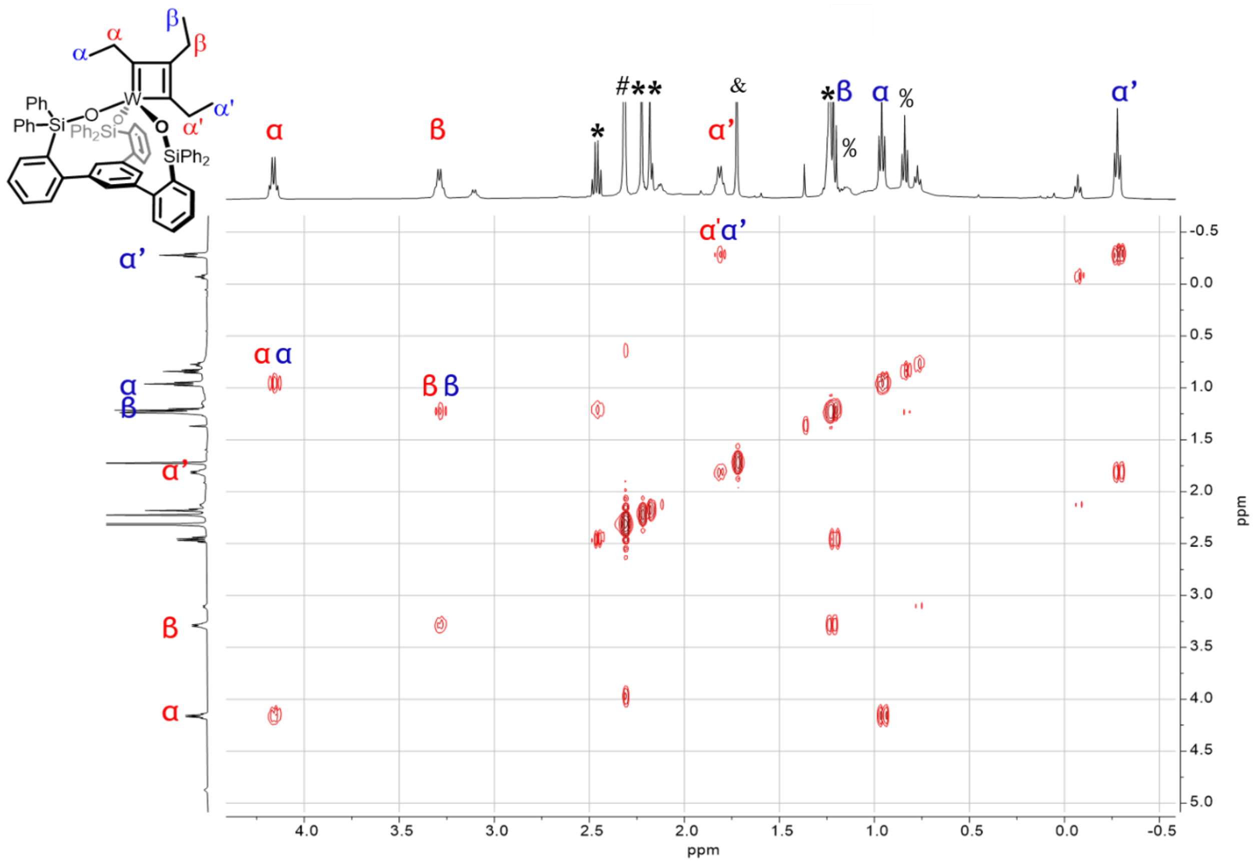

Figure S27. ^{1}H-^{1}H Through-bond coupling gCOSY NMR (500 MHz, CD_2Cl_2, -70 °C) spectrum of **MCBD4**. * Denotes mesityl-1-butyne byproduct, # denotes toluene impurity, % denotes pentane impurity and & denotes a minor impurity which forms overtime within 3-hexyne.

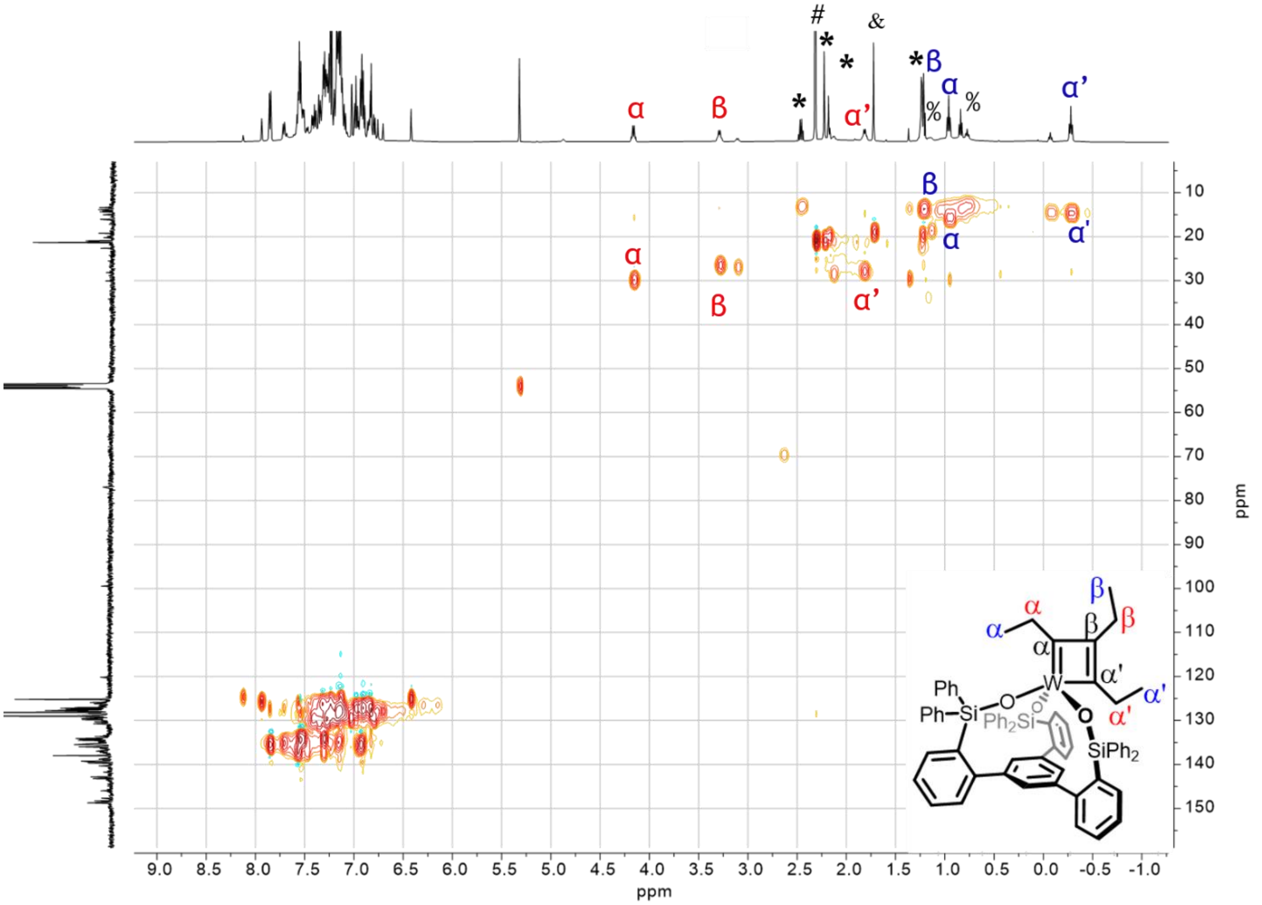

Figure S28. One-bond coupling ^{1}H-^{13}C gHSQC NMR (500 MHz, CD_2Cl_2, -70 °C) spectrum of **MCBD4.** * Denotes mesityl-1-butyne byproduct, # denotes toluene impurity, % denotes pentane impurity and & denotes a minor impurity which forms overtime within 3-hexyne.

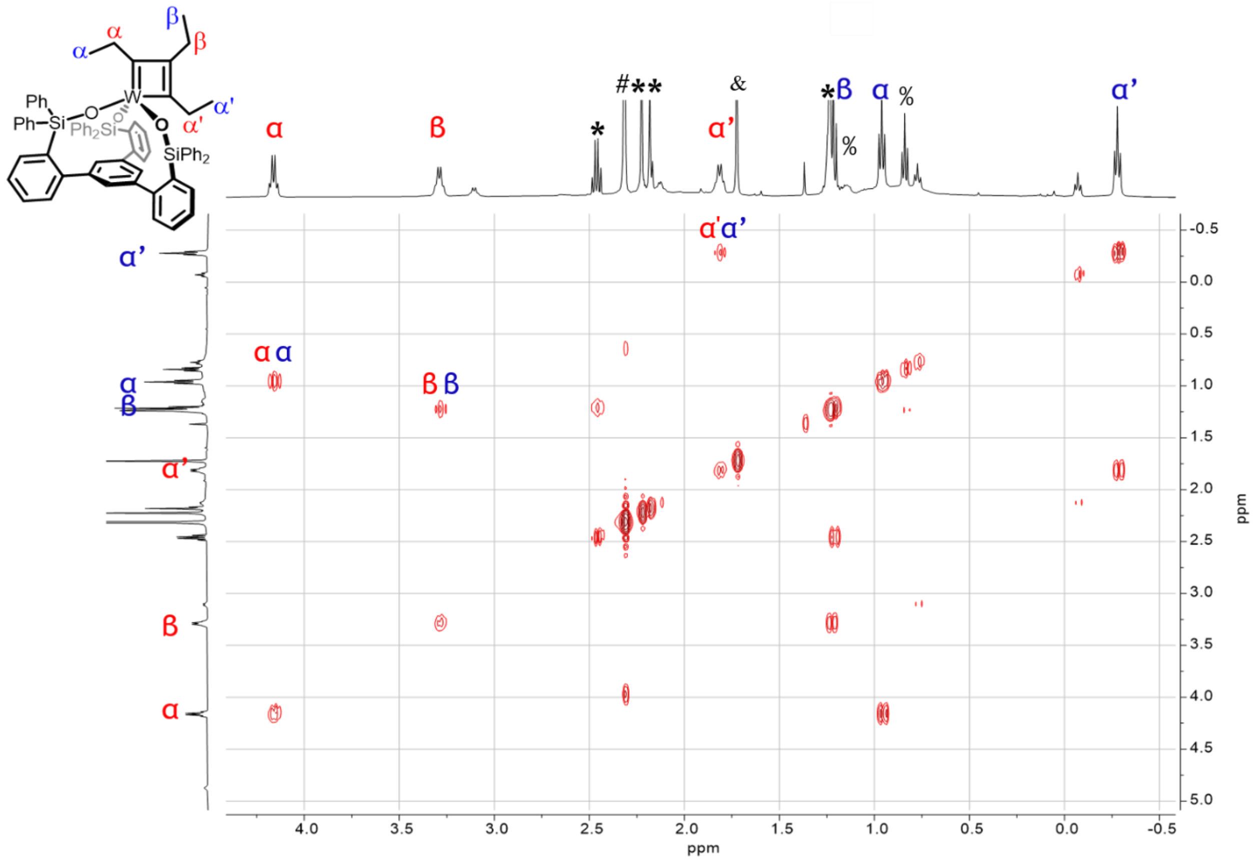

Figure S29. Long-range coupling ^1H-^{13}C gHMBC NMR (500 MHz, CD_2Cl_2, -70 °C) spectrum of **MCBD4.** * Denotes mesityl-1-butyne byproduct, # denotes toluene impurity, % denotes pentane impurity and & denotes a minor impurity which forms overtime within 3-hexyne.

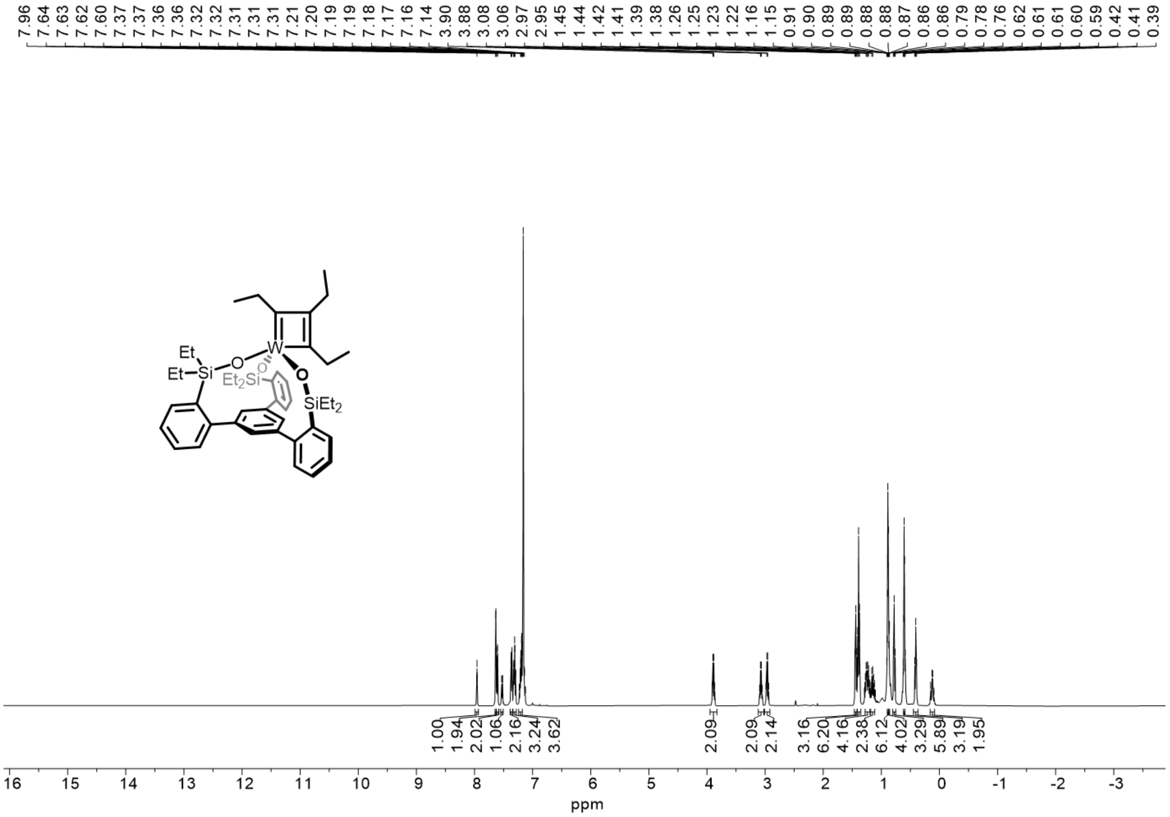

Figure S30. ^{1}H NMR (500 MHz, C_6D_6, 25 °C) spectrum of **MCBD5**.

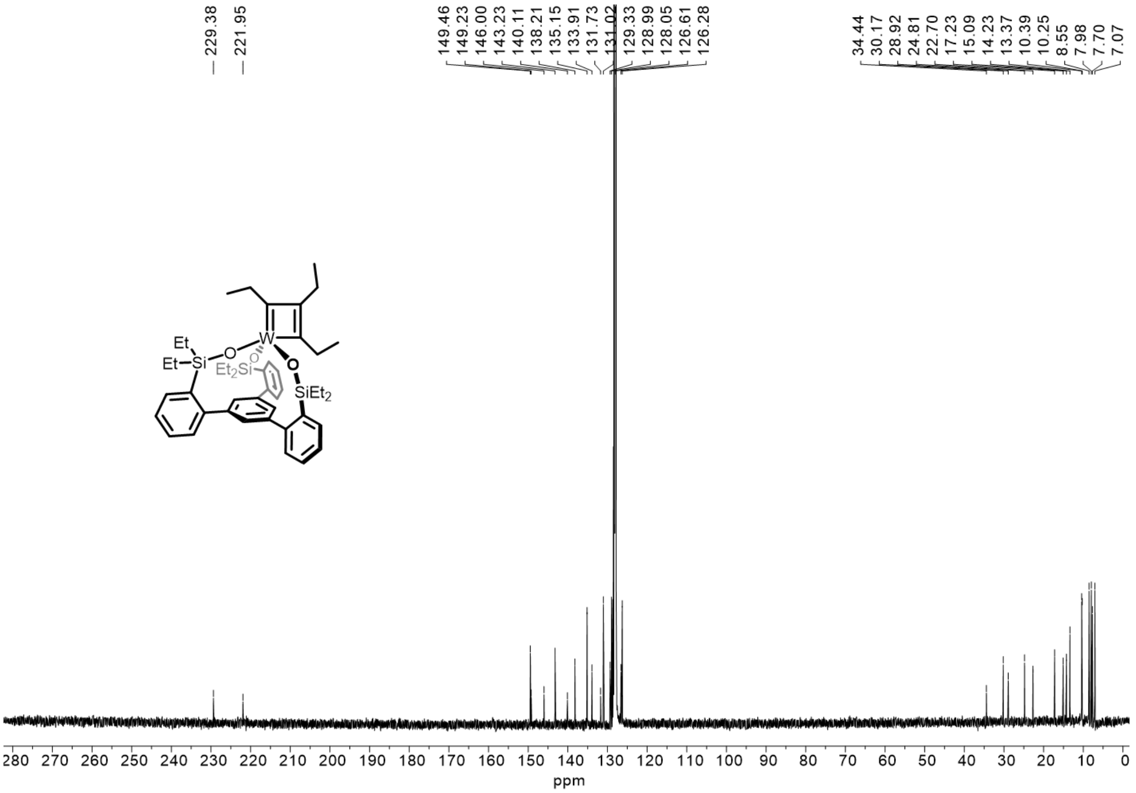

Figure S31. ^{13}C NMR (125 MHz, C_6D_6, 25 °C) spectrum of **MCBD5**.

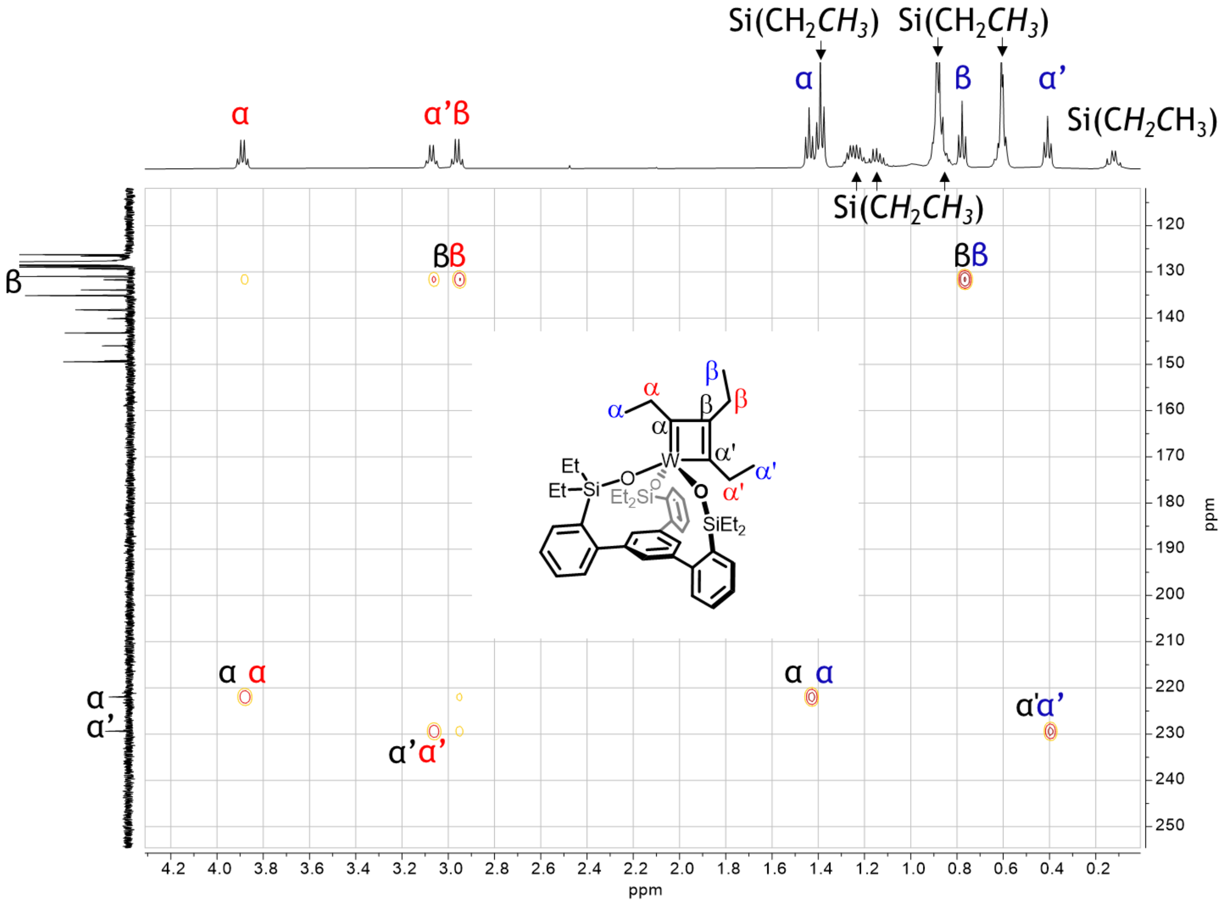

Figure S32. [1]H-^{1}H Through-bond coupling gCOSY NMR (500 MHz, C_6D_6, 25 °C) spectrum of **MCBD5**.

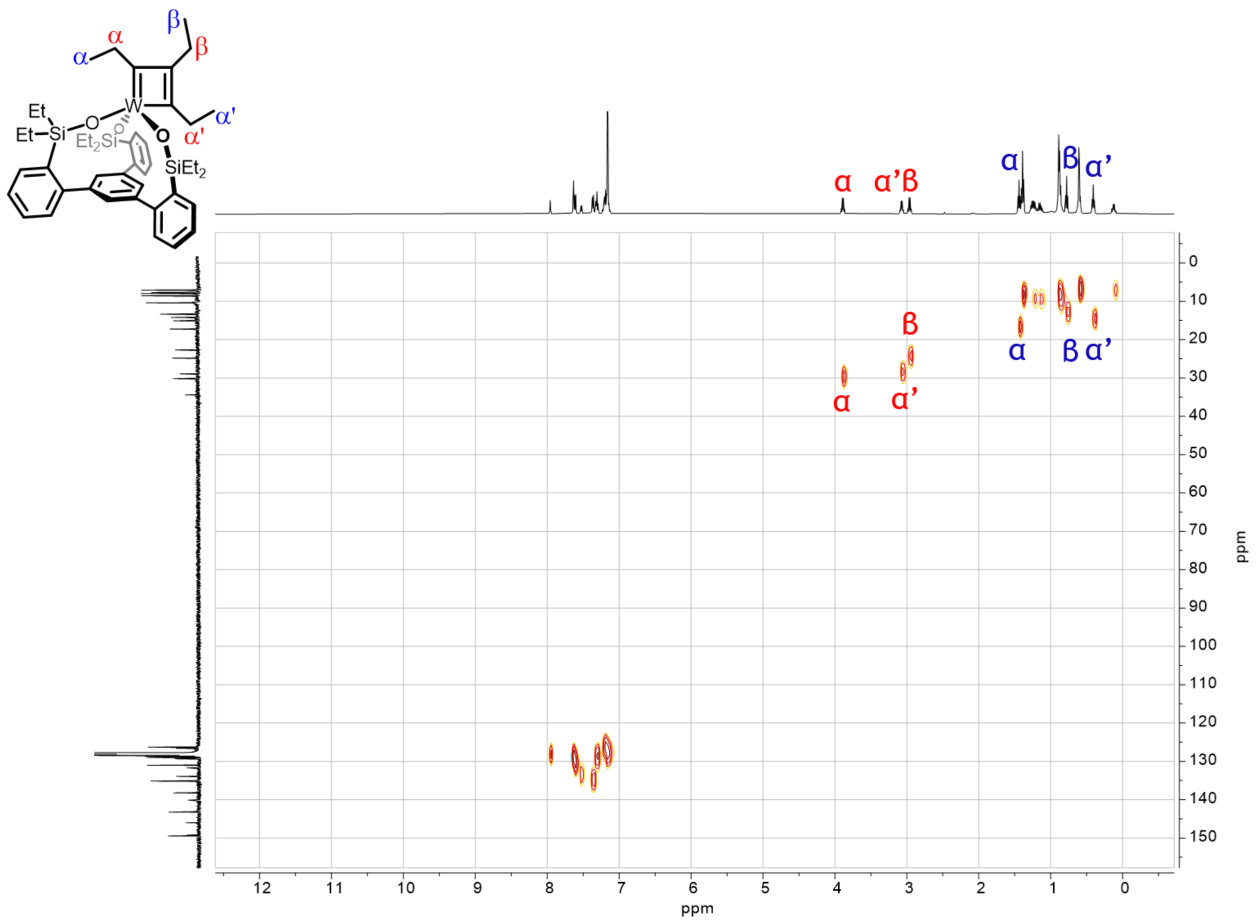

Figure S33. One-bond coupling ^1H-^{13}C gHSQC NMR (500 MHz, C_6D_6, 25 °C) spectrum of **MCBD5.**

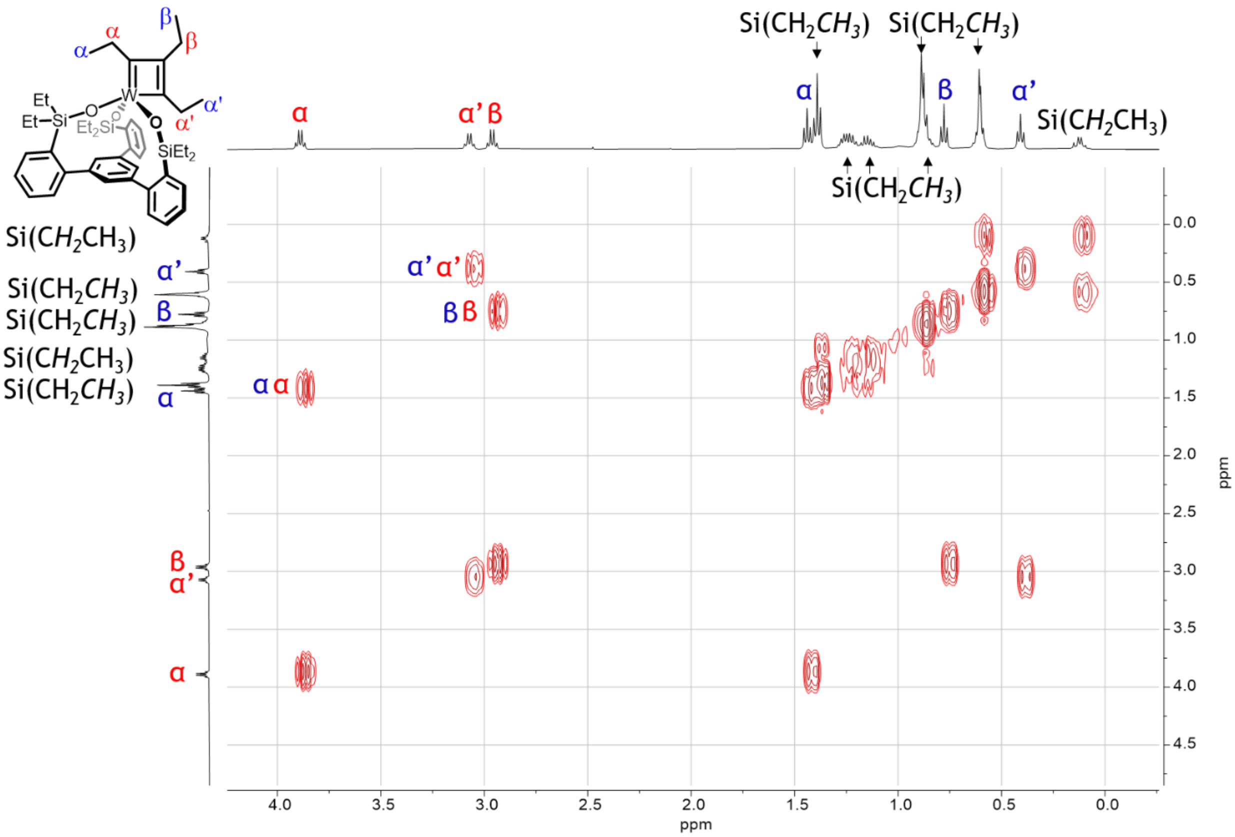

Figure S34. Long-range coupling ^{1}H-^{13}C gHMBC NMR (500 MHz, C_6D_6, 25 °C) spectrum of **MCBD5.**

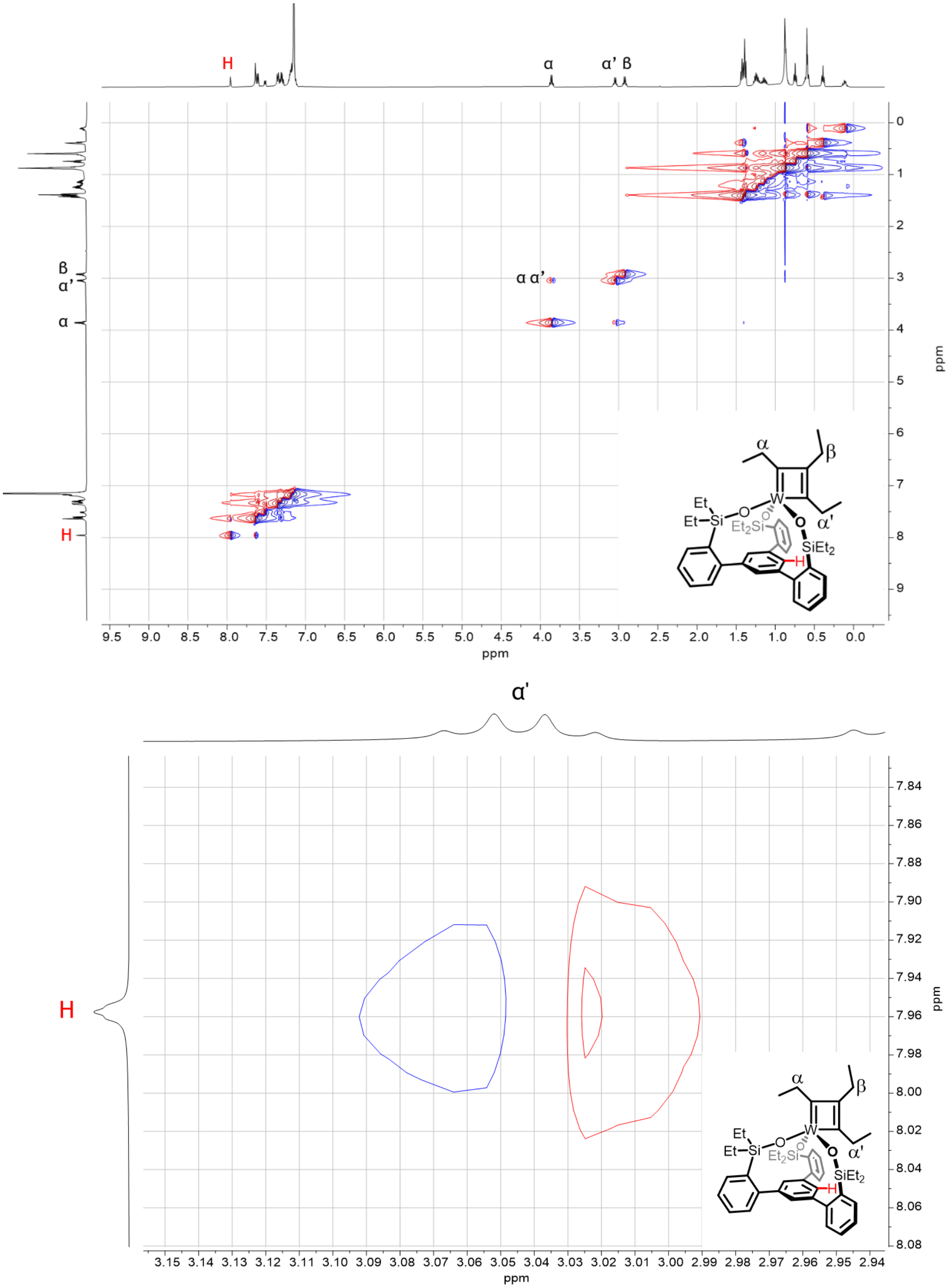

Figure S35. Long-range coupling ^{1}H-^{13}C gNOESY NMR (500 MHz, C_6D_6, 25 °C) spectrum of **MCBD5** (Top). Expanded portion showing correlation of [1]H α’ CH_2 with the basal arene proton. (Bottom).

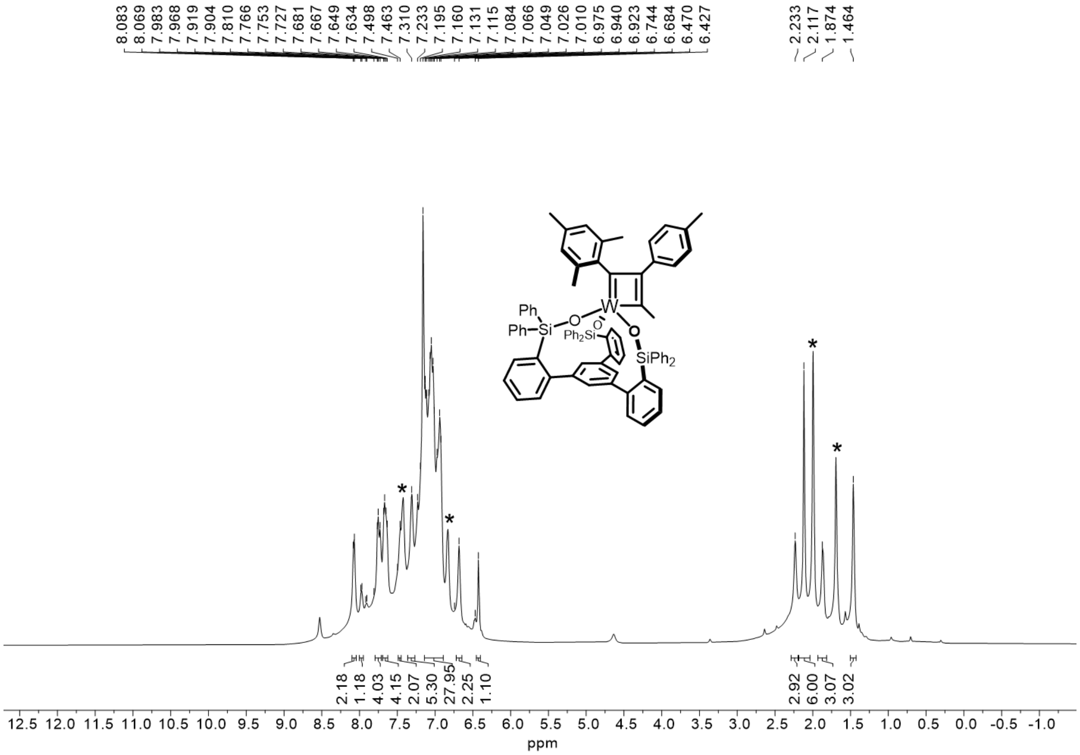

Figure S36. ^{1}H NMR (500 MHz, C_6D_6, 25 °C) spectrum of **MCBD6**. * Denotes excess *p*-tolylpropyne.

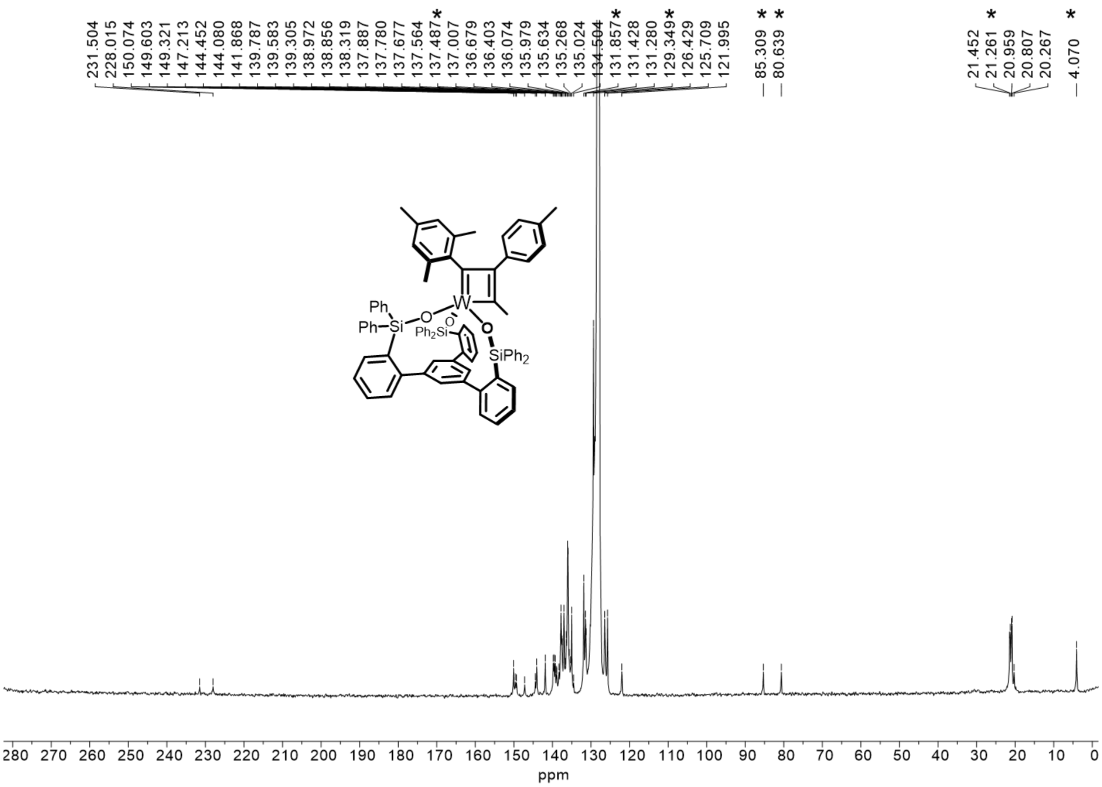

Figure S37. ^{13}C NMR (125 MHz, C_6D_6, 25 °C) spectrum of **MCBD6**. * Denotes excess *p*-tolylpropyne.

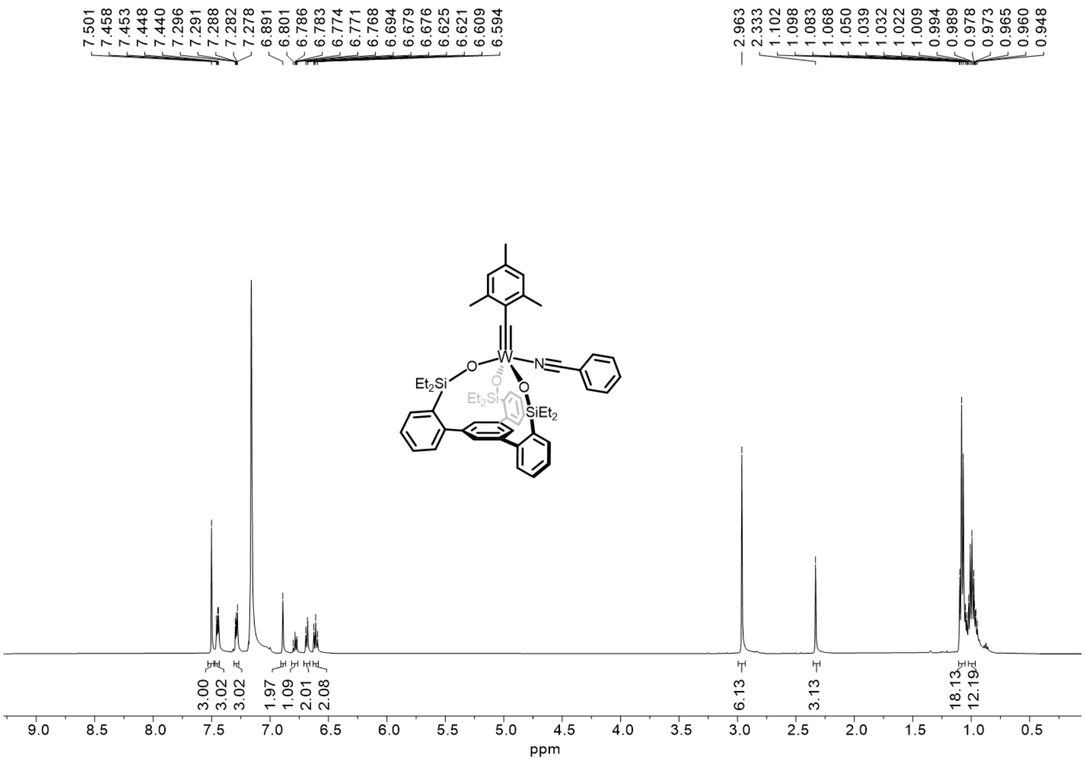

Figure S38. ^{1}H NMR (500 MHz, C_6D_6, 25 °C) spectrum of **Cat5●PhCN**.

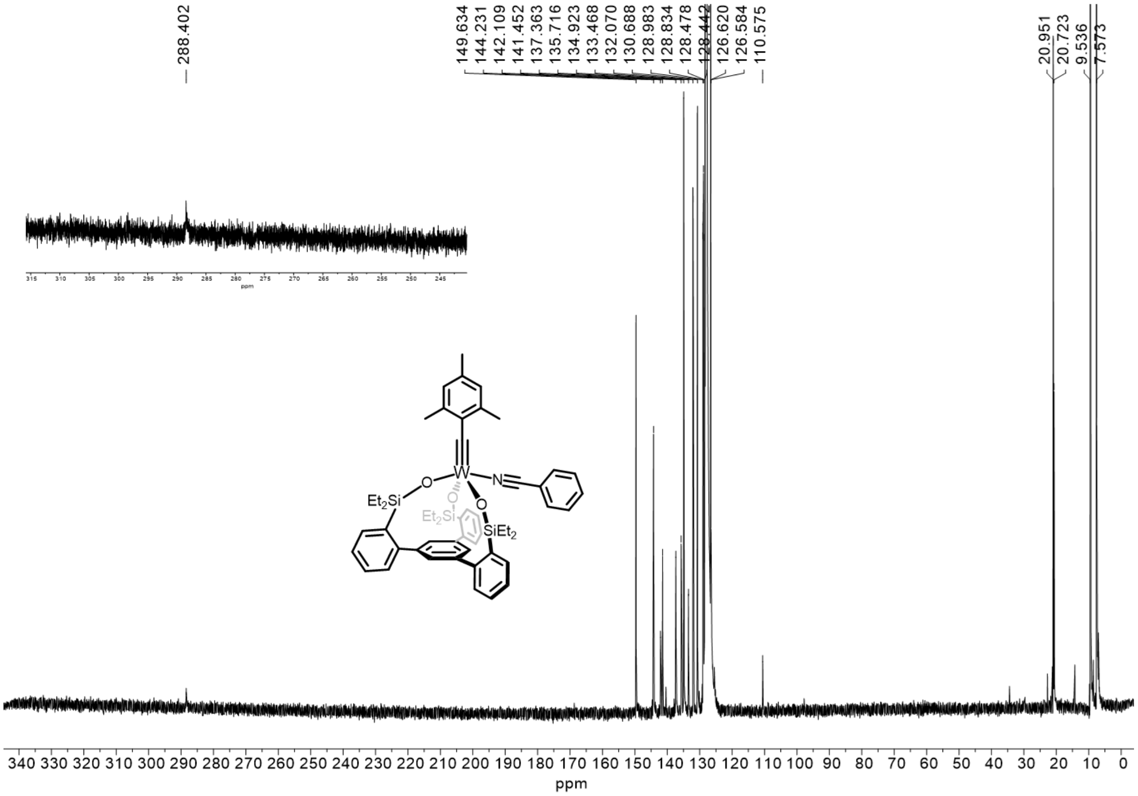

Figure S39. ^{13}C NMR (125 MHz, C_6D_6, 25 °C) spectrum of **Cat5●PhCN**.

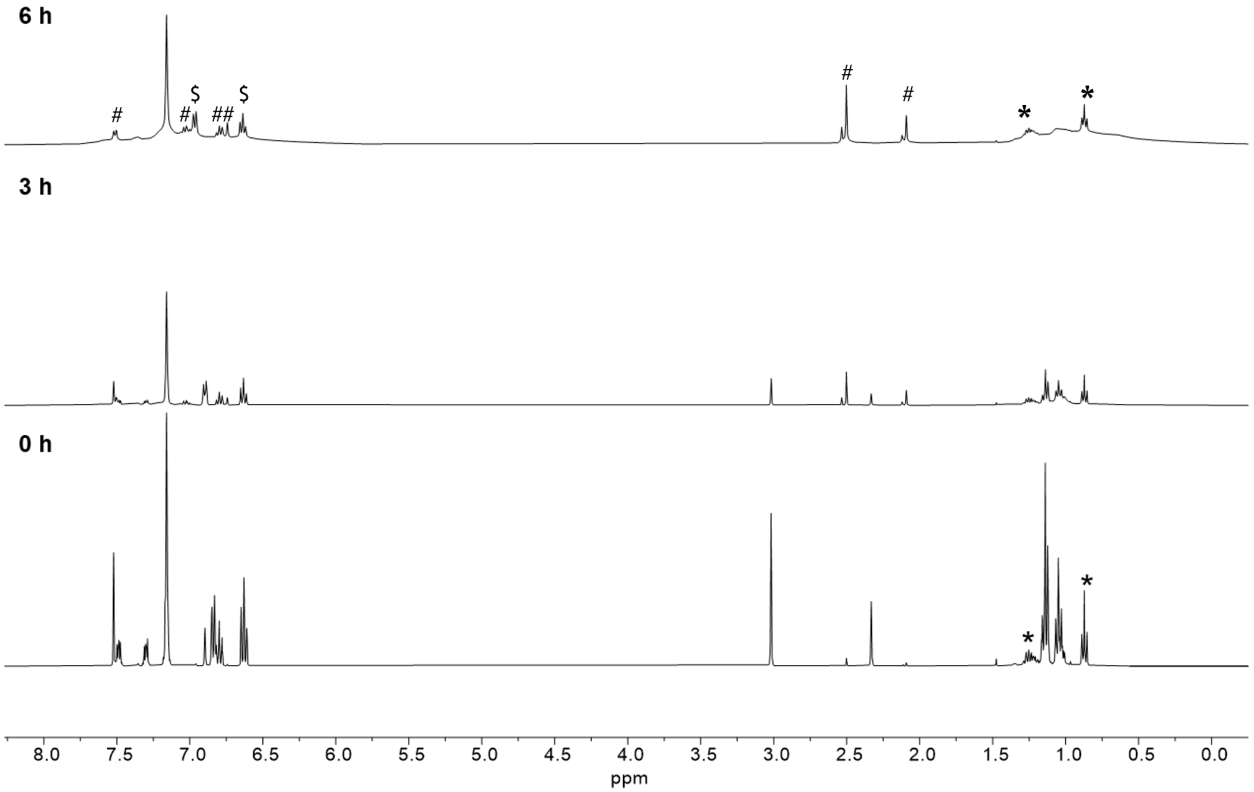

Figure S40. ^{1}H NMR (400 MHz, C_6D_6, 25 °C) decomposition of **Cat5●PhCN** to **Nitride1** and mesityl-phenylacetylene over the course of 6 h. * Denotes pentane impurity, # denotes mesityl-phenylacetylene and $ denotes residual benzonitrile.

Rate of Alkyne Metathesis using Mo(VI) and W(VI)-based SiP Catalysts

Under an inert atmosphere, a 0.2 mM/0.002 mM solution of substrate/catalyst was prepared in 0.6 mL C_6D_6. The percentage of substrate conversion was monitored by integration of the OCH_3 resonance periodically at set time points. The MestraNova fitting function was used to assist in the integration.

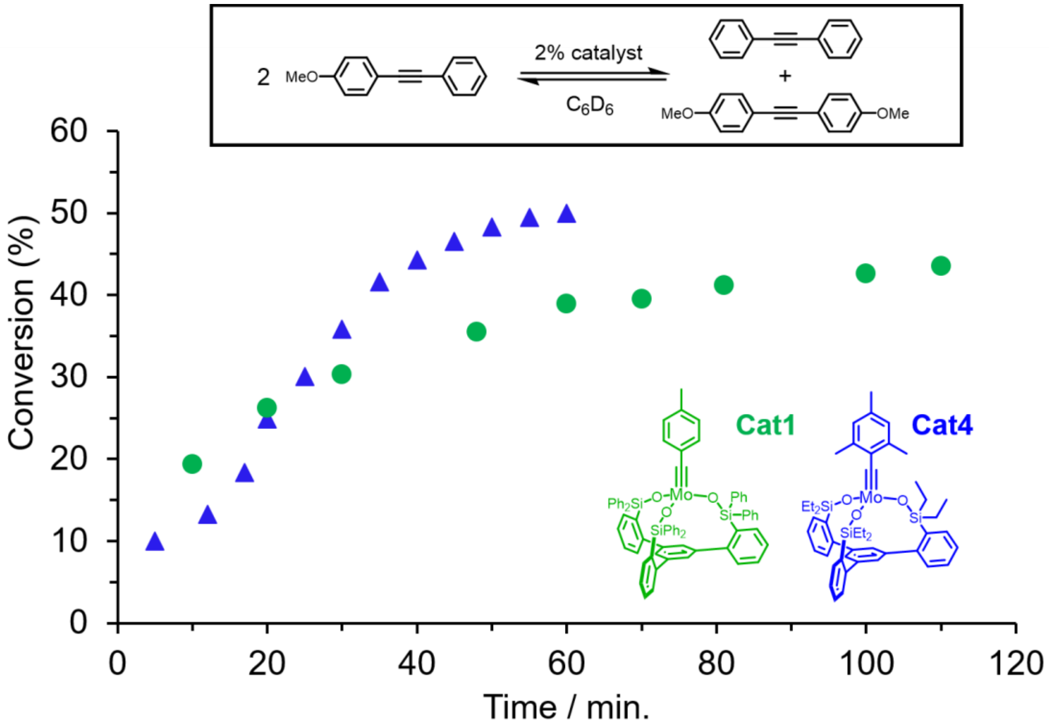

Figure S41. Dynamic scrambling of 1-methoxy-4-(phenylethynl)benzene (0.1 mM in C_6D_6) catalyzed by 2 mol% of **Cat1** and **Cat4** at rt monitored by ^{1}H NMR.

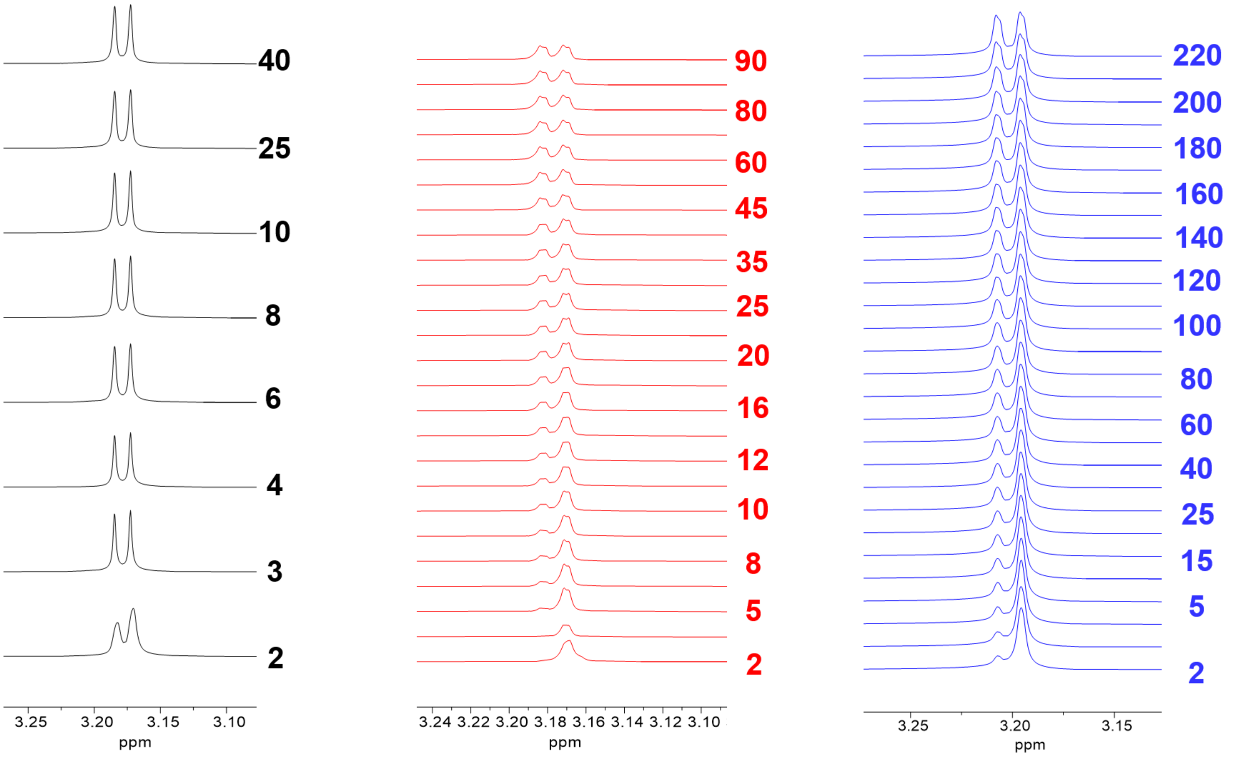

Figure S42. Stacked ^{1}H NMR plots for kinetics experiments using **Cat5** (Left, Black), **Cat3** (Center, Red) and **Cat 4** (Right, Blue). Integrations were obtained by "Generalized Lorentzian" fitting in mnova NMR software.

S.M.	T (°C)	Yield[a]
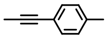	r.t.	96%
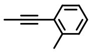	85	99%
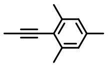	85	20%
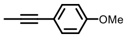	r.t.	90%
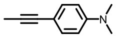	r.t.	83%
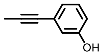	r.t.	0
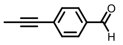	85	0
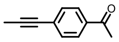	r.t.	0
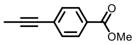	r.t.	0
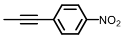	85	0
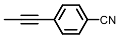	85	14%
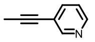	85	0
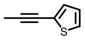	85	20%

a 2 mol% **Cat5**; all yields are isolated yields

Figure S43. Substrate Scope using **Cat5**.

Crystallographic Information

Crystallographic data are summarized in Tables S1-S4. Suitable crystals for X-ray analysis of **SiP^{Et}, Pre2, Cat3-5, MT$_d$2, MCBD2-6, Cat5●PhCN** and **Nitride1** were placed MiTeGen pins, coated in oil. The X-ray intensity data collection was carried out on a Bruker APEXII DUO CCD area detector using graphite-monochromated Mo-Kα radiation (λ = 0.71073 Å) or Cu-Kα radiation (λ = 1.54184 Å) at 90.0(5) K. Frames were integrated using SAINT,[6] producing a listing of non-averaged F^2 and $\sigma(F^2)$ values. The intensity data were corrected for Lorentz and polarization effects and for absorption using SADABS.[7] The initial structure was determined by intrinsic phasing using SHELXT.[8] The further structure determination was performed by difference Fourier methods and refined by full-matrix least squares using SHELXL[9] or olex2.refine for **Nitride1**. All reflections were used during refinements. Non-hydrogen atoms were refined anisotropically and hydrogen atoms were visible in difference maps, but placed in idealized positions and treated with riding models. Disordered phenyl groups successfully modeled. Disordered solvent was removed using the SQUEEZE procedure for **Cat3, MCBD4** and **MT$_d$2**.

Table S1. Crystallographic Data for SiP^{Et}, Cat3-5

	SiP^{Et}	Cat3	Cat4	Cat5
Molecular Formula	$C_{72}H_{96}O_6Si_6$	$C_{70}H_{56}O_3Si_3W$	$C_{46}H_{56}MoO_3Si_3$	$C_{46}H_{56}O_3Si_3W \cdot (C_5H_{12})_{0.5}$
Fw	1226.02	1489.67	837.11	961.10
Temp(K)	90.0(5)	90.0(5)	90.0(5)	90.0(5)
Crystal System	Triclinic	Triclinic	Monoclinic	Monoclinic
Space Group	P-1	P-1	$P2_1/n$	$P2_1/n$
Cell Constants				
a(Å)	8.9495(12)	13.3644(11)	12.2321(4)	21.4428(8)
b(Å)	11.8064(15)	13.7952(11)	23.6045(9)	16.0018(6)
c(Å)	35.142(4)	21.6377(17)	15.0674(5)	27.6488(10)
α(deg)	93.460(7)	105.889(3)	90	90
β(deg)	90.523(8)	95.015(3)	96.909(2)	104.890(2)
γ(deg)	111.376(7)	104.262(3)	90	90
Z	2	2	4	8
V(Å^3)	3449.5(8)	3666.9(5)	4318.9(3)	9168.4(6)
Abs Coeff, μ_{calc} (mm^{-1})	0.171	1.676	0.425	2.637
δ_{calc} (g/cm^3)	1.180	1.349	1.287	1.393
F(000) (e$^-$/Å^3)	1320	1532	1760	3944
Crystal Dimensions (mm)	1.330 x 0.830 x 0.550	0.14 x 0.13 x 0.09	0.17 x 0.15 x 0.08	0.17 x 0.14 x 0.09
Radiation	Mo Kα (λ = 0.71073 Å)	Mo Kα (λ = 0.71073 Å)	Mo Kα (λ = 0.71073 Å)	Mo Kα (λ = 0.71073 Å)
h,k,l Ranges Collected	$-11 \le h \le 11$, $-14 \le k \le 14$, $-43 \le l \le 43$	$-19 \le h \le 20$, $-22 \le k \le 21$, $-33 \le l \le 30$	$-20 \le h \le 20$, $-39 \le k \le 39$, $-24 \le l \le 25$	$-30 \le h \le 30$, $-22 \le k \le 22$, $-39 \le l \le 39$
θ Range (deg)	1.743–26.352	1.594–34.723	1.612-36.386	1.390–30.587
No. of Reflections Collected	19894	23848	144408	28138
No. of Unique Reflections	12605	12699	15695	19345
No. of Parameters	772	697	487	1092
Data/Parameter Ratio	16.33	18.22	32.23	17.72
Refinement Method	Full-matrix least-squares of F^2	Full-matrix least-squares of F^2	Full-matrix least-squares of F^2	Full-matrix least-squares of F^2
$R(F)^a$	0.0580	0.0590	0.0357	0.0475
$R_w(F^2)^b$	0.1648	0.1159	0.0745	0.1074
GOF_w^c	0.983	0.867	1.012	1.062
Largest Diff Peak and Hole (e$^-$/Å^3)	0.809 and -0.418	0.621 and -0.584	0.854 and -0.913	3.104 and -1.732

[a] $R = [\Sigma |\Delta F| / \Sigma |F_a|]$ [b] $R_w = [\Sigma w(\Delta F)^2/\Sigma w F_a^2]$ [c] Goodness of fit on F^2

Table S2. Crystallographic Data for Pre2, MT$_d$2, MCBD2,3

	Pre2	MT$_d$2	MCBD2	MCBD3
Molecular Formula	$C_{22}H_{38}O_3W$	$C_{75}H_{72}MoO_3Si_3$	$C_{81}H_{78}O_3Si_3W$	$C_{52}H_{66}O_3Si_3W$
Fw	534.37	1201.53	1367.55	1007.16
Temp(K)	90.0(5)	90.0(5)	90.0(5)	90.0(5)
Crystal System	Triclinic	Trigonal	Triclinic	Monoclinic
Space Group	P-1	P-3	P-1	$P2_1$
Cell Constants				
a(Å)	9.6691(15)	21.8844(12)	12.6748(19)	11.0934(3)
b(Å)	11.4794(18)	21.8844(12)	14.118(2)	38.3571(10)
c(Å)	12.0496(19)	10.3174(4)	19.313(3)	11.4648(3)
α(deg)	106.175(2)	90	90.047 (8)	90
β(deg)	102.620(2)	90	99.423(8)	100.574(2)
γ(deg)	102.920(2)	120	101.919(8)	90
Z	2	2	2	4
V(Å^3)	1194.3(3)	4279.3(5)	3333.6(8)	4795.6(2)
Abs Coeff, μ_{calc} (mm^{-1})	4.852	4.864	1.836	2.525
δ_{calc} (g/cm^3)	1.486	1.262	1.362	1.395
F(000) (e$^-$/Å^3)	536	1680	1408	2072
Crystal Dimensions (mm)	0.279 x 0.243 x 0.067	0.160 x 0.060 x 0.050	0.397 x 0.267 x 0.156	0.19 x 0.18 x 0.10
Radiation	Mo Kα (λ = 0.71073 Å)	Cu Kα (λ = 1.54184 Å)	Mo Kα (λ = 0.71073 Å)	Mo Kα (λ = 0.71073 Å)
h,k,l Ranges Collected	-13 ≤ h ≤ 13, -15 ≤ k ≤ 15, -16 ≤ l ≤ 16	-24 ≤ h ≤ 24, -24 ≤ k ≤ 24, -11 ≤ l ≤ 11	-15 ≤ h ≤ 15, -17 ≤ k ≤ 17, -23 ≤ l ≤ 23	-16 ≤ h ≤ 16, -56 ≤ k ≤ 56, -14 ≤ l ≤ 16
θ Range (deg)	1.845–28.929	2.331 – 61.236	1.666 – 25.682	1.062-31.544
No. of Reflections Collected	6259	4389	11380	26810
No. of Unique Reflections	5982	4080	6717	25100
No. of Parameters	247	249	800	1071
Data/Parameter Ratio	24.22	17.63	8.40	23.44
Refinement Method	Full-matrix least-squares of F^2	Full-matrix least-squares of F^2	Full-matrix least-squares of F^2	Full-matrix least-squares of F^2
R(F)^a	0.0286	0.0948	0.0509	0.0669
$R_w(F^2)^b$	0.0752	0.22.93	0.0962	0.1512
GOF_w^c	1.182	1.151	0.882	1.187
Largest Diff Peak and Hole (e$^-$/Å^3)	4.292 and -2.795	1.487 and -0.880	1.798 and -1.704	5.494 and -6.780

^{a} R= [Σ |ΔF | / Σ |F_a |] ^{b} R_w= [Σw(ΔF)^2/ΣwF_a^2] ^{c} Goodness of fit on F^2

Table S3. Crystallographic Data for MCBD4-6 and Cat5●PhCN

	**MCBD4**	**MCBD5**	**MCBD6**	**Cat5●PhCN**
Molecular Formula	$C_{69}H_{60}O_3Si_3W \cdot (CH_2Cl_2)$	$C_{45}H_{60}O_3Si_3W$	$C_{80}H_{66}O_3Si_3W$	$C_{53}H_{61}NO_3Si_3W$
Fw	1529.83	917.05	1343.44	1028.14
Temp(K)	90.0(5)	90.0(5)	90.0(5)	90.0(5)
Crystal System	Triclinic	Monoclinic	Monoclinic	Triclinic
Space Group	P-1	$P2_1/c$	$P2_1/n$	P-1
Cell Constants				
a(Å)	12.9871(11)	9.7250(6)	13.5425(4)	10.7732(8)
b(Å)	14.2927(12)	26.0899(16)	19.4843(6)	11.2917(9)
c(Å)	18.5412(15)	17.2191(12)	24.2912(7)	20.8166(16)
α(deg)	91.487(4)	90	90	76.731(3)
β(deg)	95.290(4)	106.222(2)	104.3910(10)	76.456(3)
γ(deg)	95.748(4)	90	90	82.248(3)
Z	2	4	4	2
V(Å^3)	3407.6(5)	4195.0(5)	6208.5(3)	2387.3(3)
Abs Coeff, μ_{calc} (mm^{-1})	2.095	2.878	1.971	2.538
δ_{calc} (g/cm^3)	1.491	1.452	1.437	1.430
F(000) (e$^-$/Å^3)	1549	1880	2744	1052
Crystal Dimensions (mm)	0.342 x 0.174 x 0.166	0.124 x 0.113 x 0.051	0.102 x 0.070 x 0.020	0.180 x 0.160 x 0.070
Radiation	Mo Kα (λ = 0.71073 Å)	Mo Kα (λ = 0.71073 Å)	Mo Kα (λ = 0.71073 Å)	Mo Kα (λ = 0.71073 Å)
h,k,l Ranges Collected	-25 ≤ h ≤ 25, -28 ≤ k ≤ 28, -36 ≤ l ≤ 36	-13 ≤ h ≤ 14, -38 ≤ k ≤ 37, -24 ≤ l ≤ 25	-15 ≤ h ≤ 15, -20 ≤ k ≤ 22, -20 ≤ l ≤ 28	-20 ≤ h ≤ 20, -21 ≤ k ≤ 21, -39 ≤ l ≤ 39
θ Range (deg)	1.583–44.391	1.458– 31.587	1.357-24.778	1.860-42.497
No. of Reflections Collected	54100	13285	10635	33277
No. of Unique Reflections	38346	10695	7349	29979
No. of Parameters	715	478	819	559
Data/Parameter Ratio	53.63	22.37	8.97	59.5
Refinement Method	Full-matrix least-squares of F^2	Full-matrix least-squares of F^2	Full-matrix least-squares of F^2	Full-matrix least-squares of F^2
R(F)[a]	0.0429	0.0321	0.042	0.0219
$R_w(F^2)$[b]	0.0959	0.0711	0.0842	0.0443
GOF_w[c]	0.960	1.035	0.953	1.025
Largest Diff Peak and Hole (e$^-$/Å^3)	5.752 and -3.996	2.586 and -2.749	1.625 and -1.162	1.517 and -0.958

[a] $R = [\Sigma |\Delta F| / \Sigma |F_a|]$ [b] $R_w = [\Sigma w(\Delta F)^2/\Sigma w F_a^2]$ [c] Goodness of fit on F^2

Table S4. Crystallographic Data for Nitride1

	**Nitride1**
Molecular Formula	$C_{72}H_{90}N_2O_6Si_6W_2 \cdot (C_6H_6)$
Fw	1693.84
Temp(K)	90.0(5)
Crystal System	Monoclinic
Space Group	C_2/c
Cell Constants	
a(Å)	19.956(3)
b(Å)	12.6800(17)
c(Å)	29.462(4)
α(deg)	90
β(deg)	99.791(2)
γ(deg)	90
Z	4
V(Å^3)	7346.6(17)
Abs Coeff, μ_{calc} (mm^{-1})	3.280
δ_{calc} (g/cm^3)	1.531
F(000) (e$^-$/Å^3)	3431
Crystal Dimensions (mm)	0.119 x 0.083 x 0.078
Radiation	Mo Kα<br>(λ = 0.71073 Å)
h,k,l Ranges Collected	$-25 \leq h \leq 26$, $-16 \leq k \leq 16$, $-38 \leq l \leq 38$
θ Range (deg)	1.91-27.97
No. of Reflections Collected	58838
No. of Unique Reflections	8803
No. of Parameters	432
Data/Parameter Ratio	20.4
Refinement Method	Full-matrix<br>least-squares of F^2
R(F)[a]	0.0577
$R_w(F^2)$[b]	0.1010
GOF_w[c]	0.9685
Largest Diff Peak and Hole (e$^-$/Å^3)	6.6528 and -4.3272

[a] $R = [\Sigma |\Delta F| / \Sigma |F_a|]$ [b] $R_w = [\Sigma w(\Delta F)^2/\Sigma w F_a^2]$ [c] Goodness of fit on F^2

$C_6H_3(C_6H_4SiEt_2OH)_3$ ($\mathbf{SiP^{Et}}$)

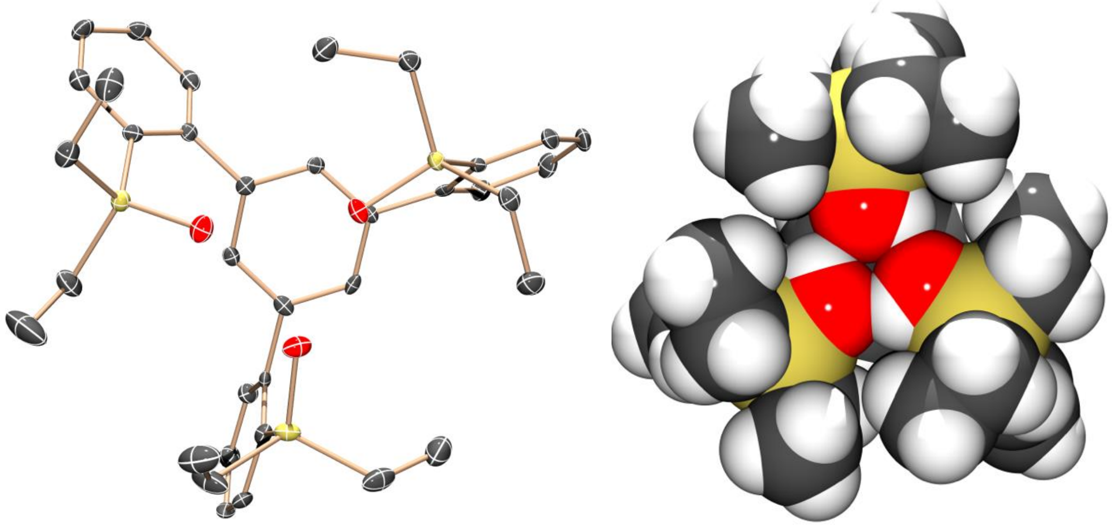

Figure S44. Crystal structure of $\mathbf{SiP^{Et}}$ (left) with the other independent molecule removed for clarity, ellipsoids for non-hydrogen atoms are shown at the 50% probability level. Space-filling model of $\mathbf{SiP^{Et}}$ (right).

$[C_6H_3(C_6H_4SiPh_2O)_3]W{\equiv}CMes$ (Cat3)

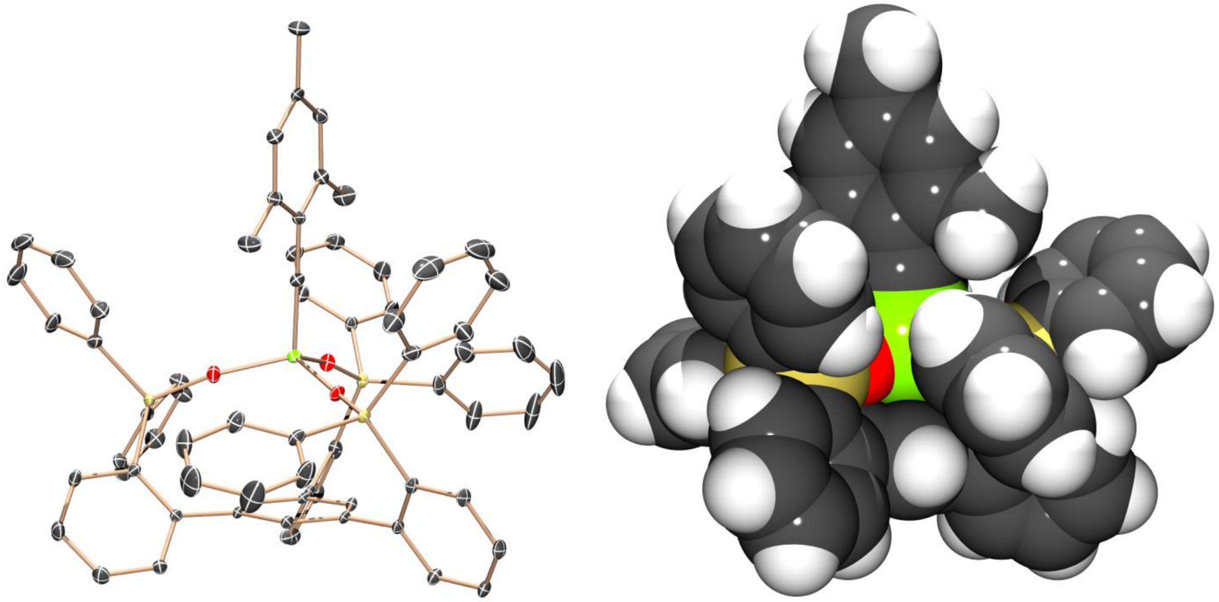

Figure S45. ORTEP of **Cat3** (left) with hydrogen atoms omitted for clarity, ellipsoids for the non-hydrogen atoms are shown at the 50% probability level. Space-filling model of **Cat3** (right).

[$C_6H_3(C_6H_4SiEt_2O)_3$]Mo≡CMes (Cat4)

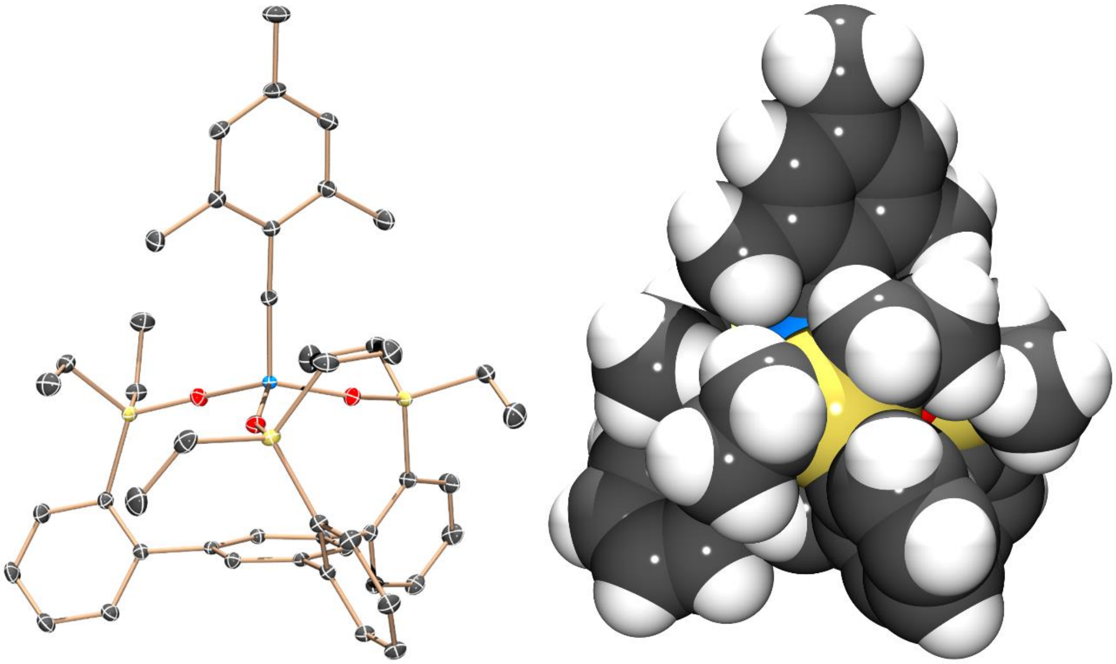

Figure S46. ORTEP of **Cat4** (left) with hydrogen atoms omitted for clarity, ellipsoids for the non-hydrogen atoms are shown at the 50% probability level. Space-filling model of **Cat4** (right).

[$C_6H_3(C_6H_4SiEt_2O)_3$]W≡CMes (Cat5)

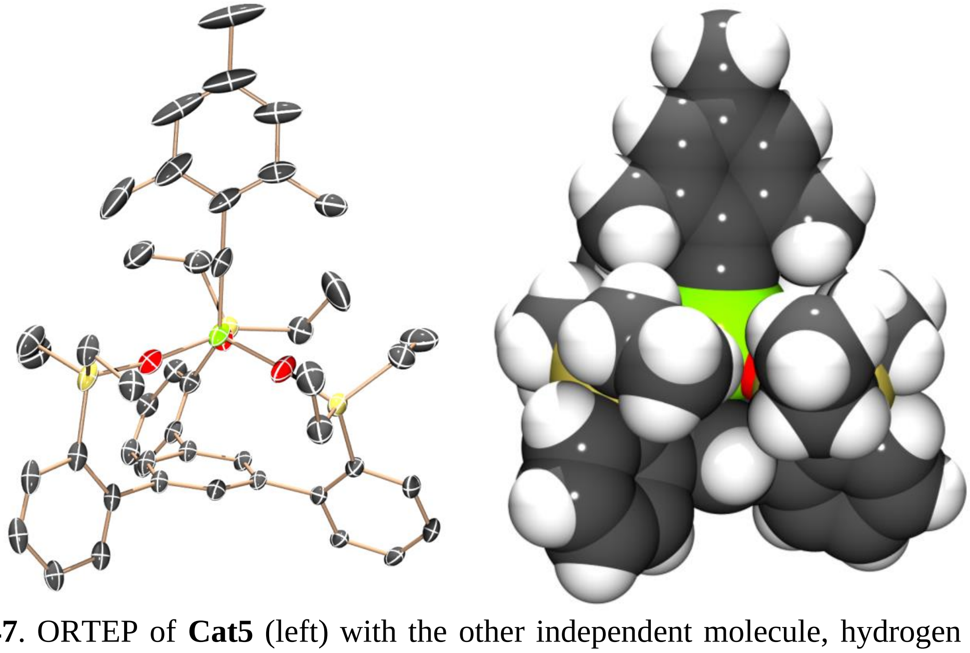

Figure S47. ORTEP of **Cat5** (left) with the other independent molecule, hydrogen atoms, and solvent omitted for clarity, ellipsoids for the non-hydrogen atoms are shown at the 50% probability level. Space-filling model of **Cat5** (right).

$(O^tBu)_3W\equiv CMes$ (Pre2)

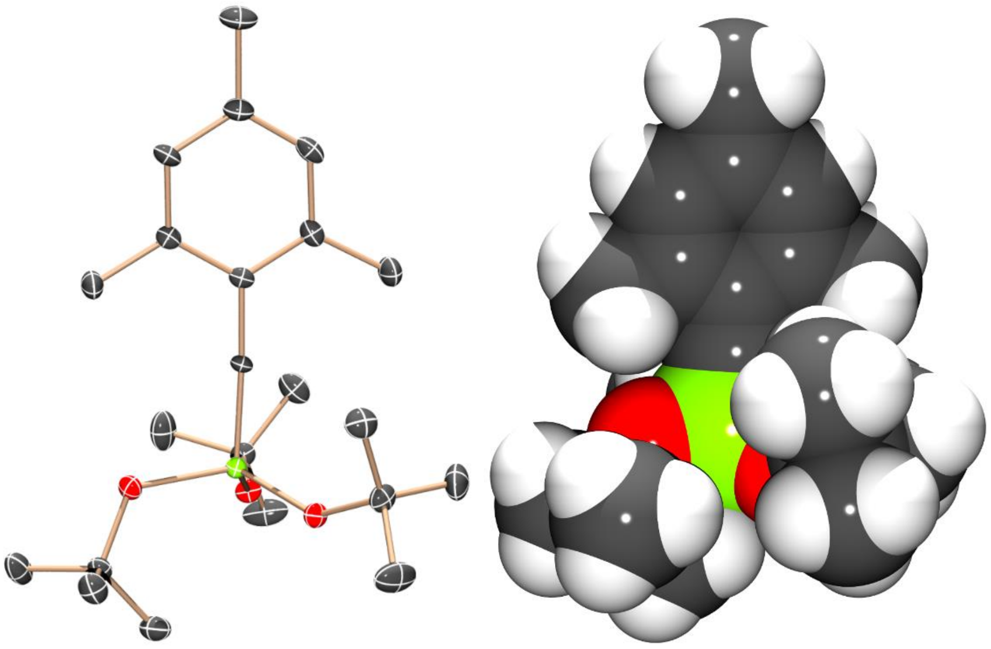

Figure S48. Crystal structure of **Pre2** (left) with hydrogen atoms removed for clarity, ellipsoids for non-hydrogen atoms are shown at the 50% probability level. Space-filling model of **Pre2** (right).

$[C_6H_3(C_6H_4SiPh_2O)_3]Mo(C_3Bu_3)$ (MT_d2)

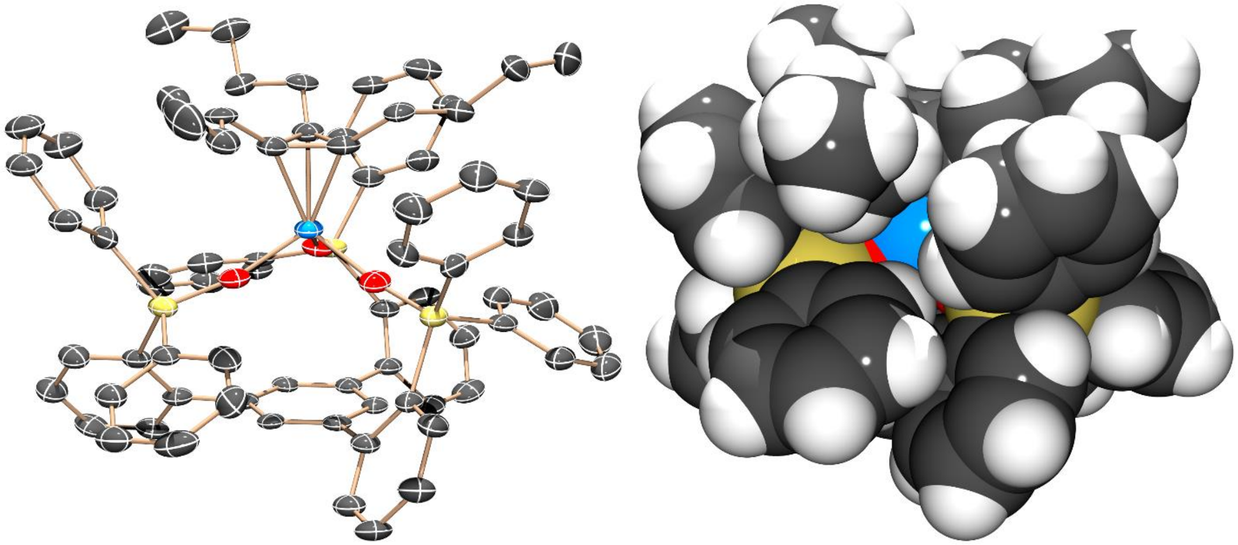

Figure S49. ORTEP of **MT_d2** (left) with hydrogen atoms omitted for clarity, ellipsoids for the non-hydrogen atoms are shown at the 50% probability level. Space-filling model of **MT_d2** (right).

$[C_6H_3(C_6H_4SiPh_2O)_3]W(C_3ArEt_2)$ (MCBD2)

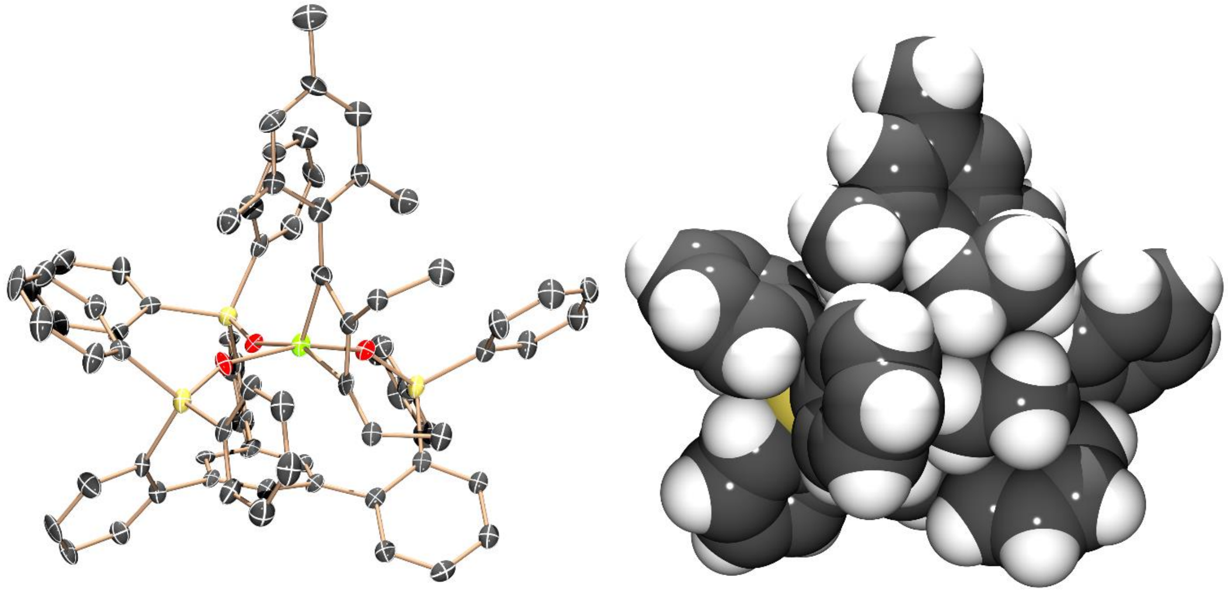

Figure S50. ORTEP of **MCBD2** (left) with hydrogen atoms and solvent omitted for clarity, ellipsoids for the non-hydrogen atoms are shown at the 50% probability level. Space-filling model of **MCBD2** (right).

$[C_6H_3(C_6H_4SiEt_2O)_3]W(C_3ArEt_2)$ (MCBD3)

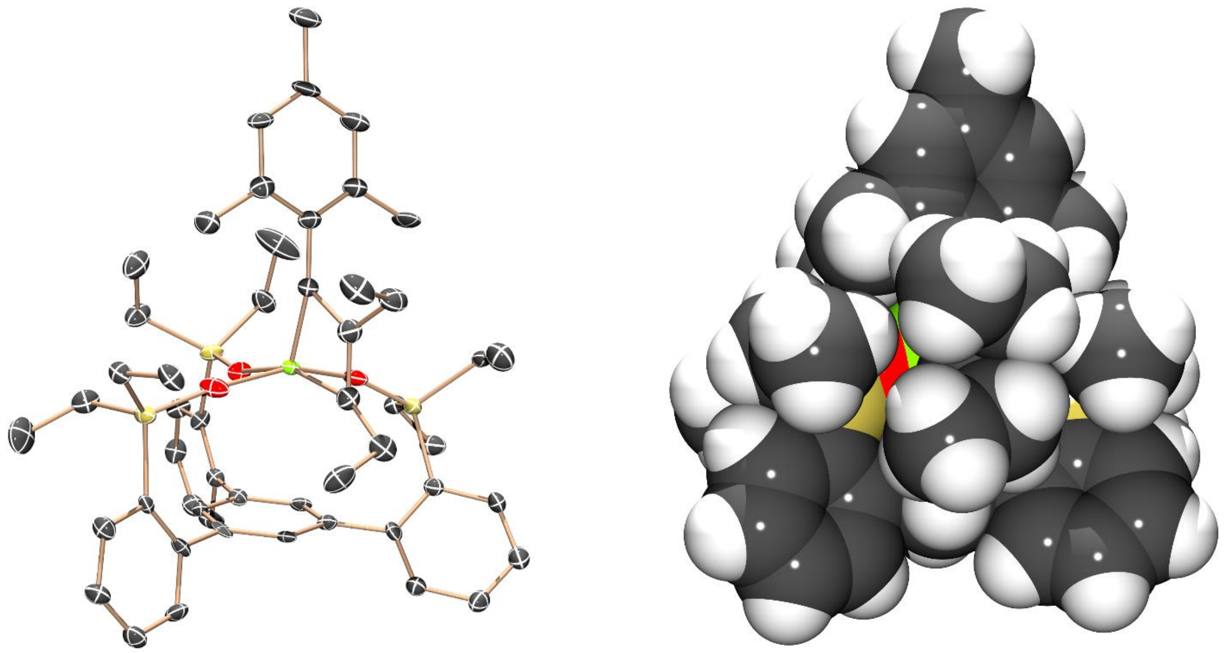

Figure S51. ORTEP of **MCBD3** (left) with hydrogen atoms, solvent and the other independent molecule omitted for clarity. Ellipsoids for the non-hydrogen atoms are shown at the 50% probability level. Space-filling model of **MCBD3** (right).

$[C_6H_3(C_6H_4SiPh_2O)_3]W(C_3Et_3)$ (MCBD4)

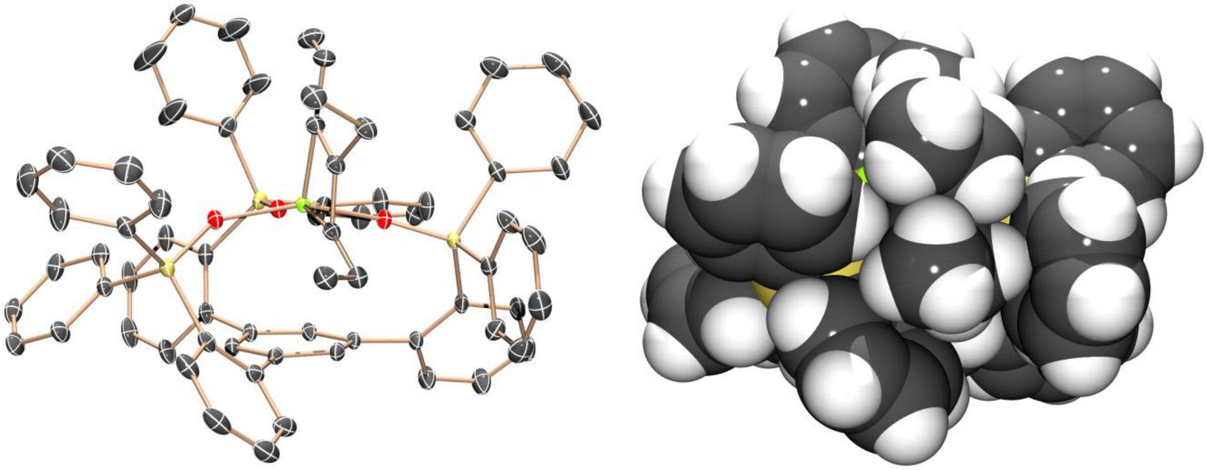

Figure S52. ORTEP of **MCBD4** (left) with hydrogen atoms and solvent omitted for clarity, ellipsoids for the non-hydrogen atoms are shown at the 50% probability level. Space-filling model of **MCBD4** (right).

$[C_6H_3(C_6H_4SiEt_2O)_3]W(C_3Et_3)$ (MCBD5)

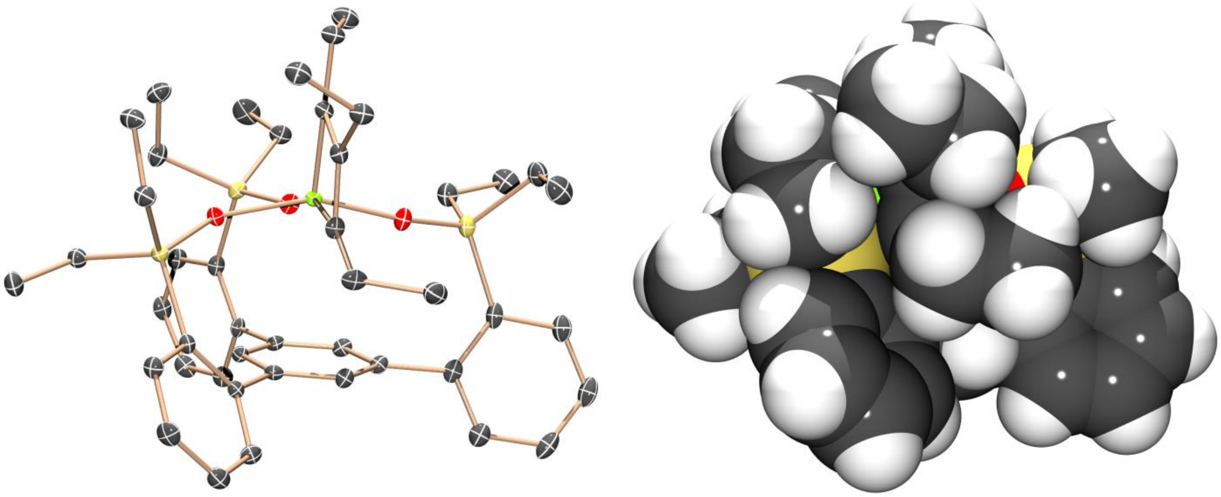

Figure S53. ORTEP of **MCBD5** (left) with hydrogen atoms omitted for clarity, ellipsoids for the non-hydrogen atoms are shown at the 50% probability level. Space-filling model of **MCBD5** (right).

$[C_6H_3(C_6H_4SiEt_2O)_3]W(C_3ArAr'Me)$ (MCBD6)

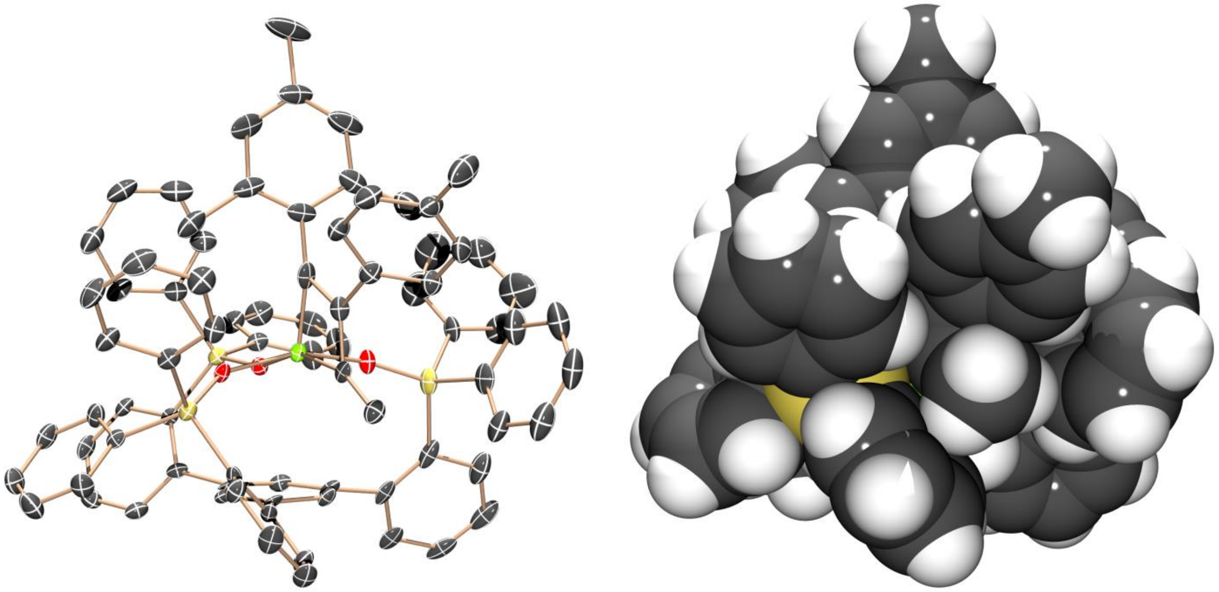

Figure S54. ORTEP of **MCBD6** (left) with hydrogen atoms omitted for clarity, ellipsoids for the non-hydrogen atoms are shown at the 50% probability level. Space-filling model of **MCBD6** (right).

$[C_6H_3(C_6H_4SiEt_2O)_3]W{\equiv}CMes$●PhCN (Cat5●PhCN)

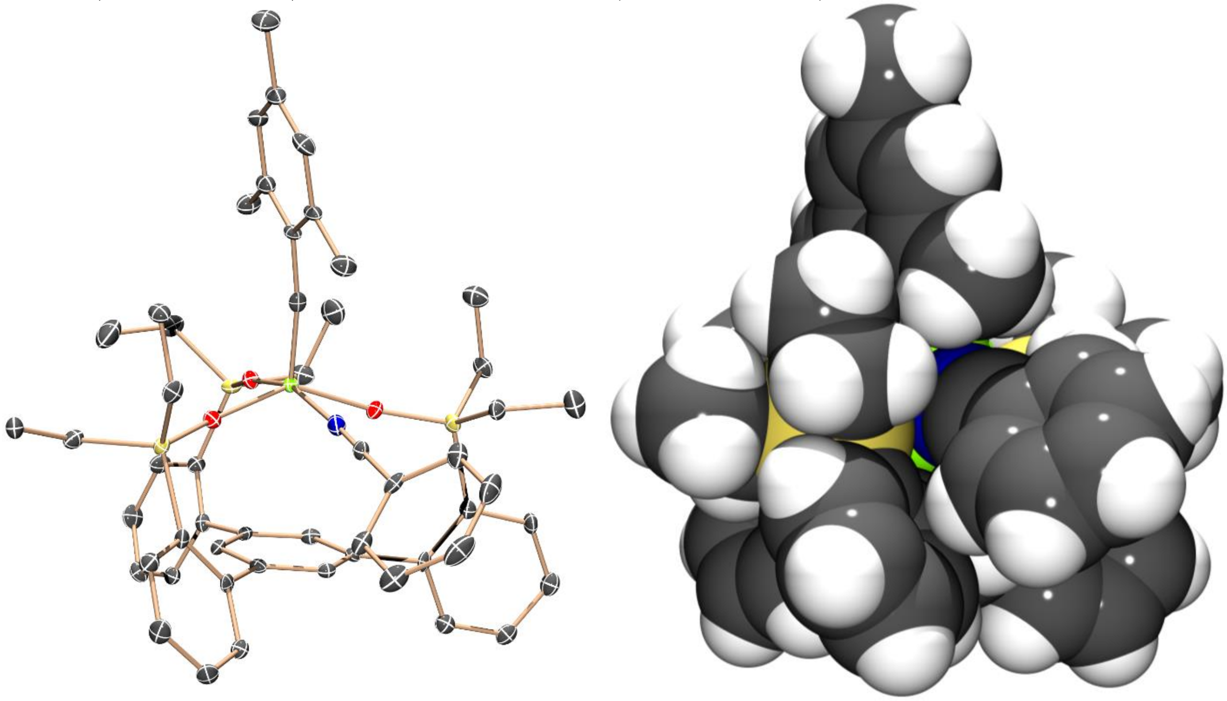

Figure S55. ORTEP of **Cat5●PhCN** (left) with hydrogen atoms omitted for clarity, ellipsoids for the non-hydrogen atoms are shown at the 50% probability level. Space-filling model of **Cat5●PhCN** (right).

([$C_6H_3(C_6H_4SiEt_2O)_3$]W≡N)$_2$ (Nitride1)

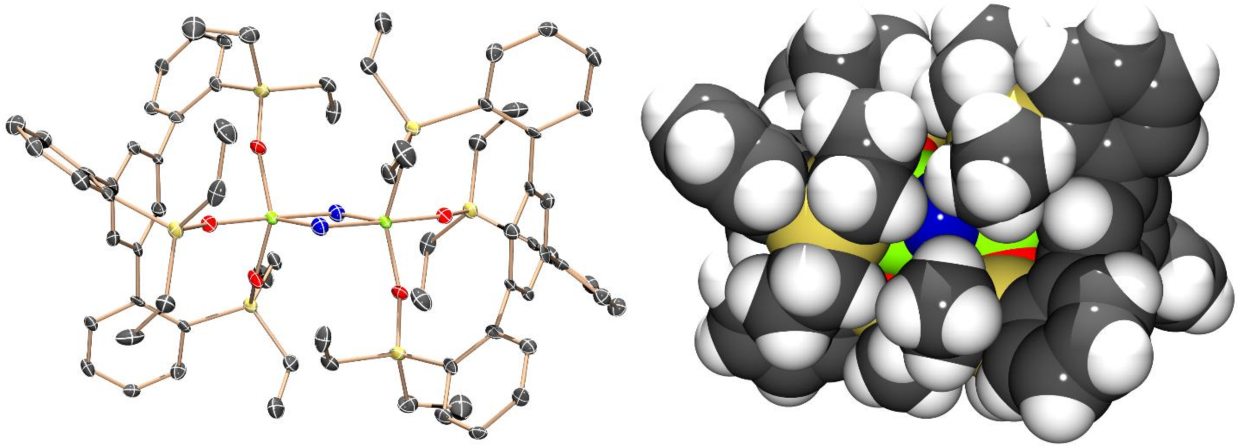

Figure S56. ORTEP of **Nitride1** (left) with hydrogen atoms and solvent omitted for clarity, ellipsoids for the non-hydrogen atoms are shown at the 50% probability level. Space-filling model of **Nitride1**(right).

Computational details

General Remarks

All optimizations of intermediates and transition states were calculated using restricted B3LYP-D3/def2SVP-LANL2DZ(M) level of the theory[10] in implicit solvent (benzene) using CPCM as solvation model[11] as implemented in Gaussian09. Frequency calculations, at the same level of theory, were used to obtain thermal corrections (at 298K) and to characterize optimized structures as transition states (only a single imaginary frequency) or intermediate (if no imaginary frequencies were found). Intrinsic reaction coordinate (IRCs) calculations were undertaken to ensure transition states connected illustrated ground states. Single point energy calculations using B3LYP-D3/def2TZVP-SDD(M) with solvent corrections calculated in implicit solvent (benzene) using CPCM were also performed on all structures.[12] For comparison, single point energy calculations with restricted PBEPBE/def2TZVP-SDD(M) were calculated with solvent corrections calculated in implicit solvent (benzene) using CPCM as a solvation model. All reported charges are from Mulliken population analysis.[13] All 3-D structures were generated using CYLview.[14] Noncovalent interaction (NCI) analysis, also known as reduce density gradient (RDG) method, was performed on Multiwfn to study the possible effect of noncovalent interaction in the metallatetrahedrane intermediates.[15] Extension distance of 0 Bohr, medium quality grid (totally about 512000 points) were set by default. Further visualization of the color-filled RDG isosurface was realized by VMD, where RDG isosurface and color range were set as 0.5, and -0.035 to 0.2, respectively.[16] The energy decomposition analysis calculations were performed using the second-generation absolutely localized molecular orbitals[17] (ALMO-EDA) method implemented in Q-Chem 5.0.[18] The HF/6-311G(d,p) method was used as employed by Liu.[19] This method decomposes the through-space interaction energies between the ligand and substrate into the energetic components including the Pauli repulsion energy (ΔE_{Pauli}), the electrostatic energy (ΔE_{elstat}), the polarization energy (ΔE_{pol}) and the charge transfer energy (ΔE_{ct}.) Distortion energies (ΔE_{dist}), or the energy required to distort the geometry of the starting intermediate to the transition

state geometry as described by the distortion-interaction model,[20] were calculated using the B3LYP-D3/defTZVP-SDD(M)-CPCM(benzene)//B3LYP-D3/def2SVP-LANL2DZ(M)-CPCM(benzene)level of theory in Gaussian 09.

Full Reference of Gaussian 09 Software

Gaussian 09, Revision E.01, M. J. Frisch, G. W. Trucks, H. B. Schlegel, G. E. Scuseria, M. A. Robb, J. R. Cheeseman, G. Scalmani, V. Barone, B. Mennucci, G. A. Petersson, H. Nakatsuji, M. Caricato, X. Li, H. P. Hratchian, A. F. Izmaylov, J. Bloino, G. Zheng, J. L. Sonnenberg, M. Hada, M. Ehara, K. Toyota, R. Fukuda, J. Hasegawa, M. Ishida, T. Nakajima, Y. Honda, O. Kitao, H. Nakai, T. Vreven, J. A. Montgomery, Jr., J. E. Peralta, F. Ogliaro, M. Bearpark, J. J. Heyd, E. Brothers, K. N. Kudin, V. N. Staroverov, R. Kobayashi, J. Normand, K. Raghavachari, A. Rendell, J. C. Burant, S. S. Iyengar, J. Tomasi, M. Cossi, N. Rega, J. M. Millam, M. Klene, J. E. Knox, J. B. Cross, V. Bakken, C. Adamo, J. Jaramillo, R. Gomperts, R. E. Stratmann, O. Yazyev, A. J. Austin, R. Cammi, C. Pomelli, J. W. Ochterski, R. L. Martin, K. Morokuma, V. G. Zakrzewski, G. A. Voth, P. Salvador, J. J. Dannenberg, S. Dapprich, A. D. Daniels, Ö. Farkas, J. B. Foresman, J. V. Ortiz, J. Cioslowski, and D. J. Fox, Gaussian, Inc., Wallingford CT, 2009.

Choice of Computational Method

The computational method of B3LYP-D3/def2TZVP-SDD(M)-CPCM(benzene)//B3LYP-D3/def2SVP-LANL2DZ(M)-CPCM(benzene) was chosen as it provides an excellent balance between accuracy and computational cost, all while reproducing structural features from x-ray crystal structures. Pseudopotentials such as LANL2DZ and SDD are very commonly used on the metal center to reduce computational cost while maintaining the accuracy of the calculation.[21] Furthermore, pseudopotentials such as used LANL2DZ and SDD have been shown to be effective in describing Mo and W compounds.[22]

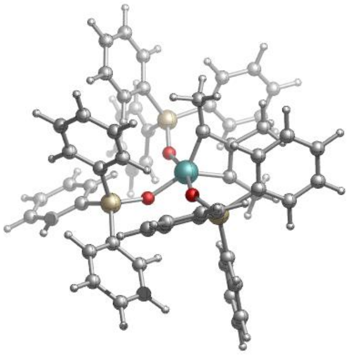

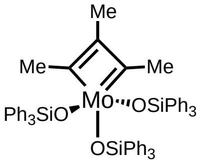

MCBD1-comp

	MCBD1-comp
**Mo1-C1**	1.920
**Mo-O$_{Avg}$**	1.991
**O-Si$_{Avg}$**	1.653
**C1-C2**	1.426
**C2-C3**	1.466
**C3-Mo1**	1.889
**Mo1-C1-C2**	79.18
**C1-C2-C3**	119.51
**C2-C3-Mo1**	79.32
**C3-Mo1-C1**	81.98
**Mo-O-Si$_{Avg}$**	143.71

Table S5. List of Bond lengths and angles for **MCBD1-comp**, optimized using B3LYP-D3/def2SVP-LANL2DZ(Mo)-CPCM(benzene).

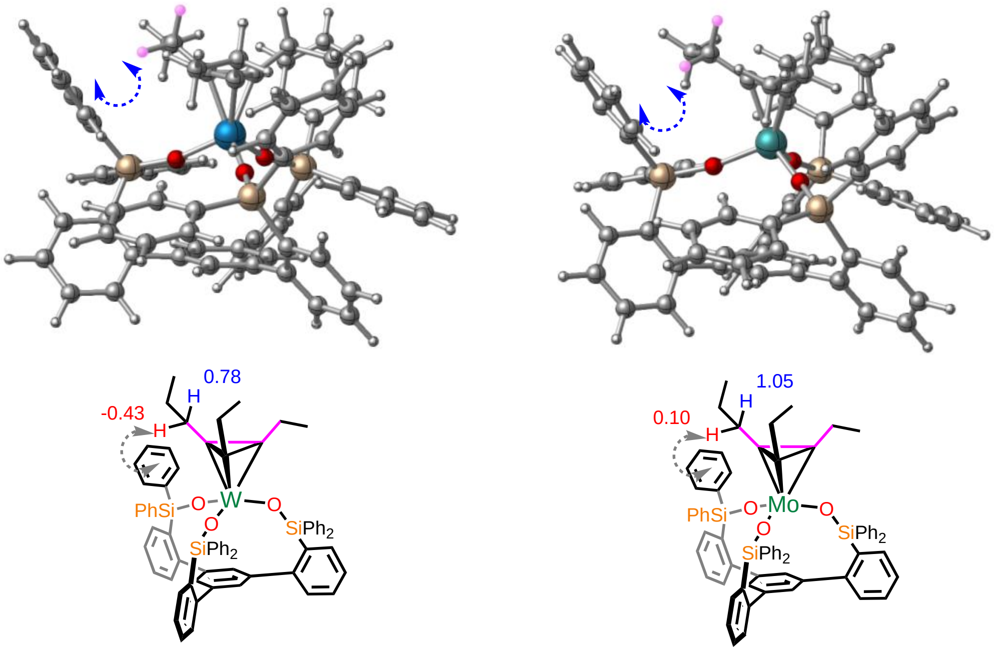

Figure S57. Calculated ^{1}H (ppm) chemical shift for tungsten with SiP^{Ph6} system and molybdenum with SiP^{Ph6} system computed at the B3LYP-D3/def2TZVP-SDD(M)-

CPCM(benzene)// B3LYP-D3/def2SVP-LANL2DZ(M)-CPCM(benzene)level of theory. The calculated chemical shifts of the shown hydrogen atoms on the ethyl groups provide evidence for the CH•••π interaction between the ethyl side chains and the aryl groups on the ligand.

Substrate-Dependence on Reaction Rates

For comparison of the experimental results of the apparent substrate-dependence on reaction rates, we have computed the lowest energy pathway to product formation for an aryl-propyne substrate with the less sterically hindered SiMe ligand for both tungsten and molybdenum. These pathways are given in Figures S58 and S59 below. While the barrier to [2+2]-cycloaddition is lower in energy for tungsten compared to molybdenum (9.2 vs. 16.6 kcal/mol), it is clear from our computations that the tungsten system would be slower due to the thermodynamically stabilized MCBD intermediate [W]-ent-C' (-0.3 kcal/mol). From here, the MCBD will undergo retro-[2+2] to yield product, with a relative energy barrier of 12.6 kcal/mol for tungsten and only 7.0 kcal/mol for molybdenum. These computations suggest that the reaction rates are dependent on the substrate for tungsten.

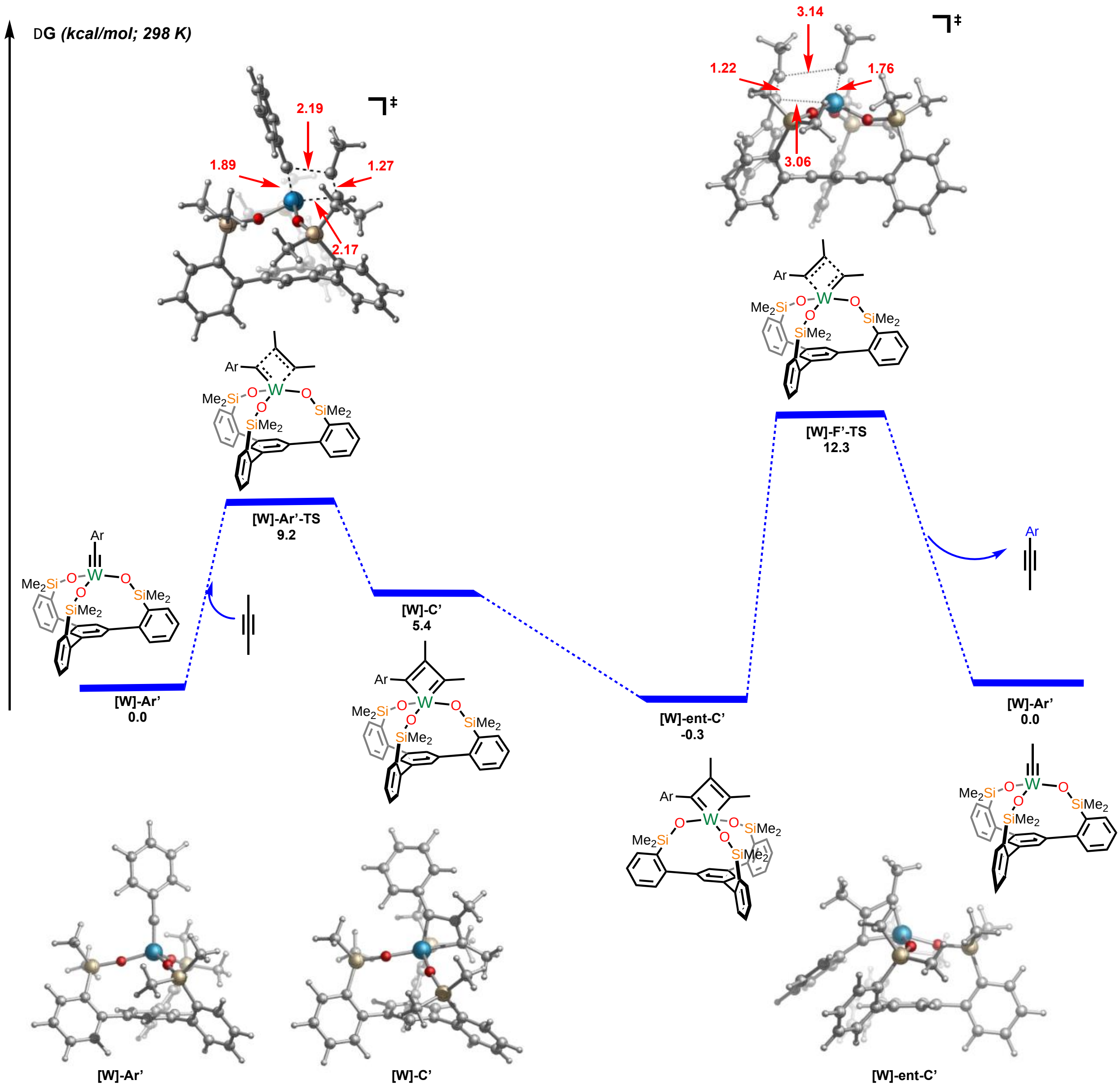

Figure S58. Energetics of MCBD formation via [2+2] cycloaddition for tungsten with **SiP^{Me}** ligand showing the cross-metathesis of aryl-propyne substrates. Free energies (kcal/mol) are computed at the B3LYP-D3/def2TZVP-SDD(W)-CPCM(benzene)// B3LYP-D3/def2SVP-LANL2DZ(W)-CPCM(benzene)level of theory.

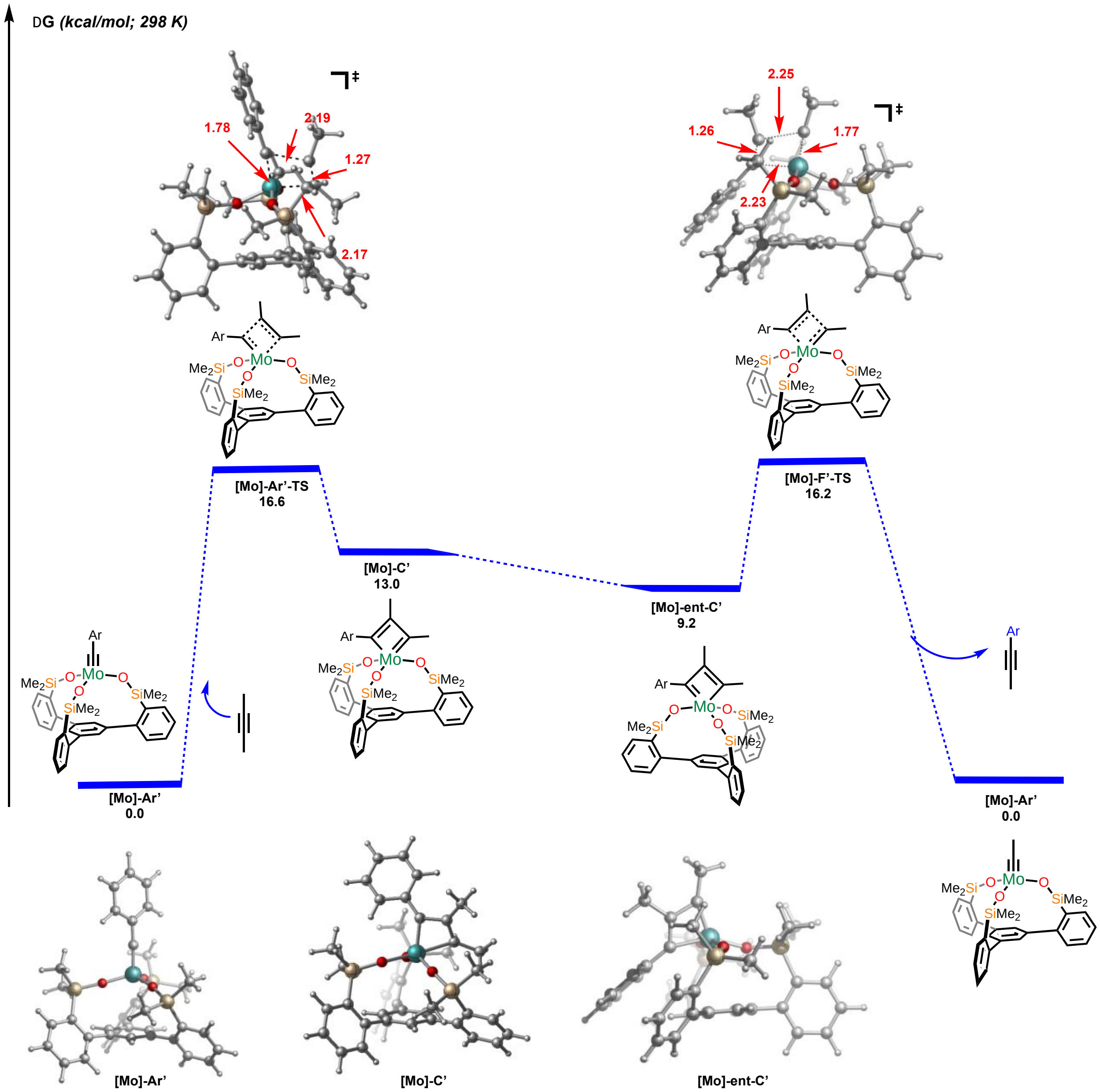

Figure S59. Energetics of MCBD formation via [2+2] cycloaddition for molybdenum with **SiP^{Me}** ligand showing the cross-metathesis of aryl-propyne substrates. Free energies (kcal/mol) are computed at the B3LYP-D3/def2TZVP-SDD(Mo)-CPCM(benzene)// B3LYP-D3/def2SVP-LANL2DZ(Mo)-CPCM(benzene)level of theory.

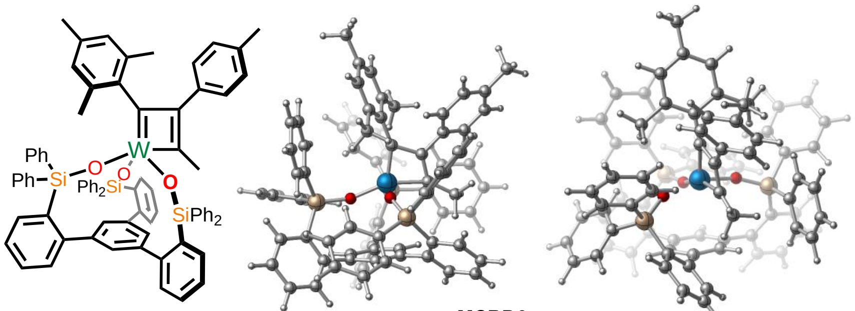

MCBD6-comp

	MCBD6-comp	MCBD6
**W1-C1**	1.866	1.870
**C1-C2**	1.533	1.504
**C2-C3**	1.397	1.417
**C3-W1**	2.009	1.966
**W1-C1-C2**	78.26	77.09
**C1-C2-C3**	119.17	121.29
**C2-C3-W1**	80.08	78.40
**C3-W1-C1**	81.71	83.16

Table S6. List of Bond lengths and angles for **MCBD6-comp**, optimized using B3LYP-D3/def2SVP-LANL2DZ(M)-CPCM(benzene) compared to those of the x-ray crystal structure of **MCBD6**. Similar to that observed in **MCBD6**, the tolyl group is pointing away from the basal arene to minimize steric crowding.

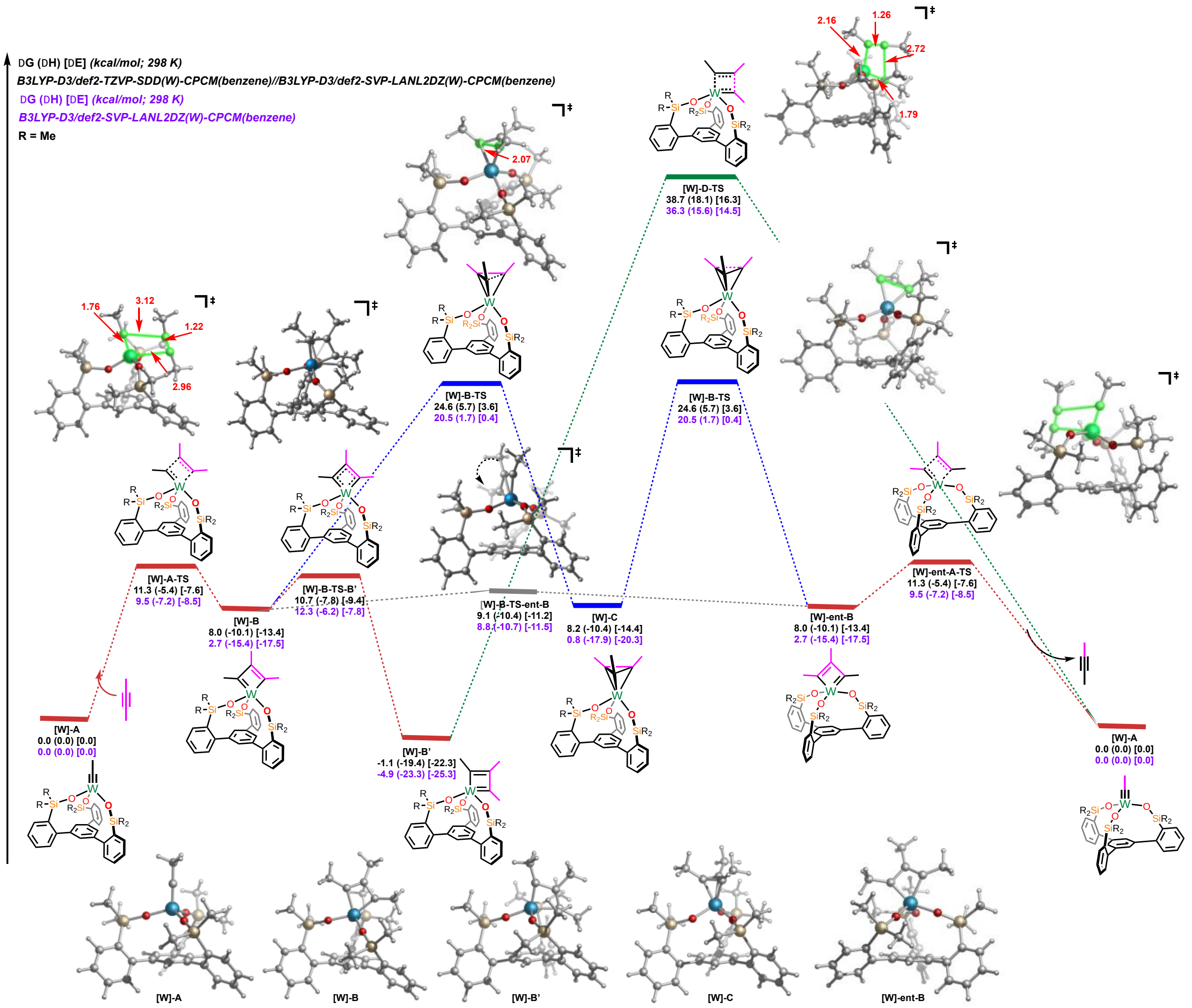

Figure S60. Energetics of MCBD and MTd formation via [2+2] cycloaddition for tungsten with SiP^{Me} system computed at the B3LYP-D3/def2TZVP-SDD(W)-CPCM(benzene)// B3LYP-D3/def2SVP-LANL2DZ(W)-CPCM(benzene)level of theory (black) and the PBEPBE/def2TZVP-SDD(W)-CPCM(benzene)// B3LYP-D3/def2SVP-LANL2DZ(W)-CPCM(benzene)level of theory (purple).

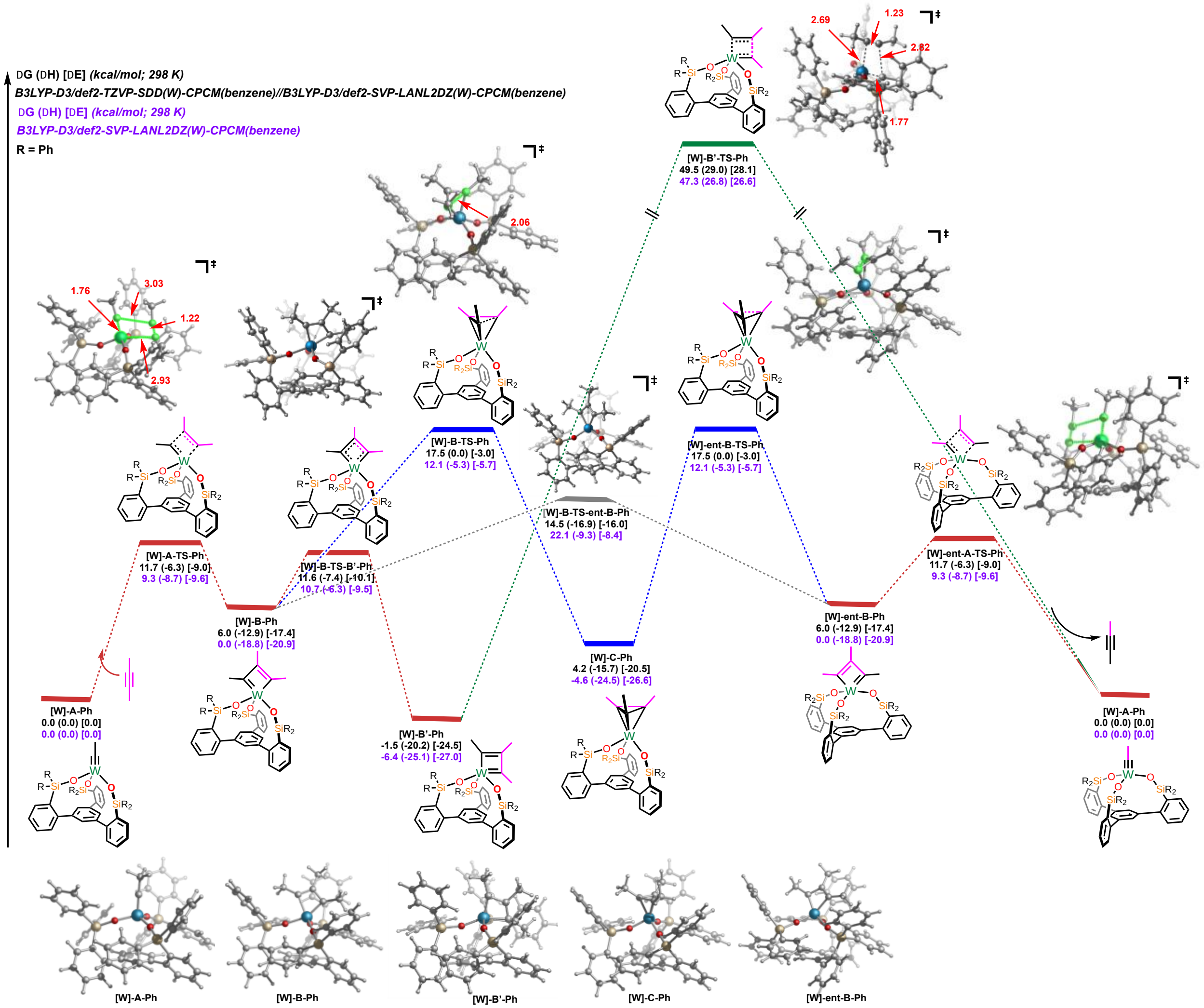

Figure S61. Energetics of MCBD and MTd formation via [2+2] cycloaddition for tungsten with SiP^{Ph} system computed at the B3LYP-D3/def2TZVP-SDD(W)-CPCM(benzene)// B3LYP-D3/def2SVP-LANL2DZ(W)-CPCM(benzene)level of theory (outside parenthesis) and the PBEPBE/def2TZVP-SDD(W)-CPCM(benzene)// B3LYP-D3/def2SVP-LANL2DZ(W)-CPCM(benzene)level of theory (inside parenthesis).

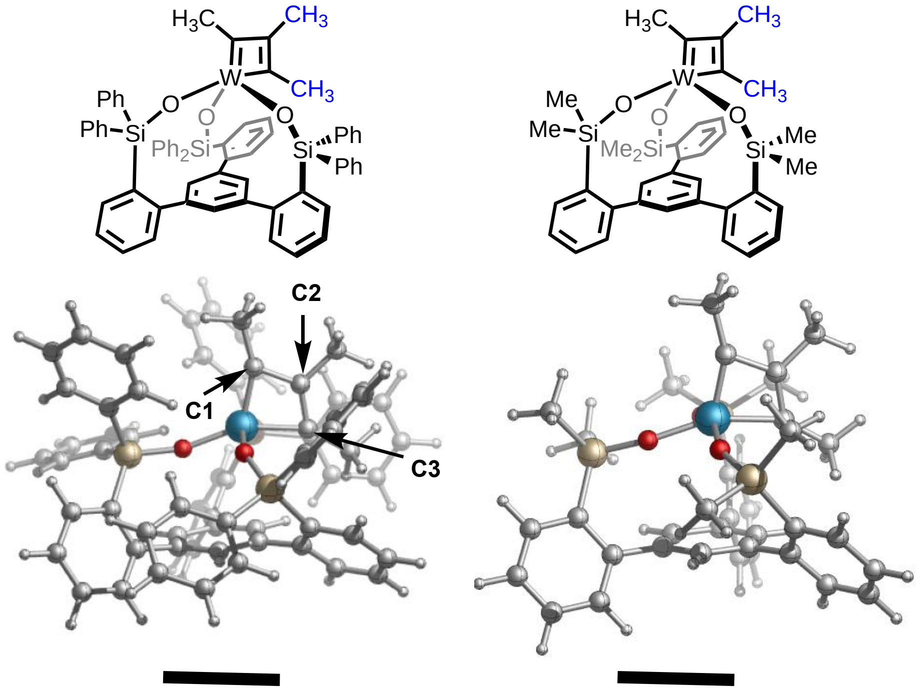

[W]-B’-Ph
-1.5

[W]-B’-Me
-1.1

	[W]-B’-Ph	MCBD4	[W]-B’-Me	MCBD5
**W1-C1**	1.868	1.862	1.862	1.865
**C1-C2**	1.511	1.493	1.525	1.487
**C2-C3**	1.401	1.412	1.387	1.415
**C3-W1**	1.994	1.982	2.010	1.967
**W1-C1-C2**	79.74	78.24	80.11	78.89
**C1-C2-C3**	120.58	122.3	120.37	121.41
**C2-C3-W1**	77.88	76.11	78.12	77.04
**C3-W1-C1**	81.80	83.28	81.40	82.63

Table S7. List of Bond Lengths (Å) and Angles (°) for computed metallacyclobutadienes **[W]-B’-Ph** and **[W]-B’-Me** compared to the analogous isolated structures **MCBD4** and **MCBD5**.

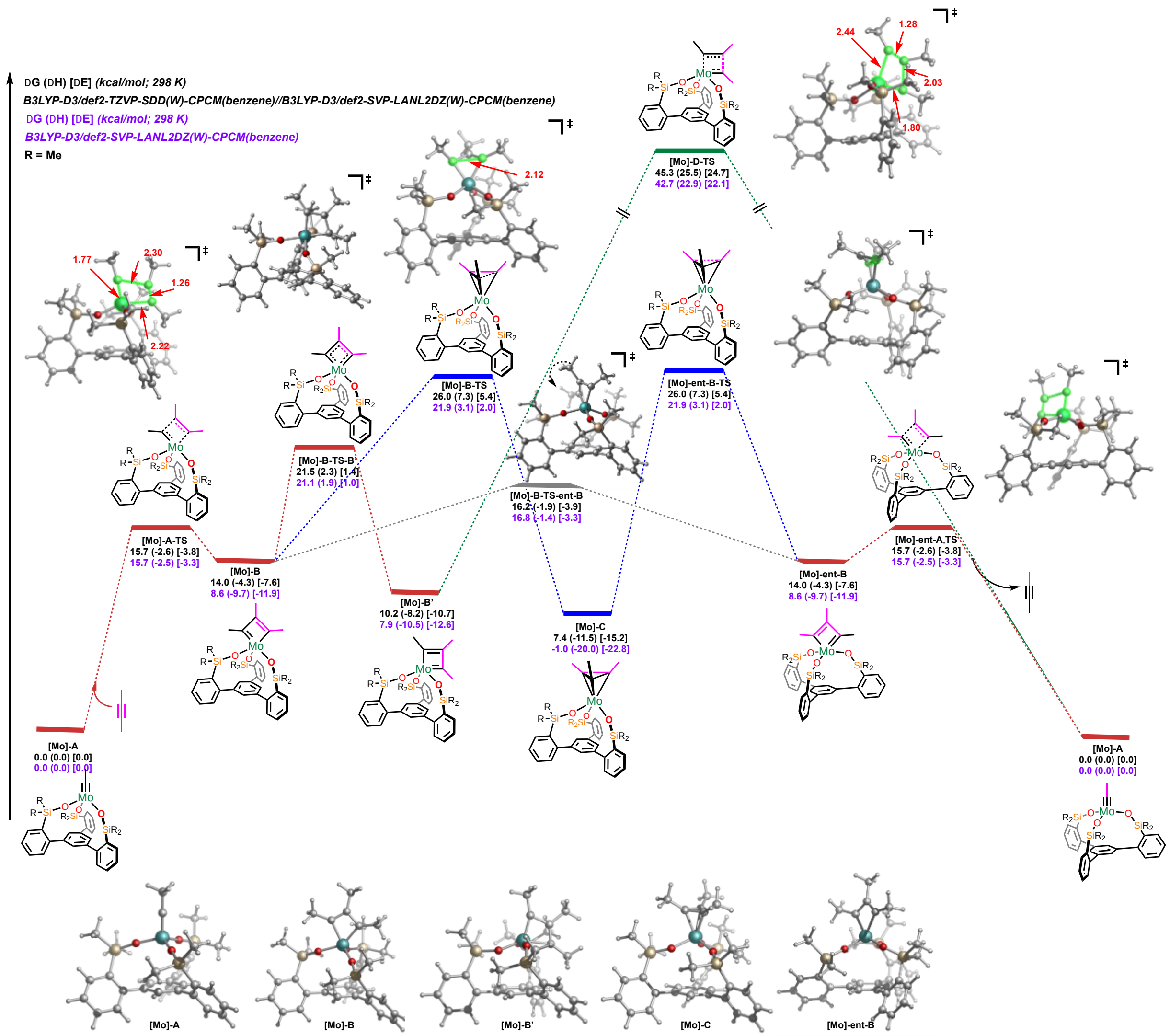

Figure S62. Energetics of MCBD and MTd formation via [2+2] cycloaddition for molybdenum with SiP^{Me} system computed at the B3LYP-D3/def2TZVP-SDD(Mo)-CPCM(benzene)// B3LYP-D3/def2SVP-LANL2DZ(Mo)-CPCM(benzene)level of theory (outside parenthesis) and the PBEPBE/def2TZVP-SDD(Mo)-CPCM(benzene)// B3LYP-D3/def2SVP-LANL2DZ(Mo)-CPCM(benzene)level of theory (inside parenthesis).

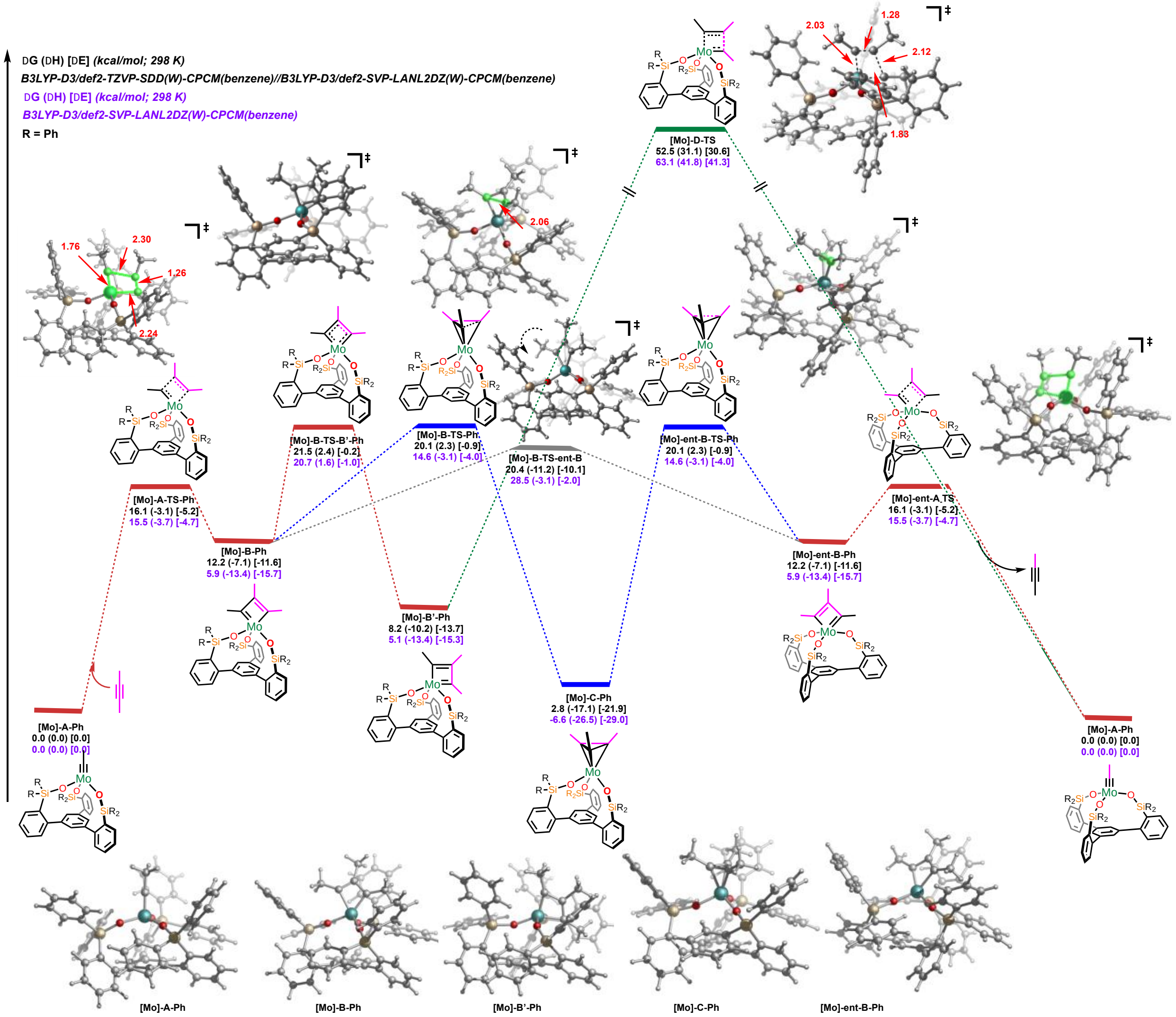

Figure S63. Energetics of MCBD and MTd formation via [2+2] cycloaddition for molybdenum with SiP^{Ph} system computed at the B3LYP-D3/def2TZVP-SDD(Mo)-CPCM(benzene)// B3LYP-D3/def2SVP-LANL2DZ(Mo)-CPCM(benzene)level of theory (outside parenthesis) and the PBEPBE/def2TZVP-SDD(Mo)-CPCM(benzene)// B3LYP-D3/def2SVP-LANL2DZ(Mo)-CPCM(benzene)level of theory (inside parenthesis).

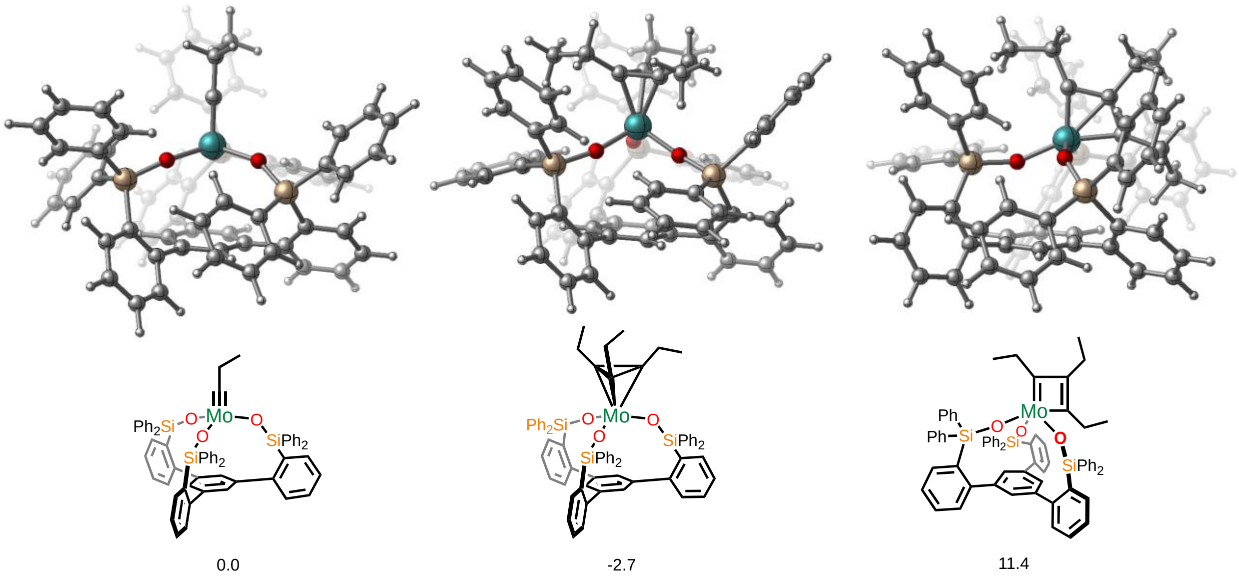

Figure S64. Energetics of MCBD and MT_d formation for molybdenum with **SiP**$^{\mathbf{Ph}}$ ligand and 3-hexyne substrate. Free energies (kcal/mol) are computed at the B3LYP-D3/def2TZVP-SDD(Mo)-CPCM(benzene)// B3LYP-D3/def2SVP-LANL2DZ(Mo)-CPCM(benzene)level of theory.

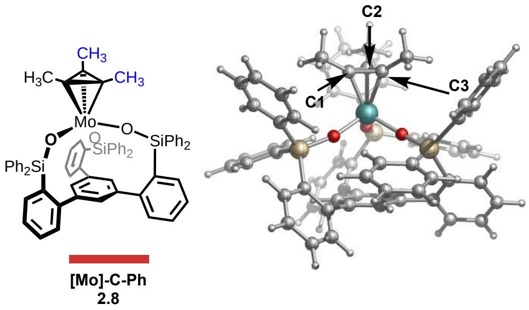

	[Mo]-C-Ph	MTd1
**Mo-C1**	2.093	2.064
**Mo-C2**	2.094	2.065
**Mo-C3**	2.094	2.064
**C1-C2**	1.453	1.445
**C2-C3**	1.454	1.458
**C3-C1**	1.451	1.458
**Mo-C1-C2**	69.65	69.2
**C1-C2-C3**	59.89	60.3
**C2-C3-Mo**	69.64	69.0
**C3-Mo-C1**	40.54	41.4

Table S8. List of Bond Lengths (Å) and Angles (°) for computed metallatetrahedrane **[Mo]-C-Ph** compared to the analogous isolated structure **MTd1.**

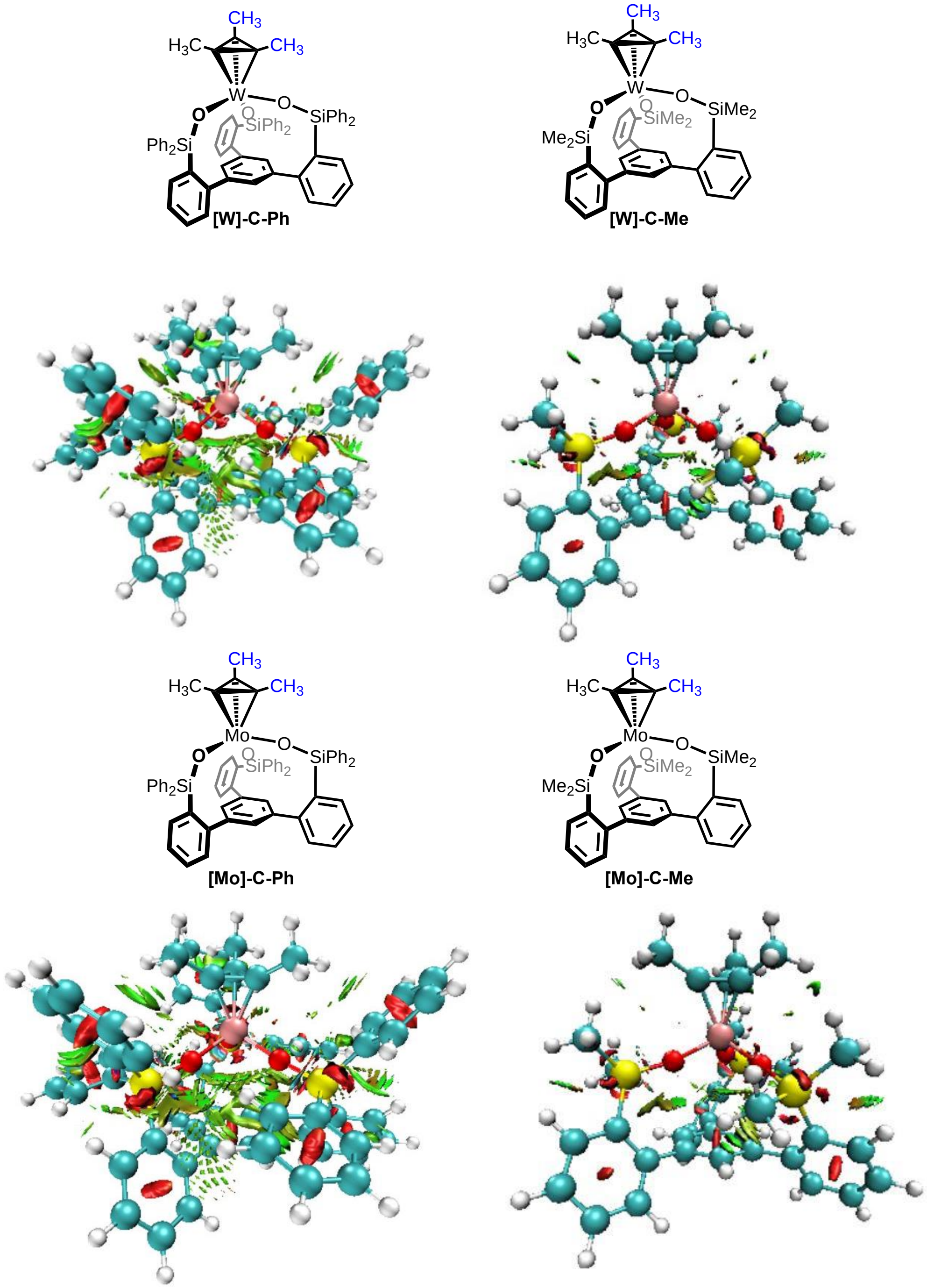

Figure S65. NCI plots showing the comparison between metallatetrahedranes in the tungsten (top) and molybdenum (bottom) systems with both the SiP^{Ph} and SiP^{Me} ligands. Key C-H···π noncovalent stabilizing interactions are visible in the NCI plots of the SiP^{Ph} metallatetrahedranes, as indicated by the green colors between substrate C-H and ligand aryl groups.

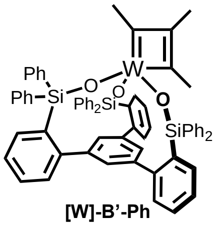

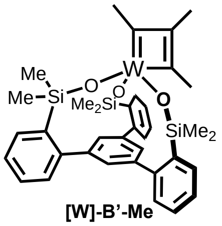

[W]-B’-Ph **[W]-B’-Me**

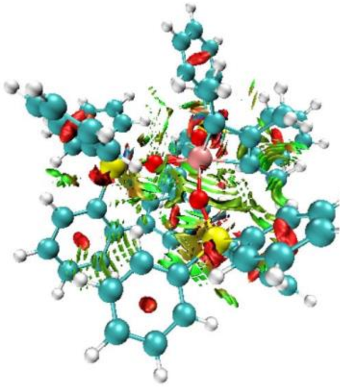

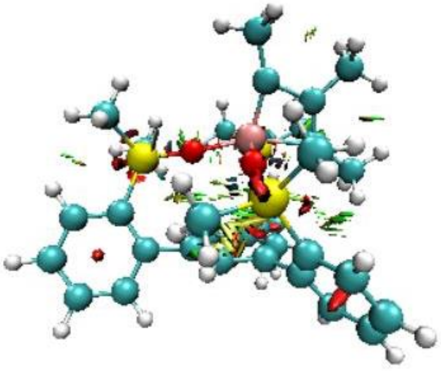

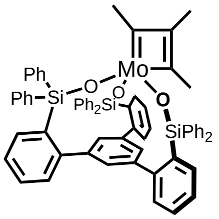

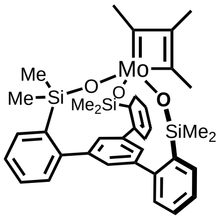

[Mo]-B’-Ph **[Mo]-B’-Me**

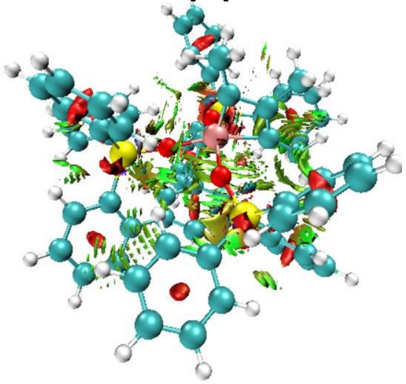

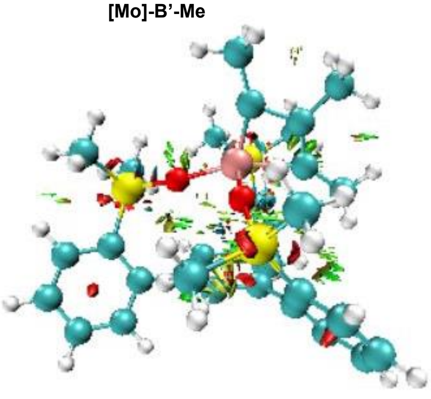

Figure S66. NCI plots showing the comparison between metallacyclobutadienes in the tungsten (top) and molybdenum (bottom) systems with both the SiP^{Ph} and SiP^{Me} ligands. Key C-H···π noncovalent stabilizing interactions visible in the NCI plots of the SiP^{Ph} metallatetrahedranes are no longer present in the MCBD structures.

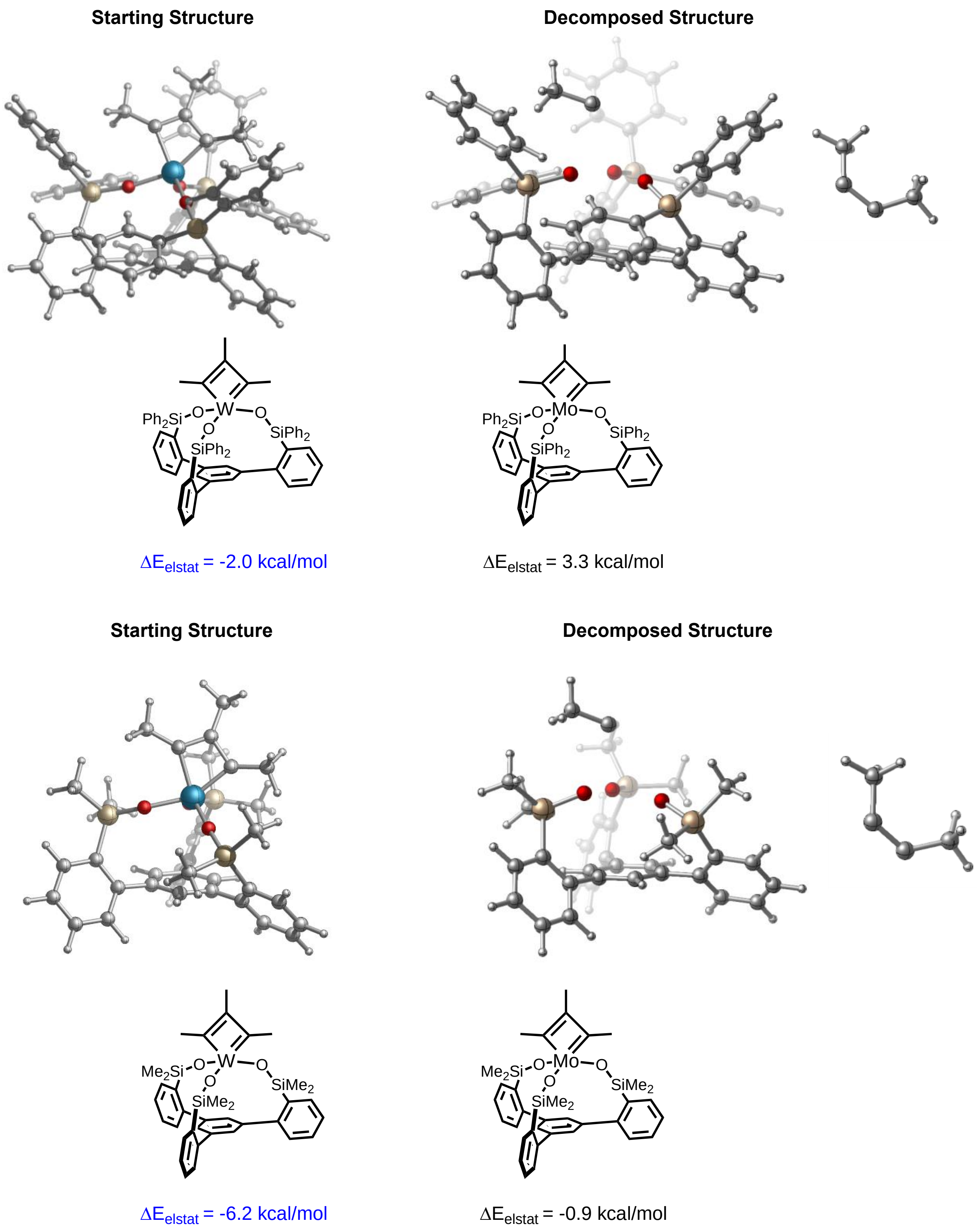

Figure S67. Electrostatic energies for the MCBD intermediates, [W]-B and [Mo]-B, computed by the second-generation ALMO-EDA method using HF/6-311G(d,p) with the shown decomposed intermediates from the given starting structure. The electrostatic energies are lower for [W]-B than [Mo]-B and thus the electrostatic energy likely contributes to tungsten's preference to form the MCBD.

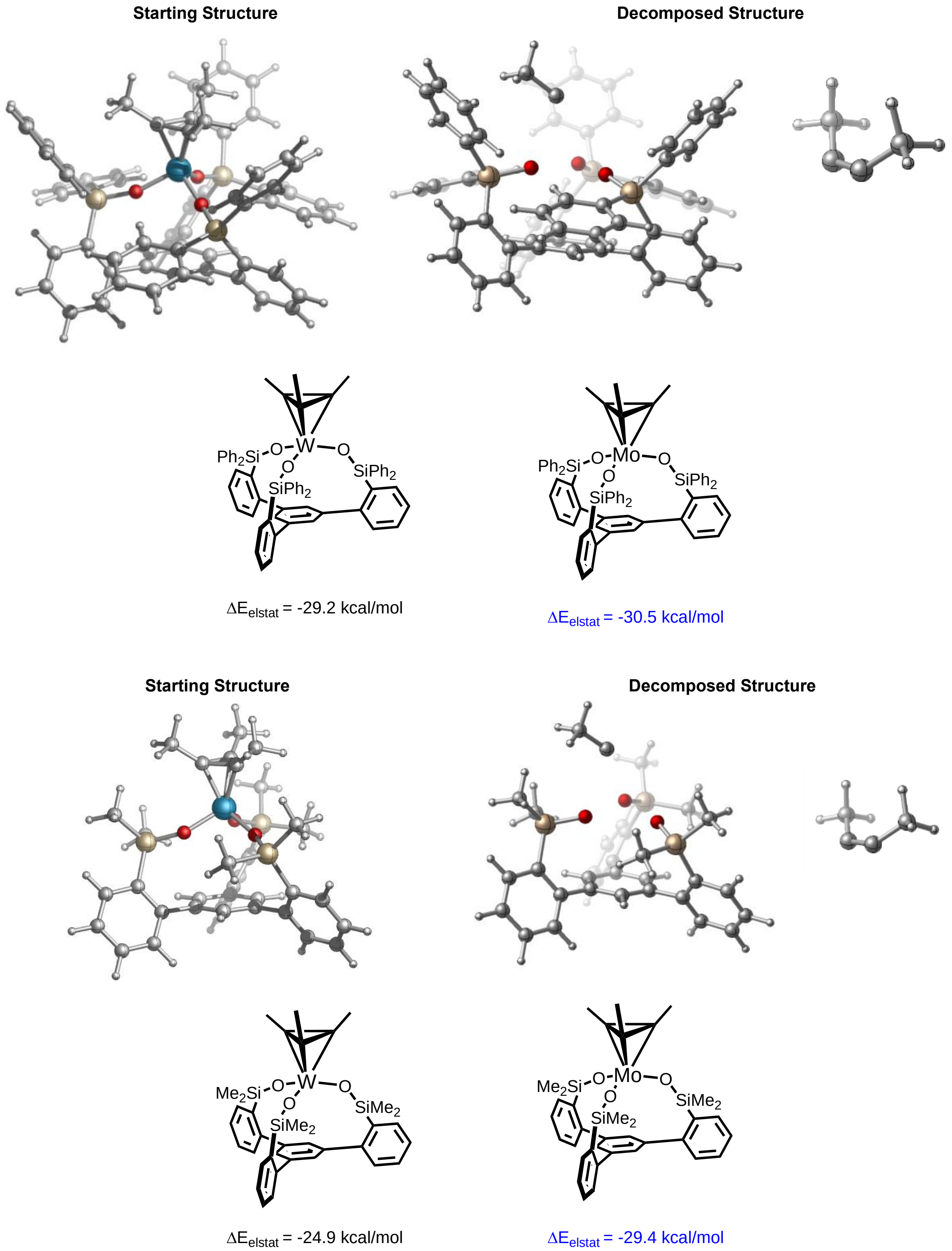

Figure S68. Electrostatic energies for the MTd intermediates, [W]-C and [Mo]-C, computed by the second-generation ALMO-EDA method using HF/6-311G(d,p) with the shown decomposed intermediates from the given starting structure. The electrostatic energies are lower for [Mo]-C than [W]-C and thus the electrostatic energy likely contributes to molybdenum's preference to form the MTd.

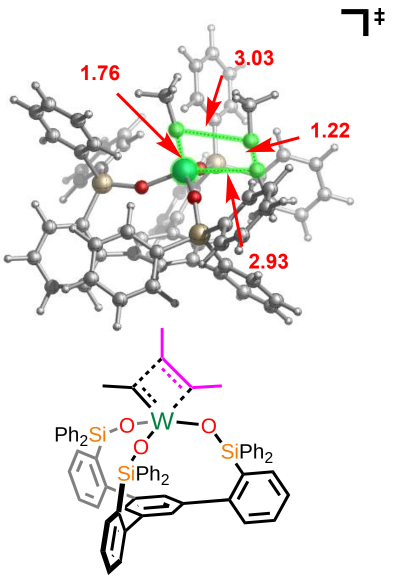

[W]-A-TS-Ph

ΔEdist = 13.3 kcal/mol

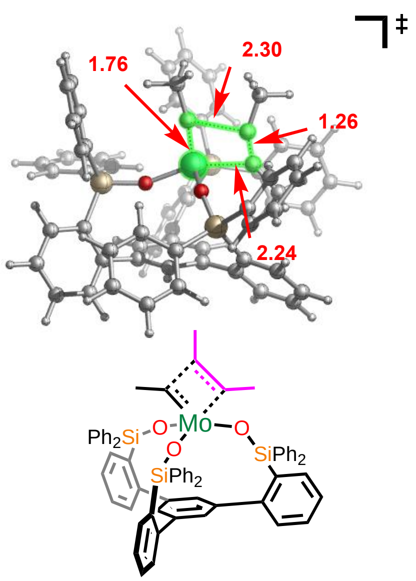

[Mo]-A-TS-Ph

ΔEdist =42.0 kcal/mol

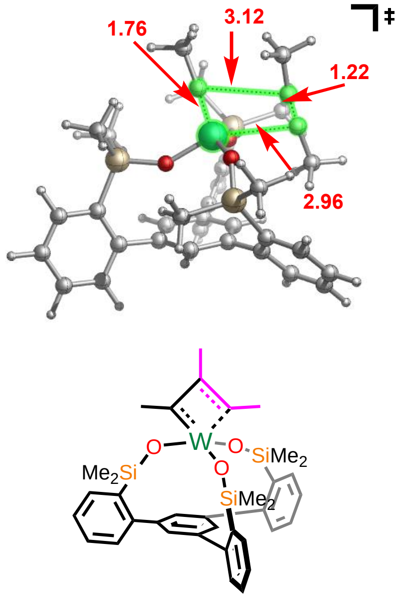

[W]-A-TS-Me

ΔEdist = 8.8 kcal/mol

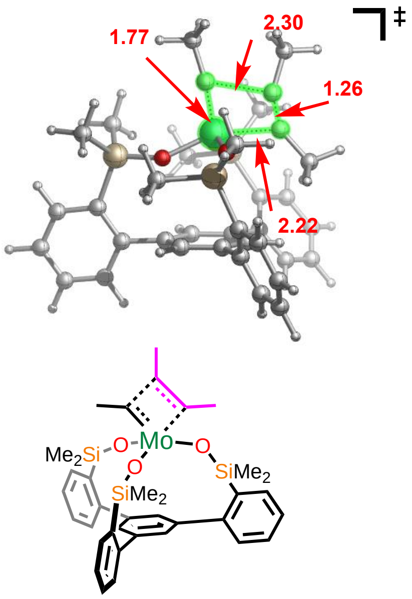

[Mo]-A-TS-Me

ΔEdist = 39.8 kcal/mol

Figure S69. Distortion energies for the formation of the MCBD intermediates, [W]-B and [Mo]-B, computed at the B3LYP-D3/def2TZVP-SDD(Mo)-CPCM(benzene)// B3LYP-D3/def2SVP-LANL2DZ(Mo)-CPCM(benzene) level of theory. The low distortion energy required to form [W]-B compared to that required to form [Mo]-B provides insight into the preference for the MCBD in the case of tungsten.

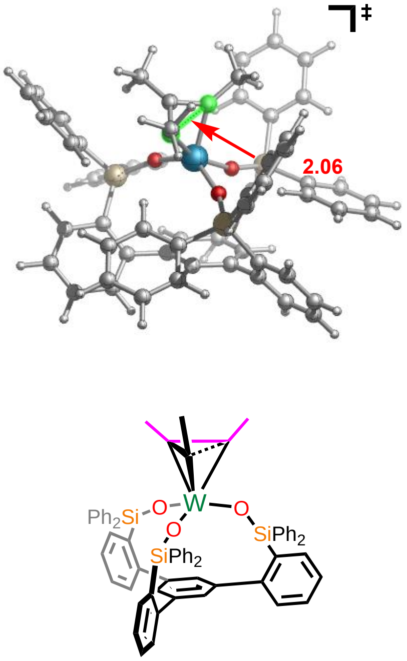

[W]-B-TS-Ph
ΔE_{dist} =0.4 kcal/mol

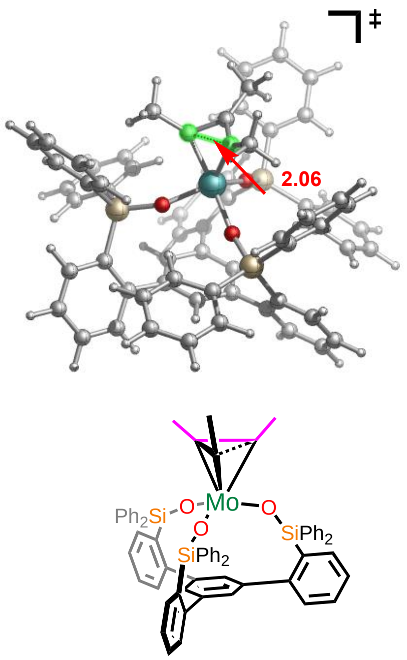

[Mo]-B-TS-Ph
ΔE_{dist} = 15.4 kcal/mol

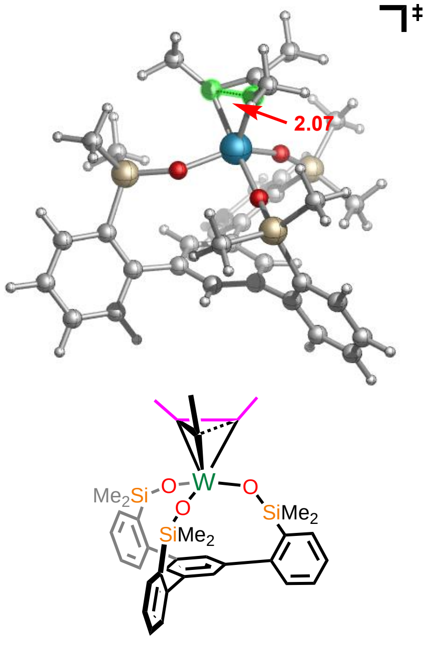

[W]-B-TS-Me
ΔE_{dist} = 0.3 kcal/mol

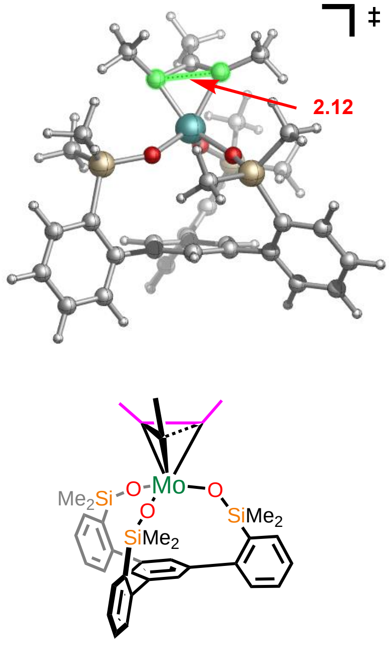

[Mo]-B-TS-Me
ΔE_{dist} = 9.4 kcal/mol

Figure S70. Distortion energies for the formation of the MTd intermediates, [W]-C and [Mo]-C, computed at the B3LYP-D3/def2TZVP-SDD(Mo)-CPCM(benzene)// B3LYP-D3/def2SVP-LANL2DZ(Mo)-CPCM(benzene) level of theory. In this case, the distortion energy to form [W]-C is lower than that to form [Mo]-C, and therefore the distortion energy does not control the preferred formation of the MTd intermediate in the molybdenum case.

Calculated Structures and Energies

[W]-A

B3LYP-D3/def2SVP-LANL2DZ(W)-CPCM(benzene)

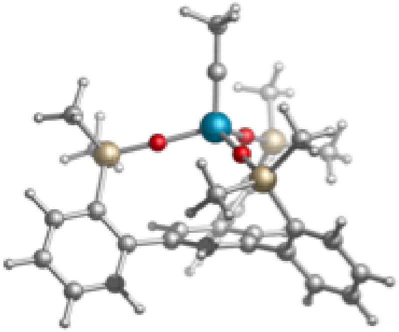

Zero-point correction= 0.588432 (Hartree/Particle)
Thermal correction to Energy= 0.631637
Thermal correction to Enthalpy= 0.632582
Thermal correction to Gibbs Free Energy= 0.511741
Sum of electronic and zero-point Energies= -2401.637346
Sum of electronic and thermal Energies= -2401.594140
Sum of electronic and thermal Enthalpies= -2401.593196
Sum of electronic and thermal Free Energies= -2401.714037

B3LYP-D3/def2TZVP-SDD(W)-CPCM(benzene)//B3LYP-D3/def2SVP-LANL2DZ(W)-CPCM(benzene)

HF = -2403.4449347

PBEPBE/def2TZVP-SDD(W)-CPCM(benzene)//B3LYP-D3/def2SVP-LANL2DZ(W)-CPCM(benzene)

HF = -2400.9668649

[W]-A-TS

B3LYP-D3/def2SVP-LANL2DZ(W)-CPCM(benzene)

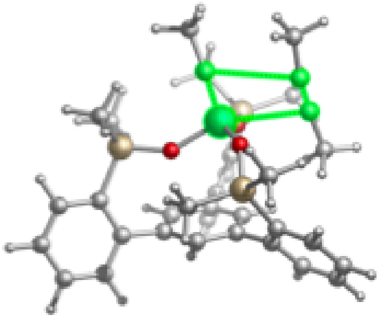

Imaginary frequency = -64.00 cm^{-1}
Zero-point correction= 0.675502 (Hartree/Particle)
Thermal correction to Energy= 0.723675
Thermal correction to Enthalpy= 0.724619
Thermal correction to Gibbs Free Energy= 0.595066
Sum of electronic and zero-point Energies= -2557.434867
Sum of electronic and thermal Energies= -2557.386694
Sum of electronic and thermal Enthalpies= -2557.385750
Sum of electronic and thermal Free Energies= -2557.515303

B3LYP-D3/def2TZVP-SDD(W)-CPCM(benzene)//B3LYP-D3/def2SVP-LANL2DZ(W)-CPCM(benzene)
HF = -2559.5021997

PBEPBE/def2TZVP-SDD(W)-CPCM(benzene)//B3LYP-D3/def2SVP-LANL2DZ(W)-CPCM(benzene)
HF = -2556.7746835

[W]-B

B3LYP-D3/def2SVP-LANL2DZ(W)-CPCM(benzene)

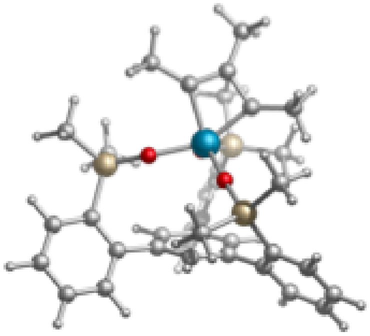

Zero-point correction= 0.677424 (Hartree/Particle)
Thermal correction to Energy= 0.725025
Thermal correction to Enthalpy= 0.725969
Thermal correction to Gibbs Free Energy= 0.598515
Sum of electronic and zero-point Energies= -2557.447293
Sum of electronic and thermal Energies= -2557.399692
Sum of electronic and thermal Enthalpies= -2557.398748
Sum of electronic and thermal Free Energies= -2557.526203

B3LYP-D3/def2TZVP-SDD(W)-CPCM(benzene)//B3LYP-D3/def2SVP-LANL2DZ(W)-CPCM(benzene)
HF = -2559.5114664
PBEPBE/def2TZVP-SDD(W)-CPCM(benzene)//B3LYP-D3/def2SVP-LANL2DZ(W)-CPCM(benzene)
HF = -2556.80022

[W]-B-TS-B'

B3LYP-D3/def2SVP-LANL2DZ(W)-CPCM(benzene)

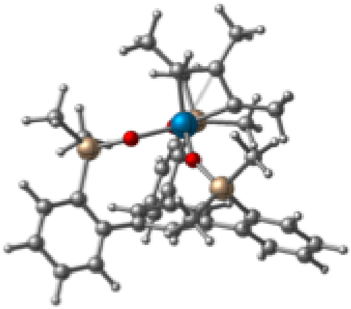

Imaginary frequency = -135.61 cm^{-1}
Zero-point correction= 0.676479 (Hartree/Particle)
Thermal correction to Energy= 0.723579
Thermal correction to Enthalpy= 0.724523
Thermal correction to Gibbs Free Energy= 0.597659
Sum of electronic and zero-point Energies= -2557.441541
Sum of electronic and thermal Energies= -2557.394440
Sum of electronic and thermal Enthalpies= -2557.393496
Sum of electronic and thermal Free Energies= -2557.520361
B3LYP-D3/def2TZVP-SDD(W)-CPCM(benzene)//B3LYP-D3/def2SVP-LANL2DZ(W)-CPCM(benzene)
HF = -2559.5050943
PBEPBE/def2TZVP-SDD(W)-CPCM(benzene)//B3LYP-D3/def2SVP-LANL2DZ(W)-CPCM(benzene)
HF = -2556.7945369

[W]-B'

B3LYP-D3/def2SVP-LANL2DZ(W)-CPCM(benzene)

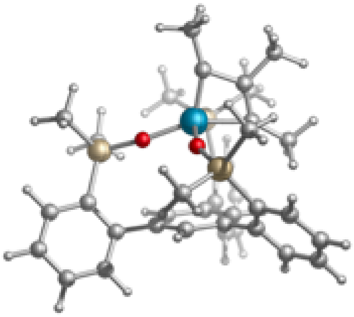

Zero-point correction= 0.677501 (Hartree/Particle)
Thermal correction to Energy= 0.725005
Thermal correction to Enthalpy= 0.725949
Thermal correction to Gibbs Free Energy= 0.598897
Sum of electronic and zero-point Energies= -2557.459719
Sum of electronic and thermal Energies= -2557.412216
Sum of electronic and thermal Enthalpies= -2557.411271
Sum of electronic and thermal Free Energies= -2557.538324

B3LYP-D3/def2TZVP-SDD(W)-CPCM(benzene)//B3LYP-D3/def2SVP-LANL2DZ(W)-CPCM(benzene)
HF = -2559.5256023
PBEPBE/def2TZVP-SDD(W)-CPCM(benzene)//B3LYP-D3/def2SVP-LANL2DZ(W)-CPCM(benzene)
HF = -2556.8148769

[W]-B-TS-ent-B

B3LYP-D3/def2SVP-LANL2DZ(W)-CPCM(benzene)

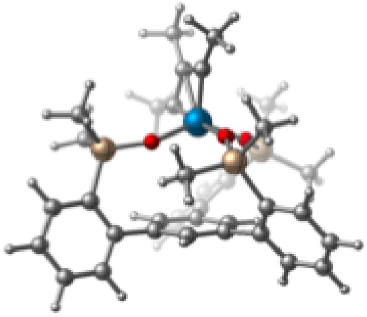

Imaginary frequency = -35.17 cm^{-1}
Zero-point correction= 0.676893 (Hartree/Particle)
Thermal correction to Energy= 0.723559
Thermal correction to Enthalpy= 0.724503
Thermal correction to Gibbs Free Energy= 0.599376
Sum of electronic and zero-point Energies= -2557.443042
Sum of electronic and thermal Energies= -2557.396376
Sum of electronic and thermal Enthalpies= -2557.395432
Sum of electronic and thermal Free Energies= -2557.520560

B3LYP-D3/def2TZVP-SDD(W)-CPCM(benzene)//B3LYP-D3/def2SVP-LANL2DZ(W)-CPCM(benzene)
HF = -2559.5078935
PBEPBE/def2TZVP-SDD(W)-CPCM(benzene)//B3LYP-D3/def2SVP-LANL2DZ(W)-CPCM(benzene)
HF = -2556.8004277

[W]-C

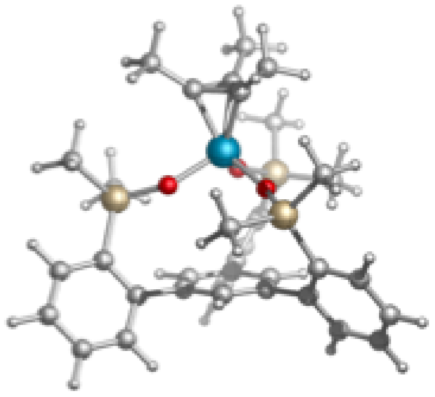

B3LYP-D3/def2SVP-LANL2DZ(W)-CPCM(benzene)

Zero-point correction= 0.678449 (Hartree/Particle)
Thermal correction to Energy= 0.725649
Thermal correction to Enthalpy= 0.726593
Thermal correction to Gibbs Free Energy= 0.600119
Sum of electronic and zero-point Energies= -2557.450847
Sum of electronic and thermal Energies= -2557.403648
Sum of electronic and thermal Enthalpies= -2557.402703
Sum of electronic and thermal Free Energies= -2557.529177

B3LYP-D3/def2TZVP-SDD(W)-CPCM(benzene)//B3LYP-D3/def2SVP-LANL2DZ(W)-CPCM(benzene)
HF = -2559.5130429
PBEPBE/def2TZVP-SDD(W)-CPCM(benzene)//B3LYP-D3/def2SVP-LANL2DZ(W)-CPCM(benzene)
HF = -2556.8112139

[W]-B-TS

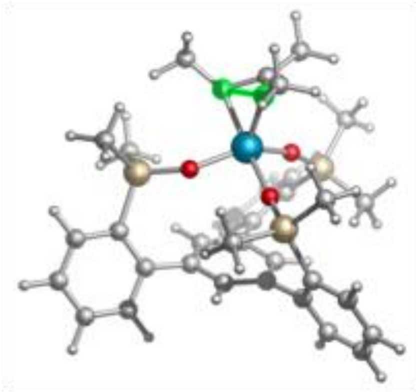

B3LYP-D3/def2SVP-LANL2DZ(W)-CPCM(benzene)

Imaginary frequency = -191.45 cm^{-1}
Zero-point correction= 0.676752 (Hartree/Particle)
Thermal correction to Energy= 0.723761
Thermal correction to Enthalpy= 0.724705
Thermal correction to Gibbs Free Energy= 0.598409
Sum of electronic and zero-point Energies= -2557.419417
Sum of electronic and thermal Energies= -2557.372408
Sum of electronic and thermal Enthalpies= -2557.371464
Sum of electronic and thermal Free Energies= -2557.497760

B3LYP-D3/def2TZVP-SDD(W)-CPCM(benzene)//B3LYP-D3/def2SVP-LANL2DZ(W)-CPCM(benzene)
HF = -2559.4843308
PBEPBE/def2TZVP-SDD(W)-CPCM(benzene)//B3LYP-D3/def2SVP-LANL2DZ(W)-CPCM(benzene)
HF = -2556.7790799

[W]-D-TS

B3LYP-D3/def2SVP-LANL2DZ(W)-CPCM(benzene)

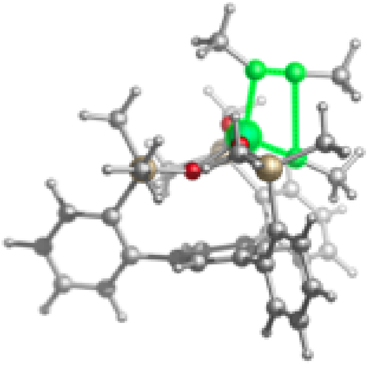

Imaginary frequency = -35.99 cm^{-1}
Zero-point correction = 0.676816 (Hartree/Particle)
Thermal correction to Energy= 0.723480
Thermal correction to Enthalpy= 0.724425
Thermal correction to Gibbs Free Energy= 0.601112
Sum of electronic and zero-point Energies= -2557.396992
Sum of electronic and thermal Energies= -2557.350328
Sum of electronic and thermal Enthalpies= -2557.349383
Sum of electronic and thermal Free Energies= -2557.472696

B3LYP-D3/def2TZVP-SDD(W)-CPCM(benzene)//B3LYP-D3/def2SVP-LANL2DZ(W)-CPCM(benzene)
HF = -2559.4641124
PBEPBE/def2TZVP-SDD(W)-CPCM(benzene)//B3LYP-D3/def2SVP-LANL2DZ(W)-CPCM(benzene)
HF = -2556.7429679

[W]-A-Ph

B3LYP-D3/def2SVP-LANL2DZ(W)-CPCM(benzene)

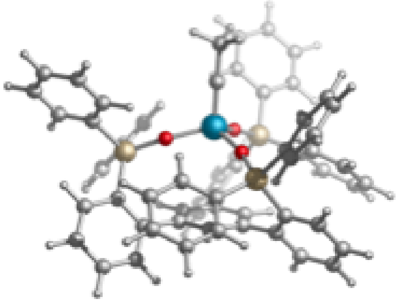

Zero-point correction= 0.918026 (Hartree/Particle)
Thermal correction to Energy= 0.978882
Thermal correction to Enthalpy= 0.979826
Thermal correction to Gibbs Free Energy= 0.816105
Sum of electronic and zero-point Energies= -3550.979886
Sum of electronic and thermal Energies= -3550.919029
Sum of electronic and thermal Enthalpies= -3550.918085
Sum of electronic and thermal Free Energies= -3551.081806

B3LYP-D3/def2TZVP-SDD(W)-CPCM(benzene)//B3LYP-D3/def2SVP-LANL2DZ(W)-CPCM(benzene)
HF = -3554.3198457
PBEPBE/def2TZVP-SDD(W)-CPCM(benzene)//B3LYP-D3/def2SVP-LANL2DZ(W)-CPCM(benzene)
HF = -3550.3014182

[W]-A-TS-Ph

B3LYP-D3/def2SVP-LANL2DZ(W)-CPCM(benzene)

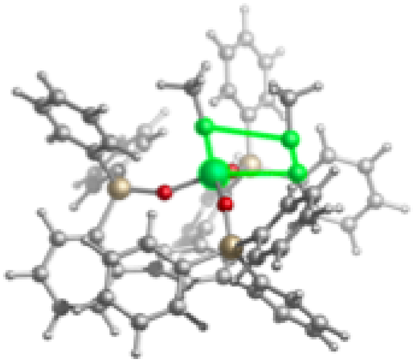

Imaginary frequency = -61.24 cm^{-1}
Zero-point correction= 1.004403 (Hartree/Particle)
Thermal correction to Energy= 1.070417
Thermal correction to Enthalpy= 1.071361
Thermal correction to Gibbs Free Energy= 0.900808
Sum of electronic and zero-point Energies= -3706.779892
Sum of electronic and thermal Energies= -3706.713879
Sum of electronic and thermal Enthalpies= -3706.712935
Sum of electronic and thermal Free Energies= -3706.883487

B3LYP-D3/def2TZVP-SDD(W)-CPCM(benzene)//B3LYP-D3/def2SVP-LANL2DZ(W)-CPCM(benzene)
HF = -3710.3792583
PBEPBE/def2TZVP-SDD(W)-CPCM(benzene)//B3LYP-D3/def2SVP-LANL2DZ(W)-CPCM(benzene)
HF = -3706.1076251

[W]-B-Ph

B3LYP-D3/def2SVP-LANL2DZ(W)-CPCM(benzene)

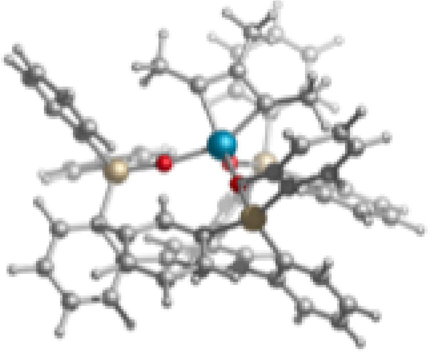

Zero-point correction= 1.006845 (Hartree/Particle)
Thermal correction to Energy= 1.072263
Thermal correction to Enthalpy= 1.073207
Thermal correction to Gibbs Free Energy= 0.904075
Sum of electronic and zero-point Energies= -3706.795399
Sum of electronic and thermal Energies= -3706.729981
Sum of electronic and thermal Enthalpies= -3706.729037
Sum of electronic and thermal Free Energies= -3706.898169

B3LYP-D3/def2TZVP-SDD(W)-CPCM(benzene)//B3LYP-D3/def2SVP-LANL2DZ(W)-CPCM(benzene)
HF = -3710.3927507
PBEPBE/def2TZVP-SDD(W)-CPCM(benzene)//B3LYP-D3/def2SVP-LANL2DZ(W)-CPCM(benzene)
HF = -3706.1336769

[W]-B-TS-B'-Ph

B3LYP-D3/def2SVP-LANL2DZ(W)-CPCM(benzene)

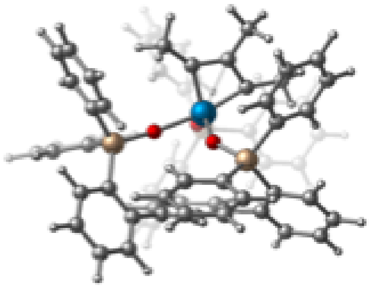

Imaginary frequency = -115.91 cm^{-1}
Zero-point correction= 1.005410 (Hartree/Particle)
Thermal correction to Energy= 1.070377
Thermal correction to Enthalpy= 1.071321
Thermal correction to Gibbs Free Energy= 0.902417
Sum of electronic and zero-point Energies= -3706.782329
Sum of electronic and thermal Energies= -3706.717362
Sum of electronic and thermal Enthalpies= -3706.716418
Sum of electronic and thermal Free Energies= -3706.885323

B3LYP-D3/def2TZVP-SDD(W)-CPCM(benzene)//B3LYP-D3/def2SVP-LANL2DZ(W)-CPCM(benzene)
HF = -3710.3811163
PBEPBE/def2TZVP-SDD(W)-CPCM(benzene)//B3LYP-D3/def2SVP-LANL2DZ(W)-CPCM(benzene)
HF = -3706.1342496

[W]-B'-Ph

B3LYP-D3/def2SVP-LANL2DZ(W)-CPCM(benzene)

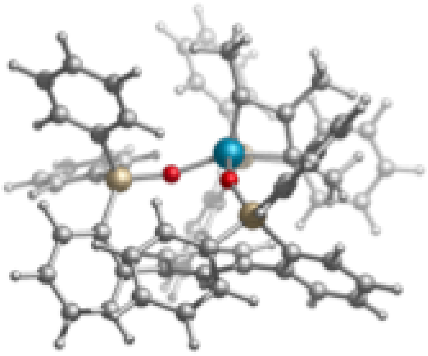

Zero-point correction= 1.006770 (Hartree/Particle)
Thermal correction to Energy= 1.071942
Thermal correction to Enthalpy= 1.072886
Thermal correction to Gibbs Free Energy= 0.903533
Sum of electronic and zero-point Energies= -3706.805211
Sum of electronic and thermal Energies= -3706.740038
Sum of electronic and thermal Enthalpies= -3706.739094
Sum of electronic and thermal Free Energies= -3706.908447

B3LYP-D3/def2TZVP-SDD(W)-CPCM(benzene)//B3LYP-D3/def2SVP-LANL2DZ(W)-CPCM(benzene)
HF = -3710.4039999
PBEPBE/def2TZVP-SDD(W)-CPCM(benzene)//B3LYP-D3/def2SVP-LANL2DZ(W)-CPCM(benzene)
HF = -3706.1525639

[W]-C-Ph

B3LYP-D3/def2SVP-LANL2DZ(W)-CPCM(benzene)

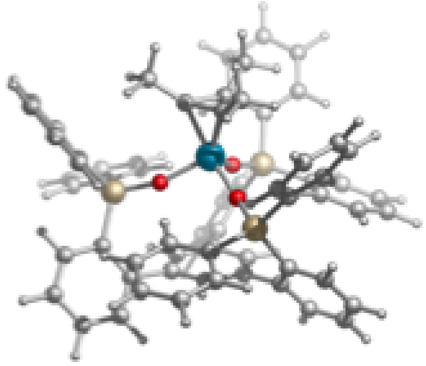

Zero-point correction= 1.007112 (Hartree/Particle)
Thermal correction to Energy= 1.072424
Thermal correction to Enthalpy= 1.073368
Thermal correction to Gibbs Free Energy= 0.905894
Sum of electronic and zero-point Energies= -3706.804354
Sum of electronic and thermal Energies= -3706.739043
Sum of electronic and thermal Enthalpies= -3706.738098
Sum of electronic and thermal Free Energies= -3706.905572

B3LYP-D3/def2TZVP-SDD(W)-CPCM(benzene)//B3LYP-D3/def2SVP-LANL2DZ(W)-CPCM(benzene)
HF = -3710.397567
PBEPBE/def2TZVP-SDD(W)-CPCM(benzene)//B3LYP-D3/def2SVP-LANL2DZ(W)-CPCM(benzene)
HF = -3706.1463563

[W]-B-TS-Ph

B3LYP-D3/def2SVP-LANL2DZ(W)-CPCM(benzene)

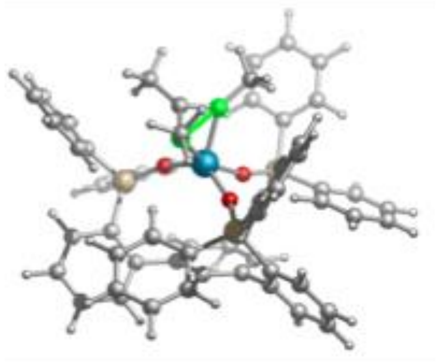

Imaginary frequency = -212.31 cm^{-1}
Zero-point correction= 1.004097 (Hartree/Particle)
Thermal correction to Energy= 1.069572
Thermal correction to Enthalpy= 1.070516
Thermal correction to Gibbs Free Energy= 0.899188
Sum of electronic and zero-point Energies= -3706.774043
Sum of electronic and thermal Energies= -3706.708568
Sum of electronic and thermal Enthalpies= -3706.707624
Sum of electronic and thermal Free Energies= -3706.878951

B3LYP-D3/def2TZVP-SDD(W)-CPCM(benzene)//B3LYP-D3/def2SVP-LANL2DZ(W)-CPCM(benzene)
HF = -3710.3697736
PBEPBE/def2TZVP-SDD(W)-CPCM(benzene)//B3LYP-D3/def2SVP-LANL2DZ(W)-CPCM(benzene)
HF = -3706.118118

[W]-D-TS-Ph

B3LYP-D3/def2SVP-LANL2DZ(W)-CPCM(benzene)

Imaginary frequency = -108.94 cm^{-1}

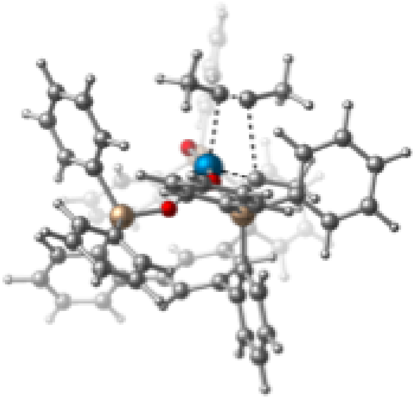

Zero-point correction= 1.004743 (Hartree/Particle)
Thermal correction to Energy= 1.069366
Thermal correction to Enthalpy= 1.070310
Thermal correction to Gibbs Free Energy= 0.903823
Sum of electronic and zero-point Energies= -3706.721912
Sum of electronic and thermal Energies= -3706.657289
Sum of electronic and thermal Enthalpies= -3706.656345
Sum of electronic and thermal Free Energies= -3706.822832

B3LYP-D3/def2TZVP-SDD(W)-CPCM(benzene)//B3LYP-D3/def2SVP-LANL2DZ(W)-CPCM(benzene)
HF = -3710.3202413
PBEPBE/def2TZVP-SDD(W)-CPCM(benzene)//B3LYP-D3/def2SVP-LANL2DZ(W)-CPCM(benzene)
HF = -3706.0555285

[Mo]-A

B3LYP-D3/def2SVP-LANL2DZ(Mo)-CPCM(benzene)

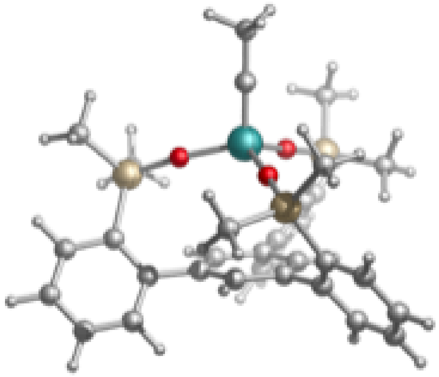

Zero-point correction= 0.588330 (Hartree/Particle)
Thermal correction to Energy= 0.631455
Thermal correction to Enthalpy= 0.632399
Thermal correction to Gibbs Free Energy= 0.511970
Sum of electronic and zero-point Energies= -2401.306719
Sum of electronic and thermal Energies= -2401.263594
Sum of electronic and thermal Enthalpies= -2401.262650
Sum of electronic and thermal Free Energies= -2401.383078

B3LYP-D3/def2TZVP-SDD(Mo)-CPCM(benzene)//B3LYP-D3/def2SVP-LANL2DZ(Mo)-CPCM(benzene)
HF = -2404.5229548
PBEPBE/def2TZVP-SDD(Mo)-CPCM(benzene)//B3LYP-D3/def2SVP-LANL2DZ(Mo)-CPCM(benzene)
HF = -2402.0456315

[Mo]-A-TS

B3LYP-D3/def2SVP-LANL2DZ(Mo)-CPCM(benzene)

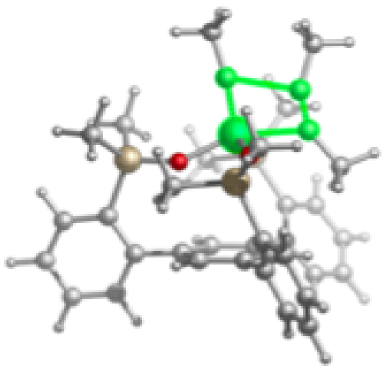

Imaginary frequency = -108.38 cm^{-1}
Zero-point correction= 0.675093 (Hartree/Particle)
Thermal correction to Energy= 0.722735
Thermal correction to Enthalpy= 0.723679
Thermal correction to Gibbs Free Energy= 0.596955
Sum of electronic and zero-point Energies= -2557.096285
Sum of electronic and thermal Energies= -2557.048643
Sum of electronic and thermal Enthalpies= -2557.047699
Sum of electronic and thermal Free Energies= -2557.174423

B3LYP-D3/def2TZVP-SDD(Mo)-CPCM(benzene)//B3LYP-D3/def2SVP-LANL2DZ(Mo)-CPCM(benzene)
HF = -2560.5741152
PBEPBE/def2TZVP-SDD(Mo)-CPCM(benzene)//B3LYP-D3/def2SVP-LANL2DZ(Mo)-CPCM(benzene)
HF = -2557.8584398

[Mo]-B

B3LYP-D3/def2SVP-LANL2DZ(Mo)-CPCM(benzene)

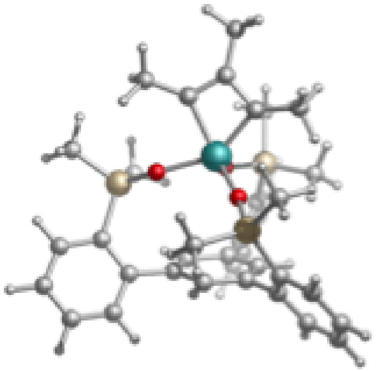

Zero-point correction= 0.677811 (Hartree/Particle)
Thermal correction to Energy= 0.725151
Thermal correction to Enthalpy= 0.726095
Thermal correction to Gibbs Free Energy= 0.599392
Sum of electronic and zero-point Energies= -2557.107330
Sum of electronic and thermal Energies= -2557.059989
Sum of electronic and thermal Enthalpies= -2557.059045
Sum of electronic and thermal Free Energies= -2557.185748

B3LYP-D3/def2TZVP-SDD(Mo)-CPCM(benzene)//B3LYP-D3/def2SVP-LANL2DZ(Mo)-CPCM(benzene)
HF = -2560.5802534
PBEPBE/def2TZVP-SDD(Mo)-CPCM(benzene)//B3LYP-D3/def2SVP-LANL2DZ(Mo)-CPCM(benzene)
HF = -2557.871772

[Mo]-B-TS-B’

B3LYP-D3/def2SVP-LANL2DZ(Mo)-CPCM(benzene)

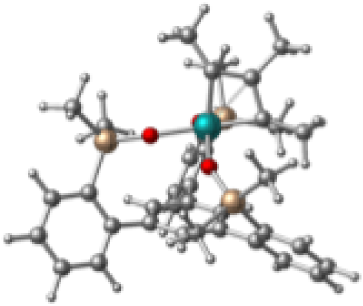

Imaginary frequency = -200.55 cm^{-1}
Zero-point correction= 0.676296 (Hartree/Particle)
Thermal correction to Energy= 0.723269
Thermal correction to Enthalpy= 0.724213
Thermal correction to Gibbs Free Energy= 0.598902
Sum of electronic and zero-point Energies= -2557.092269
Sum of electronic and thermal Energies= -2557.045296
Sum of electronic and thermal Enthalpies= -2557.044352
Sum of electronic and thermal Free Energies= -2557.169664

B3LYP-D3/def2TZVP-SDD(Mo)-CPCM(benzene)//B3LYP-D3/def2SVP-LANL2DZ(Mo)-CPCM(benzene)
HF = -2560.5658088
PBEPBE/def2TZVP-SDD(Mo)-CPCM(benzene)//B3LYP-D3/def2SVP-LANL2DZ(Mo)-CPCM(benzene)
HF = -2557.8592625

[Mo]-B’

B3LYP-D3/def2SVP-LANL2DZ(Mo)-CPCM(benzene)

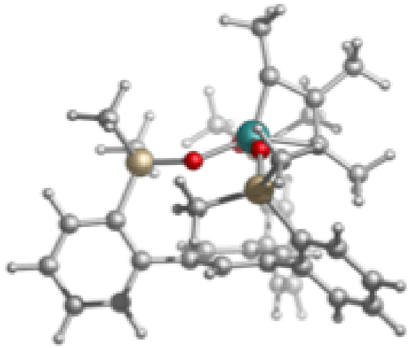

Zero-point correction= 0.677674 (Hartree/Particle)
Thermal correction to Energy= 0.724866
Thermal correction to Enthalpy= 0.725810
Thermal correction to Gibbs Free Energy= 0.599312
Sum of electronic and zero-point Energies= -2557.108581
Sum of electronic and thermal Energies= -2557.061389
Sum of electronic and thermal Enthalpies= -2557.060444
Sum of electronic and thermal Free Energies= -2557.186942

B3LYP-D3/def2TZVP-SDD(Mo)-CPCM(benzene)//B3LYP-D3/def2SVP-LANL2DZ(Mo)-CPCM(benzene)
HF = -2560.5851268
PBEPBE/def2TZVP-SDD(Mo)-CPCM(benzene)//B3LYP-D3/def2SVP-LANL2DZ(Mo)-CPCM(benzene)
HF = -2557.8759407

[Mo]-B-TS-ent-B

B3LYP-D3/def2SVP-LANL2DZ(Mo)-CPCM(benzene)

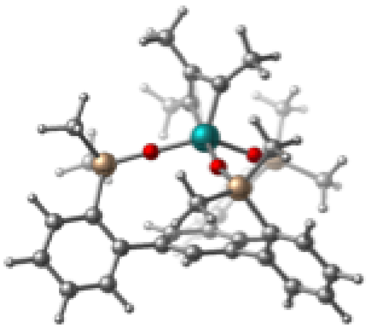

Imaginary frequency = -9.36 cm^{-1}
Zero-point correction= 0.675593 (Hartree/Particle)
Thermal correction to Energy= 0.723002
Thermal correction to Enthalpy= 0.723946
Thermal correction to Gibbs Free Energy= 0.597023
Sum of electronic and zero-point Energies= -2557.101177
Sum of electronic and thermal Energies= -2557.053768
Sum of electronic and thermal Enthalpies= -2557.052824
Sum of electronic and thermal Free Energies= -2557.179746

B3LYP-D3/def2TZVP-SDD(Mo)-CPCM(benzene)//B3LYP-D3/def2SVP-LANL2DZ(Mo)-CPCM(benzene)
HF = -2560.5742476
PBEPBE/def2TZVP-SDD(Mo)-CPCM(benzene)//B3LYP-D3/def2SVP-LANL2DZ(Mo)-CPCM(benzene)
HF = -2557.8660984

[Mo]-C

B3LYP-D3/def2SVP-LANL2DZ(Mo)-CPCM(benzene)

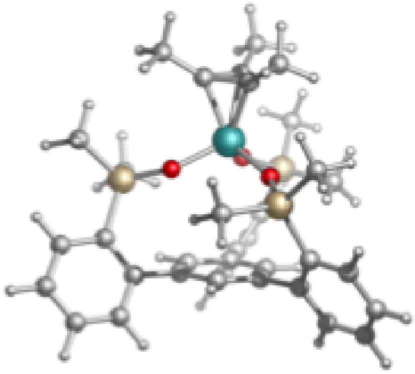

Zero-point correction= 0.678894 (Hartree/Particle)
Thermal correction to Energy= 0.725931
Thermal correction to Enthalpy= 0.726875
Thermal correction to Gibbs Free Energy= 0.601238
Sum of electronic and zero-point Energies= -2557.123525
Sum of electronic and thermal Energies= -2557.076489
Sum of electronic and thermal Enthalpies= -2557.075544
Sum of electronic and thermal Free Energies= -2557.201181

B3LYP-D3/def2TZVP-SDD(Mo)-CPCM(benzene)//B3LYP-D3/def2SVP-LANL2DZ(Mo)-CPCM(benzene)
HF = -2560.5923248
PBEPBE/def2TZVP-SDD(Mo)-CPCM(benzene)//B3LYP-D3/def2SVP-LANL2DZ(Mo)-CPCM(benzene)
HF = -2557.8885247

[Mo]-B-TS

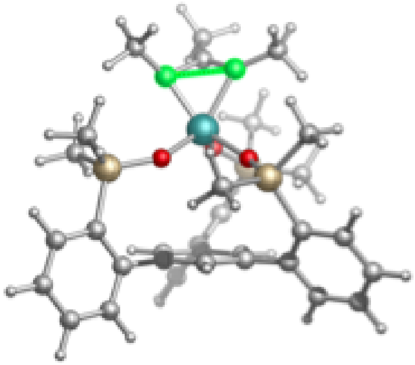

B3LYP-D3/def2SVP-LANL2DZ(Mo)-CPCM(benzene)

Imaginary frequency = -179.76 cm^{-1}
Zero-point correction= 0.676178 (Hartree/Particle)
Thermal correction to Energy= 0.723241
Thermal correction to Enthalpy= 0.724185
Thermal correction to Gibbs Free Energy= 0.598258
Sum of electronic and zero-point Energies= -2557.086727
Sum of electronic and thermal Energies= -2557.039664
Sum of electronic and thermal Enthalpies= -2557.038719
Sum of electronic and thermal Free Energies= -2557.164647

B3LYP-D3/def2TZVP-SDD(Mo)-CPCM(benzene)//B3LYP-D3/def2SVP-LANL2DZ(Mo)-CPCM(benzene)
HF = -2560.5594384
PBEPBE/def2TZVP-SDD(Mo)-CPCM(benzene)//B3LYP-D3/def2SVP-LANL2DZ(Mo)-CPCM(benzene)
HF = -2557.8535094

[Mo]-D-TS

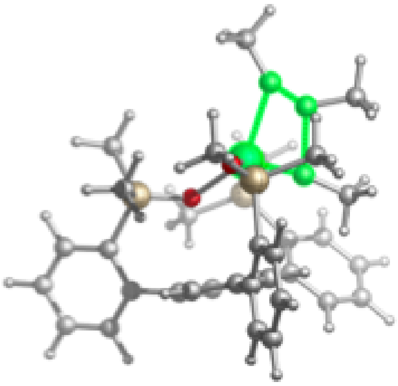

B3LYP-D3/def2SVP-LANL2DZ(Mo)-CPCM(benzene)

Imaginary frequency = -249.08 cm^{-1}
Zero-point correction= 0.675819 (Hartree/Particle)
Thermal correction to Energy= 0.722782
Thermal correction to Enthalpy= 0.723726
Thermal correction to Gibbs Free Energy= 0.599520
Sum of electronic and zero-point Energies= -2557.055119
Sum of electronic and thermal Energies= -2557.008156
Sum of electronic and thermal Enthalpies= -2557.007212
Sum of electronic and thermal Free Energies= -2557.131419

B3LYP-D3/def2TZVP-SDD(Mo)-CPCM(benzene)//B3LYP-D3/def2SVP-LANL2DZ(Mo)-CPCM(benzene)
HF = -2560.5287423
PBEPBE/def2TZVP-SDD(Mo)-CPCM(benzene)//B3LYP-D3/def2SVP-LANL2DZ(Mo)-CPCM(benzene)
HF = -2557.816809

[Mo]-A-Ph

B3LYP-D3/def2SVP-LANL2DZ(Mo)-CPCM(benzene)

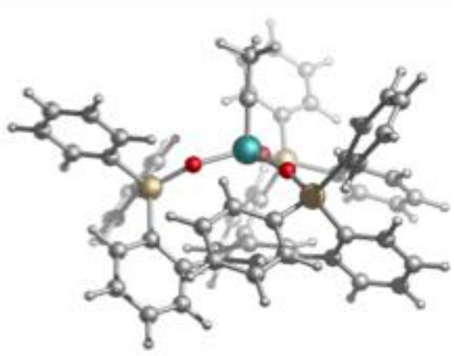

Zero-point correction= 0.917179 (Hartree/Particle)
Thermal correction to Energy= 0.978141
Thermal correction to Enthalpy= 0.979086
Thermal correction to Gibbs Free Energy= 0.814927
Sum of electronic and zero-point Energies= -3550.648228
Sum of electronic and thermal Energies= -3550.587266
Sum of electronic and thermal Enthalpies= -3550.586322
Sum of electronic and thermal Free Energies= -3550.750480

B3LYP-D3/def2TZVP-SDD(Mo)-CPCM(benzene)//B3LYP-D3/def2SVP-LANL2DZ(Mo)-CPCM(benzene)
HF = -3555.396031
PBEPBE/def2TZVP-SDD(Mo)-CPCM(benzene)//B3LYP-D3/def2SVP-LANL2DZ(Mo)-CPCM(benzene)
HF = -3551.3819436

[Mo]-A-TS-Ph

B3LYP-D3/def2SVP-LANL2DZ(Mo)-CPCM(benzene)

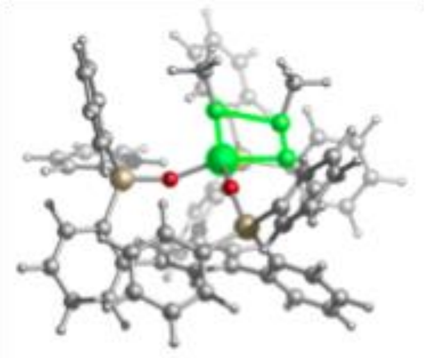

Imaginary frequency = -96.26 cm^{-1}
Zero-point correction= 1.004448 (Hartree/Particle)
Thermal correction to Energy= 1.069819
Thermal correction to Enthalpy= 1.070764
Thermal correction to Gibbs Free Energy= 0.901741
Sum of electronic and zero-point Energies= -3706.439514
Sum of electronic and thermal Energies= -3706.374143
Sum of electronic and thermal Enthalpies= -3706.373198
Sum of electronic and thermal Free Energies= -3706.542221

B3LYP-D3/def2TZVP-SDD(Mo)-CPCM(benzene)//B3LYP-D3/def2SVP-LANL2DZ(Mo)-CPCM(benzene)
HF = -3711.4494332
PBEPBE/def2TZVP-SDD(Mo)-CPCM(benzene)//B3LYP-D3/def2SVP-LANL2DZ(Mo)-CPCM(benzene)
HF = -3707.1943604

[Mo]-B-Ph

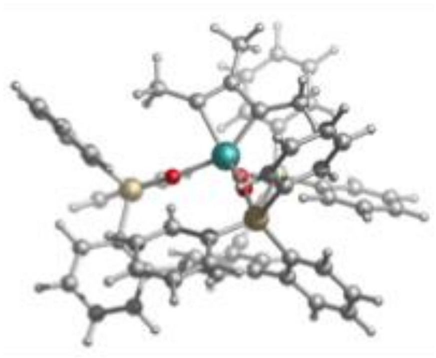

B3LYP-D3/def2SVP-LANL2DZ(Mo)-CPCM(benzene)

Zero-point correction= 1.006303 (Hartree/Particle)
Thermal correction to Energy= 1.071843
Thermal correction to Enthalpy= 1.072787
Thermal correction to Gibbs Free Energy= 0.904069
Sum of electronic and zero-point Energies= -3706.455213
Sum of electronic and thermal Energies= -3706.389672
Sum of electronic and thermal Enthalpies= -3706.388728
Sum of electronic and thermal Free Energies= -3706.557447

B3LYP-D3/def2TZVP-SDD(Mo)-CPCM(benzene)//B3LYP-D3/def2SVP-LANL2DZ(Mo)-CPCM(benzene)
HF = -3711.459603
PBEPBE/def2TZVP-SDD(Mo)-CPCM(benzene)//B3LYP-D3/def2SVP-LANL2DZ(Mo)-CPCM(benzene)
HF = -3707.2048744

[Mo]-B-TS-B'-Ph

B3LYP-D3/def2SVP-LANL2DZ(Mo)-CPCM(benzene)

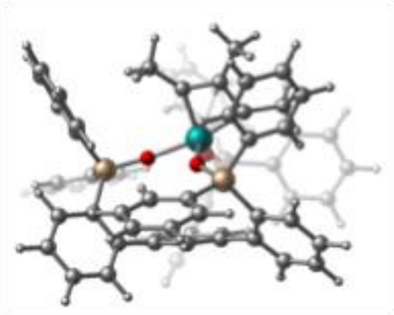

Imaginary frequency = -211.97 cm^{-1}
Zero-point correction= 1.004988 (Hartree/Particle)
Thermal correction to Energy= 1.070033
Thermal correction to Enthalpy= 1.070977
Thermal correction to Gibbs Free Energy= 0.901728
Sum of electronic and zero-point Energies= -3706.433662
Sum of electronic and thermal Energies= -3706.368617
Sum of electronic and thermal Enthalpies= -3706.367673
Sum of electronic and thermal Free Energies= -3706.536923

B3LYP-D3/def2TZVP-SDD(Mo)-CPCM(benzene)//B3LYP-D3/def2SVP-LANL2DZ(Mo)-CPCM(benzene)
HF = -3711.4414724
PBEPBE/def2TZVP-SDD(Mo)-CPCM(benzene)//B3LYP-D3/def2SVP-LANL2DZ(Mo)-CPCM(benzene)
HF = -3707.1987306

[Mo]-B'-Ph

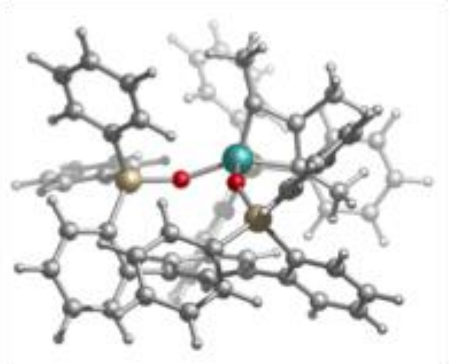

B3LYP-D3/def2SVP-LANL2DZ(Mo)-CPCM(benzene)

Zero-point correction= 1.005955 (Hartree/Particle)
Thermal correction to Energy= 1.071363
Thermal correction to Enthalpy= 1.072308
Thermal correction to Gibbs Free Energy= 0.902130
Sum of electronic and zero-point Energies= -3706.454986
Sum of electronic and thermal Energies= -3706.389578
Sum of electronic and thermal Enthalpies= -3706.388634
Sum of electronic and thermal Free Energies= -3706.558812

B3LYP-D3/def2TZVP-SDD(Mo)-CPCM(benzene)//B3LYP-D3/def2SVP-LANL2DZ(Mo)-CPCM(benzene)
HF = -3711.4630733
PBEPBE/def2TZVP-SDD(Mo)-CPCM(benzene)//B3LYP-D3/def2SVP-LANL2DZ(Mo)-CPCM(benzene)
HF = -3707.2163281

[Mo]-C-Ph

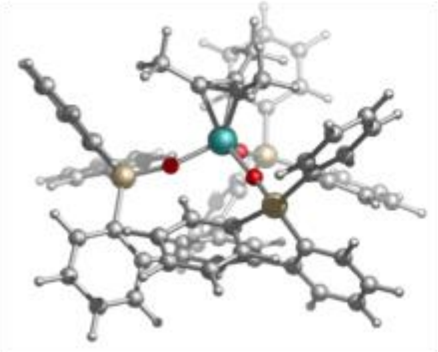

B3LYP-D3/def2SVP-LANL2DZ(Mo)-CPCM(benzene)

Zero-point correction= 1.006905 (Hartree/Particle)
Thermal correction to Energy= 1.072289
Thermal correction to Enthalpy= 1.073233
Thermal correction to Gibbs Free Energy= 0.905359
Sum of electronic and zero-point Energies= -3706.475869
Sum of electronic and thermal Energies= -3706.410485
Sum of electronic and thermal Enthalpies= -3706.409541
Sum of electronic and thermal Free Energies= -3706.577415

B3LYP-D3/def2TZVP-SDD(Mo)-CPCM(benzene)//B3LYP-D3/def2SVP-LANL2DZ(Mo)-CPCM(benzene)
HF = -3711.4760852
PBEPBE/def2TZVP-SDD(Mo)-CPCM(benzene)//B3LYP-D3/def2SVP-LANL2DZ(Mo)-CPCM(benzene)
HF = -3707.227112

[Mo]-B-TS

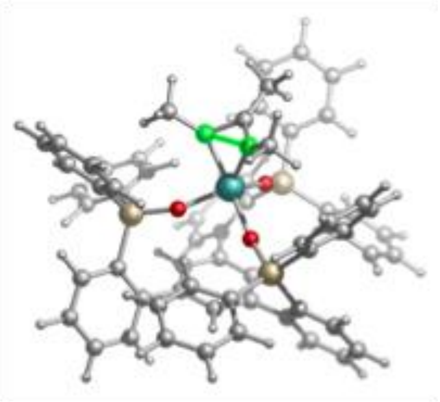

B3LYP-D3/def2SVP-LANL2DZ(Mo)-CPCM(benzene)

Imaginary frequency = -190.20 cm^{-1}
Zero-point correction= 1.003942 (Hartree/Particle)
Thermal correction to Energy= 1.069519
Thermal correction to Enthalpy= 1.070463
Thermal correction to Gibbs Free Energy= 0.899195
Sum of electronic and zero-point Energies= -3706.438866
Sum of electronic and thermal Energies= -3706.373289
Sum of electronic and thermal Enthalpies= -3706.372345
Sum of electronic and thermal Free Energies= -3706.543613

B3LYP-D3/def2TZVP-SDD(Mo)-CPCM(benzene)//B3LYP-D3/def2SVP-LANL2DZ(Mo)-CPCM(benzene)
HF = -3711.4425462
PBEPBE/def2TZVP-SDD(Mo)-CPCM(benzene)//B3LYP-D3/def2SVP-LANL2DZ(Mo)-CPCM(benzene)
HF = -3707.1925016

[Mo]-D-TS-Ph

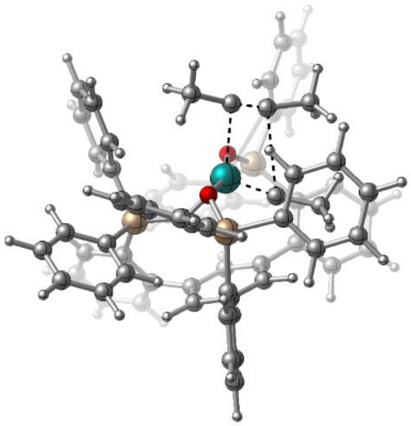

B3LYP-D3/def2SVP-LANL2DZ(Mo)-CPCM(benzene)

Imaginary frequency = -35.52 cm^{-1}
Zero-point correction= 1.004363 (Hartree/Particle)
Thermal correction to Energy= 1.068922
Thermal correction to Enthalpy= 1.069866
Thermal correction to Gibbs Free Energy= 0.904287
Sum of electronic and zero-point Energies= -3706.389618
Sum of electronic and thermal Energies= -3706.325059
Sum of electronic and thermal Enthalpies= -3706.324115
Sum of electronic and thermal Free Energies= -3706.489694

B3LYP-D3/def2TZVP-SDD(Mo)-CPCM(benzene)//B3LYP-D3/def2SVP-LANL2DZ(Mo)-CPCM(benzene)
HF = -3711.4375019
PBEPBE/def2TZVP-SDD(Mo)-CPCM(benzene)//B3LYP-D3/def2SVP-LANL2DZ(Mo)-CPCM(benzene)
HF = -3707.1314337

[W]-Ar’

B3LYP-D3/def2SVP-LANL2DZ(W)-CPCM(benzene)

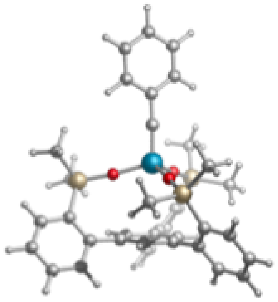

Zero-point correction= 0.642570 (Hartree/Particle)
Thermal correction to Energy= 0.688668
Thermal correction to Enthalpy= 0.689612
Thermal correction to Gibbs Free Energy= 0.559953
Sum of electronic and zero-point Energies= -2593.202538
Sum of electronic and thermal Energies= -2593.156440
Sum of electronic and thermal Enthalpies= -2593.155496
Sum of electronic and thermal Free Energies= -2593.285155

B3LYP-D3/def2TZVP-SDD(W)-CPCM(benzene)//B3LYP-D3/def2SVP-LANL2DZ(W)-CPCM(benzene)
HF = -2595.2653957

[W]-Ar’-TS

B3LYP-D3/def2SVP-LANL2DZ(W)-CPCM(benzene)

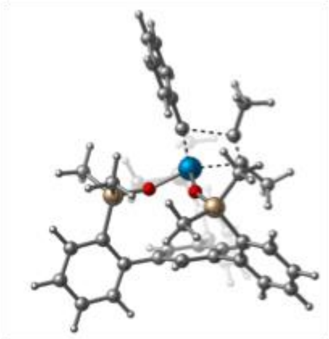

Imaginary frequency = -76.91 cm^{-1}
Zero-point correction= 0.730082 (Hartree/Particle)
Thermal correction to Energy= 0.780361
Thermal correction to Enthalpy= 0.781305
Thermal correction to Gibbs Free Energy= 0.647718
Sum of electronic and zero-point Energies= -2749.007213
Sum of electronic and thermal Energies= -2748.956934
Sum of electronic and thermal Enthalpies= -2748.955989
Sum of electronic and thermal Free Energies= -2749.089576

B3LYP-D3/def2TZVP-SDD(W)-CPCM(benzene)//B3LYP-D3/def2SVP-LANL2DZ(W)-CPCM(benzene)
HF = -2751.3314256

[W]-C’

B3LYP-D3/def2SVP-LANL2DZ(W)-CPCM(benzene)

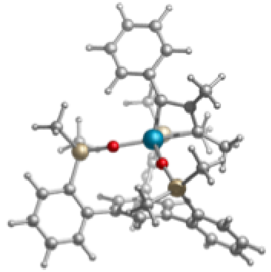

Zero-point correction= 0.731396 (Hartree/Particle)
Thermal correction to Energy= 0.782017
Thermal correction to Enthalpy= 0.782961
Thermal correction to Gibbs Free Energy= 0.647715
Sum of electronic and zero-point Energies= -2749.018238
Sum of electronic and thermal Energies= -2748.967617
Sum of electronic and thermal Enthalpies= -2748.966673
Sum of electronic and thermal Free Energies= -2749.101919

B3LYP-D3/def2TZVP-SDD(W)-CPCM(benzene)//B3LYP-D3/def2SVP-LANL2DZ(W)-CPCM(benzene)
HF = -2751.3378612

[W]-ent-C'

B3LYP-D3/def2SVP-LANL2DZ(W)-CPCM(benzene)

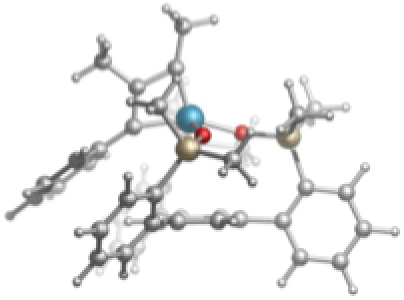

Zero-point correction= 0.731817 (Hartree/Particle)
Thermal correction to Energy= 0.782026
Thermal correction to Enthalpy= 0.782970
Thermal correction to Gibbs Free Energy= 0.650533
Sum of electronic and zero-point Energies= -2749.028774
Sum of electronic and thermal Energies= -2748.978566
Sum of electronic and thermal Enthalpies= -2748.977621
Sum of electronic and thermal Free Energies= -2749.110058

B3LYP-D3/def2TZVP-SDD(W)-CPCM(benzene)//B3LYP-D3/def2SVP-LANL2DZ(W)-CPCM(benzene)
HF = -2751.3500929

[W]-F'-TS

B3LYP-D3/def2SVP-LANL2DZ(W)-CPCM(benzene)

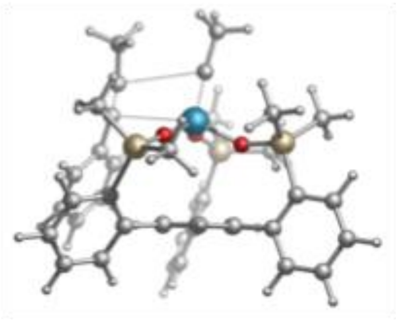

Imaginary frequency = -55.06 cm^{-1}
Zero-point correction= 0.728913 (Hartree/Particle)
Thermal correction to Energy= 0.780084
Thermal correction to Enthalpy= 0.781028
Thermal correction to Gibbs Free Energy= 0.645967
Sum of electronic and zero-point Energies= -2749.003492
Sum of electronic and thermal Energies= -2748.952322
Sum of electronic and thermal Enthalpies= -2748.951378
Sum of electronic and thermal Free Energies= -2749.086439

B3LYP-D3/def2TZVP-SDD(W)-CPCM(benzene)//B3LYP-D3/def2SVP-LANL2DZ(W)-CPCM(benzene)
HF = -2751.325516

[Mo]-Ar’

B3LYP-D3/def2SVP-LANL2DZ(Mo)-CPCM(benzene)

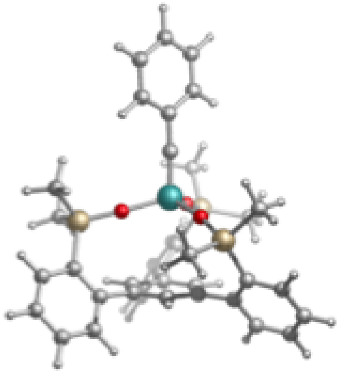

Zero-point correction= 0.642472 (Hartree/Particle)
Thermal correction to Energy= 0.688528
Thermal correction to Enthalpy= 0.689472
Thermal correction to Gibbs Free Energy= 0.560209
Sum of electronic and zero-point Energies= -2592.869563
Sum of electronic and thermal Energies= -2592.823507
Sum of electronic and thermal Enthalpies= -2592.822563
Sum of electronic and thermal Free Energies= -2592.951826
B3LYP-D3/def2TZVP-SDD(Mo)-CPCM(benzene)//B3LYP-D3/def2SVP-LANL2DZ(Mo)-CPCM(benzene)
HF = -2596.3419656

[Mo]-Ar’-TS

B3LYP-D3/def2SVP-LANL2DZ(Mo)-CPCM(benzene)

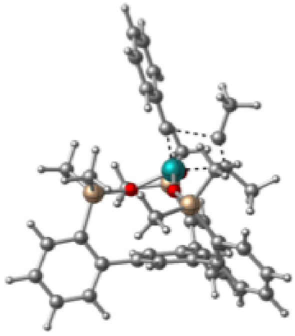

Imaginary frequency = -97.67 cm^{-1}
Zero-point correction= 0.729668 (Hartree/Particle)
Thermal correction to Energy= 0.780046
Thermal correction to Enthalpy= 0.780990
Thermal correction to Gibbs Free Energy= 0.646883
Sum of electronic and zero-point Energies= -2748.659511
Sum of electronic and thermal Energies= -2748.609133
Sum of electronic and thermal Enthalpies= -2748.608188
Sum of electronic and thermal Free Energies= -2748.742296

B3LYP-D3/def2TZVP-SDD(Mo)-CPCM(benzene)//B3LYP-D3/def2SVP-LANL2DZ(Mo)-CPCM(benzene)
HF = -2752.3941359

[Mo]-C’

B3LYP-D3/def2SVP-LANL2DZ(Mo)-CPCM(benzene)

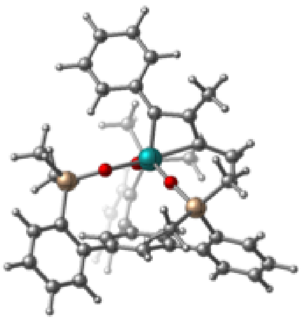

Zero-point correction= 0.731316 (Hartree/Particle)
Thermal correction to Energy= 0.781840
Thermal correction to Enthalpy= 0.782784
Thermal correction to Gibbs Free Energy= 0.648509
Sum of electronic and zero-point Energies= -2748.672521
Sum of electronic and thermal Energies= -2748.621997
Sum of electronic and thermal Enthalpies= -2748.621053
Sum of electronic and thermal Free Energies= -2748.755327

B3LYP-D3/def2TZVP-SDD(Mo)-CPCM(benzene)//B3LYP-D3/def2SVP-LANL2DZ(Mo)-CPCM(benzene)
HF = -2752.4017595

[Mo]-C'-ent

B3LYP-D3/def2SVP-LANL2DZ(Mo)-CPCM(benzene)

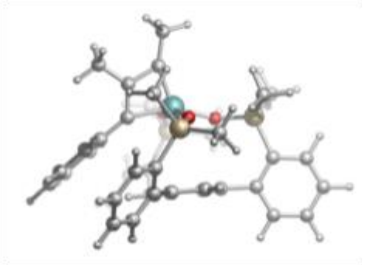

Zero-point correction= 0.727843 (Hartree/Particle)
Thermal correction to Energy= 0.780565
Thermal correction to Enthalpy= 0.781509
Thermal correction to Gibbs Free Energy= 0.639155
Sum of electronic and zero-point Energies= -2748.668908
Sum of electronic and thermal Energies= -2748.616186
Sum of electronic and thermal Enthalpies= -2748.615242
Sum of electronic and thermal Free Energies= -2748.757596

B3LYP-D3/def2TZVP-SDD(Mo)-CPCM(benzene)//B3LYP-D3/def2SVP-LANL2DZ(Mo)-CPCM(benzene)

HF = -2752.3977942

[Mo]-F'-TS

B3LYP-D3/def2SVP-LANL2DZ(Mo)-CPCM(benzene)

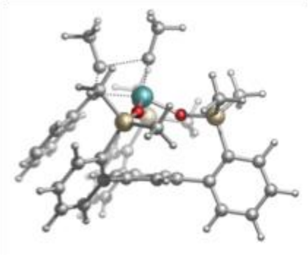

Imaginary frequency = -101.61 cm^{-1}
Zero-point correction= 0.729075 (Hartree/Particle)
Thermal correction to Energy= 0.779561
Thermal correction to Enthalpy= 0.780506
Thermal correction to Gibbs Free Energy= 0.647665
Sum of electronic and zero-point Energies= -2748.663139
Sum of electronic and thermal Energies= -2748.612653
Sum of electronic and thermal Enthalpies= -2748.611708
Sum of electronic and thermal Free Energies= -2748.744549

B3LYP-D3/def2TZVP-SDD(Mo)-CPCM(benzene)//B3LYP-D3/def2SVP-LANL2DZ(Mo)-CPCM(benzene)

HF = -2752.3955648

References

1. Trawny, D.; Quennet, M.; Rades, N.; Lentz, D.; Paulus, B.; Reissig, H., *Eur. J. Org. Chem.* **2015**, 4667-4674.
2. Gao, S.; Wu, Z.; Fang, X.; Lin, A.; Yao, H., *Org. Lett.* **2016**, *18*, 3906–3909.
3. (a) Haberlag, B.; Wu, X.; Brandhorst, K.; Grunenberg, J.; Daniliuc, C. G.; Jones, P. G.; Tamm, M., *Chem. Eur. J.* **2010**, *16*, 8868-8877. (b) Haberlag, B., Freytag, M., Daniliuc, C.G., Jones, P.G. and Tamm, M., *Angew. Chem. Int. Ed.* **2012**, *51*, 13019-13022.
4. Bittner, C.; Ehrhorn, H.; Bockfeld, D.; Brandhorst, K.; Tamm, M., *Organometallics* **2017,** *36*, 3398-3406.
5. Thompson, R. R.; Rotella, M. E.; Du, P.; Zhou, X.; Fronczek, F. R.; Kumar, R.; Gutierrez, O.; Lee, S., *Organometallics* **2019,** *38*, 4054-4059.
6. SAINT; Bruker AXS, Inc.: Madison, WI, USA. 2009.
7. Sheldrick, G. M., *SADABS;*. **2007,** University of Gottingen, Germany.
8. Sheldrick, G., SHELXT - Integrated space-group and crystal-structure determination. *Acta Cryst.* **2015,** *A71*, 3-8.
9. Sheldrick, G. M. (**2014**). SHELXL-2014. University of Gottingen, Germany.
10. a) Becke, A. D. Density-functional thermochemistry. III. The role of exact exchange. *J. Chem. Phys.*, **1993**, *98*, 5648-5652. b) Grimme, S. Semiempirical GGA-type density functional constructed with a long-range dispersion correction. *J. Comp. Chem.,* **2006**, *27*, 1787-1799. c) Weigend, F.; Ahlrichs, R. Balanced basis sets of split valence, triple zeta valence and quadruple zeta valence quality for H to Rn: Design and assessment of accuracy. *Phys. Chem. Chem. Phys.,* **2005**, *7*, 3297-3305. d) Hay, P. J.; Wadt, W. R. Ab initio effective core potentials for molecular calculations - potentials for the transition-metal atoms Sc to Hg. *J. Chem. Phys.*, **1985**, *82*, 270-283.
11. Tomasi, J.; Mennucci, B.; Cammi, R. Quantum mechanical continuum solvation models. *Chem. Rev.,* **2005**, *105*, 2999-3093.
12. a) Weigend, F. Accurate Coulomb-fitting basis sets for H to Rn. *Phys. Chem. Chem. Phys.*, **2006**, *8*, 1057-1065. b) Fuentealba, P.; Preuss, H.; Stoll, H.; Szentpály, L. V. A Proper Account of Core-polarization with Pseudopotentials - Single Valence-Electron Alkali Compounds. *Chem. Phys. Lett.*, **1982**, *89*, 418-22.
13. Mulliken, R. S., Electronic Population Analysis on LCAO–MO Molecular Wave Functions. I. *J. Chem. Phys.* **1955**, *23*, 1833-1840.
14. CYLview, 1.0b; Legault, C. Y., Université de Sherbrooke, 2009 (http://www.cylview.org)
15. Lu, T.; Chen, F., Multiwfn: A multifunctional wavefunction analyzer. *J. Comp. Chem.* **2012,** *33*, 580-592.
16. Humphrey, W.; Dalke, A.; Schulten, K., VMD – Visual Molecular Dynamics. *J. Mol. Graphics* **1996,** *14*, 33-38.
17. (a) Horn, P. R.; Head-Gordon, M. Alternative definitions of the frozen energy in energy decomposition analysis of density functional theory calculations**.** *J. Chem. Phys.* **2016**, *144*, 084118. (b) Horn, P. R.; Mao, Y.; Head-Gordon, M. Defining the contributions of permanent electrostatics, Pauli repulsion, and dispersion in density functional theory calculations of intermolecular interaction energies. *J. Chem. Phys.* **2016**, *144*, 114107. (c) Horn, P. R.; Mao, Y.; Head-Gordon, M. Probing non-covalent interactions with a second-

generation energy decomposition analysis using absolutely localized molecular orbitals. *Phys. Chem. Chem. Phys.* **2016**, *18*, 23067−23079.

18. Shao, Y.; Gan, Z.; Epifanovsky, E.; Gilbert, A. T. B.; Wormit, M.; Kussmann, J.; Lange, A. W.; Behn, A.; Deng, J.; Feng, X.; Ghosh, D.; Goldey, M.; Horn, P. R.; Jacobson, L. D.; Kaliman, I.; Khaliullin, R. Z.; Kus, T.; Landau, A.; Liu, J.; Proynov, E. I.; Rhee, Y. M.; Richard, R. M.; Rohrdanz, M. A.; Steele, R. P.; Sundstrom, E. J.; Woodcock, H. L.; Zimmerman, P. M.; Zuev, D.; Albrecht, B.; Alguire, E.; Austin, B.; Beran, G. J. O.; Bernard, Y. A.; Berquist, E.; Brandhorst, K.; Bravaya, K. B.; Brown, S. T.; Casanova, D.; Chang, C.-M.; Chen, Y.; Chien, S. H.; Closser, K. D.; Crittenden, D. L.; Diedenhofen, M.; DiStasio, R. A.; Do, H.; Dutoi, A. D.; Edgar, R. G.; Fatehi, S.; Fusti-Molnar, L.; Ghysels, A.; Golubeva-Zadorozhnaya, A.; Gomes, J.; Hanson-Heine, M. W. D.; Harbach, P. H. P.; Hauser, A. W.; Hohenstein, E. G.; Holden, Z. C.; Jagau, T.-C.; Ji, H.; Kaduk, B.; Khistyaev, K.; Kim, J.; Kim, J.; King, R. A.; Klunzinger, P.; Kosenkov, D.; Kowalczyk, T.; Krauter, C. M.; Lao, K. U.; Laurent, A. D.; Lawler, K. V.; Levchenko, S. V.; Lin, C. Y.; Liu, F.; Livshits, E.; Lochan, R. C.; Luenser, A.; Manohar, P.; Manzer, S. F.; Mao, S.-P.; Mardirossian, N.; Marenich, A. V.; Maurer, S. A.; Mayhall, N. J.; Neuscamman, E.; Oana, C. M.; Olivares-Amaya, R.; O'Neill, D. P.; Parkhill, J. A.; Perrine, T. M.; Peverati, R.; Prociuk, A.; Rehn, D. R.; Rosta, E.; Russ, N. J.; Sharada, S. M.; Sharma, S.; Small, D. W.; Sodt. Advances in molecular quantum chemistry contained in the Q-Chem 4 program package. *Mol. Phys.* **2015**, *113*, 184−215.
19. Thomas, A. A.; Speck, K.; Kevlishvili, I.; Lu, Z; Liu, P.; Buchwald, S. L. Mechanistically Guided Design of Ligands That Significantly Improve the Efficiency of CuH-Catalyzed Hydroamination Reactions. *J. Am. Chem. Soc.* **2017**, *140*, 13976 – 13984.
20. Bickelhaupt, F. M.; Houk, K. N. Analyzing Reaction Rates with the Distortion/Interaction-Activation Strain Model. *Angew. Chem., Int. Ed.* **2017**, *56*, 10070−10086.
21. Manivasagam, S.; Laury, M. L.; Wilson, A. K. Pseudopotential-Based Correlation Consistent Composite Approach (rp-ccCA) for First- and Second-Row Transition Metal Thermochemistry. *J. Phys. Chem. A* **2015**, *119*, 6867−6874.
22. For representative examples where pseudopotentials were used to describe Mo and W, refer to the following: (a) Ishiguro, Y.; Kudo, T.; Muraoka, T.; Ueno, K. Theoretical Study for the Reactions of (Silyl)(silylene)tungsten and molybdenum complexes with Ethylene Sulfide, *Organometallics* **2014**, *33*, 2704−2712. (b) Asako, S.; Ishikawa, S.; Takai, K. Synthesis of Linear Allylsilanes vis Molybdenum-Catalyzed Regioselective Hydrosilylation of Allenes. *ACS Catal.* **2016**, *6*, 3387−3395, (c) Itabashi, T.; Arashiba, K.; Tanaka, H.; Konomi, A.; Eizawa, A.; Nakajima, K.; Yoshizawa, K.; Nishibayashi, Y. Synthesis and Catalytic Reactivity of Bis(molybdenum-trihalide) Complexes Bridged by Ferrocene Skeleton toward Catalytic Nitrogen Fixation. *Organometallics* **2019**, *38*, 2863−2872. (d) Oztopcu, O.; Holzhacker, C.; Puchberger, M.; Weil, M.; Mereiter, K.;

Veiros, L. F.; Kirchner, K. Synthesis and Characterization of Hydrido Carbonyl Molybdenum and Tungsten PNP Pincer Complexes. *Organometallics* **2013**, *32*, 3042−3052. (e) Bouhoute, Y.; Garron, A.; Grekov, D.; Merle, N.; Szeto, K. C.; Mallmann, A. D.; Rosal, I. D. Maron, L., Girard, G.; Gauvin, R. M.; Delevoye, L.; Taoufik, M. Well-Defined Supported Mononuclear Tungsten Oxo Species as Olefin Metathesis Pre-Catalysts. *ACS Catal.* **2014**, *4*, 4232−4241. (f) Chen, P.; Zhang, L.; Xue, Z.; Wu, Y.; Zhang, X. Density Functional Theory Study of the Reaction between d^0 Tungsten Alkylidyne Complexes and H_2O: Addition versus Hydrolysis. *Inorg. Chem.* **2017**, *56*, 7111−7119.
